# Klinefelter syndrome and ADHD: a short systematic review

**DOI:** 10.3389/fpsyt.2025.1585259

**Published:** 2025-05-29

**Authors:** Lorenzo Zamboni, Francesca Fusina, Rebecca Casari, Fabio Lugoboni, Thomas Zandonai, Sara Cappelletti, Angela Federico

**Affiliations:** ^1^ Department of Internal Medicine, Unit of Addiction Medicine, G.B. Rossi Hospital, Verona, Italy; ^2^ Department of General Psychology, University of Padova, Padua, Italy; ^3^ Padova Neuroscience Center, University of Padova, Padua, Italy; ^4^ Department of Pharmacology, Paediatrics and Organic Chemistry, Miguel Hernández University of Elche, Elche, Spain; ^5^ Addiction Science Laboratory, Department of Psychology and Cognitive Science, University of Trento, Rovereto, Italy; ^6^ Socio-Healthcare Institution, Centro Trentino di Solidarietà Ente del terzo settore (ETS), Trento, Italy

**Keywords:** Klinefelter syndrome, ADHD (attention deficit and hyperactivity disorder), XXY (aneuploidy of Klinefelter syndrome), ADHD adulthood, attention deficit hyperactivity disorder

## Abstract

Klinefelter Syndrome (KS) is the most common sex chromosome anomaly in the world, and it is characterized by the presence of an extra X chromosome (47, XXY). It affects approximately 1 in 500 to 1 in 1000 male births and it has been associated with various neurodevelopmental conditions, including Attention Deficit Hyperactivity Disorder (ADHD). This systematic review aims to collect and analyze existing literature on the comorbidity of KS and ADHD. A comprehensive search was conducted following PRISMA guidelines in Medline and Web of Science, covering studies from 1959 to 2024. After screening and applying inclusion/exclusion criteria, 15 studies were selected for analysis. The findings suggest a significant association between KS and ADHD, particularly concerning the inattentive subtype. Several studies reported an increased prevalence of ADHD in individuals with KS compared to the general population, with estimates ranging from 25% to 63%. Some research also indicated a higher risk for comorbid conditions such as Autism Spectrum Disorder (ASD) and mood disorders. Neuroimaging studies highlighted structural and functional differences in KS patients compared with controls, particularly in brain regions involved in executive function, working memory, and attention regulation. Despite these findings, no study provided conclusive evidence regarding a causal relationship between KS and ADHD. Additionally, pharmacological treatment for ADHD appears to be effective and well-tolerated in KS patients, with minimal side effects. Overall, this review underscores the complex and heterogeneous nature of KS-ADHD comorbidity; however, variability in study populations and methodologies limits the ability to draw definitive conclusions. Further research is necessary to clarify the potential mechanisms linking KS and ADHD and to develop tailored clinical approaches for individuals affected by both conditions.

## 1 Introduction

In 1942, Klinefelter and colleagues published in a case series in which they described 9 men who had enlarged breasts, sparse facial and body hair, small testes, and an inability to produce sperm. This problematic disease was named Klinefelter syndrome (KS). KS patients were discovered to have an extra X chromosome (genotype XXY) instead of the usual male sex complement (genotype XY) (1). KS can manifest in a classical form, which is present in 80–90% of cases, which is defined by a 47, XXY karyotype resulting from the aneuploidy of the sex chromosomes, whereas higher-grade aneuploidies (e.g. 48,XXXY or 48,XXYY), structurally abnormal X chromosomes (e.g. 47,iXq,Y) or mosaicisms (e.g. 47,XXY/46,XY) make up for the remaining 10–20% of cases. The prevalence of KS (ranging from 0.1 to 0.2% in newborn male infants) rises up to 3–4% among infertile males and 10–12% in azoospermic patients (1), and it is the most frequently observed sex chromosomal anomaly, with an estimated frequency of 1:500 to 1:1000 men (2). The incidence of KS increased in the last few years (3) albeit in the absence of a concomitant rise in the prevalence of XXY aneuploidy. This may indicate that the rise of KS might be related to an increase in paternal meiotic alterations. KS patients have an extremely variable phenotype, which however lacks any obvious facial dysmorphology. This makes them indistinguishable from boys with a normal karyotype.

Several MRI studies concerning the phenotypic presentation of XXY revealed decreased gray and white brain matter consistent with deficits in executive functioning (EF) and frontal functioning. These deficits include low cognitive flexibility, difficulties in working memory, planning, inhibition, and ADHD (4–7).

To date, there are several reviews that underline the social and cognitive implications of KS.

Concerning cognitive dysfunctions associated with KS, these include deficits in executive functioning, impairments in both receptive and expressive language abilities, a higher prevalence of psychiatric disorders such as anxiety and depression, and significant social difficulties that can affect interpersonal relationships and overall social adaptation. Additionally, individuals with KS may experience a range of other associated challenges, which can vary in severity and manifest differently across different stages of life (8).

Notwithstanding these difficulties, most individuals with Klinefelter syndrome (KS) do not experience severe educational difficulties and are capable of achieving academic success. Their general cognitive abilities are comparable to those of the average population, with a mean IQ ranging from 87.9 to 110. However, boys with KS may exhibit lower verbal IQ scores compared to their non-affected peers (9, 10).

Dyslexia is also reported among learning deficits in this population, although the prevalence of this condition varies significantly depending on cohort selection and individual patient characteristics. Most boys with KS will require regular reassessment of their academic and cognitive abilities to identify those who may need additional specialized educational support (10, 11).

Ross et al. (12) reported that deficits in sustained attention manifest as prolonged and inconsistent reaction times, along with lapses in attention, such as omissions or missed targets. Subsequent studies using continuous performance tasks have confirmed these impairments (13–15), demonstrating that the effect sizes of attention deficits surpass those associated with executive dysfunction (16, 17). This suggests that failures in sustained attention may serve as a critical limiting factor in the impaired higher-order executive functions observed in individuals with an XXY karyotype.

Furthermore, attention deficits associated with KS have shown to follow an age-related distribution (18). Specifically, younger boys (aged 4–9 years) exhibited a diminished ability to maintain attention, which appeared to improve with age—by adolescence (10–18 years), their performance aligned with that of the normative sample. Additionally, inattention symptoms are frequently observed in daily life assessments of boys with KS, as indicated by the rating scales used to assess inattention in the various studies considered (16, 19). In particular, signs of inattention were shown to be detectable even in toddlerhood (20).

Concerning higher-order attention control, executive function (EF)—which encompasses self-regulatory and cognitive control processes—is frequently impaired in individuals with KS. However, the severity of these deficits varies considerably across studies. Cognitive assessments indicate a diminished ability to inhibit responses (14, 19, 21), though some studies report exceptions (16, 18). Additionally, some studies highlight deficits in working memory, reduced mental flexibility and difficulties in problem-solving and planning (13, 14, 16, 22–26).

While performance-based EF tests help predict academic success (27), behavioral rating scales, which offer stronger ecological validity (28), provide further insight into real-world self-regulation challenges in KS patients; however, only a limited number of studies have incorporated these in their research design. Findings indicate significant deficits across multiple EF subcomponents, including both cognitive and behavioral control (14, 29, 30), with early childhood precursors of executive dysfunction already apparent (31). In childhood, EF impairments may be relatively subtle, typically just over one standard deviation from the normative mean, but they tend to become more pronounced with age (32), likely due to increasing demands for self-regulation.

Attention deficit and hyperactivity disorder (ADHD) is one of the most common mental health disorders in childhood and adolescence (33). While it was traditionally perceived as a neurodevelopmental disorder first diagnosed in childhood and considered by many to resolve during adolescence and young adulthood (34), estimates of ADHD prevalence in the general population range from 6% to 9% in children to 2.5–4% in adults; indeed, longitudinal data suggests that two-thirds of the childhood ADHD cases persist into adulthood (35, 36).

Symptomatology can change over time (from childhood to adulthood); hyperactivity seems to diminish, while inattention and emotional problems prevail or even become more serious (37, 38). The prototypic symptom pattern that can be observed in adult ADHD (39) comprises concentration problems and inattention, mind wandering, problems staying on task or keeping deadlines, as well as impulsive behavior, restlessness, and difficulty regulating emotions triggered by external stimuli (40).

ADHD is a complex phenomenon, and comprises three subcategories: Combined presentation (F90.2), Predominant inattention (F90.0), Predominant hyperactivity/impulsivity (F90.1) (41).

Subjects with ADHD may incur in several difficulties in their daily lives, for example concerning relationships, maintaining a job, attention problems, car accidents, difficulties with anger control, problems with the law, depression and anxiety, addiction problems and suicide (42).

Evidence suggests that attention deficit/hyperactivity disorder (ADHD) is prevalent among individuals with an XXY karyotype, with approximately 43% of children and adolescents with KS meeting the diagnostic criteria also for ADHD (43). This prevalence is significantly higher than the estimated global rate of 5% to 7% among the general pediatric population (33, 44), indicating a markedly increased risk of ADHD and associated comorbidities in individuals with KS (45, 46). The inattentive subtype appears to be the most commonly observed (16, 19, 47), and when ADHD is present alongside KS, it further exacerbates executive function (EF) deficits and behavioral ratings (6).

Sustained attention and EF play a critical role in adaptive, goal-directed behavior in everyday life (48). Enhancing self-regulation could therefore have substantial benefits for individuals with KS. Although EF skills are strongly influenced by verbal support (49) and verbal ability is a recognized area of weakness in KS, no direct correlation between verbal impairments and EF deficits has been firmly established (16, 50). A deeper understanding of the factors underlying executive dysfunction in KS could greatly improve efforts to mitigate these challenges.

Recent findings have identified a neural basis for EF deficits in KS, suggesting a connection between the severity of these impairments and the condition’s atypical pubertal development (15). However, the impact of testosterone replacement therapy (TRT) or psychostimulants such as methylphenidate on key aspects of EF—sustained attention, initiation, impulsivity, motivation, and overall behavioral control—remains largely unknown and requires further research. Preliminary evidence, however, points to a potential positive effect of hormonal replacement therapy on EF in individuals with KS (51).

Finally, the extent to which ADHD characteristics in 47, XXY boys overlap with those observed in 46, XX girls is still uncertain. Since ADHD in females is often characterized by inattentive symptoms and internalizing behaviors that are frequently overlooked in diagnostic evaluations (52), refining clinical guidelines for KS to account for these nuances could improve diagnostic accuracy and treatment strategies.

There are several overlapping brain dysfunctions between Attention-Deficit/Hyperactivity Disorder (ADHD) and Klinefelter Syndrome (KS), particularly in regions associated with executive functions.

In individuals with ADHD, neuroimaging studies have consistently identified abnormalities in the prefrontal cortex, anterior cingulate cortex, and frontostriatal circuits. These areas are critical for attention regulation, impulse control, and executive functioning. Specifically, reductions in the size of the dorsolateral prefrontal cortex and anterior cingulate gyrus have been observed, correlating with deficits in goal-directed behavior and attention control (53).

Similarly, individuals with KS exhibit structural and functional brain differences in comparable regions. Studies have shown reduced gray matter volumes in the left temporal lobe and alterations in the frontal lobe, including the inferior frontal gyrus and anterior cingulate cortex. These changes are associated with impairments in language, executive functions, and social cognition (54, 55).

The convergence of these findings suggests that both ADHD and KS involve dysfunctions in neural circuits responsible for executive control and attention regulation. This overlap may contribute to the increased prevalence of ADHD symptoms observed in individuals with KS (45).

Further research is warranted to elucidate the shared neurobiological mechanisms underlying these conditions and to inform targeted interventions.

In conclusion KS (47,XXY) in males and Trisomy X syndrome (47,XXX) in females are characterized by an additional X chromosome and share several neurodevelopmental and cognitive features, significant differences in symptom presentation have been observed between genders.

Males with 47,XXY typically exhibit: hypogonadism and infertility, tall stature with longer limbs, language-based learning difficulties, executive function impairments and increased risk for psychiatric comorbidities such as ADHD and depression. Females with 47,XXX generally present with: mild cognitive and language delays (often less severe than in XXY), taller-than-average height, relatively preserved fertility, increased risk of internalizing symptoms (e.g., anxiety, social withdrawal), rather than externalizing behaviors.

Moreover, the severity and visibility of symptoms tend to be higher in males with XXY than in females with XXX, likely due to differences in hormonal and neurodevelopmental processes (56).

The aim of this systematic review is to provide a comprehensive collection of the existing literature regarding KF and ADHD.

## 2 Materials and methods

Preferred reporting items for systematic review and meta‐analysis (PRISMA) reporting guidelines were used in the development of this systematic review (57).

### 2.1 Eligibility criteria

Studies needed to meet the following criteria:

- Targeted client populations with ADHD an KS
- English language;
- Study years 1959 – 2024;
- RCTs;
- Quasi – RCTs;
- Pilot studies;
- Reviews with only RCTs studies included.

Exclusion criteria were:

- Studies did not assess ADHD – related outcomes;
- Qualitative studies;
- Published abstracts;
- Case reports and case series;
- Missing outcomes or results;

### 2.2 Information sources and search strategy

A literature search was conducted on Medline and Web of Science.

The reference lists of retrieved studies were assessed for further relevant publications. A search strategy was developed from Medical Subject Headings (MeSH) and text words related to patients with Klinefelter Syndrome and ADHD from 1959 to 31 December 2024. Two authors (L.Z. and A.F.) independently screened titles and abstracts identified through this search strategy. Rayyan was used to exclude duplicates and predetermined selection criteria were used to assess eligibility. An author (T.Z.) independently supervised the selection of papers made by the previous two authors. Final eligibility required the agreement of both reviewers.

### 2.3 Study selection

We used a search string: (“klinefelter syndrome” OR 47,XXY OR “47,XYY syndrome” OR “47,XXY Klinefelter syndrome”) AND (ADHD OR “attention deficit hyperactivity disorder” OR “attention-deficit/hyperactivity disorder” OR XXY). All results are collected in 3 files that were included in this paper’s **Supplementary Material**.

### 2.4 Data collection

Data extraction was applied for observational studies examining the prevalence of Klinefelter Syndrome in ADHD comorbidities.

### 2.5 Role of the funding source

There was no funding source for this study.

## 3 Results

**Figure 1:**
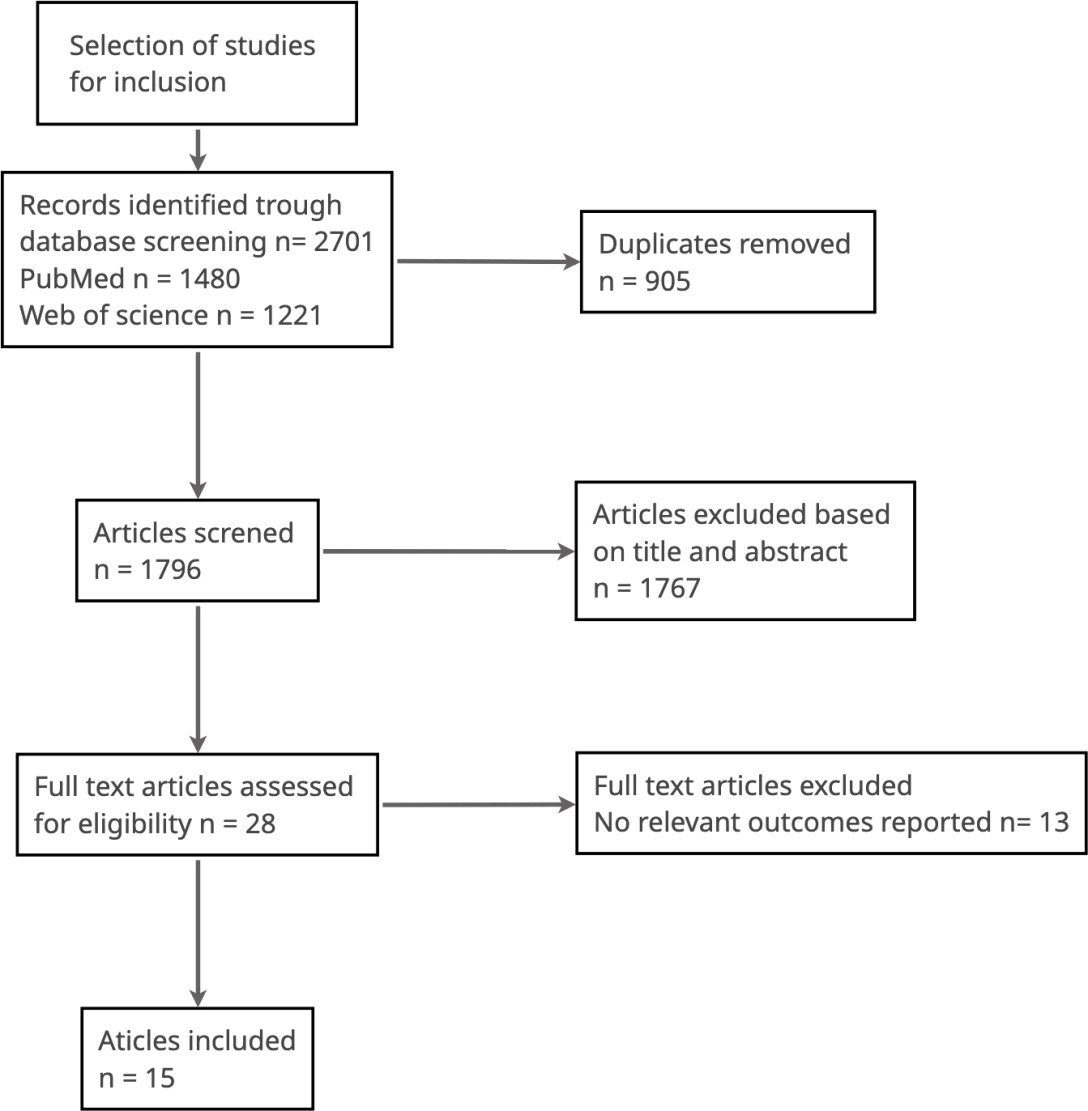
Selection of studies for inclusion.

We identified 2701 studies using MEDLINE and EMBASE. After duplicates were removed, 1796 studies remained. A review of the title and abstracts excluded a further 1767 studies. Of the remaining 28 articles assessed for eligibility, 13 were excluded because they did not report on outcomes relevant to this systematic review. 15 studies met the inclusion criteria (**Figure 1** and **Table 1**).

**Table 1:** Paper included in this review.

Paper	Chromosome Anomaly	Age	Symptoms	Brain/Hormonal Correlates
Boada et al. (58)	47,XXY (Klinefelter syndrome)	Childhood to adulthood	Language and reading disorders, executive dysfunction, verbal IQ < performance IQ	Hormonal: hypogonadism; Genetic: extra X chromosome affects cognitive phenotype
Bruning et al. (46)	47,XXY	6–19 years	Language disorder (65%), ADHD (63%), Autism Spectrum Disorder (27%)	Neurodevelopmental phenotype related to extra X chromosome
Cederlöf et al. (45)	47,XXY	Not specified	Higher risks of schizophrenia, bipolar disorder, autism spectrum disorder (OR=6.2), ADHD (OR=5.6)	X-linked contributions to psychiatric disorders
de Vries et al. (59)	47,XXY	Adults (mean 32.1 years)	Elevated rates of anxiety, depression, ADHD, autism	Increased psychiatric morbidity, self-esteem and body image affected
Green et al. (24)	45,X (Turner), 47,XXY (Klinefelter)	3–11 years	Attention problems, working memory deficits	X chromosome dosage affects attention, hyperactivity, working memory
Hong & Reiss (60)	Various SCAs incl. 47,XXY	Not specified	Motor and intellectual impairments, high rates of psychiatric disorders	Dose effect of sex chromosome genes on neuroanatomy and cognition
Lee et al.	47,XXY	9–25 years	Deficits in executive function, especially verbal working memory	ADHD comorbidity worsens EF impairments
Lo-Castro et al. (61)	47,XXY	Children	High ADHD prevalence in KS and other syndromes	Shared neurobiological circuits involved in ADHD across genetic syndromes
Printzlau et al. (62)	Sex chromosome aneuploidy	Not specified	Deficits in social communication, emotional regulation, cognition	X-linked gene dosage effects, altered brain development
Samango-Sprouse (63)	47,XXY	Children	ADHD, social communication problems, learning dysfunction	Behavioral phenotype modulated by familial learning disabilities
Skakkebæk et al. (64)	47,XXY (Klinefelter syndrome)	Childhood to adulthood	Verbal deficits, executive dysfunction, increased anxiety, depression, ADHD symptoms	Smaller brain volume, altered frontal lobe structure, functional changes in auditory, motor, language, social processing
Tartaglia et al. (17)	47,XXY	6–21 years	Developmental delays, ADHD, autism spectrum features, social-emotional difficulties	No direct biomarkers reported; behavioral assessments linked to phenotype
Tartaglia et al. (65)	47,XXY, XXX, XYY, XXYY	6–20 years	ADHD symptoms: 36% (XXY), 52% (XXX), 76% (XYY), 72% (XXYY)	SCA-related differences in attention linked to gene dosage; adaptive functioning lower in ADHD group
Van Rijn et al. (14)	47,XXY and 47,XXX	9–18 years	Executive dysfunction, behavioral problems, aggression, thought problems	Frontal lobe dysfunction; neuroimaging shows altered structure and function
Van Rijn (43)	47,XXY, 47,XXX, 47,XYY	Children and adults	Autism symptoms (12–47% in XXY), ADHD (27–63% in XXY), anxiety, depression, psychotic symptoms	Neurocognitive deficits in executive function, emotion regulation, social cognition; linked to extra sex chromosome

Cederlöf et al. (45), used the National Patient Register to identify 860 KS and ADHD patients coded according to the ICD-10. The results underline that KS patients present a six times higher risk of autism spectrum disorders and ADHD compared with a control group.

Moreover, the authors suggest that their data underlines a possible causal role of X chromosomal abnormalities in the vulnerability for autism spectrum disorders and ADHD, and they encourage further investigations.

A study by Tartaglia et al. (17) was conducted on a sample of 57 children and adolescents aged between 6 to 21 years old, with a karyotype of 47, XXY. Participants were recruited for a study on health and development on all forms of supernumerary sex chromosome aneuploidy through national advocacy groups for males with a XXY karyotype and through clinics in developmental pediatrics, endocrinology, genetics, and general pediatrics at the UC-Davis MIND Institute and The Children’s Hospital in Denver from 2005–2010.

Subjects were determined to meet criteria for ADHD if they were noted to have moderate to severe symptoms in 6 of the 9 inattentive items (ADHD-Inattentive subtype), or in 6 of the 9 items in both inattentive and hyperactive/impulsive domains (ADHD-Combined subtype).

The results of this study show that 36% of the sample met criteria for a diagnosis of ADHD based on parent reports. Strikingly, all but one of those who met DSM-IV criteria were classified as having the Inattentive subtype (previously known as ADD), without significant symptoms of hyperactivity and impulsivity.

A study with the aim of exploring sex differences in ADHD symptomatology, probed whether X chromosome absence or excess was independently associated with deficits in attention and hyperactivity, executive function, and processing speed. In this sample, 20 males with KS were recruited through pediatric endocrinologists, medical geneticists, the national TurnerSyndrome Society network, the Association for X and Y Chromosome Variations, and the Center for Interdisciplinary Brain Sciences Research website.

This study shows that 25% of males with KS were parent-reported as having attention problems while 40% had hyperactivity. Indeed, males with Klinefelter Syndrome displayed increased ADHD symptoms and weaknesses in executive functions relative to typically developed comparison groups (24).

A European large multicenter cross-sectional clinical evaluation study by de Vries et al. (59), highlighted that KS patients appear to be the most vulnerable group of the sample. Not only were their autistic and attention difficulties higher compared with the other conditions (depression, anxiety disorders, schizophrenia, autism and other mental health), but this was also the case for their levels of depression and anxiety. Their self-reported anxiety, depression, ADHD, and autism symptom levels were also higher when compared with the French reference population or population prevalence rates. This study, however, presents several limitations: in particular, it is important to underline that it only used retrospective psychiatric history data and psychiatric symptom screening measures, and no validated diagnostic instruments.

A study that included 51 boys with a mean age of 12.2 years (range 6–19 years) had the aim of exploring the extent of psychiatric morbidity in children with Klinefelter Syndrome (46). This research consisted in the administration of the K-SADS-PL interview, and had the following results: 63% (32 of 51) of the subjects were classified as having ADHD, of which 43% (22 of 51) could be classified as having the predominantly inattentive subtype, and 20% (10 of 51) were classified as having the combined subtype. This group presented an overlap with Autism Spectrum Disorder (ASD) in 27,45% of cases. This study highlights the increased percentage of ADHD in the KS population compared to the general population.

A review by Boada and colleagues (58), emphasized that several studies reported increased rates of ADHD and attentional problems in clinical samples of male children with an XXY karyotype, but they didn’t reach any conclusion about this data. Indeed, a mini-review by Printzlau et al. (62) confirmed a comorbidity of ADHD and ASD for subjects with KS (range between 36% and 63%).

A review by Lo Castro et al. (61) only evaluated studies that included subjects with ADHD and KS. The paper suggests a possible protective effect of neurosteroids, which may be lost in sexual aneuploidies because of gonadal dysfunction, thus predisposing people with KS to develop ADHD.

A review by Hong and Reiss (60) examined human studies from 2003 to 2014 to sum up the main clinical features of aneuploidies of sex chromosomes. About the KS population, the authors reported an increased risk for ADHD, especially concerning the inattentive domain. The authors report that neuroimaging data confirms the clinical data: several anatomical imaging studies have shown higher grey-matter volume in the parieto-occipital and sensorimotor cortices, and lower volume in the insula and temporal regions. There are also several studies that show compromised brain structures in the prefrontal cortex, cerebellum, and lateral ventricles; however, until 2014 only two fMRI studies concerning these patients had been conducted.

Another review explored a possible profile of KS (64). The authors confirm the high prevalence of ADHD among males with KS according to the DSM-IV diagnostic criteria and based on parent reports.

About the neuroanatomical and functional brain alterations seen in patients with KS, the authors hypothesize that they are most likely the result of both genetic and hormonal factors. However, they stress that the neurobiological underpinnings of the cognitive, psychological, social and behavioral phenotypes seen in KS have not been established yet and should be further addressed in future studies.

Van Rijn (66) published a review that investigate to the neurocognitive functioning and risk for psychopathology in sex chromosome trisomies.

The presence of ADHD symptoms was found, on average, in 35% of children with XXY (especially concerning the inattentive domain). When considering the full diagnostic classification of ADHD, 43% of the children and adolescents met diagnostic criteria for ADHD, on average.

An interesting fact is that, in adults with an XXY karyotype, ADHD was diagnosed 5.6 times more frequently according to a screening of hospital discharges in 860 adults with XXY as compared with 86.000 men with XY.

A retrospective study on 54 boys with KS, ADHD had an incidence of about 12.5% in the sample.

In this study, for all subjects, KS were referred by their physicians, parents and ancillary health care, and the respective diagnoses were confirmed through karyotype analysis and documented in the children’s medical records (63).

A large sample study by Tartaglia et al. (65), enrolled males with XXY, XYY, and XXYY and females with XXX between the ages of 6 and 20 years, of all races and ethnicities. This study compared these specific groups in relation to ADHD symptomatology. Participants were treated from 2004 to 2010 by a developmental-behavioral pediatrician (N.R.T.) at the University of California. The results present 58% (96/167) subjects with ADHD symptomatology, in this sample, while 36% present KS in comorbidities, with the total ADHD rate in these groups almost entirely due to the Inattentive subtype. Comparing overall ADHD rates yielded a significant difference between XXY and the rest of the groups, with the XXY group exhibiting a lower rate of scores in the ADHD range (any subtype).

XXY group presented a lower rate of hyperactive/impulsive symptoms than did children of XYY and XXYY groups.

About inattention, the XXY group had fewer symptoms than the XYY group, while XXX and XXYY had intermediate levels of these symptoms; however, the XXY, XXX, and XXYY groups did not differ from one another. For hyperactivity/impulsivity and combined scores, the XXY and XXX groups had fewer symptoms than XYY, with XXYY on an intermediate level and no different from XXX and XYY.

The paper adds that individuals that are prenatally diagnosed with KS exhibit ADHD less frequently compared to those diagnosed after birth.

Patients previously diagnosed with ADHD who do not seem to respond to treatment may have increased rates or severity of these comorbid factors, which may not have been fully appreciated or addressed. Furthermore, comorbid learning and social-emotional symptoms, just like ADHD symptoms, often present differently across the lifespan, so periodic reevaluation may be necessary.

Finally, the results of a review by Tartaglia et al. (65) that addressed the topic of medication show that standard stimulant treatment is effective in more than 70% of children and adolescents with SCA and ADHD. The treatment is also considered safe, with a relatively low rate of significant side effects.

Van Rijn and Swaab (14) have published a study which assess the executive domain and the emotional and behavioral functioning of patients (boys and girls, from 9 to 18 years old) with sex chromosome trisomies.

The patients (40) and non-clinical group (100) underwent cognitive test and the results demonstrated that the group of children with an extra X chromosome showed more difficulties in the areas of inhibition, mental flexibility, sustained attention and working memory. These results are in line with the parental reports about the children’s everyday life. There were no differences between males and females in the severity of executive function impairments, the only difference being information processing speed, with the girls being slower than boys.

Lee and colleagues (6) published a study with the aim to determine the impact of ADHD in the executive performance of XXY patients. Comparisons of the XXY groups with and without ADHD to the control groups indicated that the comorbid group was the most compromised.

The presence of the ADHD comorbidity in males with XXY was correlated to scarce EF skills.

## 4 Discussion

ADHD in childhood and adult age is a complex neurodivergence which presents different characteristics, not only in the attention and hyperactivity domains but also in relationships, mood regulation, low self-esteem etc. (37, 38).

Neurodiversity extends beyond cognitive differences and specific recognized neurotypes patterns of neurological structure and function shared by certain groups (67). While it does include identified neurotypes such as autism, attention deficit/hyperactivity disorder and dyslexia, it is not confined to them (68).

Neurodivergent individuals generally display cognitive and neurological variations that differ from what is typically considered the norm (69). However, defining and understanding neurodiversity is complex. Some scholars conceptualize it as a social ecosystem of cognitive functions (70), while others liken it to biodiversity in nature (71). Additionally, perspectives on neurodiversity vary: some view it as a political identity rather than a biological classification (70, 72), while others regard it as a biological impairment in contrast to neurotypical behavior.

These differing viewpoints can inadvertently diminish the experiences of neurodivergent individuals, either by framing neurodivergence as an artificial identity or reducing it solely to a set of deficits, neglecting the strengths and distinctive attributes of those who experience it. The discourse surrounding neurodiversity remains highly debated, with multiple definitions being proposed and contested.

ADHD is increasingly being examined through the lens of neurodiversity, with research focusing on two key objectives: (i) enhancing the understanding of ADHD and (ii) redefining its practical implications in both daily life and professional settings (73). Sonuga-Barke (74) challenge the dominant biomedical framework that has traditionally shaped ADHD research and treatment. Instead, the author advocates for a neurodiversity-affirming model that offers a more inclusive perspective.

This approach introduces an innovative intervention program designed to be implemented by neurodivergent researchers. Beyond challenging conventional paradigms, it actively engages neurodivergent individuals in both the design and execution of research, ensuring that interventions are tailored to their lived experiences and specific needs.

In light of all the aforementioned aspects, the landscape of neurodivergence, of which ADHD is a part, is complex and continuously evolving.

This review included 15 studies, which considered KS in comorbidity with ADHD.

Indeed, ADHD and KS present high rates of comorbidities, particularly with autism spectrum disorders (46, 66).

Several papers examined in this review underlined ADHD in comorbidity with KS (24, 58, 59) but they did not infer a causality relation. Indeed, KS could increase the risk of developing ADHD, in especially for the inattentive domain (75). Subjects with KS appear to be the most vulnerable group not only for ADHD and autistic spectrum disorders, but also for mood disorders (59).

One paper underlined that drug treatment for ADHD symptomatology could be the same with and without KS in comorbidity, with low side effects. This highlights the relevance of drug treatment for this neurodivergence, which could be useful to manage symptoms in order to prevent an increase in its severity (65).

In conclusion, the analysis of the selected papers shows an extremely heterogeneous sample of studies. Therefore, we cannot currently confirm or refute the objective of this review.

This review presents several limitations: a) we have consulted only two dataset (MedLine and EMBASE) b) we included heterogenous studies c) studies with significant results are more likely to be published, while those with null or negative findings are often overlooked, which could influence the conclusions d) we exclusively included data from studies published in indexed journals. While this criterion ensures a certain level of quality, it may also have resulted in the exclusion of potentially valuable data e) Differences in methods, sample characteristics, or diagnostic criteria among the included studies may make it challenging to draw general conclusions.

## 5 Conclusion

This review looked at research discussing ADHD alongside KS to systematically collect all available evidence as of yet.

The evidence we found is inconclusive to confirming or refuting a relationship between KS and ADHD, due to the diverse and non-overlapping populations in the studies. As a result of this diversity in the ADHD and KS populations, it is difficult to draw generalized conclusions about their correlation. Further research is necessary to better understand how ADHD and KS may be related. Moreover, this evaluation illustrates how individuals with both KS and ADHD often display variations in neurodivergent behavior. This results in an intricate clinical phenotype that calls for diverse and tailored approaches to guarantee suitable care for those affected by these conditions.
