# Supplementary material for: Klinefelter syndrome and ADHD: a short systematic review
Source: Front Psychiatry. 2025 May 29;16:1585259. doi: 10.3389/fpsyt.2025.1585259 (PMC12158946; doi:10.3389/fpsyt.2025.1585259)
Supplement: Supplementary file 1 [file DataSheet1.docx]

PMID- 30755791

OWN - NLM

STAT- MEDLINE

DCOM- 20200605

LR - 20200605

IS - 2046-1402 (Electronic)

IS - 2046-1402 (Linking)

VI - 8

DP - 2019

TI - Recent advances in managing and understanding Klinefelter syndrome.

LID - F1000 Faculty Rev-112 [pii]

LID - 10.12688/f1000research.16747.1 [doi]

AB - Klinefelter syndrome can present as a wide spectrum of clinical manifestations at

various stages in life, making it a chromosomal disorder with no standardized set

of guidelines for appropriate management. Understanding the genetic and hormonal

causes of this syndrome can allow physicians to treat each patient on a more

individualized basis. The timing of diagnosis and degree of symptoms can guide

management. This report will provide an updated review of the clinical

presentation at various stages in life and the implications for management.

FAU - Bearelly, Priyanka

AU - Bearelly P

AD - Urology, Boston University School of Medicine, 725 Albany Street, Suite 3B,

Boston, MA, 02118, USA.

FAU - Oates, Robert

AU - Oates R

AUID- ORCID: 0000-0003-2330-6697

AD - Urology, Boston University School of Medicine, 725 Albany Street, Suite 3B,

Boston, MA, 02118, USA.

LA - eng

PT - Journal Article

PT - Review

DEP - 20190128

TA - F1000Res

JT - F1000Research

JID - 101594320

SB - IM

MH - Humans

MH - Klinefelter Syndrome/*diagnosis/*therapy

PMC - PMC6352920

OTO - NOTNLM

OT - *47

OT - *Klinefelter Syndrome

OT - *TESE

OT - *XXY

OT - *azoospermia

OT - *testis

COIS- No competing interests were disclosed.No competing interests were disclosed.No

competing interests were disclosed.

EDAT- 2019/02/14 06:00

MHDA- 2020/06/06 06:00

CRDT- 2019/02/14 06:00

PHST- 2019/01/22 00:00 [accepted]

PHST- 2019/02/14 06:00 [entrez]

PHST- 2019/02/14 06:00 [pubmed]

PHST- 2020/06/06 06:00 [medline]

AID - F1000 Faculty Rev-112 [pii]

AID - 10.12688/f1000research.16747.1 [doi]

PST - epublish

SO - F1000Res. 2019 Jan 28;8:F1000 Faculty Rev-112. doi:

10.12688/f1000research.16747.1. eCollection 2019.

PMID- 23810129

OWN - NLM

STAT- MEDLINE

DCOM- 20131205

LR - 20211021

IS - 1097-6833 (Electronic)

IS - 0022-3476 (Print)

IS - 0022-3476 (Linking)

VI - 163

IP - 4

DP - 2013 Oct

TI - 47,XYY syndrome: clinical phenotype and timing of ascertainment.

PG - 1085-94

LID - S0022-3476(13)00586-6 [pii]

LID - 10.1016/j.jpeds.2013.05.037 [doi]

AB - OBJECTIVE: To describe auxologic, physical, and behavioral features in a large

cohort of males with 47,XYY (XYY), ages newborn to young adult. STUDY DESIGN:

This is a cross-sectional descriptive study of male subjects with XYY who were

evaluated at 1 of 2 specialized academic sites. Subjects underwent a history,

physical examination, laboratory testing, and cognitive/behavioral evaluation.

RESULTS: In 90 males with XYY (mean age 9.6 ± 5.3 years [range 0.5-36.5]), mean

height SD was above average (1.0 ± 1.2 SD). Macrocephaly (head circumference >2

SD) was noted in 28/84 (33%), hypotonia in 57/90 (63%), clinodactyly in 47/90

(52%), and hypertelorism in 53/90 (59%). There was testicular enlargement for age

(>2 SD) in 41/82 (50%), but no increase in genital anomalies. No physical

phenotypic differences were seen in boys diagnosed prenatally vs postnatally.

Testosterone, luteinizing hormone, and follicle stimulating hormone levels were

in the normal range in most boys. There was an increased incidence of asthma,

seizures, tremor, and autistic spectrum disorder (ASD) compared with the general

population rates. Prenatally diagnosed boys scored significantly better on

cognitive testing and were less likely to be diagnosed with ASD (P < .01).

CONCLUSIONS: The XYY phenotype commonly includes tall stature, macrocephaly,

macroorchidism, hypotonia, hypertelorism, and tremor. Physical phenotypic

features were similar in boys diagnosed prenatally vs postnatally. Prenatal

diagnosis was associated with higher cognitive function and less likelihood of an

ASD diagnosis.

CI - Copyright © 2013 Mosby, Inc. All rights reserved.

FAU - Bardsley, Martha Zeger

AU - Bardsley MZ

AD - Division of Endocrinology, Department of Pediatrics, Thomas Jefferson University,

Philadelphia, PA; Department of Pediatrics, Nemours and duPont Hospital for

Children, Wilmington, DE.

FAU - Kowal, Karen

AU - Kowal K

FAU - Levy, Carly

AU - Levy C

FAU - Gosek, Ania

AU - Gosek A

FAU - Ayari, Natalie

AU - Ayari N

FAU - Tartaglia, Nicole

AU - Tartaglia N

FAU - Lahlou, Najiba

AU - Lahlou N

FAU - Winder, Breanna

AU - Winder B

FAU - Grimes, Shannon

AU - Grimes S

FAU - Ross, Judith L

AU - Ross JL

LA - eng

GR - UL1 TR000154/TR/NCATS NIH HHS/United States

GR - UL1 TR001082/TR/NCATS NIH HHS/United States

GR - 1K23NS070337/NS/NINDS NIH HHS/United States

GR - K23 NS070337/NS/NINDS NIH HHS/United States

GR - L40 HD051024/HD/NICHD NIH HHS/United States

PT - Journal Article

PT - Research Support, N.I.H., Extramural

PT - Research Support, Non-U.S. Gov't

DEP - 20130627

TA - J Pediatr

JT - The Journal of pediatrics

JID - 0375410

RN - 47, XYY syndrome

SB - IM

MH - Adolescent

MH - Adult

MH - Child

MH - Child Behavior

MH - Child, Preschool

MH - Cognition Disorders/diagnosis

MH - Cohort Studies

MH - Cross-Sectional Studies

MH - Hand Deformities/diagnosis

MH - Humans

MH - Infant

MH - Male

MH - Megalencephaly/diagnosis

MH - Muscle Hypotonia/diagnosis

MH - Neuropsychological Tests

MH - Phenotype

MH - Sex Chromosome Disorders/*diagnosis/*genetics

MH - Social Class

MH - Surveys and Questionnaires

MH - XYY Karyotype/*diagnosis/*genetics

MH - Young Adult

PMC - PMC4097881

MID - NIHMS605710

OTO - NOTNLM

OT - 47,XYY

OT - ADHD

OT - ADI-R

OT - AMH

OT - ASD

OT - Anti-mullerian hormone

OT - Attention deficit hyperactivity disorder

OT - Autism Diagnostic Interview–Revised

OT - Autistism spectrum disorder

OT - BMI

OT - Body mass index

OT - CBCL

OT - CDI-2

OT - Child Behavior Checklist

OT - Children's Depression Inventory–Second Edition

OT - FSH

OT - FSIQ

OT - Follicle stimulating hormone

OT - Full scale IQ

OT - HC

OT - Head circumference

OT - LH

OT - Luteinzing hormone

OT - SCQ

OT - SES

OT - SRS

OT - Social Communicative Questionnaire

OT - Social Responsiveness Scale

OT - Socioeconomic status

OT - WC

OT - Waist circumference

OT - XYY

EDAT- 2013/07/03 06:00

MHDA- 2013/12/16 06:00

CRDT- 2013/07/02 06:00

PHST- 2012/12/19 00:00 [received]

PHST- 2013/04/09 00:00 [revised]

PHST- 2013/05/15 00:00 [accepted]

PHST- 2013/07/02 06:00 [entrez]

PHST- 2013/07/03 06:00 [pubmed]

PHST- 2013/12/16 06:00 [medline]

AID - S0022-3476(13)00586-6 [pii]

AID - 10.1016/j.jpeds.2013.05.037 [doi]

PST - ppublish

SO - J Pediatr. 2013 Oct;163(4):1085-94. doi: 10.1016/j.jpeds.2013.05.037. Epub 2013

Jun 27.

PMID- 21342258

OWN - NLM

STAT- MEDLINE

DCOM- 20110809

LR - 20211020

IS - 1651-2227 (Electronic)

IS - 0803-5253 (Print)

IS - 0803-5253 (Linking)

VI - 100

IP - 6

DP - 2011 Jun

TI - 48,XXYY, 48,XXXY and 49,XXXXY syndromes: not just variants of Klinefelter

syndrome.

PG - 851-60

LID - 10.1111/j.1651-2227.2011.02235.x [doi]

AB - Sex chromosome tetrasomy and pentasomy conditions occur in 1:18,000-1:100,000

male births. While often compared with 47,XXY/Klinefelter syndrome because of

shared features including tall stature and hypergonadotropic hypogonadism,

48,XXYY, 48,XXXY and 49,XXXXY syndromes are associated with additional physical

findings, congenital malformations, medical problems and psychological features.

While the spectrum of cognitive abilities extends much higher than originally

described, developmental delays, cognitive impairments and behavioural disorders

are common and require strong treatment plans. Future research should focus on

genotype-phenotype relationships and the development of evidence-based

treatments. CONCLUSION: The more complex physical, medical and psychological

phenotypes of 48,XXYY, 48,XXXY and 49,XXXXY syndromes make distinction from

47,XXY important; however, all of these conditions share features of

hypergonadotropic hypogonadism and the need for increased awareness, biomedical

research and the development of evidence-based treatments.

CI - © 2011 The Author(s)/Acta Paediatrica © 2011 Foundation Acta Paediatrica.

FAU - Tartaglia, Nicole

AU - Tartaglia N

AD - Section of Neurodevelopmental Behavioral Pediatrics, Department of Pediatrics,

University of Colorado School of Medicine, Aurora, USA.

Tartaglia.nicole@tchden.org

FAU - Ayari, Natalie

AU - Ayari N

FAU - Howell, Susan

AU - Howell S

FAU - D'Epagnier, Cheryl

AU - D'Epagnier C

FAU - Zeitler, Philip

AU - Zeitler P

LA - eng

GR - K23 NS070337/NS/NINDS NIH HHS/United States

GR - UL1 RR025780/RR/NCRR NIH HHS/United States

GR - UL1 RR025780-02/RR/NCRR NIH HHS/United States

PT - Comparative Study

PT - Journal Article

PT - Research Support, Non-U.S. Gov't

PT - Review

DEP - 20110408

TA - Acta Paediatr

JT - Acta paediatrica (Oslo, Norway : 1992)

JID - 9205968

SB - IM

MH - Humans

MH - Hypogonadism/genetics

MH - Klinefelter Syndrome/diagnosis/*genetics/psychology

MH - Male

MH - Phenotype

MH - Sex Chromosome Disorders/diagnosis/*genetics/psychology

MH - Syndrome

PMC - PMC3314712

MID - NIHMS363082

EDAT- 2011/02/24 06:00

MHDA- 2011/08/10 06:00

CRDT- 2011/02/24 06:00

PHST- 2011/02/24 06:00 [entrez]

PHST- 2011/02/24 06:00 [pubmed]

PHST- 2011/08/10 06:00 [medline]

AID - 10.1111/j.1651-2227.2011.02235.x [doi]

PST - ppublish

SO - Acta Paediatr. 2011 Jun;100(6):851-60. doi: 10.1111/j.1651-2227.2011.02235.x.

Epub 2011 Apr 8.

PMID- 32748612

OWN - NLM

STAT- MEDLINE

DCOM- 20211021

LR - 20211021

IS - 1827-1715 (Electronic)

IS - 0026-4946 (Linking)

VI - 72

IP - 6

DP - 2020 Dec

TI - Tall stature in children and adolescents.

PG - 472-483

LID - 10.23736/S0026-4946.20.05971-X [doi]

AB - Tall stature is usually defined as a height beyond 97(th) percentile or more than

2 SD above the mean height for age and sex in a defined population. Familiar tall

stature, also known as constitutional tall stature, is the most common cause of

tall stature. Overnutrition, obesity, also usually causes overgrowth. Tall

stature by itself is not a pathological condition, however, there are a number of

disorders associated with tall stature. Some genetic disorders and syndromes may

be associated with mental retardation and various complications. Therefore,

recognition of tall stature and revealing the underlying pathogenic causes and

making the diagnosis are important not to miss the serious conditions and to

provide adequate medical care and genetic counseling. Pathological causes for

tall statute include endocrine disorders, such as excessive growth hormone

secretion, hyperthyroidism, precocious puberty and lipodystrophy, chromosome

disorders, such as Trisomy X (47, XXX female), Klinefelter Syndrome (47, XXY),

XYY syndrome (47, XYY male) and fragile X syndrome, and syndromes and metabolic

disorders, such as Marfan Syndrome, Beckwith-Wiedemann Syndrome,

Simpson-Golabi-Behmel Syndrome, Sotos Syndrome and homocystinuria. Children may

require growth-reductive treatment if the predicted adult height would be

excessive and unacceptable. Some hormonal, high doses of sex steroids, or

surgical, bilateral percutaneous epiphysiodesis of the distal femur and proximal

tibia and fibula, treatment is currently available to reduce adult height.

FAU - Urakami, Tatsuhiko

AU - Urakami T

AD - Department of Pediatrics, Nihon University School of Medicine, Tokyo, Japan -

urakami.tatsuhiko@nihon-u.ac.jp.

LA - eng

PT - Journal Article

PT - Review

DEP - 20200804

PL - Italy

TA - Minerva Pediatr

JT - Minerva pediatrica

JID - 0400740

RN - Triple X syndrome

SB - IM

MH - Adolescent

MH - Algorithms

MH - *Body Height/genetics

MH - Child

MH - Chromosomes, Human, X

MH - Endocrine System Diseases/complications

MH - Fragile X Syndrome/complications

MH - *Growth Disorders/diagnosis/etiology/genetics/therapy

MH - Humans

MH - Hyperthyroidism/complications

MH - Intellectual Disability/etiology

MH - Klinefelter Syndrome/complications

MH - Overnutrition/complications

MH - Puberty, Precocious/complications

MH - Reference Values

MH - Sex Chromosome Aberrations

MH - Sex Chromosome Disorders of Sex Development/complications

MH - Trisomy

EDAT- 2020/08/05 06:00

MHDA- 2020/08/05 06:00

CRDT- 2020/08/05 06:00

PHST- 2020/08/05 06:00 [pubmed]

PHST- 2020/08/05 06:00 [medline]

PHST- 2020/08/05 06:00 [entrez]

AID - S0026-4946.20.05971-X [pii]

AID - 10.23736/S0026-4946.20.05971-X [doi]

PST - ppublish

SO - Minerva Pediatr. 2020 Dec;72(6):472-483. doi: 10.23736/S0026-4946.20.05971-X.

Epub 2020 Aug 4.

PMID- 29972105

OWN - NLM

STAT- MEDLINE

DCOM- 20190618

LR - 20220410

IS - 2212-3873 (Electronic)

IS - 1871-5303 (Print)

IS - 1871-5303 (Linking)

VI - 19

IP - 2

DP - 2019

TI - Neuropsychiatric Aspects in Men with Klinefelter Syndrome.

PG - 109-115

LID - 10.2174/1871530318666180703160250 [doi]

AB - BACKGROUND AND OBJECTIVE: Klinefelter Syndrome (KS) is the most common sex

chromosome aneuploidy (47, XXY) and cause of male hypergonadotropic hypogonadism.

It is characterized by an extreme clinical heterogeneity in presentation,

including infertility, hypogonadism, language delay, metabolic comorbidities, and

neurocognitive and psychiatric disorders. Since testosterone is known to have

organizational, neurotrophic and neuroprotective effects on brain, the condition

of primary hypogonadism could play a role. Moreover, given that KS subjects have

an additional X, genes on the extra-chromosome could also exert a significant

impact. The aim of this narrative review is to analyze the available literature

on the relationship between KS and neuropsychiatric disorders. METHODS: To extend

to the best of published literature on the topic, appropriate keywords and MeSH

terms were identified and searched in Pubmed. Finally, references of original

articles and reviews were examined. RESULTS: Both morphological and functional

studies focusing on the brain showed that there were important differences in

brain structure of KS subjects. Different psychiatric disorders such as

Schizophrenia, autism, attention deficit hyperactivity disorder, depression and

anxiety were frequently reported in KS patients according to a broad spectrum of

phenotypes. T supplementation (TRT) was not able to improve the psychotic

disorders in KS men with or without overt hypogonadism. CONCLUSION: Although the

risk of psychosis, depression and autism is increased in subjects with KS, no

definitive evidence has been found in studies aiming at identifying the

relationship between aneuploidy, T deficit and the risk of psychiatric and

cognitive disorders in subjects affected by KS.

CI - Copyright© Bentham Science Publishers; For any queries, please email at

epub@benthamscience.net.

FAU - Giagulli, Vito Angelo

AU - Giagulli VA

AD - Section of Internal Medicine, Geriatrics, Endocrinology and Rare Diseases,

Interdisciplinary Department of Medicine, University of Bari, School of Medicine,

Policlinico, Bari, Italy.

FAU - Campone, Beatrice

AU - Campone B

AD - Psychiatric Unit Department of Health Science, University of Florence, Italy.

FAU - Castellana, Marco

AU - Castellana M

AD - via Turi 44 Rutigliano, Bari, Italy.

FAU - Salzano, Ciro

AU - Salzano C

AD - Dipartimento di Medicina Clinica e Chirurgia, Sezione di Endocrinologia,

Universita "Federico II" di Napoli, Naples, Italy.

FAU - Fisher, Alessandra Daphne

AU - Fisher AD

AD - Sexual Medicine and Andrology Unit, Department of Experimental Clinical and

Biomedical Sciences "Mario Serio", University of Florence, Florence, Italy.

FAU - de Angelis, Cristina

AU - de Angelis C

AD - Dipartimento di Medicina Clinica e Chirurgia, Sezione di Endocrinologia,

Universita "Federico II" di Napoli, Naples, Italy.

FAU - Pivonello, Rosario

AU - Pivonello R

AD - Dipartimento di Medicina Clinica e Chirurgia, Sezione di Endocrinologia,

Universita "Federico II" di Napoli, Naples, Italy.

FAU - Colao, Annamaria

AU - Colao A

AD - Dipartimento di Medicina Clinica e Chirurgia, Sezione di Endocrinologia,

Universita "Federico II" di Napoli, Naples, Italy.

FAU - Pasquali, Daniela

AU - Pasquali D

AD - Department of Medical, Surgical, Neurological, Metabolic Sciences and Aging,

University of Campania "Luigi Vanvitelli", 80138 Naples, Italy.

FAU - Maggi, Mario

AU - Maggi M

AD - Sexual Medicine and Andrology Unit, Department of Experimental Clinical and

Biomedical Sciences "Mario Serio", University of Florence, Florence, Italy.

FAU - Triggiani, Vincenzo

AU - Triggiani V

AD - Section of Internal Medicine, Geriatrics, Endocrinology and Rare Diseases,

Interdisciplinary Department of Medicine, University of Bari, School of Medicine,

Policlinico, Bari, Italy.

FAU - On Behalf Of The Klinefelter ItaliaN Group King

AU - On Behalf Of The Klinefelter ItaliaN Group King

LA - eng

PT - Journal Article

PT - Review

TA - Endocr Metab Immune Disord Drug Targets

JT - Endocrine, metabolic & immune disorders drug targets

JID - 101269157

RN - 3XMK78S47O (Testosterone)

SB - IM

MH - Brain/*physiopathology

MH - Comorbidity

MH - Humans

MH - Hypogonadism/complications/epidemiology/physiopathology/psychology

MH - Klinefelter Syndrome/*complications/epidemiology/*physiopathology/*psychology

MH - Male

MH - Mental Disorders/epidemiology/*etiology/physiopathology

MH - Phenotype

MH - Testosterone/blood

PMC - PMC7360906

OTO - NOTNLM

OT - Hypergonadotropic hypogonadism

OT - Klinefelter Syndrome

OT - attention deficit

OT - autism

OT - depression; anxiety

OT - hyperactivity disorder

OT - schizophrenia

OT - testosterone.

EDAT- 2018/07/05 06:00

MHDA- 2019/06/19 06:00

CRDT- 2018/07/05 06:00

PHST- 2018/03/07 00:00 [received]

PHST- 2018/05/06 00:00 [revised]

PHST- 2018/05/07 00:00 [accepted]

PHST- 2018/07/05 06:00 [pubmed]

PHST- 2019/06/19 06:00 [medline]

PHST- 2018/07/05 06:00 [entrez]

AID - EMIDDT-EPUB-91471 [pii]

AID - EMIDDT-19-109 [pii]

AID - 10.2174/1871530318666180703160250 [doi]

PST - ppublish

SO - Endocr Metab Immune Disord Drug Targets. 2019;19(2):109-115. doi:

10.2174/1871530318666180703160250.

PMID- 34009243

OWN - NLM

STAT- MEDLINE

DCOM- 20220309

LR - 20220506

IS - 1460-2199 (Electronic)

IS - 1047-3211 (Print)

IS - 1047-3211 (Linking)

VI - 31

IP - 9

DP - 2021 Jul 29

TI - Resting-State Functional Connectivity and Psychopathology in Klinefelter Syndrome

(47, XXY).

PG - 4180-4190

LID - 10.1093/cercor/bhab077 [doi]

AB - Klinefelter syndrome (47, XXY; henceforth: XXY syndrome) is a high-impact but

poorly understood genetic risk factor for neuropsychiatric impairment. Here, we

provide the first study to map alterations of functional brain connectivity in

XXY syndrome and relate these changes to brain anatomy and psychopathology. We

used resting-state functional magnetic resonance imaging data from 75 individuals

with XXY and 84 healthy XY males to 1) implement a brain-wide screen for altered

global resting-state functional connectivity (rsFC) in XXY versus XY males and 2)

decompose these alterations through seed-based analysis. We then compared these

rsFC findings with measures of regional brain anatomy, psychopathology, and

cognition. XXY syndrome was characterized by increased global rsFC in the left

dorsolateral prefrontal cortex (DLPFC)-reflecting DLPFC overconnectivity with

diverse rsFC networks. Functional overconnectivity was partly coupled to

co-occurring regional volumetric changes in XXY syndrome, and variation in

DLPFC-precuneus rsFC was correlated with the severity of psychopathology. By

providing the first view of altered rsFC in XXY syndrome and contextualizing

observed changes relative to neuroanatomy and behavior, our study helps to

advance biological understanding of XXY syndrome-both as a disorder in its own

right and more broadly as a model of genetic risk for psychopathology.

CI - © Published by Oxford University Press 2021.

FAU - Whitman, Ethan T

AU - Whitman ET

AD - Section on Developmental Neurogenomics, Human Genetics Branch, National Institute

of Mental Health, Bethesda, MD 20814, USA.

FAU - Liu, Siyuan

AU - Liu S

AD - Section on Developmental Neurogenomics, Human Genetics Branch, National Institute

of Mental Health, Bethesda, MD 20814, USA.

FAU - Torres, Erin

AU - Torres E

AD - Section on Developmental Neurogenomics, Human Genetics Branch, National Institute

of Mental Health, Bethesda, MD 20814, USA.

FAU - Warling, Allysa

AU - Warling A

AD - Section on Developmental Neurogenomics, Human Genetics Branch, National Institute

of Mental Health, Bethesda, MD 20814, USA.

FAU - Wilson, Kathleen

AU - Wilson K

AD - Section on Developmental Neurogenomics, Human Genetics Branch, National Institute

of Mental Health, Bethesda, MD 20814, USA.

FAU - Nadig, Ajay

AU - Nadig A

AD - Section on Developmental Neurogenomics, Human Genetics Branch, National Institute

of Mental Health, Bethesda, MD 20814, USA.

FAU - McDermott, Cassidy

AU - McDermott C

AD - Section on Developmental Neurogenomics, Human Genetics Branch, National Institute

of Mental Health, Bethesda, MD 20814, USA.

FAU - Clasen, Liv S

AU - Clasen LS

AD - Section on Developmental Neurogenomics, Human Genetics Branch, National Institute

of Mental Health, Bethesda, MD 20814, USA.

FAU - Blumenthal, Jonathan D

AU - Blumenthal JD

AD - Section on Developmental Neurogenomics, Human Genetics Branch, National Institute

of Mental Health, Bethesda, MD 20814, USA.

FAU - Lalonde, François M

AU - Lalonde FM

AD - Section on Developmental Neurogenomics, Human Genetics Branch, National Institute

of Mental Health, Bethesda, MD 20814, USA.

FAU - Gotts, Stephen J

AU - Gotts SJ

AD - Section on Cognitive Neuropsychology, Laboratory of Brain and Cognition, National

Institute of Mental Health, Bethesda, MD 20814, USA.

FAU - Martin, Alex

AU - Martin A

AD - Section on Cognitive Neuropsychology, Laboratory of Brain and Cognition, National

Institute of Mental Health, Bethesda, MD 20814, USA.

FAU - Raznahan, Armin

AU - Raznahan A

AD - Section on Developmental Neurogenomics, Human Genetics Branch, National Institute

of Mental Health, Bethesda, MD 20814, USA.

LA - eng

SI - ClinicalTrials.gov/NCT00001246

GR - NCT00001246/National Institutes of Mental Health Intramural Research Program/

GR - ZIAMH002949-03/NIH Annual Report/

PT - Journal Article

PT - Research Support, N.I.H., Intramural

TA - Cereb Cortex

JT - Cerebral cortex (New York, N.Y. : 1991)

JID - 9110718

SB - IM

MH - Adolescent

MH - Child

MH - Chromosomes, Human, X/*genetics

MH - Chromosomes, Human, Y/genetics

MH - Female

MH - Humans

MH - Intelligence Tests

MH - Klinefelter Syndrome/diagnostic imaging/*genetics/*psychology

MH - Magnetic Resonance Imaging

MH - Male

MH - Mental Disorders/genetics/psychology

MH - Neural Pathways/diagnostic imaging/*physiology

MH - Neuroimaging

MH - Parietal Lobe/diagnostic imaging/physiopathology

MH - Prefrontal Cortex/diagnostic imaging/physiopathology

MH - Young Adult

PMC - PMC8485146

OTO - NOTNLM

OT - *X-chromosome

OT - *XXY syndrome

OT - *aneuploidy

OT - *fMRI

OT - *sex chromosome

EDAT- 2021/05/20 06:00

MHDA- 2022/03/11 06:00

CRDT- 2021/05/19 12:33

PHST- 2021/05/20 06:00 [pubmed]

PHST- 2022/03/11 06:00 [medline]

PHST- 2021/05/19 12:33 [entrez]

AID - 6261382 [pii]

AID - bhab077 [pii]

AID - 10.1093/cercor/bhab077 [doi]

PST - ppublish

SO - Cereb Cortex. 2021 Jul 29;31(9):4180-4190. doi: 10.1093/cercor/bhab077.

PMID- 34874073

OWN - NLM

STAT- Publisher

LR - 20220716

IS - 1097-0223 (Electronic)

IS - 0197-3851 (Print)

IS - 0197-3851 (Linking)

DP - 2021 Dec 7

TI - Prenatal phenotype of 47, XXY (Klinefelter syndrome).

LID - 10.1002/pd.6071 [doi]

AB - OBJECTIVE: There is a paucity of knowledge regarding the prenatal presentation of

Klinefelter syndrome, or 47, XXY. Accurate prenatal counseling is critical and in

utero diagnosis is currently limited by a poor understanding of the prenatal

phenotype of this condition. METHODS: This is a case series of fetuses with

cytogenetically confirmed 47, XXY in the prenatal period or up to age 5 years,

with prenatal records available for review from four academic institutions

between 2006 and 2019. Ultrasound reports were reviewed in detail to assess for

increased nuchal translucency and structural abnormalities. Additionally, we

reviewed results of cell-free DNA and serum analyte testing when performed to

inform our understanding of the detection of fetal 47, XXY through standard

genetic screening tests. RESULTS: Forty-one cases with confirmed cytogenetic

diagnosis of 47, XXY and prenatal records available for review were identified:

37 had a prenatal diagnosis and 4 had a postnatal diagnosis. Nuchal translucency

was increased ≥3.0 mm in 23.1% (6/26) of cases with a documented measurement. In

29.2% (7/24) of cases with a second trimester anatomical ultrasound available for

review, a fetal abnormality was identified (3 brain anomalies, 1 cardiac

abnormality, 1 echogenic bowel, and 2 limb abnormalities). Among those who had

cell-free DNA and serum analytes performed, 92.6% (25/27) and 36.3% (4/11) had an

abnormal result respectively. CONCLUSION: This case series expands our knowledge

of the prenatal presentation of 47, XXY by identifying first and second trimester

fetal sonographic abnormalities. Prenatal identification of this condition

enables accurate counseling, focused prenatal management, and early postnatal

interventions to ameliorate some of the known complications.

CI - © 2021 John Wiley & Sons Ltd.

FAU - Swanson, Kate

AU - Swanson K

AUID- ORCID: 0000-0003-1174-1501

AD - Division of Maternal-Fetal Medicine, Department of Obstetrics, Gynecology, and

Reproductive Sciences, University of California, San Francisco, California, USA.

AD - Division of Medical Genetics, Department of Pediatrics, University of California,

San Francisco, California, USA.

FAU - Bishop, Juliet C

AU - Bishop JC

AD - Division of Maternal-Fetal Medicine, Department of Gynecology and Obstetrics, The

Johns Hopkins University School of Medicine, Baltimore, Maryland, USA.

AD - Department of Genetic Medicine, The Johns Hopkins University School of Medicine,

Baltimore, Maryland, USA.

FAU - Al-Kouatly, Huda B

AU - Al-Kouatly HB

AUID- ORCID: 0000-0003-2922-0333

AD - Division of Maternal-Fetal Medicine, Sidney Kimmel Medical College of Thomas

Jefferson University, Philadelphia, Pennsylvania, USA.

FAU - Makhamreh, Mona

AU - Makhamreh M

AD - Division of Maternal-Fetal Medicine, Sidney Kimmel Medical College of Thomas

Jefferson University, Philadelphia, Pennsylvania, USA.

FAU - Felton, Thomas

AU - Felton T

AD - McLendon Clinical Laboratories, Cytogenetics Laboratory, University of North

Carolina Health, Chapel Hill, North Carolina, USA.

FAU - Vora, Neeta L

AU - Vora NL

AUID- ORCID: 0000-0002-2504-9455

AD - Division of Maternal-Fetal Medicine, Department of Obstetrics and Gynecology,

University of North Carolina at Chapel Hill School of Medicine, Chapel Hill,

North Carolina, USA.

FAU - Sparks, Teresa N

AU - Sparks TN

AUID- ORCID: 0000-0002-8593-2186

AD - Division of Maternal-Fetal Medicine, Department of Obstetrics, Gynecology, and

Reproductive Sciences, University of California, San Francisco, California, USA.

FAU - Jelin, Angie C

AU - Jelin AC

AUID- ORCID: 0000-0002-1792-4029

AD - Division of Maternal-Fetal Medicine, Department of Gynecology and Obstetrics, The

Johns Hopkins University School of Medicine, Baltimore, Maryland, USA.

AD - Department of Genetic Medicine, The Johns Hopkins University School of Medicine,

Baltimore, Maryland, USA.

LA - eng

GR - K12 HD001262/HD/NICHD NIH HHS/United States

GR - K23 DK119949/DK/NIDDK NIH HHS/United States

GR - K23 HD088742/HD/NICHD NIH HHS/United States

GR - Fetal Health Foundation/

GR - 5K12HD001262-18/NH/NIH HHS/United States

GR - Brianna Marie Foundation/

GR - K23HDD088742/NH/NIH HHS/United States

GR - 5K23DK119949-02/NH/NIH HHS/United States

PT - Journal Article

DEP - 20211207

TA - Prenat Diagn

JT - Prenatal diagnosis

JID - 8106540

SB - IM

PMC - PMC9170827

MID - NIHMS1765897

COIS- CONFLICTS OF INTEREST The authors report no conflicts of interest.

EDAT- 2021/12/08 06:00

MHDA- 2021/12/08 06:00

PMCR- 2023/06/07

CRDT- 2021/12/07 09:27

PHST- 2021/10/16 00:00 [revised]

PHST- 2021/07/26 00:00 [received]

PHST- 2021/11/24 00:00 [accepted]

PHST- 2023/06/07 00:00 [pmc-release]

PHST- 2021/12/07 09:27 [entrez]

PHST- 2021/12/08 06:00 [pubmed]

PHST- 2021/12/08 06:00 [medline]

AID - 10.1002/pd.6071 [doi]

PST - aheadofprint

SO - Prenat Diagn. 2021 Dec 7:10.1002/pd.6071. doi: 10.1002/pd.6071.

PMID- 34238477

OWN - NLM

STAT- MEDLINE

DCOM- 20210728

LR - 20210728

IS - 0072-9752 (Print)

IS - 0072-9752 (Linking)

VI - 181

DP - 2021

TI - Klinefelter syndrome or testicular dysgenesis: Genetics, endocrinology, and

neuropsychology.

PG - 445-462

LID - B978-0-12-820683-6.00032-4 [pii]

LID - 10.1016/B978-0-12-820683-6.00032-4 [doi]

AB - Klinefelter syndrome (47,XXY) is a frequent chromosomal disorder among males,

often presenting with hypergonadotropic hypogonadism, small firm testicles,

metabolic disorders, neurocognitive challenges, and increased height. Neurologic

disorders such as epilepsy, seizures, and tremor as well as psychiatric disorders

are also seen more frequently. The neurocognitive deficits seen are present in

many areas of cognition, typically affecting general cognitive abilities,

language, and executive functioning. Also, social dysfunction is frequent.

Dyslexia is present in more than half of all males. Brain imaging studies

generally show a typical pattern, with many nuclei and brain areas being smaller

than among controls. However, it has not been possible to link the brain

alterations found in imaging studies with the neurocognitive profile. The

genetics underlying the phenotypic traits found among males with Klinefelter

syndrome still remains to be elucidated; however, recent studies have described

pervasive changes in the methylome and transcriptome and new and interesting

candidate genes have been pinpointed, but their involvement in the phenotype of

Klinefelter syndrome has not been proven.

CI - Copyright © 2021 Elsevier B.V. All rights reserved.

FAU - Skakkebæk, Anne

AU - Skakkebæk A

AD - Department of Molecular Medicine, Aarhus University Hospital, Aarhus, Denmark;

Department of Clinical Genetics, Aarhus University Hospital, Aarhus, Denmark.

FAU - Wallentin, Mikkel

AU - Wallentin M

AD - Center of Functionally Integrative Neuroscience, Aarhus University Hospital,

Aarhus, Denmark; Center for Semiotics, Aarhus University, Aarhus, Denmark.

FAU - Gravholt, Claus Højbjerg

AU - Gravholt CH

AD - Department of Molecular Medicine, Aarhus University Hospital, Aarhus, Denmark;

Department of Endocrinology and Internal Medicine and Medical Research

Laboratories, Aarhus University Hospital, Aarhus, Denmark. Electronic address:

d280623@dadlnet.dk.

LA - eng

PT - Journal Article

PT - Review

PL - Netherlands

TA - Handb Clin Neurol

JT - Handbook of clinical neurology

JID - 0166161

SB - IM

MH - Brain

MH - Humans

MH - *Klinefelter Syndrome/genetics

MH - Male

MH - *Mental Disorders

MH - *Metabolic Diseases

MH - Neuropsychology

OTO - NOTNLM

OT - Brain imaging

OT - Epilepsy

OT - Genetics

OT - Genomics

OT - Hypogonadism

OT - Klinefelter syndrome

OT - Neurocognition

OT - Testosterone

EDAT- 2021/07/10 06:00

MHDA- 2021/07/29 06:00

CRDT- 2021/07/09 05:51

PHST- 2021/07/09 05:51 [entrez]

PHST- 2021/07/10 06:00 [pubmed]

PHST- 2021/07/29 06:00 [medline]

AID - B978-0-12-820683-6.00032-4 [pii]

AID - 10.1016/B978-0-12-820683-6.00032-4 [doi]

PST - ppublish

SO - Handb Clin Neurol. 2021;181:445-462. doi: 10.1016/B978-0-12-820683-6.00032-4.

PMID- 32733741

OWN - NLM

STAT- PubMed-not-MEDLINE

LR - 20211203

IS - 2146-4596 (Print)

IS - 2146-460X (Electronic)

IS - 2146-460X (Linking)

VI - 9

IP - 4

DP - 2020 Dec

TI - Klinefelter Syndrome Mosaicism 46,XX/47,XXY: A New Case and Literature Review.

PG - 221-226

LID - 10.1055/s-0040-1713002 [doi]

AB - Most cases of Klinefelter syndrome (KS) have 47,XXY karyotype. We reported the

first case of 46,XX/47,XXY KS whose genital ambiguity was detected prenatally

with postnatal confirmation of the mosaicism and ovotesticular disorder of sex

development (OT-DSD). The paternal origin of the extra X chromosome was

identified using trio cytogenomic single-nucleotide polymorphism array.

Additional 18 cases were also reviewed. The clinical presentation of 46,XX/47,XXY

is age-dependent with two age peaks, including ambiguous genitalia during infancy

and gynecomastia with or without cyclical hematuria and left scrotal pain and

mass in adolescence. The 46,XX is the predominant karyotype both in peripheral

blood and gonadal tissue. The risk of germ cell tumor is very high throughout

life in these individuals. Individuals with 46,XX/47,XXY mosaicism should be

treated more as OT-DSD other than a simple mosaic KS. A multidisciplinary

approach and long-term monitoring are necessary.

CI - © Thieme Medical Publishers.

FAU - Tangshewinsirikul, Chayada

AU - Tangshewinsirikul C

AD - Division of Maternal Fetal Medicine, Department of Obstetrics and Gynecology,

Faculty of Medicine Ramathibodi Hospital, Mahidol University, Bangkok, Thailand.

FAU - Dulyaphat, Wirada

AU - Dulyaphat W

AD - Division of Maternal Fetal Medicine, Department of Obstetrics and Gynecology,

Faculty of Medicine Ramathibodi Hospital, Mahidol University, Bangkok, Thailand.

FAU - Tim-Aroon, Thipwimol

AU - Tim-Aroon T

AD - Division of Medical Genetics, Department of Pediatrics, Faculty of Medicine

Ramathibodi Hospital, Mahidol University, Bangkok, Thailand.

FAU - Parinayok, Rachanee

AU - Parinayok R

AD - Department of Pathology, Faculty of Medicine Ramathibodi Hospital, Mahidol

University, Bangkok, Thailand.

FAU - Chareonsirisuthigul, Takol

AU - Chareonsirisuthigul T

AD - Department of Pathology, Faculty of Medicine Ramathibodi Hospital, Mahidol

University, Bangkok, Thailand.

FAU - Korkiatsakul, Veerawat

AU - Korkiatsakul V

AD - Department of Pathology, Faculty of Medicine Ramathibodi Hospital, Mahidol

University, Bangkok, Thailand.

FAU - Waisayarat, Jariya

AU - Waisayarat J

AD - Department of Pathology, Faculty of Medicine Ramathibodi Hospital, Mahidol

University, Bangkok, Thailand.

FAU - Sirisreetreerux, Pokket

AU - Sirisreetreerux P

AD - Division of Urology, Department of Surgery, Faculty of Medicine Ramathibodi

Hospital, Mahidol University, Bangkok, Thailand.

FAU - Tingthanatikul, Yada

AU - Tingthanatikul Y

AD - Division of Reproductive Medicine, Department of Obstetrics and Gynecology,

Faculty of Medicine Ramathibodi Hospital, Mahidol University, Bangkok, Thailands.

FAU - Wattanasirichaigoon, Duangrurdee

AU - Wattanasirichaigoon D

AD - Division of Medical Genetics, Department of Pediatrics, Faculty of Medicine

Ramathibodi Hospital, Mahidol University, Bangkok, Thailand.

LA - eng

PT - Journal Article

DEP - 20200617

TA - J Pediatr Genet

JT - Journal of pediatric genetics

JID - 101589859

PMC - PMC7384885

OTO - NOTNLM

OT - ambiguous genitalia

OT - cyclic hematuria

OT - disorder of sex development

OT - germ cell tumor

OT - left scrotal pain

COIS- Conflict of Interest None declared.

EDAT- 2020/08/01 06:00

MHDA- 2020/08/01 06:01

CRDT- 2020/08/01 06:00

PHST- 2020/03/02 00:00 [received]

PHST- 2020/04/21 00:00 [accepted]

PHST- 2020/08/01 06:00 [entrez]

PHST- 2020/08/01 06:00 [pubmed]

PHST- 2020/08/01 06:01 [medline]

AID - 2000028 [pii]

AID - 10.1055/s-0040-1713002 [doi]

PST - ppublish

SO - J Pediatr Genet. 2020 Dec;9(4):221-226. doi: 10.1055/s-0040-1713002. Epub 2020

Jun 17.

PMID- 33978253

OWN - NLM

STAT- MEDLINE

DCOM- 20211228

LR - 20211228

IS - 1365-2249 (Electronic)

IS - 0009-9104 (Print)

IS - 0009-9104 (Linking)

VI - 205

IP - 3

DP - 2021 Sep

TI - Non-organ-specific autoimmunity in adult 47,XXY Klinefelter patients and

higher-grade X-chromosome aneuploidies.

PG - 316-325

LID - 10.1111/cei.13616 [doi]

AB - Current literature regarding systemic autoimmune diseases in X-chromosome

aneuploidies is scarce and limited to case reports. Our aim was to evaluate the

frequency of anti-nuclear (ANAs), extractable nuclear (ENA), anti-double-stranded

DNA (dsDNAs), anti-smooth muscle (ASMAs) and anti-mitochondrial (AMAs) antibodies

in a large cohort of adults with Klinefelter's syndrome (KS, 47,XXY) and rare

higher-grade sex chromosome aneuploidies (HGAs) for the first time. Sera from 138

X-chromosome aneuploid patients [124 adult patients with 47,XXY KS and 14

patients with HGA (six children, eight adults)] and 50 age-matched 46,XY controls

were recruited from the Sapienza University of Rome (2007-17) and tested for

ANAs, ENAs, anti-dsDNAs, ASMAs and AMAs. Non-organ-specific immunoreactivity was

found to be significantly higher in patients with 47,XXY KS (14%) than in the

controls (2%, p = 0.002). Among all the antibodies investigated, only ANAs were

observed significantly more frequently in patients with 47,XXY KS (12.1%) than in

the controls (2%, p = 0.004). No anti-dsDNA immunoreactivity was found.

Stratifying by testosterone replacement therapy (TRT), non-organ-specific

autoantibody frequencies were higher in TRT-naive (p = 0.01) and TRT-treated

groups than in controls. No patients with HGA were found positive for the various

autoantibodies. Non-organ-specific autoantibodies were significantly present in

47,XXY adult patients. Conversely, HGAs did not appear to be target of

non-organ-specific immunoreactivity, suggesting that KS and HGAs should be

considered as two distinct conditions. The classification and diagnosis of

systemic autoimmune diseases is frequently difficult. To support a correct

clinical evaluation of KS disease and to prevent eventual secondary irreversible

immune-mediated damages, we highlight the importance of screening for

non-organ-specific autoimmunity in Klinefelter's syndrome.

CI - © 2021 British Society for Immunology.

FAU - Panimolle, Francesca

AU - Panimolle F

AD - Center of Rare Diseases, Section of Medical Pathophysiology, Department of

Experimental Medicine, Sapienza University of Rome, Roma, Italy.

FAU - Tiberti, Claudio

AU - Tiberti C

AD - Center of Rare Diseases, Section of Medical Pathophysiology, Department of

Experimental Medicine, Sapienza University of Rome, Roma, Italy.

FAU - Spaziani, Matteo

AU - Spaziani M

AD - Center of Rare Diseases, Section of Medical Pathophysiology, Department of

Experimental Medicine, Sapienza University of Rome, Roma, Italy.

FAU - Riitano, Gloria

AU - Riitano G

AD - Department of Experimental Medicine, Sapienza University of Rome, Roma, Italy.

FAU - Lucania, Giuseppe

AU - Lucania G

AD - Department of Experimental Medicine, Sapienza University of Rome, Roma, Italy.

FAU - Anzuini, Antonella

AU - Anzuini A

AD - Center of Rare Diseases, Section of Medical Pathophysiology, Department of

Experimental Medicine, Sapienza University of Rome, Roma, Italy.

FAU - Lenzi, Andrea

AU - Lenzi A

AD - Center of Rare Diseases, Section of Medical Pathophysiology, Department of

Experimental Medicine, Sapienza University of Rome, Roma, Italy.

FAU - Gianfrilli, Daniele

AU - Gianfrilli D

AD - Center of Rare Diseases, Section of Medical Pathophysiology, Department of

Experimental Medicine, Sapienza University of Rome, Roma, Italy.

FAU - Sorice, Maurizio

AU - Sorice M

AUID- ORCID: 0000-0003-3534-1502

AD - Department of Experimental Medicine, Sapienza University of Rome, Roma, Italy.

FAU - Radicioni, Antonio F

AU - Radicioni AF

AD - Center of Rare Diseases, Section of Medical Pathophysiology, Department of

Experimental Medicine, Sapienza University of Rome, Roma, Italy.

LA - eng

GR - 2017N8CK4K/MIUR research project number 2017N8CK4K/

PT - Journal Article

PT - Research Support, Non-U.S. Gov't

DEP - 20210610

TA - Clin Exp Immunol

JT - Clinical and experimental immunology

JID - 0057202

RN - 0 (Antibodies, Antinuclear)

RN - 0 (Antigens, Nuclear)

RN - 0 (Autoantibodies)

RN - 0 (anti-dsDNA autoantibody)

SB - IM

MH - Adolescent

MH - Adult

MH - Aneuploidy

MH - Antibodies, Antinuclear/*blood/immunology

MH - Antigens, Nuclear/blood/immunology

MH - Autoantibodies/*blood/immunology

MH - Autoimmune Diseases/*genetics/immunology

MH - Autoimmunity/immunology

MH - Child

MH - Child, Preschool

MH - Humans

MH - Klinefelter Syndrome/*blood/genetics/immunology

MH - Male

MH - Middle Aged

MH - Mitochondria/*immunology

MH - Muscle, Smooth/*immunology

MH - Sex Chromosome Aberrations

MH - Young Adult

PMC - PMC8374223

OTO - NOTNLM

OT - *AMA

OT - *ANA

OT - *ASMA

OT - *ENA

OT - *Klinefelter’s syndrome

OT - *X-chromosome aneuploidies

OT - *anti-DNA

OT - *non-organ-specific autoimmunity

COIS- No potential conflicts of interest relevant to this article were reported.

EDAT- 2021/05/13 06:00

MHDA- 2021/12/29 06:00

PMCR- 2022/09/01

CRDT- 2021/05/12 09:00

PHST- 2021/03/24 00:00 [revised]

PHST- 2020/11/26 00:00 [received]

PHST- 2021/04/10 00:00 [accepted]

PHST- 2022/09/01 00:00 [pmc-release]

PHST- 2021/05/13 06:00 [pubmed]

PHST- 2021/12/29 06:00 [medline]

PHST- 2021/05/12 09:00 [entrez]

AID - CEI13616 [pii]

AID - 10.1111/cei.13616 [doi]

PST - ppublish

SO - Clin Exp Immunol. 2021 Sep;205(3):316-325. doi: 10.1111/cei.13616. Epub 2021 Jun

10.

PMID- 21418719

OWN - NLM

STAT- MEDLINE

DCOM- 20150406

LR - 20211020

IS - 1469-7661 (Electronic)

IS - 1355-6177 (Print)

IS - 1355-6177 (Linking)

VI - 17

IP - 3

DP - 2011 May

TI - Executive function in young males with Klinefelter (XXY) syndrome with and

without comorbid attention-deficit/hyperactivity disorder.

PG - 522-30

LID - 10.1017/S1355617711000312 [doi]

AB - Deficits in executive function (EF) are reported to occur in individuals with

Klinefelter syndrome (XXY). The degree of impairment, if any, is variable and the

nature of these deficits has not been clearly elucidated in young males. In this

report, we (a) examine EF skills using multiple tasks in a non-clinic referred

group of youth with XXY, (b) describe the extent of EF weaknesses in XXY when

this group is compared with typical males of a similar SES or typical males with

similar verbal abilities, and (c) evaluate the contribution of comorbid

attention-deficit/hyperactivity disorder (ADHD) to EF skills. The sample included

27 males with XXY (ages 9-25), 27 typically developing age- and

vocabulary-matched males, and 22 age- and socioeconomic status-matched males. EF

tasks included Verbal Fluency, the Trail Making Test, and the CANTAB Spatial

Working Memory and Stockings of Cambridge tasks. Mixed model analysis of variance

was used to compare the groups on EF tasks and revealed a main effect of group

but no group by task interaction. Overall, the XXY group performed less well than

both control groups, but performance did not differ significantly as a function

of task. ADHD comorbidity in males with XXY was related to poorer EF skills.

FAU - Lee, Nancy Raitano

AU - Lee NR

AD - Child Psychiatry Branch, National Institute of Mental Health, NIH, Bethesda,

Maryland.

FAU - Wallace, Gregory L

AU - Wallace GL

AD - Child Psychiatry Branch, National Institute of Mental Health, NIH, Bethesda,

Maryland.

FAU - Clasen, Liv S

AU - Clasen LS

AD - Child Psychiatry Branch, National Institute of Mental Health, NIH, Bethesda,

Maryland.

FAU - Lenroot, Rhoshel K

AU - Lenroot RK

AD - Child Psychiatry Branch, National Institute of Mental Health, NIH, Bethesda,

Maryland.

FAU - Blumenthal, Jonathan D

AU - Blumenthal JD

AD - Child Psychiatry Branch, National Institute of Mental Health, NIH, Bethesda,

Maryland.

FAU - White, Samantha L

AU - White SL

AD - Child Psychiatry Branch, National Institute of Mental Health, NIH, Bethesda,

Maryland.

FAU - Celano, Mark J

AU - Celano MJ

AD - Child Psychiatry Branch, National Institute of Mental Health, NIH, Bethesda,

Maryland.

FAU - Giedd, Jay N

AU - Giedd JN

AD - Child Psychiatry Branch, National Institute of Mental Health, NIH, Bethesda,

Maryland.

LA - eng

GR - Z99 MH999999/ImNIH/Intramural NIH HHS/United States

PT - Journal Article

PT - Research Support, N.I.H., Intramural

TA - J Int Neuropsychol Soc

JT - Journal of the International Neuropsychological Society : JINS

JID - 9503760

SB - IM

MH - Adolescent

MH - Adult

MH - Analysis of Variance

MH - Attention Deficit Disorder with Hyperactivity/*complications/*epidemiology

MH - Child

MH - Cognition Disorders/*etiology

MH - Executive Function/*physiology

MH - Follow-Up Studies

MH - Humans

MH - Intelligence

MH - Klinefelter Syndrome/*complications/*epidemiology

MH - Male

MH - Neuropsychological Tests

MH - Psychiatric Status Rating Scales

MH - Young Adult

PMC - PMC3340493

MID - NIHMS347435

EDAT- 2011/03/23 06:00

MHDA- 2015/04/07 06:00

CRDT- 2011/03/23 06:00

PHST- 2011/03/23 06:00 [entrez]

PHST- 2011/03/23 06:00 [pubmed]

PHST- 2015/04/07 06:00 [medline]

AID - S1355617711000312 [pii]

AID - 10.1017/S1355617711000312 [doi]

PST - ppublish

SO - J Int Neuropsychol Soc. 2011 May;17(3):522-30. doi: 10.1017/S1355617711000312.

PMID- 22412026

OWN - NLM

STAT- MEDLINE

DCOM- 20120613

LR - 20211021

IS - 1098-4275 (Electronic)

IS - 0031-4005 (Print)

IS - 0031-4005 (Linking)

VI - 129

IP - 4

DP - 2012 Apr

TI - Behavioral and social phenotypes in boys with 47,XYY syndrome or 47,XXY

Klinefelter syndrome.

PG - 769-78

LID - 10.1542/peds.2011-0719 [doi]

AB - OBJECTIVE: To contrast the behavioral and social phenotypes including a screen

for autistic behaviors in boys with 47,XYY syndrome (XYY) or 47,XXY Klinefelter

syndrome (KS) and controls and investigate the effect of prenatal diagnosis on

the phenotype. METHODS: Patients included 26 boys with 47,XYY, 82 boys with KS,

and 50 control boys (ages 4-15 years). Participants and parents completed a

physical examination, behavioral questionnaires, and intellectual assessments.

RESULTS: Most boys with XYY or KS had Child Behavior Checklist parental ratings

within the normal range. On the Child Behavior Checklist, mean problem behaviors

t scores were higher in the XYY versus KS groups for the Problem Behavior,

Externalizing, Withdrawn, Thought Problems, and Attention Problems subscales. On

the Conners' Parent Rating Scale-Revised, the XYY versus KS group had increased

frequency of hyperactive/impulsive symptoms (P < .006). In addition, 50% and 12%

of the XYY and KS groups, respectively, had scores >15 for autism screening from

the Social Communication Questionnaire. For the boys with KS, prenatal diagnosis

was associated with fewer problem behaviors. CONCLUSIONS: A subset of the XYY and

KS groups had behavioral difficulties that were more severe in the XYY group.

These findings could guide clinical practice and inform patients and parents.

Boys diagnosed with XYY or KS should receive a comprehensive psychoeducational

evaluation and be screened for learning disabilities,

attention-deficit/hyperactivity disorder, and autism spectrum disorders.

FAU - Ross, Judith L

AU - Ross JL

AD - Department of Pediatrics, Thomas Jefferson University, Philadelphia, PA 19107,

USA. judith.ross@jefferson.edu

FAU - Roeltgen, David P

AU - Roeltgen DP

FAU - Kushner, Harvey

AU - Kushner H

FAU - Zinn, Andrew R

AU - Zinn AR

FAU - Reiss, Allan

AU - Reiss A

FAU - Bardsley, Martha Zeger

AU - Bardsley MZ

FAU - McCauley, Elizabeth

AU - McCauley E

FAU - Tartaglia, Nicole

AU - Tartaglia N

LA - eng

GR - K23 NS070337/NS/NINDS NIH HHS/United States

GR - R01 NS050597/NS/NINDS NIH HHS/United States

GR - R01NS 050597/NS/NINDS NIH HHS/United States

PT - Comparative Study

PT - Journal Article

PT - Research Support, N.I.H., Extramural

PT - Research Support, Non-U.S. Gov't

DEP - 20120312

TA - Pediatrics

JT - Pediatrics

JID - 0376422

SB - IM

MH - Adolescent

MH - *Adolescent Behavior

MH - Attention Deficit Disorder with Hyperactivity/diagnosis/*genetics

MH - Child

MH - *Child Behavior

MH - Child Development Disorders, Pervasive/diagnosis/*genetics

MH - Child, Preschool

MH - Genetic Testing/*methods

MH - Humans

MH - Karyotyping

MH - Klinefelter Syndrome/diagnosis/*genetics

MH - Male

MH - Neuropsychological Tests

MH - Phenotype

MH - XYY Karyotype/diagnosis/*genetics

PMC - PMC3356148

EDAT- 2012/03/14 06:00

MHDA- 2012/06/14 06:00

CRDT- 2012/03/14 06:00

PHST- 2012/03/14 06:00 [entrez]

PHST- 2012/03/14 06:00 [pubmed]

PHST- 2012/06/14 06:00 [medline]

AID - peds.2011-0719 [pii]

AID - 10.1542/peds.2011-0719 [doi]

PST - ppublish

SO - Pediatrics. 2012 Apr;129(4):769-78. doi: 10.1542/peds.2011-0719. Epub 2012 Mar

12.

PMID- 20573461

OWN - NLM

STAT- MEDLINE

DCOM- 20110913

LR - 20110516

IS - 1872-7131 (Electronic)

IS - 0387-7604 (Linking)

VI - 33

IP - 6

DP - 2011 Jun

TI - ADHD and genetic syndromes.

PG - 456-61

LID - 10.1016/j.braindev.2010.05.011 [doi]

AB - A high rate of Attention Deficit/Hyperactivity Disorder (ADHD)-like

characteristics has been reported in a wide variety of disorders including

syndromes with known genetic causes. In this article, we review the genetic and

the neurobiological links between ADHD symptoms and some genetic syndromes such

as: Fragile X Syndrome, Neurofibromatosis 1, DiGeorge Syndrome, Tuberous

Sclerosis Complex, Turner Syndrome, Williams Syndrome and Klinefelter Syndrome.

Although each syndrome may arise from different genetic abnormalities with

multiple molecular functions, the effects of these abnormalities may give rise to

common effects downstream in the biological pathways or neural circuits,

resulting in the presentation of ADHD symptoms. Early diagnosis of ADHD allows

for earlier treatment, and has the potential for a better outcome in children

with genetic syndromes.

CI - Copyright © 2010 The Japanese Society of Child Neurology. Published by Elsevier

B.V. All rights reserved.

FAU - Lo-Castro, Adriana

AU - Lo-Castro A

AD - Department of Neuroscience, Paediatric Neurology Unit, "Tor Vergata" University

of Rome, Italy. a.locastro@libero.it

FAU - D'Agati, Elisa

AU - D'Agati E

FAU - Curatolo, Paolo

AU - Curatolo P

LA - eng

PT - Journal Article

PT - Review

DEP - 20100622

PL - Netherlands

TA - Brain Dev

JT - Brain & development

JID - 7909235

SB - IM

CIN - Brain Dev. 2011 Jun;33(6):454-5. PMID: 21515008

MH - Attention Deficit Disorder with

Hyperactivity/diagnosis/etiology/*genetics/physiopathology

MH - Child

MH - Humans

MH - Phenotype

MH - Syndrome

EDAT- 2010/06/25 06:00

MHDA- 2011/09/14 06:00

CRDT- 2010/06/25 06:00

PHST- 2010/01/17 00:00 [received]

PHST- 2010/05/26 00:00 [revised]

PHST- 2010/05/27 00:00 [accepted]

PHST- 2010/06/25 06:00 [entrez]

PHST- 2010/06/25 06:00 [pubmed]

PHST- 2011/09/14 06:00 [medline]

AID - S0387-7604(10)00130-0 [pii]

AID - 10.1016/j.braindev.2010.05.011 [doi]

PST - ppublish

SO - Brain Dev. 2011 Jun;33(6):456-61. doi: 10.1016/j.braindev.2010.05.011. Epub 2010

Jun 22.

PMID- 34240550

OWN - NLM

STAT- MEDLINE

DCOM- 20220303

LR - 20220731

IS - 1552-4833 (Electronic)

IS - 1552-4825 (Print)

IS - 1552-4825 (Linking)

VI - 185

IP - 12

DP - 2021 Dec

TI - Early developmental impact of sex chromosome trisomies on attention

deficit-hyperactivity disorder symptomology in young children.

PG - 3664-3674

LID - 10.1002/ajmg.a.62418 [doi]

AB - Individuals with sex chromosome trisomies ([SCT], XXX, XXY, and XYY)) are at

increased risk for neurodevelopmental problems, given that a significant portion

of the sex chromosome genes impact brain functioning. An elevated risk for

psychopathology has also been described, including attention

deficit-hyperactivity disorder (ADHD). The present study aimed at identifying

early markers of ADHD, providing the first investigation of ADHD symptomology in

very young children with SCT. The variety, type, and severity of ADHD

symptomology in 1-6-year-old children with SCT (n = 104) were compared with

population-based controls (n = 101) using the strengths and weaknesses of ADHD

symptoms and normal-behavior (SWAN) parent-report questionnaire. ADHD

symptomology was significantly more prevalent in SCT and already present from

toddlerhood on, compared to controls. ADHD inattention symptoms were

significantly increased in all karyotypes (XXX, XXY, and XYY), boys with XYY also

showed significantly more hyperactivity/impulsivity symptoms than controls.

Inattentiveness was more pronounced with increasing age for SCT, in contrast to

controls. Within the SCT group, 24% of the children had significantly elevated

ADHD symptoms at a clinical level. Already from an early age on, SCT is

associated with a risk for ADHD, suggesting that its neurodevelopmental risk lies

anchored in early brain maturation. Studying this genetically vulnerable

population allows for the prospective study of risk markers to facilitate early

and preventive interventions.

CI - © 2021 The Authors. American Journal of Medical Genetics Part A published by

Wiley Periodicals LLC.

FAU - Kuiper, Kimberly

AU - Kuiper K

AUID- ORCID: 0000-0002-6429-5835

AD - Clinical Neurodevelopmental Sciences, Leiden University, Leiden, The Netherlands.

AD - Leiden Institute for Brain and Cognition, Leiden, The Netherlands.

FAU - Swaab, Hanna

AU - Swaab H

AD - Clinical Neurodevelopmental Sciences, Leiden University, Leiden, The Netherlands.

AD - Leiden Institute for Brain and Cognition, Leiden, The Netherlands.

FAU - Tartaglia, Nicole

AU - Tartaglia N

AD - eXtraordinarY Kids Clinic, Developmental Pediatrics, Children's Hospital

Colorado, Aurora, Colorado, USA.

AD - Department of Pediatrics, University of Colorado School of Medicine, Aurora,

Colorado, USA.

FAU - van Rijn, Sophie

AU - van Rijn S

AD - Clinical Neurodevelopmental Sciences, Leiden University, Leiden, The Netherlands.

AD - Leiden Institute for Brain and Cognition, Leiden, The Netherlands.

LA - eng

GR - UL1 TR002535/TR/NCATS NIH HHS/United States

GR - UL1 TR002535/NH/NIH HHS/United States

PT - Journal Article

PT - Research Support, N.I.H., Extramural

PT - Research Support, Non-U.S. Gov't

DEP - 20210708

TA - Am J Med Genet A

JT - American journal of medical genetics. Part A

JID - 101235741

SB - IM

MH - Abnormal Karyotype

MH - Attention Deficit Disorder with Hyperactivity/*diagnosis/genetics/physiopathology

MH - Child

MH - Child, Preschool

MH - Cognition Disorders/*diagnosis/genetics/physiopathology

MH - Female

MH - Humans

MH - Infant

MH - Male

MH - *Sex Chromosome Aberrations

MH - Sex Chromosomes/genetics

MH - Trisomy/*genetics

MH - XYY Karyotype/genetics

PMC - PMC9292447

OTO - NOTNLM

OT - *ADHD

OT - *Klinefelter syndrome

OT - *developmental psychopathology

OT - *sex chromosomes

OT - *trisomy X syndrome

COIS- The authors declare no conflicts of interest.

EDAT- 2021/07/10 06:00

MHDA- 2022/03/04 06:00

CRDT- 2021/07/09 07:25

PHST- 2021/05/14 00:00 [revised]

PHST- 2021/02/23 00:00 [received]

PHST- 2021/06/18 00:00 [accepted]

PHST- 2021/07/10 06:00 [pubmed]

PHST- 2022/03/04 06:00 [medline]

PHST- 2021/07/09 07:25 [entrez]

AID - AJMGA62418 [pii]

AID - 10.1002/ajmg.a.62418 [doi]

PST - ppublish

SO - Am J Med Genet A. 2021 Dec;185(12):3664-3674. doi: 10.1002/ajmg.a.62418. Epub

2021 Jul 8.

PMID- 21414026

OWN - NLM

STAT- MEDLINE

DCOM- 20110809

LR - 20220409

IS - 1651-2227 (Electronic)

IS - 0803-5253 (Linking)

VI - 100

IP - 6

DP - 2011 Jun

TI - Morbidity and mortality in Klinefelter syndrome (47,XXY).

PG - 807-13

LID - 10.1111/j.1651-2227.2011.02274.x [doi]

AB - Klinefelter syndrome (KS) (47,XXY) is the most common sex chromosome disorder in

man and is a relatively common cause of male infertility and hypogonadism. The

syndrome has been known since 1942, and many reports of different diseases

associated with KS have been reported since that, but a more systematic knowledge

about the long-term outcome was not described until the last decade, where

nation-wide epidemiological studies were reported from Britain and Denmark. We

here review the epidemiological data from two cohorts of patients with KS in

Denmark and Britain, showing a significant increase in both mortality and

morbidity from a variety of different causes. Mortality was increased by 50% (SMR

1.5 or HR 1.4) corresponding to a median loss of approximately 2 years. The risk

of being admitted to hospital with any diagnosis was increased by 70%. The

underlying reason for the poorer health in KS may be caused by interaction of

genetic, hormonal and socio-economic factors. CONCLUSION: Both morbidity and

mortality are significantly increased in Klinefelter syndrome with a 50% increase

in mortality risk and a 70% increase in risk of being admitted to hospital.

CI - © 2011 The Author(s)/Acta Paediatrica © 2011 Foundation Acta Paediatrica.

FAU - Bojesen, Anders

AU - Bojesen A

AD - Department of Clinical Genetics, Vejle Hospital, Sygehus Lillebaelt, Denmark.

anders.bojesen@dadlnet.dk

FAU - Gravholt, Claus H

AU - Gravholt CH

LA - eng

PT - Journal Article

PT - Review

DEP - 20110406

PL - Norway

TA - Acta Paediatr

JT - Acta paediatrica (Oslo, Norway : 1992)

JID - 9205968

SB - IM

MH - Denmark/epidemiology

MH - Hospitalization/statistics & numerical data

MH - Humans

MH - Klinefelter Syndrome/*epidemiology/mortality

MH - Male

MH - Morbidity

MH - Neoplasms/epidemiology

MH - Risk

MH - United Kingdom/epidemiology

EDAT- 2011/03/19 06:00

MHDA- 2011/08/10 06:00

CRDT- 2011/03/19 06:00

PHST- 2011/03/19 06:00 [entrez]

PHST- 2011/03/19 06:00 [pubmed]

PHST- 2011/08/10 06:00 [medline]

AID - 10.1111/j.1651-2227.2011.02274.x [doi]

PST - ppublish

SO - Acta Paediatr. 2011 Jun;100(6):807-13. doi: 10.1111/j.1651-2227.2011.02274.x.

Epub 2011 Apr 6.

PMID- 9645824

OWN - NLM

STAT- MEDLINE

DCOM- 19980709

LR - 20190812

IS - 0003-9926 (Print)

IS - 0003-9926 (Linking)

VI - 158

IP - 12

DP - 1998 Jun 22

TI - Klinefelter syndrome.

PG - 1309-14

AB - Klinefelter syndrome is the most common sex chromosome disorder. Affected males

carry an additional X chromosome, which results in male hypogonadism, androgen

deficiency, and impaired spermatogenesis. Some patients may exhibit all of the

classic signs of this disorder, including gynecomastia, small testes, sparse body

hair, tallness, and infertility, whereas others, because of the wide variability

in clinical expression, lack many of these features. Treatment consists of

testosterone replacement therapy to correct the androgen deficiency and to

provide patients with appropriate virilization. This therapy also has positive

effects on mood and self-esteem and has been shown to protect against

osteoporosis, although it will not reverse infertility. Although the diagnosis of

Klinefelter syndrome is now made definitively using chromosomal karyotyping,

revealing in most instances a 47,XXY genotype, the diagnosis also can be made

using a careful history and results of a physical examination, with the hallmark

being small, firm testes. As it affects 1 in 500 male patients and presents with

a variety of clinical features, primary care physicians should be familiar with

this condition.

FAU - Smyth, C M

AU - Smyth CM

AD - Department of Medicine, Veterans Affairs Puget Sound Health Care System, Seattle

98108, USA.

FAU - Bremner, W J

AU - Bremner WJ

LA - eng

PT - Journal Article

PT - Review

PL - United States

TA - Arch Intern Med

JT - Archives of internal medicine

JID - 0372440

SB - IM

MH - Diagnosis, Differential

MH - Humans

MH - Incidence

MH - *Klinefelter Syndrome/complications/diagnosis/epidemiology/genetics/therapy

MH - Male

MH - Mass Screening

RF - 69

EDAT- 1998/06/30 00:00

MHDA- 1998/06/30 00:01

CRDT- 1998/06/30 00:00

PHST- 1998/06/30 00:00 [pubmed]

PHST- 1998/06/30 00:01 [medline]

PHST- 1998/06/30 00:00 [entrez]

AID - 10.1001/archinte.158.12.1309 [doi]

PST - ppublish

SO - Arch Intern Med. 1998 Jun 22;158(12):1309-14. doi: 10.1001/archinte.158.12.1309.

PMID- 33000452

OWN - NLM

STAT- MEDLINE

DCOM- 20210922

LR - 20210922

IS - 2520-8721 (Electronic)

IS - 1109-3099 (Linking)

VI - 19

IP - 4

DP - 2020 Dec

TI - Oncologic manifestations of Klinefelter syndrome.

PG - 497-504

LID - 10.1007/s42000-020-00241-7 [doi]

AB - Klinefelter syndrome (47,XXY) has a prevalence of approximately 1 in 500 males.

It is a condition characterized by an extra X chromosome and is an underdiagnosed

clinical entity. Inactivation of genes enables their escape from regulatory

mechanisms, which can result in such classic physical manifestations as

hypogonadism, gynecomastia, infertility, and various hormonal and physical

abnormalities. While the endocrine manifestations of 47,XXY are well-known, the

oncologic manifestations have received less attention. An association between

cancer and 47,XXY has not as yet been clearly defined, with variability noted in

the prevalence of different malignancies in 47,XXY patients. The mechanisms

underlying these altered oncologic risks are still under debate. Some of the

proposed explanations include hormone imbalance, developmental malfunctions, and

failed DNA repair mechanisms. However, the recognition of the oncological

associations linked to 47,XXY could be helpful. Screening measures in certain

malignancies may enable an earlier diagnosis of 47,XXY and the implementation of

more customized care in 47,XXY and the mosaic variants.. The data for this review

was compiled from relevant PubMed articles published within the last three

decades and organized based on cancer type.

FAU - Rojas, Alexsandra P

AU - Rojas AP

AUID- ORCID: 0000-0002-8870-7275

AD - School of Medicine, Texas Tech University Health Sciences Center, Lubbock, TX,

USA. alex.rojas@ttuhsc.edu.

AD - , Lubbock, TX, USA. alex.rojas@ttuhsc.edu.

FAU - Vo, Diana V

AU - Vo DV

AD - School of Medicine, Texas Tech University Health Sciences Center, Lubbock, TX,

USA.

FAU - Mwangi, Lance

AU - Mwangi L

AD - School of Medicine, Texas Tech University Health Sciences Center, Lubbock, TX,

USA.

FAU - Rehman, Shabnam

AU - Rehman S

AD - Oncology Division of Internal medicine, Texas Tech University Health Sciences

Center, Lubbock, TX, USA.

FAU - Peiris, Alan N

AU - Peiris AN

AD - Clinical Research Institute and Department of Internal Medicine, Texas Tech

University Health Sciences Center, Lubbock, TX, USA.

LA - eng

PT - Journal Article

PT - Review

DEP - 20201001

PL - Switzerland

TA - Hormones (Athens)

JT - Hormones (Athens, Greece)

JID - 101142469

SB - IM

MH - Humans

MH - Klinefelter Syndrome/*complications/diagnosis/genetics/metabolism

MH - Male

MH - Neoplasms/diagnosis/*etiology/metabolism

OTO - NOTNLM

OT - Cancer

OT - Karyotyping

OT - Klinefelter syndrome

OT - Underdiagnosis

EDAT- 2020/10/02 06:00

MHDA- 2021/09/23 06:00

CRDT- 2020/10/01 05:41

PHST- 2019/08/03 00:00 [received]

PHST- 2020/09/01 00:00 [accepted]

PHST- 2020/10/02 06:00 [pubmed]

PHST- 2021/09/23 06:00 [medline]

PHST- 2020/10/01 05:41 [entrez]

AID - 10.1007/s42000-020-00241-7 [pii]

AID - 10.1007/s42000-020-00241-7 [doi]

PST - ppublish

SO - Hormones (Athens). 2020 Dec;19(4):497-504. doi: 10.1007/s42000-020-00241-7. Epub

2020 Oct 1.

PMID- 9160389

OWN - NLM

STAT- MEDLINE

DCOM- 19970716

LR - 20220408

IS - 0197-3851 (Print)

IS - 0197-3851 (Linking)

VI - 17

IP - 4

DP - 1997 Apr

TI - 47,XXY (Klinefelter syndrome) and 47,XYY: estimated rates of and indication for

postnatal diagnosis with implications for prenatal counselling.

PG - 363-8

AB - Cytogenetic surveys of neonates have found that approximately one boy in 500 is

born with an extra sex chromosome. Some of these boys are now being diagnosed

when prenatal karyotyping is done for the detection of Down syndrome and other

major aneuploidies. This study estimates what proportion of those not detected

prenatally will be diagnosed postnatally and what the indications for karyotyping

are likely to be. We ascertained all 47,XXY and 47,XYY males detected prenatally

and postnatally (during the 4 years 1990-1993) in the three cytogenetic

laboratories in the North Thames (West) region. The age at diagnosis and

indication for karyotyping were noted for cases diagnosed postnatally. Less than

10 per cent of the estimated number of affected fetuses were detected prenatally.

This study suggests that most males born with these chromosome patterns will go

through life without being karyotyped, that the commonest indication for a 47,XYY

male to be karyotyped will be developmental delay and/or behaviour problems, and

that the commonest indication for a Klinefelter male to be karyotyped will be

hypogonadism and/or infertility. It would appear that most undiagnosed 47,XXY and

47,XYY males do not look or behave in a manner which prompts testing for a

chromosome abnormality.

FAU - Abramsky, L

AU - Abramsky L

AD - North Thames (West) Congenital Malformation Register, Northwick Park, U.K.

FAU - Chapple, J

AU - Chapple J

LA - eng

PT - Journal Article

PT - Research Support, Non-U.S. Gov't

PL - England

TA - Prenat Diagn

JT - Prenatal diagnosis

JID - 8106540

SB - IM

CIN - Prenat Diagn. 1998 Mar;18(3):303-4. PMID: 9556051

MH - Abortion, Eugenic

MH - Adolescent

MH - Adult

MH - Child

MH - Child, Preschool

MH - Female

MH - *Genetic Counseling

MH - Humans

MH - Infant, Newborn

MH - Karyotyping

MH - Klinefelter Syndrome/complications/*diagnosis/epidemiology

MH - Male

MH - Pregnancy

MH - *Prenatal Diagnosis

MH - United Kingdom/epidemiology

MH - XYY Karyotype/*diagnosis/epidemiology

EDAT- 1997/04/01 00:00

MHDA- 2000/06/20 09:00

CRDT- 1997/04/01 00:00

PHST- 1997/04/01 00:00 [pubmed]

PHST- 2000/06/20 09:00 [medline]

PHST- 1997/04/01 00:00 [entrez]

AID - 10.1002/(SICI)1097-0223(199704)17:4<363::AID-PD79>3.0.CO;2-O [pii]

AID - 10.1002/(sici)1097-0223(199704)17:4<363::aid-pd79>3.0.co;2-o [doi]

PST - ppublish

SO - Prenat Diagn. 1997 Apr;17(4):363-8. doi:

10.1002/(sici)1097-0223(199704)17:4<363::aid-pd79>3.0.co;2-o.

PMID- 34522767

OWN - NLM

STAT- PubMed-not-MEDLINE

LR - 20220426

IS - 2376-0605 (Electronic)

IS - 2376-0605 (Linking)

VI - 7

IP - 5

DP - 2021 Sep-Oct

TI - Occurrence of Klinefelter Syndrome Mosaic 45,X/46,XY/47,XXY/48,XXYY/48,XXXY and

Primary Hyperparathyroidism.

PG - 293-298

LID - 10.1016/j.aace.2021.03.001 [doi]

AB - OBJECTIVE: The presence of primary hyperparathyroidism (PHPT) and Klinefelter

syndrome (KS) is rare, and its association with KS mosaicism is even rarer. We

report an unusual combination of these entities with a mild phenotype of KS.

METHODS: The patient was a 44-year-old male with a history of PHPT who had

recurrent urolithiasis despite being treated with a successful parathyroidectomy.

On examination, he had axillary hair growth, bilateral gynecomastia, a large

port-wine stain at the right hemithorax and upper right limb, and genitalia and

pubic hair corresponding to Tanner IV classification with small, normal

consistency testicles. RESULTS: Laboratory findings were unremarkable except for

a slightly elevated luteinizing hormone, which was normal on repeat testing.

Because of the picture of unexplained gynecomastia, laboratory findings, and

low-volume testis, a diagnosis of KS was considered. Chromosomal analysis

revealed a rare 45,X/46,XY/47,XXY/48,XXYY/48,XXXY KS mosaic. CONCLUSIONS: KS

phenotypes are largely variable, and their association with PHPT remains to be

elucidated.

CI - © 2021 AACE. Published by Elsevier Inc.

FAU - Lam-Chung, César Ernesto

AU - Lam-Chung CE

AD - Department of Endocrinology and Metabolism, Instituto Nacional de Ciencias

Médicas y Nutrición Salvador Zubirán, México City, México.

FAU - Rodríguez, Larissa López

AU - Rodríguez LL

AD - Department of Genetics, Instituto Nacional de Ciencias Médicas y Nutrición

Salvador Zubirán, México City, México.

FAU - Kato, Yayoi Segura

AU - Kato YS

AD - Unit of Molecular Biology and Genomic Medicine, Instituto Nacional de Ciencias

Médicas y Nutrición Salvador Zubirán, México City, México.

FAU - Jiménez González, Iván Josué

AU - Jiménez González IJ

AD - Department of Genetics, Instituto Nacional de Ciencias Médicas y Nutrición

Salvador Zubirán, México City, México.

FAU - Mena-Hernández, Lourdes

AU - Mena-Hernández L

AD - Department of Dermatology. Instituto Nacional de Ciencias Médicas y Nutrición

Salvador Zubirán, México City, México.

FAU - Rivera-Juárez, Renata

AU - Rivera-Juárez R

AD - Department of Genetics, Instituto Nacional de Ciencias Médicas y Nutrición

Salvador Zubirán, México City, México.

FAU - Almeda-Valdes, Paloma

AU - Almeda-Valdes P

AD - Department of Endocrinology and Metabolism, Instituto Nacional de Ciencias

Médicas y Nutrición Salvador Zubirán, México City, México.

FAU - Vázquez, Jazmín Arteaga

AU - Vázquez JA

AD - Department of Genetics, Instituto Nacional de Ciencias Médicas y Nutrición

Salvador Zubirán, México City, México.

LA - eng

PT - Case Reports

DEP - 20210313

TA - AACE Clin Case Rep

JT - AACE clinical case reports

JID - 101670593

PMC - PMC8426603

OTO - NOTNLM

OT - KS, Klinefelter syndrome

OT - Klinefelter syndrome

OT - LH, luteinizing hormone

OT - PHPT, primary hyperparathyroidism

OT - PTH, parathyroid hormone

OT - hyperparathyroidism

OT - mosaicism

OT - nephrolithiasis

EDAT- 2021/09/16 06:00

MHDA- 2021/09/16 06:01

CRDT- 2021/09/15 07:19

PHST- 2021/02/08 00:00 [received]

PHST- 2021/03/01 00:00 [accepted]

PHST- 2021/09/15 07:19 [entrez]

PHST- 2021/09/16 06:00 [pubmed]

PHST- 2021/09/16 06:01 [medline]

AID - S2376-0605(21)00041-9 [pii]

AID - 10.1016/j.aace.2021.03.001 [doi]

PST - epublish

SO - AACE Clin Case Rep. 2021 Mar 13;7(5):293-298. doi: 10.1016/j.aace.2021.03.001.

eCollection 2021 Sep-Oct.

PMID- 31695472

OWN - NLM

STAT- PubMed-not-MEDLINE

LR - 20200929

IS - 1178-704X (Print)

IS - 1178-704X (Electronic)

IS - 1178-704X (Linking)

VI - 12

DP - 2019

TI - Update On The Clinical Perspectives And Care Of The Child With 47,XXY

(Klinefelter Syndrome).

PG - 191-202

LID - 10.2147/TACG.S180450 [doi]

AB - 47,XXY (Klinefelter syndrome [KS]) is the most common sex chromosomal aneuploidy

(1:660), yet, despite this, only 25% of the males are ever diagnosed. Males with

47,XXY present with characteristic symptoms throughout their lifetime with

typical physical and neurodevelopmental manifestations focused in growth,

cognitive development, endocrine function, and reproduction. Studies have

demonstrated that optimal outcomes are dependent on early detection combined with

consistent and targeted neurodevelopmental treatment throughout the lifespan.

During infancy and into the preschool years, individuals with 47,XXY commonly

face deficits in growth and development in the areas of early hormonal, motor,

speech, and behavioral development. As they transition into school, the primary

neurodevelopmental concerns include language difficulty, executive dysfunction,

behavior, and learning and reading deficits. Adults with 47,XXY often present

with taller than average height, low levels of fertility, azoospermia, and

elevated gonadotropin levels. These presentations may persist from early

childhood through adulthood but can be mitigated by appropriate interventions.

Early neurodevelopmental and hormonal treatment has been shown to have a

minimizing effect on the physical and neurodevelopmental manifestations in

individuals with 47,XXY. With innovative and current research studies, the

features common to the neurodevelopmental profile of 47,XXY have been further

expanded and defined. Further research is necessary to elucidate and understand

the relationship between the brain, behavior, and the phenotypic profile of

47,XXY.

CI - © 2019 Samango-Sprouse et al.

FAU - Samango-Sprouse, Carole A

AU - Samango-Sprouse CA

AUID- ORCID: 0000-0001-9941-0568

AD - Department of Pediatrics, George Washington University, Washington, DC, USA.

AD - Department of Human and Molecular Genetics, Florida International University,

Miami, FL, USA.

AD - The Focus Foundation, Davidsonville, MD, USA.

FAU - Counts, Debra R

AU - Counts DR

AD - Pediatric Endocrinology, Sinai Hospital, Baltimore, MD, USA.

FAU - Tran, Selena L

AU - Tran SL

AD - The Focus Foundation, Davidsonville, MD, USA.

FAU - Lasutschinkow, Patricia C

AU - Lasutschinkow PC

AD - The Focus Foundation, Davidsonville, MD, USA.

FAU - Porter, Grace F

AU - Porter GF

AD - The Focus Foundation, Davidsonville, MD, USA.

FAU - Gropman, Andrea L

AU - Gropman AL

AUID- ORCID: 0000-0002-2106-6776

AD - Department of Neurology, George Washington University, Washington, DC, USA.

AD - Division of Neurogenetics and Developmental Pediatrics, Children's National

Medical Center, Washington, DC, USA.

LA - eng

PT - Journal Article

PT - Review

DEP - 20191023

TA - Appl Clin Genet

JT - The application of clinical genetics

JID - 101579789

PMC - PMC6815760

OTO - NOTNLM

OT - 47

OT - Klinefelter syndrome

OT - XXY

OT - hormonal treatment

OT - neurodevelopment

COIS- The authors report no conflicts of interest in this work.

EDAT- 2019/11/07 06:00

MHDA- 2019/11/07 06:01

CRDT- 2019/11/08 06:00

PHST- 2019/06/01 00:00 [received]

PHST- 2019/09/20 00:00 [accepted]

PHST- 2019/11/08 06:00 [entrez]

PHST- 2019/11/07 06:00 [pubmed]

PHST- 2019/11/07 06:01 [medline]

AID - 180450 [pii]

AID - 10.2147/TACG.S180450 [doi]

PST - epublish

SO - Appl Clin Genet. 2019 Oct 23;12:191-202. doi: 10.2147/TACG.S180450. eCollection

2019.

PMID- 19515177

OWN - NLM

STAT- MEDLINE

DCOM- 20090924

LR - 20220409

IS - 1365-2605 (Electronic)

IS - 0105-6263 (Linking)

VI - 32

IP - 4

DP - 2009 Aug

TI - Low semen volume in 47 adolescents and adults with 47,XXY Klinefelter or 46,XX

male syndrome.

PG - 376-84

LID - 10.1111/j.1365-2605.2008.00921.x [doi]

AB - Klinefelter syndrome is characterized by progressive testicular failure causing

androgen deficiency and azoospermia in most patients. The aim of this study was

to evaluate semen quality in consecutive patients with an additional X chromosome

as compared with healthy males. Forty-seven males with non-mosaic 47,XXY (n = 40)

or SRY-positive 46,XX male (n = 7) karyotypes aged 26.1 (range: 15.0-51.7) years

participated. Semen quality was compared with 2136 (control group I) men from the

general population aged 18.9 (17.9-28.6) years and with 349 fertile men (control

group II) aged 30.9 (22.0-43.8) years. Semen volume adjusted for duration of

abstinence was significantly smaller in the patients [2.0 (0.2-5.7) mL] when

compared with control group I [3.1 (0.3-12.5) mL, p < 0.0001] and group II [3.6

(0.6-12.5) mL, p < 0.0001]. There was no difference in semen volume between

47,XXY and 46,XX males. All patients had azoospermia except two 47,XXY males aged

29 years who had sperm concentrations of 0.5 and 1.6 million/mL, respectively. We

found significantly smaller semen volume in the patients when compared with

controls, and the presence of motile spermatozoa in two out of 47 patients. The

small semen volume supports the notion of 47,XXY patients being androgen

insufficient despite serum testosterone levels within the normal range.

FAU - Aksglaede, L

AU - Aksglaede L

AD - University Department of Growth and Reproduction, University of Copenhagen,

Rigshospitalet, Copenhagen, Denmark.

FAU - Jørgensen, N

AU - Jørgensen N

FAU - Skakkebaek, N E

AU - Skakkebaek NE

FAU - Juul, A

AU - Juul A

LA - eng

PT - Journal Article

PT - Research Support, Non-U.S. Gov't

DEP - 20081021

PL - England

TA - Int J Androl

JT - International journal of andrology

JID - 8000141

RN - 0 (Biomarkers)

RN - 3XMK78S47O (Testosterone)

RN - 9002-67-9 (Luteinizing Hormone)

SB - IM

MH - Adolescent

MH - Adult

MH - Azoospermia/blood/genetics/*pathology

MH - Biomarkers/blood

MH - Case-Control Studies

MH - *Chromosomes, Human, X

MH - *Chromosomes, Human, Y

MH - Humans

MH - Klinefelter Syndrome/blood/genetics/*pathology

MH - Luteinizing Hormone/blood

MH - Male

MH - *Semen Analysis

MH - Sperm Count

MH - Sperm Motility

MH - Testosterone/blood

MH - Young Adult

EDAT- 2009/06/12 09:00

MHDA- 2009/09/25 06:00

CRDT- 2009/06/12 09:00

PHST- 2009/06/12 09:00 [entrez]

PHST- 2009/06/12 09:00 [pubmed]

PHST- 2009/09/25 06:00 [medline]

AID - IJA921 [pii]

AID - 10.1111/j.1365-2605.2008.00921.x [doi]

PST - ppublish

SO - Int J Androl. 2009 Aug;32(4):376-84. doi: 10.1111/j.1365-2605.2008.00921.x. Epub

2008 Oct 21.

PMID- 24416272

OWN - NLM

STAT- MEDLINE

DCOM- 20140910

LR - 20211021

IS - 1932-6203 (Electronic)

IS - 1932-6203 (Linking)

VI - 9

IP - 1

DP - 2014

TI - Social attention, affective arousal and empathy in men with Klinefelter syndrome

(47,XXY): evidence from eyetracking and skin conductance.

PG - e84721

LID - 10.1371/journal.pone.0084721 [doi]

LID - e84721

AB - Individuals with an extra X chromosome (Klinefelter syndrome) are at risk for

problems in social functioning and have an increased vulnerability for autism

traits. In the search for underlying mechanisms driving this increased risk, this

study focused on social attention, affective arousal and empathy. Seventeen

adults with XXY and 20 non-clinical controls participated in this study.

Eyetracking was used to investigate social attention, as expressed in visual

scanning patterns in response to the viewing of empathy evoking video clips. Skin

conductance levels, reflecting affective arousal, were recorded continuously

during the clips as well. Empathic skills, i.e. participants' understanding of

own and others' emotions in response to the clips was also assessed. Results

showed reduced empathic understanding, decreased visual fixation to the eye

region, but increased affective arousal in individuals with Klinefelter syndrome.

We conclude that individuals with XXY tend to avoid the eye region. Considering

the increased affective arousal, we speculate that this attentional deployment

strategy may not be sufficient to successfully downregulate affective

hyper-responsivity. As increased affective arousal was related to reduced

empathic ability, we hypothesize that own affective responses to social cues play

an important role in difficulties in understanding the feelings and intentions of

others. This knowledge may help in the identification of risk factors for

psychopathology and targets for treatment.

FAU - van Rijn, Sophie

AU - van Rijn S

AD - Clinical Child and Adolescent Studies, Leiden University, Leiden, The Netherlands

; Leiden Institute for Brain and Cognition, Leiden, The Netherlands.

FAU - Barendse, Marjolein

AU - Barendse M

AD - Clinical Child and Adolescent Studies, Leiden University, Leiden, The

Netherlands.

FAU - van Goozen, Stephanie

AU - van Goozen S

AD - Clinical Child and Adolescent Studies, Leiden University, Leiden, The Netherlands

; School of Psychology, Cardiff University, Cardiff, United Kingdom.

FAU - Swaab, Hanna

AU - Swaab H

AD - Clinical Child and Adolescent Studies, Leiden University, Leiden, The Netherlands

; Leiden Institute for Brain and Cognition, Leiden, The Netherlands.

LA - eng

PT - Journal Article

PT - Research Support, Non-U.S. Gov't

DEP - 20140108

TA - PLoS One

JT - PloS one

JID - 101285081

SB - IM

MH - Adult

MH - Attention/*physiology

MH - Emotions/*physiology

MH - Empathy/*physiology

MH - *Eye Movements

MH - *Galvanic Skin Response

MH - Humans

MH - Klinefelter Syndrome/*physiopathology/*psychology

MH - Male

MH - Self Report

MH - Visual Perception

PMC - PMC3885757

COIS- Competing Interests: The authors have declared that no competing interests exist.

EDAT- 2014/01/15 06:00

MHDA- 2014/09/11 06:00

CRDT- 2014/01/14 06:00

PHST- 2013/04/23 00:00 [received]

PHST- 2013/11/18 00:00 [accepted]

PHST- 2014/01/14 06:00 [entrez]

PHST- 2014/01/15 06:00 [pubmed]

PHST- 2014/09/11 06:00 [medline]

AID - PONE-D-13-17806 [pii]

AID - 10.1371/journal.pone.0084721 [doi]

PST - epublish

SO - PLoS One. 2014 Jan 8;9(1):e84721. doi: 10.1371/journal.pone.0084721. eCollection

2014.

PMID- 31593332

OWN - NLM

STAT- MEDLINE

DCOM- 20201210

LR - 20210110

IS - 1097-4679 (Electronic)

IS - 0021-9762 (Print)

IS - 0021-9762 (Linking)

VI - 76

IP - 1

DP - 2020 Jan

TI - Emotion regulation in adults with Klinefelter syndrome (47,XXY): Neurocognitive

underpinnings and associations with mental health problems.

PG - 228-238

LID - 10.1002/jclp.22871 [doi]

AB - OBJECTIVES: The aim of this study is to evaluate if language and executive

functioning deficits in individuals with the 47,XXY chromosomal pattern

contribute to emotion regulation problems and related symptoms of

psychopathology. METHODS: A group of 26 adult men with 47,XXY completed measures

of cognitive emotion regulation strategies, neurocognitive functioning, and

symptoms of psychopathology. RESULTS: Atypical emotion regulation strategies were

found in the XXY group, with increased expression of emotions (69%), avoiding

(65%), distraction seeking (54%), and passive coping (54%). More difficulties in

mental flexibility and attention regulation, and speeded responding were

associated with more pronounced emotion expression (emotional outbursts). Emotion

regulation problems were associated with symptoms of anxiety, depression, thought

problems, and hostility. CONCLUSION: This study has identified emotion regulation

as a potential target for treatment and intervention, with a specific focus on

executive functions in the management of emotions in individuals with 47,XXY.

CI - © 2019 The Authors. Journal of Clinical Psychology published by Wiley

Periodicals, Inc.

FAU - van Rijn, Sophie

AU - van Rijn S

AUID- ORCID: 0000-0002-9179-7515

AD - Clinical Neurodevelopmental Sciences, Leiden University, Leiden, The Netherlands.

AD - Leiden Institute for Brain and Cognition, Leiden, The Netherlands.

FAU - Swaab, Hanna

AU - Swaab H

AD - Clinical Neurodevelopmental Sciences, Leiden University, Leiden, The Netherlands.

AD - Leiden Institute for Brain and Cognition, Leiden, The Netherlands.

LA - eng

PT - Journal Article

PT - Research Support, Non-U.S. Gov't

DEP - 20191008

TA - J Clin Psychol

JT - Journal of clinical psychology

JID - 0217132

SB - IM

MH - Adult

MH - *Emotional Regulation

MH - Executive Function/physiology

MH - Female

MH - Humans

MH - Klinefelter Syndrome/*psychology

MH - Male

MH - *Mental Disorders

MH - Middle Aged

MH - Neuropsychological Tests

MH - Surveys and Questionnaires

PMC - PMC6916332

OTO - NOTNLM

OT - *47,XXY

OT - *Klinefelter syndrome

OT - *cognition

OT - *coping

OT - *emotion regulation

OT - *mental health

OT - *psychopathology

OT - *sex chromosome aneuploidy

EDAT- 2019/10/09 06:00

MHDA- 2020/12/15 06:00

CRDT- 2019/10/09 06:00

PHST- 2019/10/09 06:00 [pubmed]

PHST- 2020/12/15 06:00 [medline]

PHST- 2019/10/09 06:00 [entrez]

AID - JCLP22871 [pii]

AID - 10.1002/jclp.22871 [doi]

PST - ppublish

SO - J Clin Psychol. 2020 Jan;76(1):228-238. doi: 10.1002/jclp.22871. Epub 2019 Oct 8.

PMID- 20014371

OWN - NLM

STAT- MEDLINE

DCOM- 20100319

LR - 20211020

IS - 1940-5529 (Electronic)

IS - 1940-5510 (Print)

IS - 1940-5529 (Linking)

VI - 15

IP - 4

DP - 2009

TI - An extra X or Y chromosome: contrasting the cognitive and motor phenotypes in

childhood in boys with 47,XYY syndrome or 47,XXY Klinefelter syndrome.

PG - 309-17

LID - 10.1002/ddrr.85 [doi]

AB - OBJECTIVE: The goal of this study was to contrast the cognitive phenotypes in

boys with 47,XYY (XYY) karyotype and boys with 47,XXY karyotype [Klinefelter

syndrome, (KS)], who share an extra copy of the X-Y pseudoautosomal region but

differ in their dosage of strictly sex-linked genes. METHODS: Neuropsychological

evaluation of general cognitive ability, language, memory, attention,

visual-spatial abilities, visual-motor skills, and motor function. RESULTS: Study

cohort: 21 boys with 47,XYY and 93 boys with 47,XXY (KS), age 4-17 years, and 36

age-matched control boys. Both the XYY and KS groups performed less well, on

average, than the controls on tests of general cognitive ability, achievement,

language, verbal memory, some aspects of attention, and executive function, and

motor function. The boys with XYY on average had more severe and pervasive

language impairment, at both simple and complex levels, and the boys with KS on

average had greater motor impairment in gross motor function and coordination,

especially in running speed and agility. CONCLUSIONS: The results from these

large XYY and KS cohorts have important neurocognitive and educational

implications. From the neurocognitive standpoint, the presenting findings afford

an opportunity to gain insights into brain development in boys with XYY and those

with KS. From the educational standpoint, it is critical that boys with XYY or KS

receive appropriate educational interventions that target their specific learning

challenges. These findings also provide important information for counseling

clinicians and families about these disorders.

FAU - Ross, Judith L

AU - Ross JL

AD - Department of Pediatrics, Thomas Jefferson University, 1025 Walnut Street, Suite

726, Philadelphia, PA 19107, USA. judith.ross@mail.tju.edu

FAU - Zeger, Martha P D

AU - Zeger MP

FAU - Kushner, Harvey

AU - Kushner H

FAU - Zinn, Andrew R

AU - Zinn AR

FAU - Roeltgen, David P

AU - Roeltgen DP

LA - eng

GR - R01 NS050597/NS/NINDS NIH HHS/United States

GR - R01 NS050597-01A2/NS/NINDS NIH HHS/United States

GR - R01 NS050597-03S1/NS/NINDS NIH HHS/United States

GR - R01NS050597/NS/NINDS NIH HHS/United States

PT - Journal Article

PT - Research Support, N.I.H., Extramural

TA - Dev Disabil Res Rev

JT - Developmental disabilities research reviews

JID - 101319448

RN - 3XMK78S47O (Testosterone)

SB - IM

MH - Achievement

MH - Adolescent

MH - Child

MH - Child, Preschool

MH - Chromosomes, Human, X/*genetics

MH - Chromosomes, Human, Y/*genetics

MH - Cognition Disorders/diagnosis/*epidemiology

MH - Humans

MH - Karyotyping

MH - Klinefelter Syndrome/blood/*epidemiology/*genetics

MH - Male

MH - Motor Skills Disorders/diagnosis/*epidemiology

MH - Neuropsychological Tests

MH - *Phenotype

MH - Testosterone/blood/genetics

PMC - PMC2876236

MID - NIHMS201338

EDAT- 2009/12/17 06:00

MHDA- 2010/03/20 06:00

CRDT- 2009/12/17 06:00

PHST- 2009/12/17 06:00 [entrez]

PHST- 2009/12/17 06:00 [pubmed]

PHST- 2010/03/20 06:00 [medline]

AID - 10.1002/ddrr.85 [doi]

PST - ppublish

SO - Dev Disabil Res Rev. 2009;15(4):309-17. doi: 10.1002/ddrr.85.

PMID- 24439539

OWN - NLM

STAT- MEDLINE

DCOM- 20140418

LR - 20161209

IS - 2213-0276 (Electronic)

IS - 0755-4982 (Linking)

VI - 43

IP - 2

DP - 2014 Feb

TI - [Fertility in Klinefelter syndrome].

PG - 162-70

LID - S0755-4982(13)00865-8 [pii]

LID - 10.1016/j.lpm.2013.12.002 [doi]

AB - In Klinefelter syndrome with non-mosaic 47,XXY caryotype, a biological paternity

can be obtained by TEsticular Sperm Extraction and Intra-Cytoplasmic sperm

injection (TESE-ICSI). TESE is positive in about 50 % of the cases in published

series of non-mosaic 47,XXY Klinefelter syndrome. Age is the main prognosis

factor for TESE. Among patients seeking children, the percentage of positive TESE

is higher in younger men. Sperm cells are extracted from focal spermatogenesis.

They differenciate from spermatogonia which have corrected their chromosome

complement (46,XY). The risk of aneuploidy is similar in Klinefelter syndrome and

in non-obstructive azoospermia with normal caryotype. Among more than 100 born

children reported in the literature, all have a normal caryotype. Only one

foetus, within a triple pregnancy, had a 47,XXY caryotype. Whether the percentage

of positive TESE is better for adolescent than for adult Klinefelter patients

should be addressed by performing a TESE in adolescent (from 15 years old)

similarly to adult Klinefelter patients. TESE will be followed by

cryopreservation of extracted sperms. They will be used latter for ICSI when the

patient will seek children. This early TESE can be performed before the beginning

of the androgenic treatment, avoiding the potential deleterious feedback effect

of exogenous testosterone on the gonadotropin secretion and on the focal

spermatogenesis. Any androgenic treatment should be interrupted at least six

months before the TESE to avoid the feedback lowering effect on gonadotropin

secretion.

CI - Copyright © 2013 Elsevier Masson SAS. All rights reserved.

FAU - Lejeune, Hervé

AU - Lejeune H

AD - Hospices Civils de Lyon, hôpital femme-mère-enfant, service de médecine de la

reproduction, 69677 Bron cedex, France; Université Claude-Bernard Lyon 1, 69100

Villeurbanne, France; Inserm U 846, 69500 Bron, France. Electronic address:

herve.lejeune@chu-lyon.fr.

FAU - Brosse, Aurélie

AU - Brosse A

AD - Hospices Civils de Lyon, hôpital femme-mère-enfant, service de médecine de la

reproduction, 69677 Bron cedex, France.

CN - Groupe Fertipreserve

FAU - Plotton, Ingrid

AU - Plotton I

AD - Hospices Civils de Lyon, hôpital femme-mère-enfant, service de médecine de la

reproduction, 69677 Bron cedex, France; Université Claude-Bernard Lyon 1, 69100

Villeurbanne, France; Inserm U 846, 69500 Bron, France; Hospices Civils de Lyon,

centre de biologie et de pathologie Est, laboratoire d'hormonologie,

endocrinologie moléculaire et maladies rares, 69677 Bron cedex, France.

LA - fre

PT - English Abstract

PT - Journal Article

PT - Review

TT - Fertilité dans le syndrome de Klinefelter.

DEP - 20140116

PL - France

TA - Presse Med

JT - Presse medicale (Paris, France : 1983)

JID - 8302490

SB - IM

MH - Adolescent

MH - Adult

MH - Female

MH - Fertility/*physiology

MH - Humans

MH - Klinefelter Syndrome/*physiopathology/therapy

MH - Male

MH - Pregnancy

MH - Sperm Injections, Intracytoplasmic/*methods

MH - Treatment Outcome

EDAT- 2014/01/21 06:00

MHDA- 2014/04/20 06:00

CRDT- 2014/01/21 06:00

PHST- 2012/09/25 00:00 [received]

PHST- 2013/12/05 00:00 [revised]

PHST- 2013/12/05 00:00 [accepted]

PHST- 2014/01/21 06:00 [entrez]

PHST- 2014/01/21 06:00 [pubmed]

PHST- 2014/04/20 06:00 [medline]

AID - S0755-4982(13)00865-8 [pii]

AID - 10.1016/j.lpm.2013.12.002 [doi]

PST - ppublish

SO - Presse Med. 2014 Feb;43(2):162-70. doi: 10.1016/j.lpm.2013.12.002. Epub 2014 Jan

16.

PMID- 33627828

OWN - NLM

STAT- MEDLINE

DCOM- 20210707

LR - 20220210

IS - 1530-0366 (Electronic)

IS - 1098-3600 (Linking)

VI - 23

IP - 6

DP - 2021 Jun

TI - The effect of early hormonal treatment (EHT) on expressive and receptive language

capabilities in boys with 47,XXY (Klinefelter syndrome) during infancy and early

childhood.

PG - 1017-1022

LID - 10.1038/s41436-021-01098-w [doi]

AB - PURPOSE: 47,XXY is associated with variable neurodevelopmental outcomes including

deficits in expressive and receptive language development. Early hormonal

treatment (EHT) has been associated with mitigating some deficiencies in boys

with 47,XXY. This study investigates these language capabilities of 47,XXY boys

in the first five years of life and the associated effects of EHT on these

capabilities. METHODS: One hundred and seventy-five boys with 47,XXY between the

ages of 0 and 5 years, 11 months completed neurodevelopmental assessments

specific to age examining their expressive and receptive language capabilities.

Subjects were grouped by treatment (EHT and No-T) and differences were analyzed.

RESULTS: In the age groups of under 12 months, 24-35 months, 36-47 months, and

60-71 months, the EHT group scored significantly higher on expressive language

assessments than the No-T group (p = 0.09, p = 0.0002, p = 0.009, and p = 0.02,

respectively). In the age groups of under 12 months and 24-35 months, the EHT

group scored significantly better on the auditory comprehension domain of the

PLS-4/5 (p = 0.02 and p = 0.05, respectively) than the No-T group. CONCLUSION:

Study data suggest EHT may be essential in optimizing receptive and expressive

language development in 47,XXY boys during early childhood, which is critical in

fostering reading skills and later academic success.

FAU - Samango-Sprouse, Carole

AU - Samango-Sprouse C

AUID- ORCID: 0000-0001-9941-0568

AD - Department of Pediatrics, George Washington University, Washington, DC, USA.

cssprouse@email.gwu.edu.

AD - Department of Human and Molecular Genetics, Florida International University,

Miami, FL, USA. cssprouse@email.gwu.edu.

AD - Department of Research, The Focus Foundation, Davidsonville, MD, USA.

cssprouse@email.gwu.edu.

FAU - Brooks, Michaela Reiko

AU - Brooks MR

AD - Department of Research, The Focus Foundation, Davidsonville, MD, USA.

FAU - Lasutchinkow, Patricia

AU - Lasutchinkow P

AD - Department of Research, The Focus Foundation, Davidsonville, MD, USA.

FAU - Sadeghin, Teresa

AU - Sadeghin T

AD - Department of Research, The Focus Foundation, Davidsonville, MD, USA.

FAU - Powell, Sherida

AU - Powell S

AD - Department of Economics, George Washington University, Washington, DC, USA.

FAU - Hamzik, Mary Pat

AU - Hamzik MP

AD - Department of Research, The Focus Foundation, Davidsonville, MD, USA.

FAU - Song, Sophia

AU - Song S

AD - Department of Research, The Focus Foundation, Davidsonville, MD, USA.

FAU - Gropman, Andrea L

AU - Gropman AL

AD - Department of Neurology, George Washington University, Washington, DC, USA.

AD - Division of Neurogenetics and Developmental Pediatrics, Children's National

Health System, Washington, DC, USA.

LA - eng

PT - Journal Article

PT - Research Support, Non-U.S. Gov't

DEP - 20210224

PL - United States

TA - Genet Med

JT - Genetics in medicine : official journal of the American College of Medical

Genetics

JID - 9815831

SB - IM

MH - Child, Preschool

MH - Comprehension

MH - Humans

MH - Infant

MH - Infant, Newborn

MH - *Klinefelter Syndrome/drug therapy/genetics

MH - Language Development

MH - Male

EDAT- 2021/02/26 06:00

MHDA- 2021/07/08 06:00

CRDT- 2021/02/25 05:40

PHST- 2020/09/24 00:00 [received]

PHST- 2021/01/04 00:00 [accepted]

PHST- 2021/01/04 00:00 [revised]

PHST- 2021/02/26 06:00 [pubmed]

PHST- 2021/07/08 06:00 [medline]

PHST- 2021/02/25 05:40 [entrez]

AID - S1098-3600(21)05212-6 [pii]

AID - 10.1038/s41436-021-01098-w [doi]

PST - ppublish

SO - Genet Med. 2021 Jun;23(6):1017-1022. doi: 10.1038/s41436-021-01098-w. Epub 2021

Feb 24.

PMID- 29423966

OWN - NLM

STAT- MEDLINE

DCOM- 20190228

LR - 20200930

IS - 1552-4833 (Electronic)

IS - 1552-4825 (Linking)

VI - 176

IP - 4

DP - 2018 Apr

TI - International investigation of neurocognitive and behavioral phenotype in 47,XXY

(Klinefelter syndrome): Predicting individual differences.

PG - 877-885

LID - 10.1002/ajmg.a.38621 [doi]

AB - 47,XXY (KS) occurs in 1:650 male births, though less than 25% are ever

identified. We assessed stability of neurocognitive features across diverse

populations and quantified factors mediating outcome. Forty-four boys from the

Netherlands (NL) and 54 boys from the United States (US) participated. The

Wechsler Intelligence Scales assessed intellectual functioning; the ANT program

evaluated cognitive function; and the CBCL assessed behavioral functioning. ANOVA

was used for group comparisons. Hierarchical regressions assessed variance

explained by each independent variable: parental education, timing of diagnosis,

testosterone, age, and nationality. Parental education, timing of diagnosis, and

hormonal treatment all played an important role in neurocognitive performance.

The observed higher IQ and better attention regulation in the US group as

compared to the NL group was observed with decreased levels of behavioral

problems in the US group. Cognitive measures that were different between the NL

and US groups, i.e., attention regulation and IQ scores, were also significantly

influenced by external factors including timing of diagnosis, testosterone

treatment, and parental education. On the ANT, a cognitive phenotype of 47,XXY

was observed, with similar scores on 9 out of the 10 ANT subtests for the NL and

US groups. This study lays additional features to the foundation for an algorithm

linking external variables to outcome on various neurodevelopmental measures.

CI - © 2018 Wiley Periodicals, Inc.

FAU - Samango-Sprouse, Carole

AU - Samango-Sprouse C

AUID- ORCID: 0000-0001-9941-0568

AD - The Focus Foundation, Davidsonville, Maryland.

AD - George Washington University, Washington, District of Columbia.

AD - Florida International University, Miami, Florida.

FAU - Stapleton, Emily

AU - Stapleton E

AD - The Focus Foundation, Davidsonville, Maryland.

FAU - Chea, Selena

AU - Chea S

AD - The Focus Foundation, Davidsonville, Maryland.

FAU - Lawson, Patrick

AU - Lawson P

AD - The Focus Foundation, Davidsonville, Maryland.

FAU - Sadeghin, Teresa

AU - Sadeghin T

AD - The Focus Foundation, Davidsonville, Maryland.

FAU - Cappello, Chris

AU - Cappello C

AD - The Focus Foundation, Davidsonville, Maryland.

FAU - de Sonneville, Leo

AU - de Sonneville L

AD - Leiden University, Rapenburg, Leiden, Netherlands.

FAU - van Rijn, Sophie

AU - van Rijn S

AD - Leiden University, Rapenburg, Leiden, Netherlands.

LA - eng

PT - Journal Article

PT - Research Support, Non-U.S. Gov't

DEP - 20180209

PL - United States

TA - Am J Med Genet A

JT - American journal of medical genetics. Part A

JID - 101235741

RN - 3XMK78S47O (Testosterone)

SB - IM

MH - Abnormal Karyotype

MH - Adolescent

MH - *Behavior

MH - Child

MH - *Cognition

MH - Emotions

MH - Female

MH - *Genetic Association Studies

MH - Humans

MH - Intelligence

MH - Klinefelter Syndrome/diagnosis/drug therapy/*genetics/*psychology

MH - Male

MH - Netherlands

MH - Neuropsychological Tests

MH - *Phenotype

MH - Testosterone/pharmacology

MH - United States

OTO - NOTNLM

OT - *47,XXY

OT - *Klinefelter syndrome

OT - *XXY

OT - *sex chromosome

OT - *sex chromosome aneuploidy

EDAT- 2018/02/10 06:00

MHDA- 2019/03/01 06:00

CRDT- 2018/02/10 06:00

PHST- 2017/06/06 00:00 [received]

PHST- 2017/10/27 00:00 [revised]

PHST- 2018/01/04 00:00 [accepted]

PHST- 2018/02/10 06:00 [pubmed]

PHST- 2019/03/01 06:00 [medline]

PHST- 2018/02/10 06:00 [entrez]

AID - 10.1002/ajmg.a.38621 [doi]

PST - ppublish

SO - Am J Med Genet A. 2018 Apr;176(4):877-885. doi: 10.1002/ajmg.a.38621. Epub 2018

Feb 9.

PMID- 28346690

OWN - NLM

STAT- MEDLINE

DCOM- 20180326

LR - 20180326

IS - 1097-0223 (Electronic)

IS - 0197-3851 (Linking)

VI - 37

IP - 5

DP - 2017 May

TI - The benefits and limitations of cell-free DNA screening for 47, XXY (Klinefelter

syndrome).

PG - 497-501

LID - 10.1002/pd.5044 [doi]

AB - OBJECTIVE: The purpose of this paper is to provide an overview of the 47, XXY

syndrome, which is the most commonly occurring X and Y chromosomal variation.

This paper seeks to review what is currently known of noninvasive prenatal

testing (NIPT) and 47, XXY and investigate potential risks and benefits of

prenatal identification. METHOD: A literature review of NIPT and 47, XXY was

performed to identify limitations of current NIPT techniques. RESULTS: As NIPT

becomes an increasingly more routine procedure, prenatal findings of 47, XXY may

increase. Awareness of this disorder and appropriate genetic counseling is

necessary. CONCLUSION: X and Y chromosomal variations will be identified through

this screening, and the benefits and limitations to this finding need to be

thoughtfully considered. © 2017 John Wiley & Sons, Ltd.

CI - © 2017 John Wiley & Sons, Ltd.

FAU - Samango-Sprouse, Carole

AU - Samango-Sprouse C

AUID- ORCID: 0000-0001-9941-0568

AD - Department of Pediatrics, George Washington University, Washington, DC, USA.

AD - Department of Molecular Genetics, Florida International University, Miami, FL,

USA.

FAU - Keen, Colleen

AU - Keen C

AD - The Focus Foundation, Davidsonville, MD, USA.

FAU - Sadeghin, Teresa

AU - Sadeghin T

AD - The Focus Foundation, Davidsonville, MD, USA.

FAU - Gropman, Andrea

AU - Gropman A

AUID- ORCID: 0000-0002-2106-6776

AD - Department of Neurodevelopmental Disorders and Neurogenetics, Children's National

Medical Center, Washington, DC, USA.

LA - eng

PT - Journal Article

PT - Review

DEP - 20170417

PL - England

TA - Prenat Diagn

JT - Prenatal diagnosis

JID - 8106540

RN - 0 (Cell-Free Nucleic Acids)

SB - IM

MH - Cell-Free Nucleic Acids/*analysis/blood

MH - Female

MH - Genetic Testing/methods

MH - Humans

MH - Klinefelter Syndrome/*diagnosis/genetics

MH - Predictive Value of Tests

MH - Pregnancy

MH - Prenatal Diagnosis/*methods

MH - Risk Assessment

EDAT- 2017/03/28 06:00

MHDA- 2018/03/27 06:00

CRDT- 2017/03/28 06:00

PHST- 2016/11/07 00:00 [received]

PHST- 2017/02/24 00:00 [revised]

PHST- 2017/03/22 00:00 [accepted]

PHST- 2017/03/28 06:00 [pubmed]

PHST- 2018/03/27 06:00 [medline]

PHST- 2017/03/28 06:00 [entrez]

AID - 10.1002/pd.5044 [doi]

PST - ppublish

SO - Prenat Diagn. 2017 May;37(5):497-501. doi: 10.1002/pd.5044. Epub 2017 Apr 17.

PMID- 24139812

OWN - NLM

STAT- MEDLINE

DCOM- 20140718

LR - 20220310

IS - 1879-1379 (Electronic)

IS - 0022-3956 (Linking)

VI - 48

IP - 1

DP - 2014 Jan

TI - Klinefelter syndrome and risk of psychosis, autism and ADHD.

PG - 128-30

LID - S0022-3956(13)00309-9 [pii]

LID - 10.1016/j.jpsychires.2013.10.001 [doi]

AB - BACKGROUND: Schizophrenia, bipolar disorder, autism spectrum disorders and ADHD

might be overrepresented in Klinefelter syndrome, but previous investigations

have yielded inconclusive results. METHODS: We compared a national sample of 860

Klinefelter patients in Sweden with 86 000 matched population controls. To assess

the risks of schizophrenia, bipolar disorder, autism spectrum disorder and ADHD

in Klinefelter patients, we estimated odds ratios and 95% confidence intervals

using conditional logistic regressions. RESULTS: Klinefelter patients had almost

four times higher risks of schizophrenia, odds ratio (OR) = 3.6, 95% confidence

interval (CI) 2.0-6.7 and bipolar disorder (OR = 3.8, CI 1.8-7.6) and about six

times higher risk of autism spectrum disorder (OR = 6.2, CI 4.0-9.4) and ADHD (OR

= 5.6, CI 4.0-7.8). CONCLUSIONS: The risk of psychosis, autism and ADHD is

increased in Klinefelter patients. These findings indicate an X

chromosome-related factor in the etiology of the studied psychiatric disorders,

and may also have implications for treatment of patients with Klinefelter

syndrome.

CI - Copyright © 2013 Elsevier Ltd. All rights reserved.

FAU - Cederlöf, Martin

AU - Cederlöf M

AD - Department of Medical Epidemiology and Biostatistics, Karolinska Institutet, Box

281, SE-171 77 Stockholm, Sweden. Electronic address: Martin.Cederlof@ki.se.

FAU - Ohlsson Gotby, Agnes

AU - Ohlsson Gotby A

FAU - Larsson, Henrik

AU - Larsson H

FAU - Serlachius, Eva

AU - Serlachius E

FAU - Boman, Marcus

AU - Boman M

FAU - Långström, Niklas

AU - Långström N

FAU - Landén, Mikael

AU - Landén M

FAU - Lichtenstein, Paul

AU - Lichtenstein P

LA - eng

PT - Journal Article

DEP - 20131011

PL - England

TA - J Psychiatr Res

JT - Journal of psychiatric research

JID - 0376331

SB - IM

MH - Adolescent

MH - Attention Deficit Disorder with Hyperactivity/*epidemiology

MH - Autistic Disorder/*epidemiology

MH - Child

MH - Humans

MH - Klinefelter Syndrome/*epidemiology

MH - Male

MH - Psychotic Disorders/*epidemiology

MH - Sweden/epidemiology

MH - Young Adult

OTO - NOTNLM

OT - ADHD

OT - Autism spectrum disorder

OT - Bipolar disorder

OT - Epidemiology

OT - Klinefelter syndrome

OT - Schizophrenia

EDAT- 2013/10/22 06:00

MHDA- 2014/07/19 06:00

CRDT- 2013/10/22 06:00

PHST- 2013/04/12 00:00 [received]

PHST- 2013/09/10 00:00 [revised]

PHST- 2013/10/02 00:00 [accepted]

PHST- 2013/10/22 06:00 [entrez]

PHST- 2013/10/22 06:00 [pubmed]

PHST- 2014/07/19 06:00 [medline]

AID - S0022-3956(13)00309-9 [pii]

AID - 10.1016/j.jpsychires.2013.10.001 [doi]

PST - ppublish

SO - J Psychiatr Res. 2014 Jan;48(1):128-30. doi: 10.1016/j.jpsychires.2013.10.001.

Epub 2013 Oct 11.

PMID- 28647951

OWN - NLM

STAT- MEDLINE

DCOM- 20180226

LR - 20180226

IS - 1565-4753 (Print)

IS - 1565-4753 (Linking)

VI - 14

IP - Suppl 2

DP - 2017 Jun

TI - Complexities of Care in Klinefelter Syndrome: An APRN Perspective.

PG - 462-471

LID - 10.17458/per.vol14.2017.ctf.complexitiescareklinefelter [doi]

AB - 47,XXY (Klinefelter Syndrome) is associated with a spectrum of complex clinical

needs that are associated with variable physical, neurocognitive and psychosocial

aspects. For patients and families affected by this sex chromosome trisomy,

navigation of health care services is difficult due to lack of 47,XXY awareness

among many health care providers and little evidence to support endocrine and

additional treatment plans. While endocrine management of androgen deficiency has

been the mainstay of treatment for patients from puberty through adulthood,

testosterone replacement, alone, fails to mitigate many symptoms and issues.

Prior to the onset of puberty, boys with 47,XXY often do not receive

interdisciplinary evaluations and treatment. Since multiple health and ancillary

therapeutic services are required for the management of 47,XXY, patients and

families often experience disjointed and uncoordinated care. We discuss

complexities of caring for patients with 47,XXY and the benefit of integrating

advanced practice nursing and medical perspectives to improve care delivery.

CI - Copyright© of YS Medical Media ltd.

FAU - Close, Sharron

AU - Close S

AD - Emory University, Nell Hodgson Woodruff School of Nursing, Atlanta, Georgia, USA,

Department of Pediatric Genetics Emory University School of Medicine, USA.

FAU - Talboy, Amy

AU - Talboy A

AD - Department of Pediatric Genetics Emory University School of Medicine, USA.

FAU - Fennoy, Ilene

AU - Fennoy I

AD - Division of Pediatric Endocrinology Columbia University Medical Center, New York

NY, USA.

LA - eng

PT - Journal Article

PT - Review

PL - Israel

TA - Pediatr Endocrinol Rev

JT - Pediatric endocrinology reviews : PER

JID - 101202124

SB - IM

MH - *Advanced Practice Nursing/methods/standards

MH - Cognition/physiology

MH - Genetic Heterogeneity

MH - Humans

MH - Klinefelter Syndrome/diagnosis/*nursing/psychology/*therapy

MH - Mental Disorders/complications/epidemiology/therapy

MH - Phenotype

MH - *Quality of Health Care/standards

MH - Social Behavior Disorders/complications/epidemiology/therapy

OTO - NOTNLM

OT - Klinefelter Syndrome

OT - Sex Chromosome Variation

OT - XXY

EDAT- 2017/06/26 06:00

MHDA- 2018/02/27 06:00

CRDT- 2017/06/26 06:00

PHST- 2017/06/26 06:00 [entrez]

PHST- 2017/06/26 06:00 [pubmed]

PHST- 2018/02/27 06:00 [medline]

AID - 10.17458/per.vol14.2017.ctf.complexitiescareklinefelter [doi]

PST - ppublish

SO - Pediatr Endocrinol Rev. 2017 Jun;14(Suppl 2):462-471. doi:

10.17458/per.vol14.2017.ctf.complexitiescareklinefelter.

PMID- 21207006

OWN - NLM

STAT- MEDLINE

DCOM- 20110517

LR - 20211020

IS - 1433-0563 (Electronic)

IS - 0340-2592 (Linking)

VI - 50

IP - 1

DP - 2011 Jan

TI - [Fertility in patients with Klinefelter syndrome (47,XXY)].

PG - 26-32

LID - 10.1007/s00120-010-2443-0 [doi]

AB - In the past 10 years, our knowledge about fertility chances of patients with

Klinefelter syndrome (KS, 47,XXY) has changed considerably, especially when

regarding the possibility of IVF ICSI treatment (in vitro fertilisation,

intracytoplasmic sperm injection) with single testicular spermatozoa. Thus, it is

important to take this knowledge into consideration when counselling Klinefelter

patients.Germ cell degeneration in the testicles of Klinefelter patients due to

their additional X chromosome is an important phenomenon in this disease which is

not yet fully understood. When entering puberty, the testicular volume of KS

patients increases for a short time with rising testosterone and inhibin B levels

at the same time. These decrease, however, and FSH increases during puberty. This

seems to indicate a critical point in time when spermatogenetic function of the

testicles could still be existent. Thus, in early puberty there could possibly be

a time slot when spermatozoa could be detected in the ejaculate or-if not-at

least in the testicular tissue. These could be extracted by testicular sperm

extraction, cryopreserved and used for intracytoplasmic sperm injection therapy

later on. In the literature, a total of 133 births of children from Klinefelter

fathers have been reported. This early specific procedure could lead to a better

acceptance of their diagnosis and also offer the option of not being incurably

infertile.

FAU - Kliesch, S

AU - Kliesch S

AD - Klinische Andrologie, Centrum für Reproduktionsmedizin und Andrologie,

Universitätsklinikum Münster, Domagkstraße 11, 48149 Münster, Deutschland.

Sabine.Kliesch@ukmuenster.de

FAU - Zitzmann, M

AU - Zitzmann M

FAU - Behre, H M

AU - Behre HM

LA - ger

PT - English Abstract

PT - Journal Article

PT - Review

TT - Fertilität bei Patienten mit einem Klinefelter-Syndrom (47,XXY).

PL - Germany

TA - Urologe A

JT - Der Urologe. Ausg. A

JID - 1304110

SB - IM

MH - Azoospermia/diagnosis/*genetics/*therapy

MH - Genetic Testing/*trends

MH - Humans

MH - Infertility, Male/diagnosis/*genetics/*therapy

MH - Klinefelter Syndrome/diagnosis/*genetics/*therapy

MH - Male

MH - Reproductive Techniques, Assisted/trends

EDAT- 2011/01/06 06:00

MHDA- 2011/05/18 06:00

CRDT- 2011/01/06 06:00

PHST- 2011/01/06 06:00 [entrez]

PHST- 2011/01/06 06:00 [pubmed]

PHST- 2011/05/18 06:00 [medline]

AID - 10.1007/s00120-010-2443-0 [doi]

PST - ppublish

SO - Urologe A. 2011 Jan;50(1):26-32. doi: 10.1007/s00120-010-2443-0.

PMID- 27651829

OWN - NLM

STAT- PubMed-not-MEDLINE

DCOM- 20160921

LR - 20201001

IS - 1753-2000 (Print)

IS - 1753-2000 (Electronic)

IS - 1753-2000 (Linking)

VI - 10

DP - 2016

TI - A boy with conduct disorder (CD), attention deficit hyperactivity disorder

(ADHD), borderline intellectual disability, and 47,XXY syndrome in combination

with a 7q11.23 duplication, 11p15.5 deletion, and 20q13.33 deletion.

PG - 33

LID - 10.1186/s13034-016-0121-8 [doi]

LID - 33

AB - BACKGROUND: This is a case with multiple chromosomal aberrations which are likely

etiological for the observed psychiatric phenotype consisting of attention

deficit hyperactivity and conduct disorders. CASE PRESENTATION: We report on an

11 year-old boy, admitted to the pediatric hospital for behavioral difficulties

and a delayed neurodevelopmental trajectory. A cytogenetic analysis and

high-resolution microarray comparative genomic hybridization (CGH) analysis was

performed. The cytogenetic analysis revealed 47,XYY syndrome, while CGH analysis

revealed an additional duplication and two deletions. The 7q11.23 duplication is

associated with speech and language delay and behavioral symptoms, a 20q13.33

deletion is associated with autism and early onset schizophrenia and the 11p15.5

microdeletion is associated with developmental delay, autism, and epilepsy. The

patient underwent a psychiatric history, physical examination, laboratory

testing, and a detailed cognitive, psychiatric, and occupational therapy

evaluation which are reported here in detail. CONCLUSIONS: In the case of

psychiatric patients presenting with complex genetic aberrations and additional

psychosocial problems, traditional psychiatric and psychological approaches can

lead to significantly improved functioning. Genetic diagnostic testing can be

highly informative in the diagnostic process and may be applied to patients in

psychiatry in case of complex clinical presentations.

FAU - Kolaitis, Gerasimos

AU - Kolaitis G

AD - Department of Child and Adolescent Psychiatry/Psychology, Erasmus University

Medical Center, 3015 CN Rotterdam, The Netherlands.

FAU - Bouwkamp, Christian G

AU - Bouwkamp CG

AD - Department of Psychiatry and Department of Clinical Genetics, Erasmus University

Medical Center, 3015 CN Rotterdam, The Netherlands.

FAU - Papakonstantinou, Alexia

AU - Papakonstantinou A

AD - Department of Child Psychiatry, Medical School, National and Kapodistrian

University of Athens, "Aghia Sophia" Children's Hospital, 11527 Athens, Greece.

FAU - Otheiti, Ioanna

AU - Otheiti I

AD - Department of Child Psychiatry, Medical School, National and Kapodistrian

University of Athens, "Aghia Sophia" Children's Hospital, 11527 Athens, Greece.

FAU - Belivanaki, Maria

AU - Belivanaki M

AD - Department of Child Psychiatry, Medical School, National and Kapodistrian

University of Athens, "Aghia Sophia" Children's Hospital, 11527 Athens, Greece.

FAU - Haritaki, Styliani

AU - Haritaki S

AD - Department of Child Psychiatry, Medical School, National and Kapodistrian

University of Athens, "Aghia Sophia" Children's Hospital, 11527 Athens, Greece.

FAU - Korpa, Terpsihori

AU - Korpa T

AD - Department of Child Psychiatry, Medical School, National and Kapodistrian

University of Athens, "Aghia Sophia" Children's Hospital, 11527 Athens, Greece.

FAU - Albani, Zinovia

AU - Albani Z

AD - Department of Child Psychiatry, Medical School, National and Kapodistrian

University of Athens, "Aghia Sophia" Children's Hospital, 11527 Athens, Greece.

FAU - Terzioglou, Elena

AU - Terzioglou E

AD - Department of Child Psychiatry, Medical School, National and Kapodistrian

University of Athens, "Aghia Sophia" Children's Hospital, 11527 Athens, Greece.

FAU - Apostola, Polyxeni

AU - Apostola P

AD - Department of Child Psychiatry, Medical School, National and Kapodistrian

University of Athens, "Aghia Sophia" Children's Hospital, 11527 Athens, Greece.

FAU - Skamnaki, Aggeliki

AU - Skamnaki A

AD - Department of Child Psychiatry, Medical School, National and Kapodistrian

University of Athens, "Aghia Sophia" Children's Hospital, 11527 Athens, Greece.

FAU - Xaidara, Athena

AU - Xaidara A

AD - 1st Department of Pediatrics, Medical School, National and Kapodistrian

University of Athens, "Aghia Sophia" Children's Hospital, 11527 Athens, Greece.

FAU - Kosma, Konstantina

AU - Kosma K

AD - Department of Medical Genetics, Medical School, National and Kapodistrian

University of Athens, Athens, 11527 Greece.

FAU - Kitsiou-Tzeli, Sophia

AU - Kitsiou-Tzeli S

AD - Department of Medical Genetics, Medical School, National and Kapodistrian

University of Athens, Athens, 11527 Greece.

FAU - Tzetis, Maria

AU - Tzetis M

AD - Department of Medical Genetics, Medical School, National and Kapodistrian

University of Athens, Athens, 11527 Greece.

LA - eng

PT - Case Reports

DEP - 20160915

TA - Child Adolesc Psychiatry Ment Health

JT - Child and adolescent psychiatry and mental health

JID - 101297974

PMC - PMC5024517

OTO - NOTNLM

OT - 11p15.5 deletion

OT - 20q13.33 deletion syndrome

OT - 47,XYY syndrome

OT - 7q11.23 Williams–Beuren syndrome region micro duplication

OT - ADHD

OT - ASD

OT - Conduct disorder

EDAT- 2016/09/22 06:00

MHDA- 2016/09/22 06:01

CRDT- 2016/09/22 06:00

PHST- 2016/07/15 00:00 [received]

PHST- 2016/09/01 00:00 [accepted]

PHST- 2016/09/22 06:00 [entrez]

PHST- 2016/09/22 06:00 [pubmed]

PHST- 2016/09/22 06:01 [medline]

AID - 121 [pii]

AID - 10.1186/s13034-016-0121-8 [doi]

PST - epublish

SO - Child Adolesc Psychiatry Ment Health. 2016 Sep 15;10:33. doi:

10.1186/s13034-016-0121-8. eCollection 2016.

PMID- 29406610

OWN - NLM

STAT- MEDLINE

DCOM- 20181105

LR - 20181105

IS - 1601-183X (Electronic)

IS - 1601-183X (Linking)

VI - 17

IP - 6

DP - 2018 Jul

TI - The nature of social cognitive deficits in children and adults with Klinefelter

syndrome (47,XXY).

PG - e12465

LID - 10.1111/gbb.12465 [doi]

AB - About 1 in 650 boys are born with an extra X chromosome (47,XXY or Klinefelter

syndrome). 47,XXY is associated with vulnerabilities in socio-emotional

development. This study was designed to assess types of cognitive deficits in

individuals with 47,XXY that may contribute to social-emotional dysfunction, and

to evaluate the nature of such deficits at various levels: ranging from basic

visuospatial processing deficits, impairments in face recognition (FR), to

emotion expression impairments. A total of 70 boys and men with 47,XXY, aged 8 to

60 years old, participated in the study. The subtests feature identification, FR

and identification of facial emotions of the Amsterdam Neuropsychological Tasks

were used. Level of intellectual functioning was assessed with the child and

adult versions of the Wechsler Intelligence Scales. Reaction time data showed

that in the 47,XXY group, 17% had difficulties in visuospatial processing (no

social load), 26% had difficulties with FR (medium social load) and an even

higher number of 33% had difficulties with facial expressions of emotions

(high-social load). Information processing impairments increased as a function of

"social load" of the stimuli, independent of intellectual functioning. Taken

together, our data suggest that on average individuals with XXY may have more

difficulties in information processing when "social load" increases, suggesting a

specific difficulty in the higher-order labeling and interpretation of social

cues, which cannot be explained by more basic visuospatial perceptual skills.

Considering the increased risk for social cognitive impairments, routine

assessment of social cognitive functioning as part of neuropsychological

screening is warranted.

CI - © 2018 John Wiley & Sons Ltd and International Behavioural and Neural Genetics

Society.

FAU - van Rijn, S

AU - van Rijn S

AD - Clinical Child and Adolescent Studies, Leiden University, Leiden, The

Netherlands.

AD - Leiden Institute for Brain and Cognition, Leiden, The Netherlands.

FAU - de Sonneville, L

AU - de Sonneville L

AD - Clinical Child and Adolescent Studies, Leiden University, Leiden, The

Netherlands.

AD - Leiden Institute for Brain and Cognition, Leiden, The Netherlands.

FAU - Swaab, H

AU - Swaab H

AD - Clinical Child and Adolescent Studies, Leiden University, Leiden, The

Netherlands.

AD - Leiden Institute for Brain and Cognition, Leiden, The Netherlands.

LA - eng

PT - Journal Article

PT - Research Support, Non-U.S. Gov't

DEP - 20180322

PL - England

TA - Genes Brain Behav

JT - Genes, brain, and behavior

JID - 101129617

SB - IM

MH - Adolescent

MH - Adult

MH - Affective Symptoms/genetics

MH - Aged

MH - Child

MH - Cognition

MH - Cognition Disorders/genetics

MH - Cognitive Dysfunction/genetics

MH - Emotions/physiology

MH - Humans

MH - Intelligence Tests

MH - Klinefelter Syndrome/*genetics/*psychology

MH - Male

MH - Middle Aged

MH - Neuropsychological Tests

MH - Social Behavior

MH - *Social Skills

OTO - NOTNLM

OT - *Klinefelter syndrome

OT - *X chromosome

OT - *XXY

OT - *cognition

OT - *emotions

OT - *facial expressions

OT - *sex chromosome aneuploidies

OT - *sex chromosome variations

OT - *social cognition

OT - *visual processing

EDAT- 2018/02/07 06:00

MHDA- 2018/11/06 06:00

CRDT- 2018/02/07 06:00

PHST- 2017/07/27 00:00 [received]

PHST- 2018/01/09 00:00 [revised]

PHST- 2018/02/01 00:00 [accepted]

PHST- 2018/02/07 06:00 [pubmed]

PHST- 2018/11/06 06:00 [medline]

PHST- 2018/02/07 06:00 [entrez]

AID - 10.1111/gbb.12465 [doi]

PST - ppublish

SO - Genes Brain Behav. 2018 Jul;17(6):e12465. doi: 10.1111/gbb.12465. Epub 2018 Mar

22.

PMID- 20197378

OWN - NLM

STAT- MEDLINE

DCOM- 20100831

LR - 20100520

IS - 1460-2407 (Electronic)

IS - 1360-9947 (Linking)

VI - 16

IP - 6

DP - 2010 Jun

TI - Klinefelter's syndrome and psychoneurologic function.

PG - 425-33

LID - 10.1093/molehr/gaq018 [doi]

AB - Klinefelter's syndrome (KS) is due to the presence of one or more supernumerary X

chromosomes. Aneuploidy 47,XXY is the most common abnormality of sex chromosomes

in humans, with an incidence of 1/500 male live births. Only one-third of

subjects with KS is, however, diagnosed. The aim of this work is to present a

review of current literature about neurogenetic functions in KS, referring to

both clinical and therapeutics aspects. If it is well known that the majority of

subjects with 47,XXY karyotype have a normal intellectual level, the

identification of strengths and weaknesses of their intellectual functioning is

important for the purpose of planning early psycho-educational interventions.

Language difficulties are one of the more distinctive traits in cognitive

functioning of people with KS. It has also been suggested that the limitations in

communication markedly affect social adaptation and behavioral aspects, as well

as the development of personality. Moreover, difficulties in learning language

appear to be related to an altered functional lateralization; therefore, KS

subjects are a suitable model for studying genetic abnormalities of

lateralization. In this, perspective psychopathological risk is analyzed. Early

recognition of this aspect is needed to address the educational and therapeutic

perspectives for KS subjects.

FAU - Verri, Annapia

AU - Verri A

AD - Laboratorio di Psicologia Cognitivo-Comportamentale, Fondazione IRCCS Istituto

Neurologico Casimiro Mondino, Pavia, Italy. annapia.verri@mondino.it

FAU - Cremante, Anna

AU - Cremante A

FAU - Clerici, Federica

AU - Clerici F

FAU - Destefani, Valeria

AU - Destefani V

FAU - Radicioni, Antonio

AU - Radicioni A

LA - eng

PT - Journal Article

PT - Research Support, Non-U.S. Gov't

PT - Review

DEP - 20100302

PL - England

TA - Mol Hum Reprod

JT - Molecular human reproduction

JID - 9513710

SB - IM

MH - Animals

MH - Attention/physiology

MH - Humans

MH - Intelligence/physiology

MH - Klinefelter Syndrome/physiopathology/*psychology

MH - Language

MH - Male

MH - Mental Processes/*physiology

MH - Models, Biological

MH - Neuropsychological Tests

MH - Psychomotor Performance/physiology

RF - 65

EDAT- 2010/03/04 06:00

MHDA- 2010/09/02 06:00

CRDT- 2010/03/04 06:00

PHST- 2010/03/04 06:00 [entrez]

PHST- 2010/03/04 06:00 [pubmed]

PHST- 2010/09/02 06:00 [medline]

AID - gaq018 [pii]

AID - 10.1093/molehr/gaq018 [doi]

PST - ppublish

SO - Mol Hum Reprod. 2010 Jun;16(6):425-33. doi: 10.1093/molehr/gaq018. Epub 2010 Mar

2.

PMID- 32220052

OWN - NLM

STAT- MEDLINE

DCOM- 20210603

LR - 20210603

IS - 1552-4833 (Electronic)

IS - 1552-4825 (Linking)

VI - 182

IP - 8

DP - 2020 Aug

TI - Neurodevelopmental outcome of prenatally diagnosed boys with 47,XXY (Klinefelter

syndrome) and the potential influence of early hormonal therapy.

PG - 1881-1889

LID - 10.1002/ajmg.a.61561 [doi]

AB - This cross-sectional study examined the neurodevelopment of a large, prenatally

diagnosed population of boys with 47,XXY; investigated the potentially positive

effects of early hormonal therapy (EHT) on language, cognition, and motor in this

population; and identified novel at risk biomarkers associated with 47,XXY.

Two-hundred and seventy two evaluations were collected from 148 prenatally

diagnosed boys with 47,XXY between 0 and 36 months and separated into one of

three groups, depending on visit age: Y1 (0-12 months; n = 100), Y2

(13-24 months; n = 90), and Y3 (25-36 months; n = 82). Those who received EHT

(administered by 12 months) were further separated (Y1, n = 37; Y2, n = 34; Y3, n

= 30). Neurodevelopmental evaluations consisted of Preschool Language Scales,

Early Language Milestone Scale, and Bayley Scales of Infant and Toddler

Development and evaluated the effect of EHT on auditory comprehension, expressive

communication, receptive language, cognition, and motor. EHT was found to be

associated with a positive effect within the first year of life in these domains,

as well as in the second and third year of life. Additionally, three novel

at-risk biomarkers were identified in this cohort: feeding difficulties in

infancy, positional torticollis, and the need for orthotics. The positive effects

of EHT observed in language, cognition, and motor at variable stages within the

first 3 years of life provide additional evidence into the possible efficacy of

early biological treatment for boys with 47,XXY to address the neurodevelopmental

dysfunction.

CI - © 2020 Wiley Periodicals, Inc.

FAU - Samango-Sprouse, Carole A

AU - Samango-Sprouse CA

AUID- ORCID: 0000-0001-9941-0568

AD - Department of Pediatrics, George Washington University, Washington, District of

Columbia, USA.

AD - Department of Human and Molecular Genetics, Florida International University,

Miami, Florida, USA.

AD - Department of Research, The Focus Foundation, Davidsonville, Maryland, USA.

FAU - Tran, Selena L

AU - Tran SL

AD - Department of Research, The Focus Foundation, Davidsonville, Maryland, USA.

FAU - Lasutschinkow, Patricia C

AU - Lasutschinkow PC

AD - Department of Research, The Focus Foundation, Davidsonville, Maryland, USA.

FAU - Sadeghin, Teresa

AU - Sadeghin T

AD - Department of Research, The Focus Foundation, Davidsonville, Maryland, USA.

FAU - Powell, Sherida

AU - Powell S

AD - Department of Pediatrics, George Washington University, Washington, District of

Columbia, USA.

AD - Department of Economics, George Washington University, Washington, District of

Columbia, USA.

FAU - Mitchell, Francie L

AU - Mitchell FL

AD - Department of Research, The Focus Foundation, Davidsonville, Maryland, USA.

FAU - Gropman, Andrea

AU - Gropman A

AD - Division of Neurogenetics and Developmental Pediatrics, Children's National

Health System, Washington, District of Columbia, USA.

AD - Department of Neurology, George Washington University, Washington, District of

Columbia, USA.

LA - eng

PT - Journal Article

DEP - 20200327

PL - United States

TA - Am J Med Genet A

JT - American journal of medical genetics. Part A

JID - 101235741

RN - 0 (Biomarkers)

RN - 0 (Hormones)

RN - 47, XYY syndrome

SB - IM

MH - Biomarkers/blood

MH - Child, Preschool

MH - Cognition/drug effects/physiology

MH - Female

MH - Hormones/*administration & dosage/adverse effects

MH - Humans

MH - Infant

MH - Klinefelter Syndrome/diagnosis/*drug therapy/genetics/physiopathology

MH - Male

MH - Neurodevelopmental Disorders/diagnosis/epidemiology/genetics/physiopathology

MH - Pregnancy

MH - *Prenatal Diagnosis

MH - Risk Factors

MH - Sex Chromosome Disorders/diagnosis/*drug therapy/genetics/physiopathology

MH - XYY Karyotype/diagnosis/*drug therapy/genetics/physiopathology

OTO - NOTNLM

OT - *47,XXY

OT - *Klinefelter syndrome

OT - *early hormonal therapy

OT - *neurodevelopment

OT - *prenatally diagnosed

EDAT- 2020/03/29 06:00

MHDA- 2021/06/04 06:00

CRDT- 2020/03/29 06:00

PHST- 2019/12/27 00:00 [received]

PHST- 2020/03/09 00:00 [accepted]

PHST- 2020/03/29 06:00 [pubmed]

PHST- 2021/06/04 06:00 [medline]

PHST- 2020/03/29 06:00 [entrez]

AID - 10.1002/ajmg.a.61561 [doi]

PST - ppublish

SO - Am J Med Genet A. 2020 Aug;182(8):1881-1889. doi: 10.1002/ajmg.a.61561. Epub 2020

Mar 27.

PMID- 33726973

OWN - NLM

STAT- MEDLINE

DCOM- 20210709

LR - 20210709

IS - 1873-4898 (Electronic)

IS - 1477-5131 (Linking)

VI - 17

IP - 3

DP - 2021 Jun

TI - Androgenization in Klinefelter syndrome: Clinical spectrum from infancy through

young adulthood.

PG - 346-352

LID - S1477-5131(21)00103-0 [pii]

LID - 10.1016/j.jpurol.2021.02.021 [doi]

AB - Klinefelter syndrome (KS) is an uncommon chromosomal disorder in males that has a

variable clinical appearance. Classic KS involves an extra X chromosome, (47,

XXY), although other variations may exist, including a milder mosaic form as well

as multiple extra sex chromosomes with more dramatic phenotypes. KS is

underdiagnosed, especially pre-pubertally, owing to a paucity of concrete

clinical signs; however, diagnostic rates increase during and after puberty, as

the consequences of hypergonadotropic hypogonadism begin to manifest. Testicular

failure causing decreased circulating testosterone (T) and germ cell depletion, a

hallmark feature in KS, commonly begins shortly after the onset of puberty and

leads to the most commonly recognized KS traits: small testes, azoospermia,

gynecomastia, decreased facial and pubic hair. While many KS men maintain

adequate T levels leading up to young adulthood, some may have lower T levels at

an earlier age leading to varied levels of androgenization and clinical KS

features. At certain critical time points, absent or decreased T may alter the

development of normal male reproductive organs, external genitalia, development

of secondary sexual characteristics and spermatogenesis. Testicular failure in

utero may lead to ambiguous genitalia, cryptorchidism and/or hypospadias, all of

which depend on fetal T production. In the neonatal period and childhood,

decreased T levels during the mini-puberty of infancy may negatively impact germ

cell differentiation and male neuropsychological development. Finally, decreased

T during pubertal and young adulthood can lead to decreased virilization during

puberty, eunuchoid skeleton and decreased spermatogenesis. Depending on the

timing of the testicular failure, a reproductive window of sperm production may

exist to achieve paternity for KS men. The presence or absence of clinical

characteristics reflecting decreased androgenization provides an insight to the

relative testicular function during these developmental time points for those

with KS and contributes to variability within the syndrome.

CI - Copyright © 2021 Journal of Pediatric Urology Company. Published by Elsevier Ltd.

All rights reserved.

FAU - Nassau, Daniel E

AU - Nassau DE

AD - Division of Pediatric Urology, Nicklaus Children's Hospital, Miami, FL, USA;

Department of Urology, University of Miami Miller School of Medicine, Miami, FL,

USA. Electronic address: daniel.nassau@nicklaushealth.org.

FAU - Best, Jordan C

AU - Best JC

AD - Department of Urology, University of Miami Miller School of Medicine, Miami, FL,

USA.

FAU - Cohen, Jordan

AU - Cohen J

AD - Department of Urology, University of Miami Miller School of Medicine, Miami, FL,

USA.

FAU - Gonzalez, Daniel C

AU - Gonzalez DC

AD - Department of Urology, University of Miami Miller School of Medicine, Miami, FL,

USA.

FAU - Alam, Alireza

AU - Alam A

AD - Division of Pediatric Urology, Nicklaus Children's Hospital, Miami, FL, USA.

FAU - Ramasamy, Ranjith

AU - Ramasamy R

AD - Department of Urology, University of Miami Miller School of Medicine, Miami, FL,

USA.

LA - eng

PT - Journal Article

PT - Review

DEP - 20210224

PL - England

TA - J Pediatr Urol

JT - Journal of pediatric urology

JID - 101233150

SB - IM

MH - Adult

MH - *Azoospermia

MH - Child

MH - *Cryptorchidism

MH - Female

MH - Humans

MH - Infant

MH - Infant, Newborn

MH - *Klinefelter Syndrome/complications/diagnosis

MH - Male

MH - Spermatogenesis

MH - Testis

MH - Virilism

MH - Young Adult

OTO - NOTNLM

OT - *Androgenization

OT - *Hypergonadotrophic hypogonadism

OT - *Klinefelter syndrome

OT - *Testosterone

COIS- Conflicts of interest statement The authors have no relevant conflicts of

interests to disclose.

EDAT- 2021/03/18 06:00

MHDA- 2021/07/10 06:00

CRDT- 2021/03/17 06:02

PHST- 2020/11/05 00:00 [received]

PHST- 2021/02/02 00:00 [revised]

PHST- 2021/02/19 00:00 [accepted]

PHST- 2021/03/18 06:00 [pubmed]

PHST- 2021/07/10 06:00 [medline]

PHST- 2021/03/17 06:02 [entrez]

AID - S1477-5131(21)00103-0 [pii]

AID - 10.1016/j.jpurol.2021.02.021 [doi]

PST - ppublish

SO - J Pediatr Urol. 2021 Jun;17(3):346-352. doi: 10.1016/j.jpurol.2021.02.021. Epub

2021 Feb 24.

PMID- 20308053

OWN - NLM

STAT- MEDLINE

DCOM- 20100831

LR - 20100520

IS - 1460-2407 (Electronic)

IS - 1360-9947 (Linking)

VI - 16

IP - 6

DP - 2010 Jun

TI - Animal models for Klinefelter's syndrome and their relevance for the clinic.

PG - 375-85

LID - 10.1093/molehr/gaq024 [doi]

AB - In mammals, the contribution of the Y chromosome is paramount for male sexual

determination; however, the presence of a single functional X chromosome is also

of importance. In contrast to females where X inactivation is seen; the X

chromosome of the male stays active. When, due to meiotic non-disjunction events,

males are born with a supernumerary X chromosome, the resulting 47, XXY karyotype

is referred to as Klinefelter's syndrome. This frequent genetic condition is most

commonly associated with infertility, hypogonadism, gynecomastia and cognitive

impairments. The condition has also been associated with a reduced life

expectancy, insulin resistance, dyslipidemia, increased body fat mass and reduced

bone mineral content. In a variety of species, male animals with karyotypes

resembling Klinefelter's syndrome arise and develop a subset of features similar

to those seen in humans. The availability of these animals is driving efforts to

experimentally address the pathophysiology of the condition. To date, two models,

41, XXY and 41, XX(Y)* (mutated Y chromosome) male mice, have been established

which resemble aspects of the pathophysiology of Klinefelter's syndrome.

Experiments performed in these models confirm that the presence of a

supernumerary X chromosome causes germ cell loss, cognitive deficits, Leydig cell

hyperplasia, and that their Sertoli cells are capable of supporting germ cells of

normal karyotype. This review summarizes the generation and characterization of

the animal models for Klinefelter's syndrome and suggests experimental strategies

to improve our understanding of the mechanisms underlying the pathophysiology of

Klinefelter's syndrome.

FAU - Wistuba, Joachim

AU - Wistuba J

AD - Institute of Reproductive and Regenerative Biology, Centre of Reproductive

Medicine and Andrology, University Clinics, Domagkstrasse 11, 48149 Münster,

Germany. joachim.wistuba@ukmuenster.de

LA - eng

PT - Journal Article

PT - Research Support, Non-U.S. Gov't

PT - Review

DEP - 20100321

PL - England

TA - Mol Hum Reprod

JT - Molecular human reproduction

JID - 9513710

SB - IM

MH - Animals

MH - Biomedical Research/methods/trends

MH - Chromosome Aberrations

MH - Chromosomes, Human, X/genetics

MH - *Disease Models, Animal

MH - Female

MH - Humans

MH - Klinefelter Syndrome/genetics/*pathology/*therapy

MH - Male

MH - Mammals/genetics

MH - Mice

MH - Models, Biological

RF - 70

EDAT- 2010/03/24 06:00

MHDA- 2010/09/02 06:00

CRDT- 2010/03/24 06:00

PHST- 2010/03/24 06:00 [entrez]

PHST- 2010/03/24 06:00 [pubmed]

PHST- 2010/09/02 06:00 [medline]

AID - gaq024 [pii]

AID - 10.1093/molehr/gaq024 [doi]

PST - ppublish

SO - Mol Hum Reprod. 2010 Jun;16(6):375-85. doi: 10.1093/molehr/gaq024. Epub 2010 Mar

21.

PMID- 32415901

OWN - NLM

STAT- MEDLINE

DCOM- 20210505

LR - 20210505

IS - 1552-4876 (Electronic)

IS - 1552-4868 (Linking)

VI - 184

IP - 2

DP - 2020 Jun

TI - Gonadal dysfunction and beyond: Clinical challenges in children, adolescents, and

adults with 47,XXY Klinefelter syndrome.

PG - 302-312

LID - 10.1002/ajmg.c.31786 [doi]

AB - Klinefelter syndrome (KS) is the most frequent sex chromosomal aneuploidy. The

karyotype 47,XXY originates from either paternal or maternal meiotic

nondisjunction during gametogenesis. KS males are very likely to exhibit marked

gonadal dysfunctions, presenting both in severely attenuated spermatogenesis as

well as hypergonadotropic hypogonadism. In addition, neurocognitive and

psychosocial impairments, as well as cardiovascular, metabolic and bone disorders

are often found in KS and might explain for an increased morbidity/mortality. All

conditions in KS are likely to be induced by both gene overdosage effects

resulting from supernumerary X-chromosomal genes as well as testosterone

deficiency. Notwithstanding, the clinical features are highly variable between KS

men. Symptoms can become obvious at infancy, childhood, or adolescence. However,

the majority of KS subjects is diagnosed during adulthood. KS adolescents require

specific attention regarding pubertal development, in order to exploit their

remaining fertility potential and allow for timely and tailored testosterone

replacement. The chances for sperm retrieval might decline with age and could be

hampered by testosterone replacement; therefore, cryostorage of spermatozoa is an

option during adolescence, before the decompensation of endocrine and exocrine

testicular functions becomes more overt. Sperm from semen or surgically

retrieved, in combination with intracytoplasmic sperm injection enables KS males

to become biological fathers of healthy children. The aim of this article is to

present the current knowledge on KS, to guide clinical care and to highlight

research needs.

CI - © 2020 The Authors. American Journal of Medical Genetics Part C: Seminars in

Medical Genetics published by Wiley Periodicals, LLC.

FAU - Zitzmann, Michael

AU - Zitzmann M

AUID- ORCID: 0000-0003-3629-7160

AD - Center for Reproductive Medicine and Andrology/Clinical Andrology, University

Clinics Muenster, Muenster, Germany.

FAU - Rohayem, Julia

AU - Rohayem J

AD - Center for Reproductive Medicine and Andrology/Clinical Andrology, University

Clinics Muenster, Muenster, Germany.

LA - eng

PT - Journal Article

PT - Review

DEP - 20200516

PL - United States

TA - Am J Med Genet C Semin Med Genet

JT - American journal of medical genetics. Part C, Seminars in medical genetics

JID - 101235745

RN - 47, XYY syndrome

SB - IM

MH - Adolescent

MH - Adult

MH - Child

MH - Child, Preschool

MH - Chromosomes, Human, X/*genetics

MH - Gonadal Disorders/genetics/pathology/*therapy

MH - Gonads/growth & development/pathology

MH - Humans

MH - Klinefelter Syndrome/*genetics/pathology

MH - Male

MH - Sex Chromosome Disorders/genetics/pathology/*therapy

MH - XYY Karyotype/genetics/pathology

MH - Young Adult

OTO - NOTNLM

OT - *47,XXY

OT - *Klinefelter syndrome

OT - *children with Klinefelter syndrome

OT - *fertility in Klinefelter syndrome

OT - *hypogonadism in Klinefelter syndrome

EDAT- 2020/05/18 06:00

MHDA- 2021/05/06 06:00

CRDT- 2020/05/17 06:00

PHST- 2020/04/07 00:00 [received]

PHST- 2020/04/09 00:00 [revised]

PHST- 2020/04/13 00:00 [accepted]

PHST- 2020/05/18 06:00 [pubmed]

PHST- 2021/05/06 06:00 [medline]

PHST- 2020/05/17 06:00 [entrez]

AID - 10.1002/ajmg.c.31786 [doi]

PST - ppublish

SO - Am J Med Genet C Semin Med Genet. 2020 Jun;184(2):302-312. doi:

10.1002/ajmg.c.31786. Epub 2020 May 16.

PMID- 23345262

OWN - NLM

STAT- MEDLINE

DCOM- 20130520

LR - 20130916

IS - 1552-4876 (Electronic)

IS - 1552-4868 (Linking)

VI - 163C

IP - 1

DP - 2013 Feb 15

TI - 47,XXY Klinefelter syndrome: clinical characteristics and age-specific

recommendations for medical management.

PG - 55-63

LID - 10.1002/ajmg.c.31349 [doi]

AB - 47,XXY (Klinefelter syndrome) is the most frequent sex chromosomal disorder and

affects approximately one in 660 newborn boys. The syndrome is characterized by

varying degrees of cognitive, social, behavioral, and learning difficulties and

in adulthood additionally primary testicular failure with small testes,

hypergonadotropic hypogonadism, tall stature, and eunuchoid body proportions. The

phenotype is variable ranging from "near-normal" to a significantly affected

individual. In addition, newborns with Klinefelter syndrome generally present

with a normal male phenotype and the only consistent clinical finding in KS is

small testes, that are most often not identified until after puberty. Decreased

awareness of this syndrome among health professionals and a general perception

that all patients with 47,XXY exhibit the classic textbook phenotype results in a

highly under-diagnosed condition with up to 75% of the patients left undetected.

Typically, diagnosis is delayed with the majority of patients identified during

fertility workup in adulthood, and only 10% of patients diagnosed prior to

puberty. Early detection of this syndrome is recommended in order to offer

treatment and intervention at the appropriate ages and stages of development for

the purpose of preventing osteopenia/osteoporosis, metabolic syndrome, and other

medical conditions related to hypogonadism and to the XXY as well as minimizing

potential learning and psychosocial problems. The aim of this review is to

present the clinical aspects of XXY and the age-specific recommendations for

medical management. © 2013 Wiley Periodicals, Inc.

CI - Copyright © 2013 Wiley Periodicals, Inc.

FAU - Aksglaede, Lise

AU - Aksglaede L

AD - Department of Growth and Reproduction GR, Rigshospitalet, Blegdamsvej, Copenhagen

Ø, Denmark. lise.aksglaede@rh.regionh.dk

FAU - Link, Katarina

AU - Link K

FAU - Giwercman, Aleksander

AU - Giwercman A

FAU - Jørgensen, Niels

AU - Jørgensen N

FAU - Skakkebaek, Niels E

AU - Skakkebaek NE

FAU - Juul, Anders

AU - Juul A

LA - eng

PT - Journal Article

PT - Research Support, Non-U.S. Gov't

DEP - 20130123

PL - United States

TA - Am J Med Genet C Semin Med Genet

JT - American journal of medical genetics. Part C, Seminars in medical genetics

JID - 101235745

SB - IM

MH - Aging/*genetics

MH - Humans

MH - Klinefelter Syndrome/*genetics/*physiopathology/*therapy

MH - Male

MH - Phenotype

MH - Sex Chromosome Disorders of Sex Development/diagnosis/genetics

EDAT- 2013/01/25 06:00

MHDA- 2013/05/22 06:00

CRDT- 2013/01/25 06:00

PHST- 2013/01/25 06:00 [entrez]

PHST- 2013/01/25 06:00 [pubmed]

PHST- 2013/05/22 06:00 [medline]

AID - 10.1002/ajmg.c.31349 [doi]

PST - ppublish

SO - Am J Med Genet C Semin Med Genet. 2013 Feb 15;163C(1):55-63. doi:

10.1002/ajmg.c.31349. Epub 2013 Jan 23.

PMID- 29512178

OWN - NLM

STAT- MEDLINE

DCOM- 20181018

LR - 20181018

IS - 1439-0272 (Electronic)

IS - 0303-4569 (Linking)

VI - 50

IP - 5

DP - 2018 Jun

TI - Klinefelter syndrome and fertility-Impact of X-chromosomal inheritance on

spermatogenesis.

PG - e13004

LID - 10.1111/and.13004 [doi]

AB - With the use of testicular sperm extraction (TESE), spermatozoa can be retrieved

in about 30%-50% of men with Klinefelter syndrome (KS). The reason for the

absence or presence of spermatozoa in half of the men with KS remains unknown.

Therefore, the search for an objective marker for a positive prediction in

finding spermatozoa is of significant clinical value to avoid unnecessary

testicular biopsies in males with (mostly) low testicular volume and impaired

testosterone. The objective of this study was to determine whether paternal or

maternal inheritance of the additional X-chromosome can predict the absence or

presence of spermatogenesis in men with KS. Men with KS who have had a testicular

biopsy for diagnostic fertility workup TESE were eligible for inclusion. Buccal

swabs from nine KS patients and parents (trios) were taken to compare

X-chromosomal inheritance to determine the parental origin of both X-chromosomes

in the males with KS. Spermatozoa were found in TESE biopsies 8 of 35 (23%)

patients after performing a unilateral or bilateral TESE. Different levels of

spermatogenesis (from the only presence of spermatogonia, up to maturation arrest

or hypospermatogenesis) appeared to be present in 19 of 35 (54%) men, meaning

that the presence of spermatogenesis not always yields mature spermatozoa. From

the nine KS-trios that were genetically analysed for X-chromosomal inheritance

origin, no evidence of a correlation between the maternal or paternal origin of

the additional X-chromosome and the presence of spermatogenesis was found. In

conclusion, the maternal or paternal origin of the additional X-chromosome in men

with KS does not predict the presence or absence of spermatogenesis.

CI - © 2018 The Authors Andrologia Published by Blackwell Verlag GmbH.

FAU - Franik, S

AU - Franik S

AUID- ORCID: 0000-0002-3434-1765

AD - Department of Obstetrics and Gynaecology, Radboudumc Nijmegen, Nijmegen, The

Netherlands.

AD - Department of Obstetrics and Gynaecology, University Hospital Münster, Münster,

Germany.

FAU - Smeets, D

AU - Smeets D

AD - Department of Human Genetics, Radboudumc Nijmegen, Nijmegen, The Netherlands.

FAU - van de Zande, G

AU - van de Zande G

AD - Department of Human Genetics, Radboudumc Nijmegen, Nijmegen, The Netherlands.

FAU - Gomes, I

AU - Gomes I

AD - Department of Human Genetics, Radboudumc Nijmegen, Nijmegen, The Netherlands.

FAU - D'Hauwers, K

AU - D'Hauwers K

AD - Department of Urology, Radboudumc Nijmegen, Nijmegen, The Netherlands.

FAU - Braat, D D M

AU - Braat DDM

AD - Department of Obstetrics and Gynaecology, Radboudumc Nijmegen, Nijmegen, The

Netherlands.

FAU - Fleischer, K

AU - Fleischer K

AD - Department of Obstetrics and Gynaecology, Radboudumc Nijmegen, Nijmegen, The

Netherlands.

FAU - Ramos, L

AU - Ramos L

AD - Department of Obstetrics and Gynaecology, Radboudumc Nijmegen, Nijmegen, The

Netherlands.

LA - eng

PT - Journal Article

DEP - 20180307

PL - Germany

TA - Andrologia

JT - Andrologia

JID - 0423506

RN - 3XMK78S47O (Testosterone)

RN - 57285-09-3 (Inhibins)

RN - 9002-67-9 (Luteinizing Hormone)

RN - 9002-68-0 (Follicle Stimulating Hormone)

SB - IM

MH - Adult

MH - Biopsy

MH - Fertility/*genetics

MH - Follicle Stimulating Hormone/blood

MH - Humans

MH - Inhibins/blood

MH - Klinefelter Syndrome/blood/genetics/*pathology

MH - Luteinizing Hormone/blood

MH - Male

MH - Sperm Retrieval

MH - Spermatogenesis/*genetics

MH - Spermatozoa/*pathology

MH - Testis/*pathology

MH - Testosterone/blood

OTO - NOTNLM

OT - TESE

OT - 47,XXY

OT - Klinefelter syndrome

OT - andrology

OT - genetics

EDAT- 2018/03/08 06:00

MHDA- 2018/10/20 06:00

CRDT- 2018/03/08 06:00

PHST- 2018/02/06 00:00 [accepted]

PHST- 2018/03/08 06:00 [pubmed]

PHST- 2018/10/20 06:00 [medline]

PHST- 2018/03/08 06:00 [entrez]

AID - 10.1111/and.13004 [doi]

PST - ppublish

SO - Andrologia. 2018 Jun;50(5):e13004. doi: 10.1111/and.13004. Epub 2018 Mar 7.

PMID- 34375016

OWN - NLM

STAT- MEDLINE

DCOM- 20211103

LR - 20211103

IS - 1439-0272 (Electronic)

IS - 0303-4569 (Linking)

VI - 53

IP - 11

DP - 2021 Dec

TI - A rare variant Klinefelter syndrome seen 40 years later: 47,X,del(Xq24),Y.

PG - e14213

LID - 10.1111/and.14213 [doi]

AB - Patients with Klinefelter syndrome (KS) show a typically 47,XXY karyotype;

however, some variations have been observed, including 47,XX,der(Y),

46,XY/47,XXY, 48,XXXY, 48,XXYY, and mosaicism or structural sex chromosome

abnormalities in some patients. In the literature, a rare KS variant,

47,X,del(Xq),Y karyotype, was reported in only a few cases prior to 1981. A

40-year-old man (IV-3) was referred to our department due to infertility. His

phenotype did not differ from the classic KS phenotype. He had two siblings

(1-male; 1-female). His brother (IV-5) had mental retardation and died one year

earlier at age 32. Additionally, his sister (IV-2) also had a history of

infertility due to her husband's azoospermia. His mother had a history of 12

miscarriages. Karyotype analysis revealed the 47,X,del(Xq24),Y karyotype, and no

deletions were seen in the AZF and SRY regions. We thought this chromosomal

abnormality in the patient might have resulted from X-autosome translocation in

one of his parents since his mother had recurrent pregnancy loss and his sibling

had mental retardation. However, we could not confirm it due to his parents were

not alive. This study shows the first case of a long-arm X-chromosome deletion

after a long period and reviews current knowledge concerning variant KS (deletion

Xq).

CI - © 2021 Wiley-VCH GmbH.

FAU - Özkent, Mehmet Serkan

AU - Özkent MS

AUID- ORCID: 0000-0002-6613-0671

AD - Department of Urology, Konya City Hospital, Konya, Turkey.

FAU - Balasar, Özgür

AU - Balasar Ö

AD - Department of Medical Genetics, Konya City Hospital, Konya, Turkey.

LA - eng

PT - Case Reports

DEP - 20210810

PL - Germany

TA - Andrologia

JT - Andrologia

JID - 0423506

SB - IM

MH - Adult

MH - *Azoospermia

MH - Female

MH - Humans

MH - Karyotyping

MH - *Klinefelter Syndrome/genetics

MH - Male

MH - Mosaicism

MH - Sex Chromosome Aberrations

OTO - NOTNLM

OT - deletion Xq

OT - klinefelter syndrome

OT - medical genetics

OT - urology

OT - variant klinefelter syndrome

EDAT- 2021/08/11 06:00

MHDA- 2021/11/04 06:00

CRDT- 2021/08/10 12:42

PHST- 2021/08/01 00:00 [revised]

PHST- 2021/06/12 00:00 [received]

PHST- 2021/08/02 00:00 [accepted]

PHST- 2021/08/11 06:00 [pubmed]

PHST- 2021/11/04 06:00 [medline]

PHST- 2021/08/10 12:42 [entrez]

AID - 10.1111/and.14213 [doi]

PST - ppublish

SO - Andrologia. 2021 Dec;53(11):e14213. doi: 10.1111/and.14213. Epub 2021 Aug 10.

PMID- 34038819

OWN - NLM

STAT- MEDLINE

DCOM- 20210624

LR - 20220731

IS - 1872-7506 (Electronic)

IS - 0925-4927 (Print)

IS - 0925-4927 (Linking)

VI - 313

DP - 2021 Jul 30

TI - Cortical gray matter structure in boys with Klinefelter syndrome.

PG - 111299

LID - S0925-4927(21)00051-2 [pii]

LID - 10.1016/j.pscychresns.2021.111299 [doi]

AB - Klinefelter syndrome (KS, 47,XXY) is a common sex chromosome aneuploidy in males

that is associated with a wide range of cognitive, social and emotional

characteristics. The neural bases of these symptoms, however, are unclear. Brain

structure in 19 pre- or early-pubertal boys with KS (11.5 ± 1.8 years) and 22

typically developing (control) boys (8.1 ± 2.3 years) was examined using

surface-based analyses of cortical gray matter volume, thickness and surface

area. Boys in the KS group were treatment-naïve with respect to testosterone

replacement therapy. Reduced volume in the insula and dorsomedial prefrontal

cortex was observed in the KS relative to the TD group, as well as increased

volume in the parietal, occipital and motor regions. Further inspection of

surface-based metrics indicated that whereas KS-associated increases in volume

were driven by differences in thickness, KS-associated reductions in volume were

associated with decreases in surface area. Exploratory analyses additionally

indicated several correlations between brain structure and behavior, providing

initial support for a neural basis of cognitive and emotional symptoms of this

condition. Taken together, these data add support for a neuroanatomical phenotype

of KS and extend previous studies through clarifying the precise neuroanatomical

structural characteristics of that give rise to volumetric alterations.

CI - Copyright © 2021 Elsevier B.V. All rights reserved.

FAU - Foland-Ross, Lara C

AU - Foland-Ross LC

AD - Center for Interdisciplinary Brain Sciences Research, Department of Psychiatry

and Behavioral Sciences, Stanford University, Stanford, CA, United States.

Electronic address: lfolandross@stanford.edu.

FAU - Gil, Maureen

AU - Gil M

AD - Center for Interdisciplinary Brain Sciences Research, Department of Psychiatry

and Behavioral Sciences, Stanford University, Stanford, CA, United States.

FAU - Shrestha, Sharon Bade

AU - Shrestha SB

AD - Center for Interdisciplinary Brain Sciences Research, Department of Psychiatry

and Behavioral Sciences, Stanford University, Stanford, CA, United States.

FAU - Chromik, Lindsay C

AU - Chromik LC

AD - Division of Child Neurology, Stanford University School of Medicine, Stanford,

CA, United States.

FAU - Hong, David

AU - Hong D

AD - Center for Interdisciplinary Brain Sciences Research, Department of Psychiatry

and Behavioral Sciences, Stanford University, Stanford, CA, United States.

FAU - Reiss, Allan L

AU - Reiss AL

AD - Center for Interdisciplinary Brain Sciences Research, Department of Psychiatry

and Behavioral Sciences, Stanford University, Stanford, CA, United States;

Department of Pediatrics, Stanford University School of Medicine, Stanford, CA,

United States; Department of Radiology, Stanford University School of Medicine,

Stanford, CA, United States.

LA - eng

GR - R01 HD049653/HD/NICHD NIH HHS/United States

GR - R01 HD092847/HD/NICHD NIH HHS/United States

GR - R21 MH099630/MH/NIMH NIH HHS/United States

PT - Journal Article

PT - Research Support, N.I.H., Extramural

DEP - 20210504

TA - Psychiatry Res Neuroimaging

JT - Psychiatry research. Neuroimaging

JID - 101723001

SB - IM

MH - Brain

MH - Gray Matter/diagnostic imaging

MH - Humans

MH - *Klinefelter Syndrome/diagnostic imaging/genetics

MH - Male

MH - Phenotype

PMC - PMC8321133

MID - NIHMS1711005

OTO - NOTNLM

OT - *Brain structure

OT - *Genetics

OT - *Klinefelter syndrome

OT - *MRI

OT - *Testosterone

OT - *XXY

COIS- Conflict of Interest The authors report no conflicts of interest.

EDAT- 2021/05/27 06:00

MHDA- 2021/06/25 06:00

CRDT- 2021/05/26 20:13

PHST- 2021/02/01 00:00 [received]

PHST- 2021/04/27 00:00 [revised]

PHST- 2021/04/29 00:00 [accepted]

PHST- 2021/05/27 06:00 [pubmed]

PHST- 2021/06/25 06:00 [medline]

PHST- 2021/05/26 20:13 [entrez]

AID - S0925-4927(21)00051-2 [pii]

AID - 10.1016/j.pscychresns.2021.111299 [doi]

PST - ppublish

SO - Psychiatry Res Neuroimaging. 2021 Jul 30;313:111299. doi:

10.1016/j.pscychresns.2021.111299. Epub 2021 May 4.

PMID- 33370785

OWN - NLM

STAT- MEDLINE

DCOM- 20211025

LR - 20211025

IS - 1944-7558 (Electronic)

IS - 1944-7558 (Linking)

VI - 126

IP - 1

DP - 2021 Jan 1

TI - Social Management Training in Males With 47,XXY (Klinefelter Syndrome): A Pilot

Study of a Neurocognitive-Behavioral Treatment Targeting Social, Emotional, and

Behavioral Problems.

PG - 1-13

LID - 10.1352/1944-7558-126.1.1 [doi]

AB - Klinefelter syndrome (47,XXY) is associated with problems in social interaction

and behavioral adaptation. Sixteen adolescents and adult men with 47,XXY enrolled

in a pilot-study evaluating the effectiveness of Social Management Training

(SMT), a novel neurocognitive-behavioral treatment program targeted at improving

social, emotional, and behavioral functioning. Participants reported improved

emotional stability from pre- to post-test (5 months). Informants reported

reductions in internalizing and externalizing symptoms, including improvement in

self-regulation. Although informants did not report changes in autism-like

symptoms, increased awareness of social challenges was found. SMT may improve

emotional stability, self-regulation, and self-reflection in people males with

Klinefelter syndrome. This potentially efficacious treatment approach may prove

to be a promising psychosocial therapeutic intervention for this population.

CI - ©AAIDD.

FAU - Martin, Francien

AU - Martin F

AD - Francien Martin and Sophie van Rijn, Leiden University.

FAU - van Rijn, Sophie

AU - van Rijn S

AD - Francien Martin and Sophie van Rijn, Leiden University.

FAU - Bierman, Marit

AU - Bierman M

AD - Marit Bierman, GGZ Centraal Fornhese, Clinical Child and Adolescent Psychiatric

Care Centre.

FAU - Swaab, Hanna

AU - Swaab H

AD - Hanna Swaab, Leiden University.

LA - eng

PT - Journal Article

PL - United States

TA - Am J Intellect Dev Disabil

JT - American journal on intellectual and developmental disabilities

JID - 101492916

SB - IM

MH - Adolescent

MH - Adult

MH - *Autistic Disorder

MH - Emotions

MH - Humans

MH - *Klinefelter Syndrome/therapy

MH - Male

MH - Pilot Projects

MH - *Problem Behavior

OTO - NOTNLM

OT - *intervention

OT - *psychosocial treatment

OT - *sex chromosome aneuploidy

OT - *social functioning

EDAT- 2020/12/29 06:00

MHDA- 2021/10/26 06:00

CRDT- 2020/12/28 20:10

PHST- 2017/04/10 00:00 [received]

PHST- 2020/08/05 00:00 [accepted]

PHST- 2020/12/28 20:10 [entrez]

PHST- 2020/12/29 06:00 [pubmed]

PHST- 2021/10/26 06:00 [medline]

AID - 450130 [pii]

AID - 10.1352/1944-7558-126.1.1 [doi]

PST - ppublish

SO - Am J Intellect Dev Disabil. 2021 Jan 1;126(1):1-13. doi:

10.1352/1944-7558-126.1.1.

PMID- 32959501

OWN - NLM

STAT- MEDLINE

DCOM- 20210607

LR - 20210607

IS - 2324-9269 (Electronic)

IS - 2324-9269 (Linking)

VI - 8

IP - 11

DP - 2020 Nov

TI - Identification of common differentially expressed genes in Turner (45,X) and

Klinefelter (47,XXY) syndromes using bioinformatics analysis.

PG - e1503

LID - 10.1002/mgg3.1503 [doi]

LID - e1503

AB - BACKGROUND: Analysis of patients with chromosomal abnormalities, including Turner

syndrome and Klinefelter syndrome, has highlighted the importance of X-linked

gene dosage as a contributing factor for disease susceptibility. Escape from

X-inactivation and X-linked imprinting can result in transcriptional differences

between normal men and women as well as in patients with sex chromosome

abnormalities. OBJECTIVE: To identify differentially expressed genes among

patients with Turner (45,X) and Klinefelter (46,XXY) syndrome using

bioinformatics analysis. METHODOLOGY: Two gene expression data sets of Turner

(45,X) and Klinefelter syndrome (47,XXY) were obtained from the Gene Omnibus

Expression (GEO) database of the National Center for Biotechnology Information

(NCBI). Statistical analysis was performed using R Bioconductor libraries.

Differentially expressed genes (DEGs) were determined using significance analysis

of microarray (SAM). The functional annotation of the DEGs was performed with

DAVID v6.8 (The Database for Annotation, Visualization, and Integrated

Discovery). RESULTS: There are no genes over-expressed simultaneously in both

diseases. However, when crossing the list of under-expressed genes for 45,X cells

and the list of over-expressed genes for 47,XXY cells, there are 16 common genes:

SLC25A6, AKAP17A, ASMTL, KDM5C, KDM6A, ATRX, CSF2RA, DHRSX, CD99, ZBED1, EIF1AX,

MVB12B, SMC1A, P2RY8, DOCK7, DDX3X, eight of which are involved in the regulation

of gene expression by epigenetic mechanisms, regulation of splicing processes and

protein synthesis. CONCLUSION: Of the 16 identified as under-expressed in 45,X

cells and over-expressed in 47,XXY cells, 14 are located in X chromosome and 2 in

autosomal chromosome; 8 of these genes are involved in the regulation of gene

expression: 5 genes are related to epigenetic mechanisms, 2 in regulation of

splicing processes, and 1 in the protein synthesis process. Our results are

limited by it being the product of a bioinformatic analysis from mRNA isolated

from whole blood, this makes necessary further exploration of the relationships

between these genes and Turner syndrome and Klinefelter syndrome in the future.

CI - © 2020 The Authors. Molecular Genetics & Genomic Medicine published by Wiley

Periodicals LLC.

FAU - Manotas, María Carolina

AU - Manotas MC

AUID- ORCID: 0000-0002-4900-4948

AD - Institute of Human Genetics. Faculty of Medicine, Pontificia Universidad

Javeriana, Bogotá, Colombia.

FAU - Calderón, Juan Camilo

AU - Calderón JC

AUID- ORCID: 0000-0002-3433-272X

AD - Department of Statistics, Faculty of Science, Universidad Nacional de Colombia,

Ciudad Universitaria, Bogotá, Colombia.

FAU - López-Kleine, Liliana

AU - López-Kleine L

AUID- ORCID: 0000-0001-9325-9529

AD - Department of Statistics, Faculty of Science, Universidad Nacional de Colombia,

Ciudad Universitaria, Bogotá, Colombia.

FAU - Suárez-Obando, Fernando

AU - Suárez-Obando F

AUID- ORCID: 0000-0003-1005-2210

AD - Institute of Human Genetics. Faculty of Medicine, Pontificia Universidad

Javeriana, Bogotá, Colombia.

FAU - Moreno, Olga M

AU - Moreno OM

AUID- ORCID: 0000-0002-0826-6191

AD - Institute of Human Genetics. Faculty of Medicine, Pontificia Universidad

Javeriana, Bogotá, Colombia.

FAU - Rojas, Adriana

AU - Rojas A

AUID- ORCID: 0000-0001-8528-4433

AD - Institute of Human Genetics. Faculty of Medicine, Pontificia Universidad

Javeriana, Bogotá, Colombia.

LA - eng

PT - Journal Article

PT - Research Support, Non-U.S. Gov't

DEP - 20200921

TA - Mol Genet Genomic Med

JT - Molecular genetics & genomic medicine

JID - 101603758

SB - IM

MH - Chromatin Assembly and Disassembly

MH - DNA Methylation

MH - Epigenesis, Genetic

MH - Gene Expression Profiling

MH - Genetic Loci

MH - Humans

MH - Klinefelter Syndrome/*genetics/metabolism

MH - RNA Splicing

MH - *Transcriptome

MH - Turner Syndrome/*genetics/metabolism

MH - Up-Regulation

PMC - PMC7667333

COIS- None declared.

EDAT- 2020/09/23 06:00

MHDA- 2021/06/08 06:00

CRDT- 2020/09/22 05:47

PHST- 2020/04/08 00:00 [received]

PHST- 2020/08/14 00:00 [revised]

PHST- 2020/08/30 00:00 [accepted]

PHST- 2020/09/23 06:00 [pubmed]

PHST- 2021/06/08 06:00 [medline]

PHST- 2020/09/22 05:47 [entrez]

AID - MGG31503 [pii]

AID - 10.1002/mgg3.1503 [doi]

PST - ppublish

SO - Mol Genet Genomic Med. 2020 Nov;8(11):e1503. doi: 10.1002/mgg3.1503. Epub 2020

Sep 21.

PMID- 21375582

OWN - NLM

STAT- MEDLINE

DCOM- 20110809

LR - 20220408

IS - 1651-2227 (Electronic)

IS - 0803-5253 (Print)

IS - 0803-5253 (Linking)

VI - 100

IP - 6

DP - 2011 Jun

TI - Klinefelter's syndrome (47,XXY) among men with systemic lupus erythematosus.

PG - 819-23

LID - 10.1111/j.1651-2227.2011.02185.x [doi]

AB - AIM: To determine the rate of Klinefelter's syndrome among men with systemic

lupus erythematosus (SLE), and to determine whether the manifestations of SLE in

these men are different from that seen in 46,XY men. METHODS: A total of 276 men

with SLE underwent a real-time PCR procedure to screen for more than one X

chromosome. Those with results consistent with two X chromosomes were further

characterized by karyotype and FISH. Clinical manifestations of SLE were

determined by interview, questionnaire and medical chart review. Each man with

Klinefelter's and SLE was matched to four 46,XY men with SLE. Rates of SLE

manifestations were compared with chi-square analyses. RESULTS: We found seven of

the 286 men with SLE had Klinefelter's syndrome. Four of these seven were

nonmosaic 47,XXY, while two were mosaic 46,XY/47,XXY and one was 46,XX/47,XXY.

The men with 47,XXY did not have severe manifestations of SLE including no

proliferative renal disease, neurological disease, thrombocytopenia, autoimmune

haemolytic anaemia, discoid skin disease or anti-RNP/Sm. CONCLUSION: 47,XXY is

found in excess among men with SLE. Men commonly have SLE that is more severe

than that found among women, but the 47,XXY men had less severe SLE than other

men.

CI - © 2011 The Author(s)/Acta Paediatrica © 2011 Foundation Acta Paediatrica.

FAU - Dillon, Skyler

AU - Dillon S

AD - Department of Medicine, University of Oklahoma Health Sciences Center, Oklahoma

City, USA.

FAU - Aggarwal, Rachna

AU - Aggarwal R

FAU - Harding, James W

AU - Harding JW

FAU - Li, Liang-Jing

AU - Li LJ

FAU - Weissman, Michael H

AU - Weissman MH

FAU - Li, Shibo

AU - Li S

FAU - Cavett, Joshua W

AU - Cavett JW

FAU - Sevier, Sydney T

AU - Sevier ST

FAU - Ojwang, Joshua W

AU - Ojwang JW

FAU - D'Souza, Anil

AU - D'Souza A

FAU - Harley, John B

AU - Harley JB

FAU - Scofield, R Hal

AU - Scofield RH

LA - eng

GR - I01 BX001451/BX/BLRD VA/United States

GR - R01 AR053734/AR/NIAMS NIH HHS/United States

GR - U54 GM104938/GM/NIGMS NIH HHS/United States

PT - Journal Article

DEP - 20110307

TA - Acta Paediatr

JT - Acta paediatrica (Oslo, Norway : 1992)

JID - 9205968

SB - IM

MH - Humans

MH - Klinefelter Syndrome/complications/*epidemiology/genetics

MH - Lupus Erythematosus, Systemic/complications/*epidemiology

MH - Male

MH - Mosaicism

MH - Severity of Illness Index

PMC - PMC7304292

MID - NIHMS1596381

EDAT- 2011/03/08 06:00

MHDA- 2011/08/10 06:00

CRDT- 2011/03/08 06:00

PHST- 2011/03/08 06:00 [entrez]

PHST- 2011/03/08 06:00 [pubmed]

PHST- 2011/08/10 06:00 [medline]

AID - 10.1111/j.1651-2227.2011.02185.x [doi]

PST - ppublish

SO - Acta Paediatr. 2011 Jun;100(6):819-23. doi: 10.1111/j.1651-2227.2011.02185.x.

Epub 2011 Mar 7.

PMID- 31630461

OWN - NLM

STAT- MEDLINE

DCOM- 20200819

LR - 20200819

IS - 1365-2826 (Electronic)

IS - 0953-8194 (Linking)

VI - 31

IP - 11

DP - 2019 Nov

TI - Hypogonadotrophic hypogonadism, delayed puberty and risk for neurodevelopmental

disorders.

PG - e12803

LID - 10.1111/jne.12803 [doi]

AB - Hypogonadotrophic hypogonadism (HH) is a rare disorder that manifests absent

puberty and infertility. Genetic syndromes with hypogonadism, such as Klinefelter

syndrome, are associated with an increased risk of neurodevelopmental disorders

(NDDs). However, it is not clear whether patients with HH or transient delayed

puberty in general, have an increased risk of NDDs. We performed a register-based

study on a national cohort of 264 patients with HH and 7447 patients diagnosed

with delayed puberty that was matched with 2640 and 74 470 controls,

respectively. The outcome was defined as having any of the following NDD

diagnoses: (i) autism spectrum disorder (ASD); (ii) attention deficit

hyperactivity disorder (ADHD); or (iii) intellectual disability (ID). Additional

sensitivity analyses were performed to control for different parental and birth

variables, as well as diagnosed malformation syndromes and chromosomal anomalies

(ie, Down's and Turner syndromes). Patients with HH had increased risk for being

diagnosed with ASD (odds ratio [OR] = 5.7; 95% confidence interval

[CI] = 2.6-12.6), ADHD (OR = 3.0; 95% CI = 1.8-5.1) and ID (OR = 18.0; 95%

CI = 8.9-36.3) compared to controls. Patients with delayed puberty also had a

significantly increased risk of being diagnosed with an NDD. These associations

remained significant after adjustments. This is the first study to demonstrate a

significant association between HH, delayed puberty and NDDs in a

population-based cohort. Clinicians should be aware of the overlap between these

disorders. Further studies should explore the mechanisms behind these

associations.

CI - © 2019 British Society for Neuroendocrinology.

FAU - Ohlsson Gotby, Vide

AU - Ohlsson Gotby V

AD - Department of Medical Epidemiology and Biostatistics, Karolinska Institutet,

Stockholm, Sweden.

FAU - Söder, Olle

AU - Söder O

AD - Division of Pediatric Endocrinology, Department of Women's and Children's Health,

Karolinska Institutet, Stockholm, Sweden.

FAU - Frisén, Louise

AU - Frisén L

AD - Department of Clinical Neuroscience, Centre for Psychiatry Research, Karolinska

Institutete, Stockholm, Sweden.

AD - Stockholm Health Care Services, Stockholm County Council, Stockholm, Sweden.

FAU - Serlachius, Eva

AU - Serlachius E

AD - Department of Clinical Neuroscience, Centre for Psychiatry Research, Karolinska

Institutete, Stockholm, Sweden.

AD - Stockholm Health Care Services, Stockholm County Council, Stockholm, Sweden.

FAU - Bölte, Sven

AU - Bölte S

AD - Stockholm Health Care Services, Stockholm County Council, Stockholm, Sweden.

AD - Division of Neuropsychiatry, Department of Women's and Children's Health, Center

of Neurodevelopmental Disorders at Karolinska Institutet (KIND), Centre for

Psychiatry Research, Karolinska Institutet, Stockholm, Sweden.

FAU - Almqvist, Catarina

AU - Almqvist C

AD - Department of Medical Epidemiology and Biostatistics, Karolinska Institutet,

Stockholm, Sweden.

AD - Astrid Lindgren Children's Hospital, Karolinska University Hospital, Stockholm,

Sweden.

FAU - Larsson, Henrik

AU - Larsson H

AD - Department of Medical Epidemiology and Biostatistics, Karolinska Institutet,

Stockholm, Sweden.

AD - School of Medical Sciences, Örebro University, Örebro, Sweden.

FAU - Lichtenstein, Paul

AU - Lichtenstein P

AD - Department of Medical Epidemiology and Biostatistics, Karolinska Institutet,

Stockholm, Sweden.

FAU - Tammimies, Kristiina

AU - Tammimies K

AUID- ORCID: 0000-0002-8324-4697

AD - Division of Neuropsychiatry, Department of Women's and Children's Health, Center

of Neurodevelopmental Disorders at Karolinska Institutet (KIND), Centre for

Psychiatry Research, Karolinska Institutet, Stockholm, Sweden.

AD - Astrid Lindgren Children's Hospital, Karolinska University Hospital, Stockholm,

Sweden.

LA - eng

PT - Journal Article

PT - Research Support, Non-U.S. Gov't

DEP - 20191112

PL - United States

TA - J Neuroendocrinol

JT - Journal of neuroendocrinology

JID - 8913461

SB - IM

MH - Attention Deficit Disorder with Hyperactivity/complications/epidemiology

MH - Autism Spectrum Disorder/complications/epidemiology

MH - Case-Control Studies

MH - Child

MH - Cohort Studies

MH - Comorbidity

MH - Female

MH - Humans

MH - Hypogonadism/complications/*epidemiology

MH - Klinefelter Syndrome/complications/epidemiology

MH - Male

MH - Neurodevelopmental Disorders/*epidemiology/*etiology

MH - Puberty, Delayed/*epidemiology/etiology

MH - Registries/statistics & numerical data

MH - Risk Factors

OTO - NOTNLM

OT - *International Classification of Diseases

OT - *attention deficit hyperactivity disorder

OT - *autism spectrum disorder

OT - *intellectual disability

OT - *sex hormones

EDAT- 2019/10/21 06:00

MHDA- 2020/08/20 06:00

CRDT- 2019/10/21 06:00

PHST- 2019/02/22 00:00 [received]

PHST- 2019/08/31 00:00 [revised]

PHST- 2019/10/18 00:00 [accepted]

PHST- 2019/10/21 06:00 [pubmed]

PHST- 2020/08/20 06:00 [medline]

PHST- 2019/10/21 06:00 [entrez]

AID - 10.1111/jne.12803 [doi]

PST - ppublish

SO - J Neuroendocrinol. 2019 Nov;31(11):e12803. doi: 10.1111/jne.12803. Epub 2019 Nov

12.

PMID- 26018944

OWN - NLM

STAT- MEDLINE

DCOM- 20161108

LR - 20161230

IS - 1469-7661 (Electronic)

IS - 1355-6177 (Linking)

VI - 21

IP - 5

DP - 2015 May

TI - Social Attention in 47,XXY (Klinefelter Syndrome): Visual Scanning of Facial

Expressions Using Eyetracking.

PG - 364-72

LID - 10.1017/S1355617715000302 [doi]

AB - Boys and men with an extra X chromosome (47,XXY, Klinefelter syndrome) are at

risk for problems in social functioning and have an increased vulnerability for

autism spectrum disorders (ASD). In the search for underlying mechanisms driving

this increased risk, this study focused on social attention, that is, spontaneous

orientation toward facial expressions. Seventeen adults with 47,XXY and 20

non-clinical controls participated in this study. Social attention was measured

using an eyetracking method that quantifies the visual scanning patterns of faces

expressing different types of emotions (happy, fearful, angry, neutral) and their

varying intensity levels (25%, 50%, 75%, 100%). Overall, the group with

Klinefelter syndrome fixated less on the eye region of faces when compared to

controls (Cohen's d 1.4), and did not show the typical tendency, as was found in

the control group, to first fixate on the eyes when presented with a face

(Cohen's d 1.0). There was no significant effect of type or intensity of emotion.

Shorter looking times toward eyes showed a borderline significant correlation

with self-reports of poorer social functioning, with 29% explained variance.

These findings suggest a reduced tendency to rapidly and automatically attend to

the eyes of others in individuals with 47,XXY. This may have impact on more

complex social-cognitive abilities that build upon this. In addition to studies

of behaviorally defined disorders such as ASD, studying individuals with

Klinefelter syndrome provide insight into mechanisms underlying various "at risk"

pathways of social dysfunction and the factors that mediate this risk.

FAU - van Rijn, Sophie

AU - van Rijn S

AD - 1Clinical Child and Adolescent Studies,Leiden University,Leiden,the Netherlands.

LA - eng

PT - Journal Article

DEP - 20150528

PL - England

TA - J Int Neuropsychol Soc

JT - Journal of the International Neuropsychological Society : JINS

JID - 9503760

SB - IM

MH - Adult

MH - Analysis of Variance

MH - Attention Deficit Disorder with Hyperactivity/*etiology

MH - Eye Movements/physiology

MH - *Facial Expression

MH - Humans

MH - Intelligence Tests

MH - Klinefelter Syndrome/*complications/*psychology

MH - Male

MH - Middle Aged

MH - Mood Disorders/*etiology

MH - Neuropsychological Tests

MH - Photic Stimulation

MH - *Social Behavior

OTO - NOTNLM

OT - Klinefelter

OT - autism

OT - emotion

OT - eyetracking

OT - faces

OT - social attention

EDAT- 2015/05/29 06:00

MHDA- 2016/11/09 06:00

CRDT- 2015/05/29 06:00

PHST- 2015/05/29 06:00 [entrez]

PHST- 2015/05/29 06:00 [pubmed]

PHST- 2016/11/09 06:00 [medline]

AID - S1355617715000302 [pii]

AID - 10.1017/S1355617715000302 [doi]

PST - ppublish

SO - J Int Neuropsychol Soc. 2015 May;21(5):364-72. doi: 10.1017/S1355617715000302.

Epub 2015 May 28.

PMID- 32519473

OWN - NLM

STAT- MEDLINE

DCOM- 20210505

LR - 20210817

IS - 1552-4876 (Electronic)

IS - 1552-4868 (Print)

IS - 1552-4868 (Linking)

VI - 184

IP - 2

DP - 2020 Jun

TI - Executive function in XXY: Comparison of performance-based measures and rating

scales.

PG - 469-481

LID - 10.1002/ajmg.c.31804 [doi]

AB - Few studies have systematically assessed executive functioning (EF) skills in

boys with XXY, and these are limited by small samples and restricted EF

assessment. This study used a broader battery of performance-based measures as

well as parent-rating scales of EF in 77 boys and adolescents with XXY (mean age

= 12.5 years), recruited from a clinical trial and an outpatient clinic.

Exploratory factor analyses were used to create EF domains from performance-based

measures, and similar domains were measured using the Behavior Rating Inventory

of Executive Function and Conners Parent-Rating Scales. The boys with XXY showed

a distinct EF profile, with the greatest deficit in attention and more moderate

deficits in working memory, switching, and planning/problem solving. Parent

ratings showed similar challenges, as well as impaired inhibition. Independent

sample t-tests showed no difference on performance measures between boys

diagnosed or not diagnosed with attention-deficit/hyperactivity disorder (ADHD),

although parents of boys diagnosed with ADHD reported more difficulties. There

were no differences on performance-based tests between those diagnosed pre- and

postnatally, although parents of postnatally diagnosed boys reported more

metacognitive problems. Language deficits, cognition, and socio-economic status

did not account for EF deficits.

CI - © 2020 Wiley Periodicals LLC.

FAU - Janusz, Jennifer

AU - Janusz J

AUID- ORCID: 0000-0002-6877-0947

AD - Department of Pediatrics, University of Colorado School of Medicine, Aurora,

Colorado, USA.

AD - Division of Neurology, Children's Hospital Colorado, Aurora, Colorado, USA.

AD - eXtraordinarY Kids Program, Children's Hospital Colorado, Aurora, Colorado, USA.

FAU - Harrison, Caroline

AU - Harrison C

AD - Department of Pediatrics, University of Colorado School of Medicine, Aurora,

Colorado, USA.

FAU - Boada, Cristina

AU - Boada C

AD - Department of Pediatrics, University of Colorado School of Medicine, Aurora,

Colorado, USA.

FAU - Cordeiro, Lisa

AU - Cordeiro L

AD - Department of Pediatrics, University of Colorado School of Medicine, Aurora,

Colorado, USA.

FAU - Howell, Susan

AU - Howell S

AD - Department of Pediatrics, University of Colorado School of Medicine, Aurora,

Colorado, USA.

AD - eXtraordinarY Kids Program, Children's Hospital Colorado, Aurora, Colorado, USA.

FAU - Tartaglia, Nicole

AU - Tartaglia N

AUID- ORCID: 0000-0002-8529-6722

AD - Department of Pediatrics, University of Colorado School of Medicine, Aurora,

Colorado, USA.

AD - eXtraordinarY Kids Program, Children's Hospital Colorado, Aurora, Colorado, USA.

FAU - Boada, Richard

AU - Boada R

AD - Department of Pediatrics, University of Colorado School of Medicine, Aurora,

Colorado, USA.

AD - Division of Neurology, Children's Hospital Colorado, Aurora, Colorado, USA.

AD - eXtraordinarY Kids Program, Children's Hospital Colorado, Aurora, Colorado, USA.

LA - eng

GR - R01 HD091251/HD/NICHD NIH HHS/United States

GR - UL1 TR002535/TR/NCATS NIH HHS/United States

GR - K23NS070337/NIH/NINDS/International

GR - UL1TR00235/NIH/NCATS/International

GR - K23 NS070337/NS/NINDS NIH HHS/United States

GR - L40 HD051024/HD/NICHD NIH HHS/United States

PT - Journal Article

PT - Research Support, N.I.H., Extramural

DEP - 20200609

TA - Am J Med Genet C Semin Med Genet

JT - American journal of medical genetics. Part C, Seminars in medical genetics

JID - 101235745

SB - IM

MH - Adolescent

MH - Attention/physiology

MH - Attention Deficit Disorder with Hyperactivity/*physiopathology

MH - Child

MH - Cognition/*physiology

MH - Executive Function/*physiology

MH - Female

MH - Humans

MH - Inhibition, Psychological

MH - Male

MH - Memory, Short-Term/*physiology

MH - Neuropsychological Tests

MH - Parents/psychology

PMC - PMC8363474

MID - NIHMS1721737

OTO - NOTNLM

OT - *Klinefelter syndrome

OT - *XXY

OT - *executive function

COIS- CONFLICT OF INTEREST None.

EDAT- 2020/06/11 06:00

MHDA- 2021/05/06 06:00

CRDT- 2020/06/11 06:00

PHST- 2020/03/10 00:00 [received]

PHST- 2020/04/28 00:00 [revised]

PHST- 2020/05/11 00:00 [accepted]

PHST- 2020/06/11 06:00 [pubmed]

PHST- 2021/05/06 06:00 [medline]

PHST- 2020/06/11 06:00 [entrez]

AID - 10.1002/ajmg.c.31804 [doi]

PST - ppublish

SO - Am J Med Genet C Semin Med Genet. 2020 Jun;184(2):469-481. doi:

10.1002/ajmg.c.31804. Epub 2020 Jun 9.

PMID- 22395004

OWN - NLM

STAT- MEDLINE

DCOM- 20120709

LR - 20130118

IS - 1473-6551 (Electronic)

IS - 1350-7540 (Linking)

VI - 25

IP - 2

DP - 2012 Apr

TI - Advances in research on the neurological and neuropsychiatric phenotype of

Klinefelter syndrome.

PG - 138-43

LID - 10.1097/WCO.0b013e32835181a0 [doi]

AB - PURPOSE OF REVIEW: Klinefelter syndrome, 47,XXY is the most common chromosomal

aberration among men. It represents a naturally occurring human model for studies

of both X-chromosome gene expression and potential androgen effects on brain

development and function. The aim of this review is to combine available brain

imaging and behavioral data to provide an overview of what we have learned about

the neural underpinnings of cognitive, emotional and behavioral dysunctions in

Klinefelter syndrome. RECENT FINDINGS: The behavioral phenotype of 47,XXY is

characterized by language, executive and psychomotor dysfunction, as well as

socioemotional impairment. The prevalence of schizophrenia, attention deficit

hyperactivity disorder, autism spectrum disorders and affective regulation

problems is increased. Neuroimaging studies of children and adults with

Klinefelter syndrome syndrome show characteristic structural changes from typical

individuals. There are increases in the grey matter volume of the sensorimotor

and parietooccipital regions, as well as significant reductions in amygdala,

hippocampal, insular, temporal and inferior-frontal grey matter volumes.

Widespread white matter abnormalities have been revealed, with reductions in some

areas (including anterior cingulate, bilaterally) but increases in others (such

as left parietal lobe). Mechanisms underlying these developmental anomalies could

include imbalance in gene dosage relative to typical men or women, as well as the

potential consequence of endocrinological deficits. SUMMARY: Studies of

Klinefelter syndrome could generate important information about the impact of

anomalies in sex chromosome gene regulation on the development of cerebral grey

and white matter and, ultimately, on human behavior.

FAU - Savic, Ivanka

AU - Savic I

AD - Department of Clinical Neuroscience, Karolinska Institute, Solna, Stockholm,

Sweden. ivanka.savic-berglund@ki.se

LA - eng

PT - Journal Article

PT - Research Support, Non-U.S. Gov't

PT - Review

PL - England

TA - Curr Opin Neurol

JT - Current opinion in neurology

JID - 9319162

SB - IM

MH - Brain/pathology/physiopathology

MH - Cognition Disorders/etiology

MH - Humans

MH - Klinefelter Syndrome/*complications/pathology

MH - Language Disorders/etiology

MH - Male

MH - Mental Disorders/*etiology

MH - Nervous System Diseases/*etiology/pathology

MH - Neuroimaging

MH - Phenotype

MH - Psychomotor Disorders/etiology

MH - *Sex Chromosome Aberrations

EDAT- 2012/03/08 06:00

MHDA- 2012/07/10 06:00

CRDT- 2012/03/08 06:00

PHST- 2012/03/08 06:00 [entrez]

PHST- 2012/03/08 06:00 [pubmed]

PHST- 2012/07/10 06:00 [medline]

AID - 00019052-201204000-00007 [pii]

AID - 10.1097/WCO.0b013e32835181a0 [doi]

PST - ppublish

SO - Curr Opin Neurol. 2012 Apr;25(2):138-43. doi: 10.1097/WCO.0b013e32835181a0.

PMID- 32468713

OWN - NLM

STAT- MEDLINE

DCOM- 20210505

LR - 20210505

IS - 1552-4876 (Electronic)

IS - 1552-4868 (Linking)

VI - 184

IP - 2

DP - 2020 Jun

TI - Psychological functioning, brain morphology, and functional neuroimaging in

Klinefelter syndrome.

PG - 506-517

LID - 10.1002/ajmg.c.31806 [doi]

AB - Klinefelter syndrome (KS; 47,XXY) impacts neurodevelopment and is associated with

an increased risk of cognitive, psychological and social impairments, although

significant heterogeneity in the neurodevelopmental profile is seen. KS is

characterized by a specific cognitive profile with predominantly verbal deficits,

preserved function in non-verbal and visuo-spatial domains, executive dysfunction

and social impairments, and by an increased vulnerability toward psychiatric

disorders. The neurobiological underpinnings of the observed neuropsychological

profile have not been established. A distinct pattern of both global and regional

brain volumetric differences has been demonstrated in addition to preliminary

findings of functional brain alterations related to auditory, motor, language and

social processing. When present, the combination of cognitive, psychological and

social challenges has the potential to negatively affect quality of life. This

review intends to provide information and insight to the neuropsychological

outcome and brain correlates of KS. Possible clinical intervention and future

directions of research will be discussed.

CI - © 2020 Wiley Periodicals, Inc.

FAU - Skakkebaek, Anne

AU - Skakkebaek A

AUID- ORCID: 0000-0001-9178-4901

AD - Department of Clinical Genetics, Aarhus University Hospital, Aarhus, Denmark.

AD - Department of Endocrinology and Internal Medicine, Aarhus University Hospital,

Aarhus, Denmark.

FAU - Gravholt, Claus H

AU - Gravholt CH

AUID- ORCID: 0000-0001-5924-1720

AD - Department of Endocrinology and Internal Medicine, Aarhus University Hospital,

Aarhus, Denmark.

AD - Department of Molecular Medicine, Aarhus University Hospital, Aarhus, Denmark.

FAU - Chang, Simon

AU - Chang S

AUID- ORCID: 0000-0003-1130-3659

AD - Department of Endocrinology and Internal Medicine, Aarhus University Hospital,

Aarhus, Denmark.

AD - Department of Internal Medicine, Lillebaelt Hospital, Kolding, Denmark.

FAU - Moore, Philip J

AU - Moore PJ

AD - Department of Psychological and Brain Sciences, The George Washington University,

Washington, DC, USA.

FAU - Wallentin, Mikkel

AU - Wallentin M

AD - Department of Linguistics, Cognitive Science, and Semiotics, Aarhus University,

Aarhus, Denmark.

AD - Center of Functionally Integrative Neuroscience, Aarhus University Hospital,

Aarhus, Denmark.

LA - eng

PT - Journal Article

PT - Research Support, Non-U.S. Gov't

PT - Review

DEP - 20200528

PL - United States

TA - Am J Med Genet C Semin Med Genet

JT - American journal of medical genetics. Part C, Seminars in medical genetics

JID - 101235745

SB - IM

MH - Brain/*diagnostic imaging/physiopathology

MH - Cognition Disorders/*diagnostic imaging/physiopathology

MH - *Functional Neuroimaging

MH - Humans

MH - Klinefelter Syndrome/*diagnostic imaging/physiopathology/psychology

MH - Quality of Life

OTO - NOTNLM

OT - *Klinefelter syndrome

OT - *brain morphology

OT - *cognition

OT - *neuropsychology

OT - *sex chromosomes

EDAT- 2020/05/30 06:00

MHDA- 2021/05/06 06:00

CRDT- 2020/05/30 06:00

PHST- 2020/02/28 00:00 [received]

PHST- 2020/05/11 00:00 [revised]

PHST- 2020/05/12 00:00 [accepted]

PHST- 2020/05/30 06:00 [pubmed]

PHST- 2021/05/06 06:00 [medline]

PHST- 2020/05/30 06:00 [entrez]

AID - 10.1002/ajmg.c.31806 [doi]

PST - ppublish

SO - Am J Med Genet C Semin Med Genet. 2020 Jun;184(2):506-517. doi:

10.1002/ajmg.c.31806. Epub 2020 May 28.

PMID- 21217607

OWN - NLM

STAT- MEDLINE

DCOM- 20110303

LR - 20220408

IS - 1565-4753 (Print)

IS - 1565-4753 (Linking)

VI - 8 Suppl 1

IP - 0 1

DP - 2010 Dec

TI - The spectrum of the behavioral phenotype in boys and adolescents 47,XXY

(Klinefelter syndrome).

PG - 151-9

AB - The behavioral phenotype of 47,XXY (Klinefelter syndrome) includes increased

risks for developmental delays, language-based learning disabilities, executive

dysfunction/ADHD, and socialemotional difficulties. However there is significant

variability between individuals with 47,XXY, and many children and adolescents

have minimal or no behavioral features while others have quite significant

involvement. This paper describes behavioral features in a cohort of 57 children

and adolescents with 47,XXY, including results on standardized measures of

behavior (BASC-2), attention (Conner's Rating Scales), and social skills (Social

Responsiveness Scale). A subset was directly assessed for autism spectrum

disorders using the ADOS and ADIR. We discuss our results within the context of

previous literature, including implications for genetic counseling,

recommendations for care, and areas for future research.

FAU - Tartaglia, Nicole

AU - Tartaglia N

AD - University of Colorado School of Medicine, Department of Pediatrics, and Child

Development Unit, The Children's Hospital, Aurora, CO 80045, USA.

Tartaglia.nicole@tchden.org

FAU - Cordeiro, Lisa

AU - Cordeiro L

FAU - Howell, Susan

AU - Howell S

FAU - Wilson, Rebecca

AU - Wilson R

FAU - Janusz, Jennifer

AU - Janusz J

LA - eng

GR - L40 HD051024/HD/NICHD NIH HHS/United States

PT - Journal Article

PT - Review

TA - Pediatr Endocrinol Rev

JT - Pediatric endocrinology reviews : PER

JID - 101202124

SB - IM

MH - Adolescent

MH - Attention Deficit Disorder with Hyperactivity/psychology

MH - Child

MH - Child Development Disorders, Pervasive/psychology

MH - Cognition

MH - Humans

MH - Klinefelter Syndrome/*psychology

MH - Language Development Disorders/psychology

MH - Male

MH - Social Behavior

MH - Young Adult

PMC - PMC3740580

MID - NIHMS495649

COIS- Disclosure The authors of this article declare no conflict of interest.

EDAT- 2011/02/09 06:00

MHDA- 2011/03/04 06:00

CRDT- 2011/01/11 06:00

PHST- 2011/01/11 06:00 [entrez]

PHST- 2011/02/09 06:00 [pubmed]

PHST- 2011/03/04 06:00 [medline]

PST - ppublish

SO - Pediatr Endocrinol Rev. 2010 Dec;8 Suppl 1(0 1):151-9.

PMID- 24476718

OWN - NLM

STAT- MEDLINE

DCOM- 20141204

LR - 20220129

IS - 1559-2308 (Electronic)

IS - 1559-2294 (Print)

IS - 1559-2294 (Linking)

VI - 9

IP - 4

DP - 2014 Apr

TI - Epigenomic and transcriptomic signatures of a Klinefelter syndrome (47,XXY)

karyotype in the brain.

PG - 587-99

LID - 10.4161/epi.27806 [doi]

AB - Klinefelter syndrome (KS) is the most common sex-chromosome aneuploidy in humans.

Most affected individuals carry one extra X-chromosome (47,XXY karyotype) and the

condition presents with a heterogeneous mix of reproductive, physical and

psychiatric phenotypes. Although the mechanism(s) by which the supernumerary

X-chromosome determines these features of KS are poorly understood, skewed

X-chromosome inactivation (XCI), gene-dosage dysregulation, and the parental

origin of the extra X-chromosome have all been implicated, suggesting an

important role for epigenetic processes. We assessed genomic, methylomic and

transcriptomic variation in matched prefrontal cortex and cerebellum samples

identifying an individual with a 47,XXY karyotype who was comorbid for

schizophrenia and had a notably reduced cerebellum mass compared with other

individuals in the study (n = 49). We examined methylomic and transcriptomic

differences in this individual relative to female and male samples with 46,XX or

46,XY karyotypes, respectively, and identified numerous locus-specific

differences in DNA methylation and gene expression, with many differences being

autosomal and tissue-specific. Furthermore, global DNA methylation, assessed via

the interrogation of LINE-1 and Alu repetitive elements, was significantly

altered in the 47,XXY patient in a tissue-specific manner with extreme

hypomethylation detected in the prefrontal cortex and extreme hypermethylation in

the cerebellum. This study provides the first detailed molecular characterization

of the prefrontal cortex and cerebellum from an individual with a 47,XXY

karyotype, identifying widespread tissue-specific epigenomic and transcriptomic

alterations in the brain.

FAU - Viana, Joana

AU - Viana J

AD - University of Exeter Medical School; Exeter University; Exeter, UK.

FAU - Pidsley, Ruth

AU - Pidsley R

AD - Institute of Psychiatry; King's College London; London, UK; Garvan Institute of

Medical Research; Sydney, NSW Australia.

FAU - Troakes, Claire

AU - Troakes C

AD - Institute of Psychiatry; King's College London; London, UK.

FAU - Spiers, Helen

AU - Spiers H

AD - Institute of Psychiatry; King's College London; London, UK.

FAU - Wong, Chloe Cy

AU - Wong CC

AD - Institute of Psychiatry; King's College London; London, UK.

FAU - Al-Sarraj, Safa

AU - Al-Sarraj S

AD - Institute of Psychiatry; King's College London; London, UK.

FAU - Craig, Ian

AU - Craig I

AD - Institute of Psychiatry; King's College London; London, UK.

FAU - Schalkwyk, Leonard

AU - Schalkwyk L

AD - Institute of Psychiatry; King's College London; London, UK.

FAU - Mill, Jonathan

AU - Mill J

AD - University of Exeter Medical School; Exeter University; Exeter, UK; Institute of

Psychiatry; King's College London; London, UK.

LA - eng

GR - G1100695/MRC_/Medical Research Council/United Kingdom

GR - MR/K013807/1/MRC_/Medical Research Council/United Kingdom

PT - Journal Article

PT - Research Support, Non-U.S. Gov't

DEP - 20140129

TA - Epigenetics

JT - Epigenetics

JID - 101265293

SB - IM

MH - Alu Elements

MH - Brain/*metabolism

MH - Case-Control Studies

MH - Cerebellum/metabolism

MH - DNA Methylation

MH - *Epigenesis, Genetic

MH - Female

MH - Humans

MH - Klinefelter Syndrome/complications/*genetics/metabolism

MH - Long Interspersed Nucleotide Elements

MH - Male

MH - Prefrontal Cortex/metabolism

MH - Schizophrenia/complications

MH - *Transcriptome

PMC - PMC4121369

OTO - NOTNLM

OT - 47,XXY

OT - DNA methylation

OT - Klinefelter syndrome

OT - cerebellum

OT - gene expression

OT - prefrontal cortex

EDAT- 2014/01/31 06:00

MHDA- 2014/12/15 06:00

CRDT- 2014/01/31 06:00

PHST- 2014/01/31 06:00 [entrez]

PHST- 2014/01/31 06:00 [pubmed]

PHST- 2014/12/15 06:00 [medline]

AID - 27806 [pii]

AID - 2013EPI0431R1 [pii]

AID - 10.4161/epi.27806 [doi]

PST - ppublish

SO - Epigenetics. 2014 Apr;9(4):587-99. doi: 10.4161/epi.27806. Epub 2014 Jan 29.

PMID- 30592247

OWN - NLM

STAT- MEDLINE

DCOM- 20200417

LR - 20210524

IS - 1541-3144 (Electronic)

IS - 0194-2638 (Print)

IS - 0194-2638 (Linking)

VI - 39

IP - 4

DP - 2019

TI - The Association of Motor Skills and Adaptive Functioning in XXY/Klinefelter and

XXYY Syndromes.

PG - 446-459

LID - 10.1080/01942638.2018.1541040 [doi]

AB - Aims: Klinefelter (XXY) and XXYY syndromes are genetic disorders in males

characterized by additional sex chromosomes compared to the typical male

karyotype of 46, XY. Both conditions have been previously associated with motor

delays and motor skills deficits. We aimed to describe and compare motor skills

in males with XXY and XXYY syndromes, and to analyze associations with age,

cognitive abilities, and adaptive functioning. Methods: Sixty-four males with XXY

and 46 males with XXYY, ages 4-20 were evaluated using the Beery Test of Visual

Motor Integration and the Bruininks-Oseretsky Test of Motor Proficiency - 2nd

Edition assessments, Vineland-2 adaptive scales, and cognitive testing. Results:

Motor coordination impairments were found in 39% of the males with XXY and 73% of

the males with XXYY. Both groups showed strengths in visual perceptual skills.

Males with XXYY had lower mean scores compared to males with XXY across all

assessments. Fine motor dexterity and coordination deficits were common. There

was a positive correlation between VMI scores and adaptive functioning.

Conclusion: Occupational and physical therapists should be aware of the motor

phenotype in XXY and XXYY both to aid in diagnosis of unidentified cases and to

guide intervention.

FAU - Martin, Sydney

AU - Martin S

AD - a Occupational Therapy Department , Children's Hospital Colorado , Aurora ,

Colorado , USA.

AD - b Department of Occupational Therapy , San Jose State University , San Jose ,

California , USA.

FAU - Cordeiro, Lisa

AU - Cordeiro L

AD - c Department of Pediatrics , University of Colorado School of Medicine , Aurora ,

Colorado , USA.

FAU - Richardson, Pamela

AU - Richardson P

AD - b Department of Occupational Therapy , San Jose State University , San Jose ,

California , USA.

FAU - Davis, Shanlee

AU - Davis S

AD - c Department of Pediatrics , University of Colorado School of Medicine , Aurora ,

Colorado , USA.

FAU - Tartaglia, Nicole

AU - Tartaglia N

AD - a Occupational Therapy Department , Children's Hospital Colorado , Aurora ,

Colorado , USA.

AD - c Department of Pediatrics , University of Colorado School of Medicine , Aurora ,

Colorado , USA.

LA - eng

GR - K23 HD092588/HD/NICHD NIH HHS/United States

GR - K23 NS070337/NS/NINDS NIH HHS/United States

GR - L40 HD051024/HD/NICHD NIH HHS/United States

GR - R01 HD091251/HD/NICHD NIH HHS/United States

PT - Journal Article

DEP - 20181228

TA - Phys Occup Ther Pediatr

JT - Physical & occupational therapy in pediatrics

JID - 8109120

SB - IM

MH - Adolescent

MH - Child

MH - Child, Preschool

MH - Cross-Sectional Studies

MH - Disability Evaluation

MH - Humans

MH - Klinefelter Syndrome/*physiopathology

MH - Male

MH - Motor Skills Disorders/*physiopathology

MH - Young Adult

PMC - PMC7425033

MID - NIHMS1520202

OTO - NOTNLM

OT - *47

OT - *Klinefelter syndrome

OT - *activities of daily living

OT - *motor skills

OT - *occupational therapy

OT - *physical therapy

OT - *visual motor integration

OT - *xxy

OT - *xxyy

EDAT- 2018/12/29 06:00

MHDA- 2020/04/18 06:00

CRDT- 2018/12/29 06:00

PHST- 2018/12/29 06:00 [pubmed]

PHST- 2020/04/18 06:00 [medline]

PHST- 2018/12/29 06:00 [entrez]

AID - 10.1080/01942638.2018.1541040 [doi]

PST - ppublish

SO - Phys Occup Ther Pediatr. 2019;39(4):446-459. doi: 10.1080/01942638.2018.1541040.

Epub 2018 Dec 28.

PMID- 24407186

OWN - NLM

STAT- MEDLINE

DCOM- 20141114

LR - 20220408

IS - 1745-7262 (Electronic)

IS - 1008-682X (Print)

IS - 1008-682X (Linking)

VI - 16

IP - 2

DP - 2014 Mar-Apr

TI - The role of hypogonadism in Klinefelter syndrome.

PG - 185-91

LID - 10.4103/1008-682X.122201 [doi]

AB - Klinefelter syndrome (KS) (47, XXY) is the most abundant sex-chromosome disorder,

and is a common cause of infertility and hypogonadism in men. Most men with KS go

through life without knowing the diagnosis, as only 25% are diagnosed and only a

few of these before puberty. Apart from hypogonadism and azoospermia, most men

with KS suffer from some degree of learning disability and may have various kinds

of psychiatric problems. The effects of long-term hypogonadism may be diffi cult

to discern from the gene dose effect of the extra X-chromosome. Whatever the

cause, alterations in body composition, with more fat and less muscle mass and

diminished bone mineral mass, as well as increased risk of metabolic

consequences, such as type 2 diabetes and the metabolic syndrome are all common

in KS. These findings should be a concern as they are not simply laboratory

findings; epidemiological studies in KS populations show an increased risk of

both hospitalization and death from various diseases. Testosterone treatment

should be offered to KS patients from early puberty, to secure a proper masculine

development, nonetheless the evidence is weak or nonexisting, since no randomized

controlled trials have ever been published. Here, we will review the current

knowledge of hypogonadism in KS and the rationale for testosterone treatment and

try to give our best recommendations for surveillance of this rather common, but

often ignored, syndrome.

FAU - Høst, Christian

AU - Høst C

FAU - Skakkebæk, Anne

AU - Skakkebæk A

FAU - Groth, Kristian A

AU - Groth KA

FAU - Bojesen, Anders

AU - Bojesen A

AD - Department of Clinical Genetics, Vejle Hospital, Vejle; Institute of Regional

Health Research, University of Southern Denmark, Odense, Denmark.

LA - eng

PT - Journal Article

PT - Review

TA - Asian J Androl

JT - Asian journal of andrology

JID - 100942132

RN - 3XMK78S47O (Testosterone)

SB - IM

MH - Body Composition

MH - Brain/physiopathology

MH - Cognition

MH - Fertility

MH - Hormone Replacement Therapy

MH - Humans

MH - Hypogonadism/*etiology/metabolism/physiopathology

MH - Insulin Resistance

MH - Klinefelter Syndrome/*complications/metabolism/physiopathology

MH - Male

MH - Osteoporosis/etiology/physiopathology

MH - Testis/physiopathology

MH - Testosterone/administration & dosage

PMC - PMC3955327

EDAT- 2014/01/11 06:00

MHDA- 2014/11/15 06:00

CRDT- 2014/01/11 06:00

PHST- 2014/01/11 06:00 [entrez]

PHST- 2014/01/11 06:00 [pubmed]

PHST- 2014/11/15 06:00 [medline]

AID - 122201 [pii]

AID - AJA-16-185 [pii]

AID - 10.4103/1008-682X.122201 [doi]

PST - ppublish

SO - Asian J Androl. 2014 Mar-Apr;16(2):185-91. doi: 10.4103/1008-682X.122201.

PMID- 30823838

OWN - NLM

STAT- MEDLINE

DCOM- 20190715

LR - 20190715

IS - 0042-773X (Print)

IS - 0042-773X (Linking)

VI - 65

IP - 1

DP - 2019 Winter

TI - The combination of acromegaly and Klinefelter syndrome in one patient.

PG - 51-54

AB - Acromegaly is a rare disorder usually caused by a benign tumour of the pituitary

gland. Long-term presence of elevated growth hormone (GH) and insulin like growth

factor 1 (IGF1) levels accompanying this disease is associated with complications

such as cardiomyopathy, diabetes mellitus, sleep apnoea and arthropathy.

Incidence of acromegaly is 3-4 patients per million per year. Klinefelter

syndrome (KS) is the most common sex chromosome disorder occuring in about

1/500 live male births. Common physical features include particularly small

testes, among other symptoms are tall stature, reduced muscle tone, delayed

pubertal development, lack of secondary male sex characteristics and

gynecomastia. We present a 32-year-old man suffering from both acromegaly and 47,

XXY Klinefelter syndrome. The patient with typical acromegalic features.

Laboratory tests revealed high level of GH which was not suppressed after glucose

administration, high level of IGF1, low testosterone concentration with high

concentation of luteinizing hormone and follicle stimulating hormone. A magnetic

resonance imaging scan revealed a 25 × 18 × 18 mm macroadenoma involving the

pituitary gland. A diagnosis of acromegaly was established. After this

examination trans-sphenoidal resection was performed. Histopathologic and

immunohistochemical findings revealed growth hormoneproducing pituitary adenoma.

The presence of infertility with clinical features such as small testes, lack of

secondary male sex characteristics and laboratory findings revealed

hypergonadotropic hypogonadism that could not be explained by the diagnosis of

acromegaly. A chromosomal karyotyping revealed a 47, XXY, confirming the

diagnosis of KS. Testosterone replacement therapy wasn´t begun because of patient

disagreement Postoperatively elevated plasma concentration of GH and IGF1 levels

persist. Treatment by somatostatin analogues (lanreotid) was initiated at dose

120 mg every 28 days. Control magnetic resonance imaging of the sella

demonstrated a residue of pituary adenoma size 14 × 14 × 7 mm. The patient is

currently undergoing endoscopic revision of the residue. acromegaly - growth

hormone - IGF1 - Klinefelter syndrome - testosterone.

FAU - Ságová, Ivana

AU - Ságová I

FAU - Pávai, Dušan

AU - Pávai D

FAU - Kantárová, Daniela

AU - Kantárová D

FAU - Vaňuga, Anton

AU - Vaňuga A

FAU - Sadloňová, Jurina

AU - Sadloňová J

FAU - Vaňuga, Peter

AU - Vaňuga P

FAU - Dragula, Milan

AU - Dragula M

LA - eng

PT - Case Reports

PT - Journal Article

TT - Spojenie akromegálie a Klinefelterovho syndrómu u jedného pacienta.

PL - Czech Republic

TA - Vnitr Lek

JT - Vnitrni lekarstvi

JID - 0413602

RN - 12629-01-5 (Human Growth Hormone)

RN - 67763-96-6 (Insulin-Like Growth Factor I)

SB - IM

MH - *Acromegaly/complications/diagnosis/genetics

MH - *Adenoma/complications/diagnosis/genetics

MH - Adult

MH - Human Growth Hormone

MH - Humans

MH - Insulin-Like Growth Factor I

MH - *Klinefelter Syndrome/complications/diagnosis/genetics

MH - Male

MH - *Pituitary Neoplasms/complications/diagnosis/genetics

EDAT- 2019/03/03 06:00

MHDA- 2019/07/16 06:00

CRDT- 2019/03/03 06:00

PHST- 2019/03/03 06:00 [entrez]

PHST- 2019/03/03 06:00 [pubmed]

PHST- 2019/07/16 06:00 [medline]

AID - 107917 [pii]

PST - ppublish

SO - Vnitr Lek. 2019 Winter;65(1):51-54.

PMID- 24793990

OWN - NLM

STAT- MEDLINE

DCOM- 20150717

LR - 20161031

IS - 2213-3941 (Electronic)

IS - 0003-4266 (Linking)

VI - 75

IP - 2

DP - 2014 May

TI - New approaches to the Klinefelter syndrome.

PG - 88-97

LID - S0003-4266(14)00029-8 [pii]

LID - 10.1016/j.ando.2014.03.007 [doi]

AB - The Klinefelter syndrome (KS), with an incidence of 1 to 2 per 1000 male

neonates, is one of the most frequent congenital chromosome disorders. The 47,XXY

karyotype causes infertility, testosterone deficiency and a spectrum of further

symptoms and comorbidities. In recent years, significant progress has been made

in the elucidation of the pathophysiology and the treatment of the KS. It became

clear that, to a large extent, the clinical picture is determined by gene dosage

effects of the supernumerary X-chromosome. The origin of the extra X-chromosome

from either the father or the mother influences behavioural features of patients

with KS. The CAGn polymorphism of the androgen receptor, located on the

X-chromosome, has a distinct impact on the KS phenotype. KS predisposes to the

metabolic syndrome and its cardiovascular sequelae, contributing to the increased

mortality of patients with KS. Neuroimaging studies have correlated anomalies in

brain structures with psychosocial problems. The unexpected possibility to

produce pregnancies and live birth with either ejaculated sperm--about 8% of KS

men have a few sperm in semen--or with sperm extracted from individual tubules

obtained by testicular biopsy can be considered a breakthrough. Testosterone

substitution requires further optimisation in terms of when to initiate therapy

and which preparations and dosages to use. Recently developed animal models help

to further elucidation the genetic and pathophysiological basis and may lead to

new therapeutic approaches to KS.

CI - Copyright © 2014 Elsevier Masson SAS. All rights reserved.

FAU - Nieschlag, Eberhard

AU - Nieschlag E

AD - Centre of Reproductive Medicine and Andrology, University of Münster, D-48129

Münster, Germany; Center of Excellence in Genomic Medicine Research, King

Abdulaziz University, Jeddah, Saudi Arabia. Electronic address:

eberhard.nieschlag@ukmuenster.de.

FAU - Werler, Steffi

AU - Werler S

AD - Centre of Reproductive Medicine and Andrology, University of Münster, D-48129

Münster, Germany.

FAU - Wistuba, Joachim

AU - Wistuba J

AD - Centre of Reproductive Medicine and Andrology, University of Münster, D-48129

Münster, Germany.

FAU - Zitzmann, Michael

AU - Zitzmann M

AD - Centre of Reproductive Medicine and Andrology, University of Münster, D-48129

Münster, Germany.

LA - eng

PT - Journal Article

PT - Research Support, Non-U.S. Gov't

PT - Review

DEP - 20140430

PL - France

TA - Ann Endocrinol (Paris)

JT - Annales d'endocrinologie

JID - 0116744

RN - 0 (RNA, Long Noncoding)

RN - 0 (Receptors, Androgen)

RN - 0 (XIST non-coding RNA)

RN - 3XMK78S47O (Testosterone)

SB - IM

MH - Animals

MH - Chromosomes, Human, X

MH - Disease Models, Animal

MH - Genomic Imprinting

MH - *Hormone Replacement Therapy

MH - Humans

MH - Hypogonadism/genetics/psychology

MH - Klinefelter Syndrome/*drug therapy/genetics/physiopathology/psychology

MH - Male

MH - Mice

MH - Oligospermia/etiology/therapy

MH - Phenotype

MH - RNA, Long Noncoding/biosynthesis/genetics

MH - Receptors, Androgen/genetics

MH - Reproductive Techniques, Assisted

MH - Testosterone/deficiency/*therapeutic use

OTO - NOTNLM

OT - 47,XXY karyotype

OT - Klinefelter

EDAT- 2014/05/06 06:00

MHDA- 2015/07/18 06:00

CRDT- 2014/05/06 06:00

PHST- 2014/03/19 00:00 [received]

PHST- 2014/03/19 00:00 [accepted]

PHST- 2014/05/06 06:00 [entrez]

PHST- 2014/05/06 06:00 [pubmed]

PHST- 2015/07/18 06:00 [medline]

AID - S0003-4266(14)00029-8 [pii]

AID - 10.1016/j.ando.2014.03.007 [doi]

PST - ppublish

SO - Ann Endocrinol (Paris). 2014 May;75(2):88-97. doi: 10.1016/j.ando.2014.03.007.

Epub 2014 Apr 30.

PMID- 21540567

OWN - NLM

STAT- MEDLINE

DCOM- 20111027

LR - 20220408

IS - 1661-5433 (Electronic)

IS - 1661-5425 (Linking)

VI - 5

IP - 3

DP - 2011

TI - Chromosomal variants in klinefelter syndrome.

PG - 109-23

LID - 10.1159/000327324 [doi]

AB - Klinefelter syndrome (KS) describes the phenotype of the most common sex

chromosome abnormality in humans and occurs in one of every 600 newborn males.

The typical symptoms are a tall stature, narrow shoulders, broad hips, sparse

body hair, gynecomastia, small testes, absent spermatogenesis, normal to

moderately reduced Leydig cell function, increased secretion of

follicle-stimulating hormone, androgen deficiency, and normal to slightly

decreased verbal intelligence. Apart from that, amongst others, osteoporosis,

varicose veins, thromboembolic disease, or diabetes mellitus are observed. Some

of the typical features can be very weakly pronounced so that the affected men

often receive the diagnosis only at the adulthood by their infertility. With a

frequency of 4%, KS is described to be the most common genetic reason for male

infertility. The most widespread karyotype in affected patients is 47,XXY. Apart

from that, various other karyotypes have been described, including 46,XX in

males, 47,XXY in females, 47,XX,der(Y), 47,X,der(X),Y, or other numeric sex

chromosome abnormalities (48,XXXY, 48,XXYY, and 49,XXXXY). The focus of this

review was to abstract the different phenotypes, which come about by the various

karyotypes and to compare them to those with a 'normal' KS karyotype. For that

the patients have been divided into 6 different groups: Klinefelter patients with

an additional isochromosome Xq, with additional rearrangements on 1 of the 2 X

chromosomes or accordingly on the Y chromosome, as well as XX males and true

hermaphrodites, 47,XXY females and Klinefelter patients with other numeric sex

chromosome abnormalities. In the latter, an almost linear increase in height and

developmental delay was observed. Men with an additional isochromosome Xq show

infertility and other minor features of 'normal' KS but not an increased height.

Aside from the infertility, in male patients with other der(X) as well as der(Y)

rearrangements and in XXY women no specific phenotype is recognizable amongst

others due to the small number of cases. The phenotype of XX males depends on the

presence of SRY (sex-determining region Y) and the level of X inactivation at

which SRY-negative patients are generally rarely observed.

CI - Copyright © 2011 S. Karger AG, Basel.

FAU - Frühmesser, A

AU - Frühmesser A

AD - Division for Human Genetics, Department for Medical Genetics, Molecular and

Clinical Pharmacology, Innsbruck Medical University, Austria.

FAU - Kotzot, D

AU - Kotzot D

LA - eng

PT - Journal Article

PT - Review

DEP - 20110429

PL - Switzerland

TA - Sex Dev

JT - Sexual development : genetics, molecular biology, evolution, endocrinology,

embryology, and pathology of sex determination and differentiation

JID - 101316472

SB - IM

MH - Chromosomes, Human, X/genetics

MH - Chromosomes, Human, Y/genetics

MH - Female

MH - Humans

MH - Karyotyping

MH - Klinefelter Syndrome/*genetics

MH - Male

MH - *Sex Chromosome Aberrations

EDAT- 2011/05/05 06:00

MHDA- 2011/10/28 06:00

CRDT- 2011/05/05 06:00

PHST- 2011/02/03 00:00 [accepted]

PHST- 2011/05/05 06:00 [entrez]

PHST- 2011/05/05 06:00 [pubmed]

PHST- 2011/10/28 06:00 [medline]

AID - 000327324 [pii]

AID - 10.1159/000327324 [doi]

PST - ppublish

SO - Sex Dev. 2011;5(3):109-23. doi: 10.1159/000327324. Epub 2011 Apr 29.

PMID- 32489693

OWN - NLM

STAT- PubMed-not-MEDLINE

LR - 20200928

IS - 2152-5250 (Print)

IS - 2152-5250 (Electronic)

IS - 2152-5250 (Linking)

VI - 11

IP - 3

DP - 2020 May

TI - Molecular Aging Markers in Patients with Klinefelter Syndrome.

PG - 470-476

LID - 10.14336/AD.2019.0801 [doi]

AB - Molecular aging markers provide the opportunity for biological age determination

in humans and to study factors, such as genetic determinants, affecting the

ageing process. In males with Klinefelter syndrome (KS, non-mosaic karyotype 47,

XXY), which is the most common sex chromosome aneuploidy, age-related morbidity

and mortality are increased, and a significantly reduced life span has been

observed. The aim of this study was to investigate whether Klinefelter patients

exhibit molecular signs of premature ageing. We studied, specifically,

age-associated DNA methylation patterns (by pyrosequencing) and relative telomere

length (TL; by quantitative polymerase chain reaction) in blood in a cohort of

Klinefelter patients (n=178 and 266 for DNA methylation and TL, respectively)

aged 18-71 years and compared them to the data of age-matched healthy male (n =

184 and 196 for DNA methylation and TL, respectively) and female controls (n =

50). Age-associated DNA methylation patterns were not indicative of accelerated

ageing in Klinefelter men. Significantly longer telomeres were found in the young

Klinefelter subjects aged 18-24 years (mean=1.51 vs. 1.09 and 1.26 in female and

male controls, respectively). However, telomere length in subsequent age groups

showed no difference to controls. Gonosomal aneuploidy in Klinefelter syndrome is

associated with higher baseline TL at adolescent age, but comparable TL with

progressive age in other age groups.

CI - Copyright: © 2020 Pohl et al.

FAU - Pohl, Eva

AU - Pohl E

AD - 1Institute for Human Genetics, University of Münster, 48149 Münster, Germany.

FAU - Muschal, Sina

AU - Muschal S

AD - 2Department of Clinical and Surgical Andrology, Centre of Reproductive Medicine

and Andrology, University of Münster, 48149 Münster, Germany.

FAU - Kliesch, Sabine

AU - Kliesch S

AD - 2Department of Clinical and Surgical Andrology, Centre of Reproductive Medicine

and Andrology, University of Münster, 48149 Münster, Germany.

FAU - Zitzmann, Michael

AU - Zitzmann M

AD - 2Department of Clinical and Surgical Andrology, Centre of Reproductive Medicine

and Andrology, University of Münster, 48149 Münster, Germany.

FAU - Rohayem, Julia

AU - Rohayem J

AD - 2Department of Clinical and Surgical Andrology, Centre of Reproductive Medicine

and Andrology, University of Münster, 48149 Münster, Germany.

FAU - Gromoll, Jörg

AU - Gromoll J

AD - 3Institute of Reproductive and Regenerative Biology, Centre of Reproductive

Medicine and Andrology, University of Münster, 48149 Münster, Germany.

FAU - Laurentino, Sandra

AU - Laurentino S

AD - 3Institute of Reproductive and Regenerative Biology, Centre of Reproductive

Medicine and Andrology, University of Münster, 48149 Münster, Germany.

LA - eng

PT - Journal Article

DEP - 20200509

TA - Aging Dis

JT - Aging and disease

JID - 101540533

PMC - PMC7220296

OTO - NOTNLM

OT - DNA methylation

OT - Klinefelter syndrome

OT - ageing

OT - telomere length

COIS- Disclosures The authors declare that there is no conflict of interest.

EDAT- 2020/06/04 06:00

MHDA- 2020/06/04 06:01

CRDT- 2020/06/04 06:00

PHST- 2019/03/27 00:00 [received]

PHST- 2019/08/01 00:00 [accepted]

PHST- 2020/06/04 06:00 [entrez]

PHST- 2020/06/04 06:00 [pubmed]

PHST- 2020/06/04 06:01 [medline]

AID - ad-11-3-470 [pii]

AID - 10.14336/AD.2019.0801 [doi]

PST - epublish

SO - Aging Dis. 2020 May 9;11(3):470-476. doi: 10.14336/AD.2019.0801. eCollection 2020

May.

PMID- 33960260

OWN - NLM

STAT- MEDLINE

DCOM- 20211213

LR - 20220423

IS - 1473-0766 (Electronic)

IS - 0951-3590 (Linking)

VI - 37

IP - 6

DP - 2021 Jun

TI - 18-Year-old patient with Klinefelter syndrome (47, XXY) and complete androgen

insensitivity syndrome (CAIS) - case report.

PG - 572-575

LID - 10.1080/09513590.2021.1921139 [doi]

AB - AIMS: The aims of the presented case report are to emphasize the importance of a

proper diagnostics and treatment in the case of the coexistence of Klinefelter

syndrome (KS, 47 XXY) and complete androgen insensitivity syndrome (CAIS). Since

there is no causal treatment it is necessary to provide the patient with a good

quality of life, including psychological and sexological support. MATERIALS AND

METHODS: The presented case report is the retrospective analysis of the patient's

medical history over the 3 years. RESULTS: At the age of 15, the patient was

directed to genetic testing due to primary amenorrhea. The results of the patient

showed an incorrect male karyotype with the SRY gene present (47, XXY). A

molecular diagnostics revealed a very rare variant of the androgen receptor (AR)

mutation responsible for tissue insensitivity to androgens. The detected mutation

has not been described in the available databases so far. Following a diagnosis

of the presence of Klinefelter syndrome (KS, 47 XXY) together with complete

androgen insensitivity syndrome (CAIS), the patient underwent a bilateral

gonadectomy. CONCLUSIONS: In women with KS and CAIS physiological reproduction

and maintenance of normal sex, hormone levels are not possible. A gonadectomy is

performed due to the risk of malignant testicular tumors.

FAU - Skalska, Karolina

AU - Skalska K

AD - Department of Internal Diseases, Pneumology and Allergology, University Clinical

Center, Medical University of Warsaw, Warsaw, Poland.

FAU - Ziółkowski, Maciej

AU - Ziółkowski M

AD - Międzyleski Specialist Hospital in Warsaw, Warsaw, Poland.

FAU - Skoczylas, Adrian

AU - Skoczylas A

AD - Faculty of Medicine, Medical University of Warsaw, Warsaw, Poland.

FAU - Teleon, Marta

AU - Teleon M

AD - Faculty of Medicine, Medical University of Warsaw, Warsaw, Poland.

FAU - Grymowicz, Monika

AU - Grymowicz M

AD - Department of Gynecological Endocrinology, Medical University of Warsaw, Warsaw,

Poland.

FAU - Pollak, Agnieszka

AU - Pollak A

AD - Department of Medical Genetics, Medical University of Warsaw, Warsaw, Poland.

FAU - Smolarczyk, Roman

AU - Smolarczyk R

AD - Department of Gynecological Endocrinology, Medical University of Warsaw, Warsaw,

Poland.

FAU - Płoski, Rafał

AU - Płoski R

AD - Department of Medical Genetics, Medical University of Warsaw, Warsaw, Poland.

FAU - Męczekalski, Błażej

AU - Męczekalski B

AUID- ORCID: 0000-0002-0761-5775

AD - Department of Gynecological Endocrinology, Poznan University of Medical Sciences,

Poznan, Poland.

LA - eng

PT - Case Reports

PT - Journal Article

DEP - 20210507

PL - England

TA - Gynecol Endocrinol

JT - Gynecological endocrinology : the official journal of the International Society

of Gynecological Endocrinology

JID - 8807913

RN - 0 (AR protein, human)

RN - 0 (Receptors, Androgen)

RN - 0 (SRY protein, human)

RN - 0 (Sex-Determining Region Y Protein)

SB - IM

MH - Adolescent

MH - Amenorrhea/diagnosis/etiology/genetics/surgery

MH - Androgen-Insensitivity Syndrome/complications/*diagnosis/genetics/surgery

MH - Castration

MH - Female

MH - Humans

MH - Karyotyping

MH - Klinefelter Syndrome/complications/*diagnosis/genetics/surgery

MH - Male

MH - Mutation

MH - Receptors, Androgen/genetics

MH - Retrospective Studies

MH - Sex-Determining Region Y Protein/genetics

MH - Testis/surgery

OTO - NOTNLM

OT - Klinefelter syndrome

OT - androgen insensitivity syndrome

OT - disorder of sexual development

EDAT- 2021/05/08 06:00

MHDA- 2021/12/15 06:00

CRDT- 2021/05/07 08:54

PHST- 2021/05/08 06:00 [pubmed]

PHST- 2021/12/15 06:00 [medline]

PHST- 2021/05/07 08:54 [entrez]

AID - 10.1080/09513590.2021.1921139 [doi]

PST - ppublish

SO - Gynecol Endocrinol. 2021 Jun;37(6):572-575. doi: 10.1080/09513590.2021.1921139.

Epub 2021 May 7.

PMID- 17062147

OWN - NLM

STAT- MEDLINE

DCOM- 20071002

LR - 20181113

IS - 1750-1172 (Electronic)

IS - 1750-1172 (Linking)

VI - 1

DP - 2006 Oct 24

TI - Klinefelter syndrome and other sex chromosomal aneuploidies.

PG - 42

AB - The term Klinefelter syndrome (KS) describes a group of chromosomal disorder in

which there is at least one extra X chromosome to a normal male karyotype, 46,XY.

XXY aneuploidy is the most common disorder of sex chromosomes in humans, with

prevalence of one in 500 males. Other sex chromosomal aneuploidies have also been

described, although they are much less frequent, with 48,XXYY and 48,XXXY being

present in 1 per 17,000 to 1 per 50,000 male births. The incidence of 49,XXXXY is

1 per 85,000 to 100,000 male births. In addition, 46,XX males also exist and it

is caused by translocation of Y material including sex determining region (SRY)

to the X chromosome during paternal meiosis. Formal cytogenetic analysis is

necessary to make a definite diagnosis, and more obvious differences in physical

features tend to be associated with increasing numbers of sex chromosomes. If the

diagnosis is not made prenatally, 47,XXY males may present with a variety of

subtle clinical signs that are age-related. In infancy, males with 47,XXY may

have chromosomal evaluations done for hypospadias, small phallus or

cryptorchidism, developmental delay. The school-aged child may present with

language delay, learning disabilities, or behavioral problems. The older child or

adolescent may be discovered during an endocrine evaluation for delayed or

incomplete pubertal development with eunuchoid body habitus, gynecomastia, and

small testes. Adults are often evaluated for infertility or breast malignancy.

Androgen replacement therapy should begin at puberty, around age 12 years, in

increasing dosage sufficient to maintain age appropriate serum concentrations of

testosterone, estradiol, follicle stimulating hormone (FSH), and luteinizing

hormone (LH). The effects on physical and cognitive development increase with the

number of extra Xs, and each extra X is associated with an intelligence quotient

(IQ) decrease of approximately 15-16 points, with language most affected,

particularly expressive language skills.

FAU - Visootsak, Jeannie

AU - Visootsak J

AD - Department of Human Genetics, Emory University School of Medicine, Atlanta, GA

30033, USA. Jvisootsak@genetics.emory.edu

FAU - Graham, John M Jr

AU - Graham JM Jr

LA - eng

PT - Journal Article

PT - Review

DEP - 20061024

TA - Orphanet J Rare Dis

JT - Orphanet journal of rare diseases

JID - 101266602

SB - IM

MH - Adult

MH - Age Factors

MH - *Aneuploidy

MH - Child

MH - Chromosomes, Human, X/*genetics

MH - Humans

MH - Karyotyping

MH - Klinefelter Syndrome/drug therapy/*genetics/*pathology

MH - Male

MH - *Sex Chromosome Aberrations

PMC - PMC1634840

EDAT- 2006/10/26 09:00

MHDA- 2007/10/03 09:00

CRDT- 2006/10/26 09:00

PHST- 2006/09/15 00:00 [received]

PHST- 2006/10/24 00:00 [accepted]

PHST- 2006/10/26 09:00 [pubmed]

PHST- 2007/10/03 09:00 [medline]

PHST- 2006/10/26 09:00 [entrez]

AID - 1750-1172-1-42 [pii]

AID - 10.1186/1750-1172-1-42 [doi]

PST - epublish

SO - Orphanet J Rare Dis. 2006 Oct 24;1:42. doi: 10.1186/1750-1172-1-42.

PMID- 17415352

OWN - NLM

STAT- MEDLINE

DCOM- 20070501

LR - 20220408

IS - 1743-4289 (Electronic)

IS - 1743-4270 (Linking)

VI - 4

IP - 4

DP - 2007 Apr

TI - Klinefelter syndrome in clinical practice.

PG - 192-204

AB - Klinefelter syndrome is the most common sex-chromosome disorder; it affects

approximately one in every 660 men. This syndrome is characterized by the

presence of one or more extra X chromosomes, and the karyotype 47,XXY is the most

prevalent type. The 'prototypic' man with Klinefelter syndrome has traditionally

been described as tall, with narrow shoulders, broad hips, sparse body hair,

gynecomastia, small testicles, androgen deficiency, azoospermia and decreased

verbal intelligence. A less distinct phenotype has, however, been described.

Klinefelter syndrome is an underdiagnosed condition; only 25% of the expected

number of patients are diagnosed, and of these only a minority are diagnosed

before puberty. Patients with Klinefelter syndrome should be treated with

lifelong testosterone supplementation that begins at puberty, to secure proper

masculine development of sexual characteristics, muscle bulk and bone structure,

and to prevent the long-term deleterious consequences of hypogonadism; however,

the optimal testosterone regimen for patients with Klinefelter syndrome remains

to be established.

FAU - Bojesen, Anders

AU - Bojesen A

AD - Department of Clinical Genetics, Vejle Hospital, Aarhus University Hospital,

Denmark. anders.bojesen@dadlnet.dk

FAU - Gravholt, Claus H

AU - Gravholt CH

LA - eng

PT - Journal Article

PT - Review

PL - United States

TA - Nat Clin Pract Urol

JT - Nature clinical practice. Urology

JID - 101226508

RN - 3XMK78S47O (Testosterone)

SB - IM

MH - Alleles

MH - *Chromosomes, Human, X

MH - Congenital Abnormalities/diagnosis/epidemiology

MH - Drug Administration Schedule

MH - Follow-Up Studies

MH - *Gene Silencing

MH - Gynecomastia/genetics/physiopathology

MH - Humans

MH - Infertility, Male/genetics/physiopathology

MH - Klinefelter Syndrome/diagnosis/*drug therapy/*epidemiology/genetics

MH - Long-Term Care

MH - Male

MH - Prevalence

MH - Risk Assessment

MH - Testis/physiopathology

MH - Testosterone/*therapeutic use

MH - Treatment Outcome

RF - 75

EDAT- 2007/04/07 09:00

MHDA- 2007/05/02 09:00

CRDT- 2007/04/07 09:00

PHST- 2006/11/20 00:00 [received]

PHST- 2007/01/12 00:00 [accepted]

PHST- 2007/04/07 09:00 [pubmed]

PHST- 2007/05/02 09:00 [medline]

PHST- 2007/04/07 09:00 [entrez]

AID - ncpuro0775 [pii]

AID - 10.1038/ncpuro0775 [doi]

PST - ppublish

SO - Nat Clin Pract Urol. 2007 Apr;4(4):192-204. doi: 10.1038/ncpuro0775.

PMID- 33333453

OWN - NLM

STAT- Publisher

LR - 20210202

IS - 1876-7753 (Electronic)

IS - 1873-5061 (Linking)

VI - 50

DP - 2020 Dec 10

TI - Generation of an iPSC cohort of isogenic iPSC lines (46-XY and 47-XXY) from a

non-mosaic Klinefelter Syndrome patient (47-XXY) (KAUSTi008-A, KAUSTi008-B,

KAUSTi008-C, KAUSTi008-D, KAUSTi008-E, KAUSTi008-F, KAUSTi008-G).

PG - 102119

LID - S1873-5061(20)30420-7 [pii]

LID - 10.1016/j.scr.2020.102119 [doi]

AB - Klinefelter Syndrome (KS) is the most common X chromosome aneuploidy in males

characterized by highly heterogeneous clinical manifestations including a subtle

cognitive impairment and multisystemic disorders such as infertility, metabolic

syndrome, gynecomastia and cardiovascular diseases. To date dosage-dependent

correlation studies of X-linked genes and low- and high-grade KS clinical

phenotypes have not been performed. Here we generated multiple isogenic 47-XXY

and 46-XY iPSC lines from one 47-XXY patient. Leveraging on a fully matched

genetic background, our cohort represents a highly informative tool to study the

impact of X chromosome dosage on KS pathophysiology.

CI - Copyright © 2020 The Author(s). Published by Elsevier B.V. All rights reserved.

FAU - Fiacco, Elisabetta

AU - Fiacco E

AD - Biological and Environmental Science and Engineering Division, King Abdullah

University of Science and Technology, Thuwal 23955 6900, Saudi Arabia.

FAU - Alowaysi, Maryam

AU - Alowaysi M

AD - Biological and Environmental Science and Engineering Division, King Abdullah

University of Science and Technology, Thuwal 23955 6900, Saudi Arabia.

FAU - Astro, Veronica

AU - Astro V

AD - Biological and Environmental Science and Engineering Division, King Abdullah

University of Science and Technology, Thuwal 23955 6900, Saudi Arabia.

FAU - Adamo, Antonio

AU - Adamo A

AD - Biological and Environmental Science and Engineering Division, King Abdullah

University of Science and Technology, Thuwal 23955 6900, Saudi Arabia. Electronic

address: antonio.adamo@kaust.edu.sa.

LA - eng

PT - Journal Article

DEP - 20201210

PL - England

TA - Stem Cell Res

JT - Stem cell research

JID - 101316957

SB - IM

EDAT- 2020/12/18 06:00

MHDA- 2020/12/18 06:00

CRDT- 2020/12/17 20:15

PHST- 2020/11/16 00:00 [received]

PHST- 2020/11/29 00:00 [revised]

PHST- 2020/12/06 00:00 [accepted]

PHST- 2020/12/18 06:00 [pubmed]

PHST- 2020/12/18 06:00 [medline]

PHST- 2020/12/17 20:15 [entrez]

AID - S1873-5061(20)30420-7 [pii]

AID - 10.1016/j.scr.2020.102119 [doi]

PST - aheadofprint

SO - Stem Cell Res. 2020 Dec 10;50:102119. doi: 10.1016/j.scr.2020.102119.

PMID- 30127341

OWN - NLM

STAT- MEDLINE

DCOM- 20190211

LR - 20220417

IS - 1740-634X (Electronic)

IS - 0893-133X (Print)

IS - 0893-133X (Linking)

VI - 44

IP - 1

DP - 2019 Jan

TI - Sex differences in psychiatric disorders: what we can learn from sex chromosome

aneuploidies.

PG - 9-21

LID - 10.1038/s41386-018-0153-2 [doi]

AB - The study of sexual dimorphism in psychiatric and neurodevelopmental disorders is

challenging due to the complex interplay of diverse biological, psychological,

and social factors. Males are more susceptible to neurodevelopmental disorders

including intellectual disability, autism spectrum disorder, and

attention-deficit activity disorder. Conversely, after puberty, females are more

prone to major depressive disorder and anxiety disorders compared to males. One

major biological factor contributing to sex differences is the sex chromosomes.

First, the X and Y chromosomes have unique and specific genetic effects as well

as downstream gonadal effects. Second, males have one X chromosome and one Y

chromosome, while females have two X chromosomes. Thus, sex chromosome

constitution also differs between the sexes. Due to this complexity, determining

genetic and downstream biological influences on sexual dimorphism in humans is

challenging. Sex chromosome aneuploidies, such as Turner syndrome (X0) and

Klinefelter syndrome (XXY), are common genetic conditions in humans. The study of

individuals with sex chromosome aneuploidies provides a promising framework for

studying sexual dimorphism in neurodevelopmental and psychiatric disorders. Here

we will review and contrast four syndromes caused by variation in the number of

sex chromosomes: Turner syndrome, Klinefelter syndrome, XYY syndrome, and XXX

syndrome. Overall we describe an increased rate of attention-deficit

hyperactivity disorder and autism spectrum disorder, along with the increased

rates of major depressive disorder and anxiety disorders in one or more of these

conditions. In addition to contributing unique insights about sexual dimorphism

in neuropsychiatric disorders, awareness of the increased risk of

neurodevelopmental and psychiatric disorders in sex chromosome aneuploidies can

inform appropriate management of these common genetic disorders.

FAU - Green, Tamar

AU - Green T

AD - Center for Interdisciplinary Brain Sciences Research, Stanford University,

Stanford, CA, 94305, USA. tgreen2@stanford.edu.

AD - Department of Psychiatry and Behavioral Sciences, Stanford University, Stanford,

CA, 94305, USA. tgreen2@stanford.edu.

FAU - Flash, Shira

AU - Flash S

AD - Center for Interdisciplinary Brain Sciences Research, Stanford University,

Stanford, CA, 94305, USA.

FAU - Reiss, Allan L

AU - Reiss AL

AD - Center for Interdisciplinary Brain Sciences Research, Stanford University,

Stanford, CA, 94305, USA.

AD - Department of Psychiatry and Behavioral Sciences, Stanford University, Stanford,

CA, 94305, USA.

AD - Department of Radiology, Stanford University, Stanford, CA, 94305, USA.

LA - eng

GR - K23 HD090209/HD/NICHD NIH HHS/United States

GR - R01 HD049653/HD/NICHD NIH HHS/United States

GR - R21 MH099630/MH/NIMH NIH HHS/United States

PT - Journal Article

PT - Research Support, N.I.H., Extramural

PT - Research Support, Non-U.S. Gov't

PT - Review

DEP - 20180716

TA - Neuropsychopharmacology

JT - Neuropsychopharmacology : official publication of the American College of

Neuropsychopharmacology

JID - 8904907

RN - 47, XYY syndrome

RN - Triple X syndrome

SB - IM

MH - Chromosomes, Human, X/genetics

MH - Female

MH - Humans

MH - Klinefelter Syndrome/*genetics/psychology

MH - Male

MH - Mental Disorders/*genetics/psychology

MH - *Sex Characteristics

MH - Sex Chromosome Aberrations

MH - Sex Chromosome Disorders/*genetics/psychology

MH - Sex Chromosome Disorders of Sex Development/*genetics/psychology

MH - *Sex Chromosomes

MH - Trisomy/*genetics

MH - Turner Syndrome/*genetics/psychology

MH - XYY Karyotype/*genetics/psychology

PMC - PMC6235860

COIS- The authors declare no competing interests.

EDAT- 2018/08/22 06:00

MHDA- 2019/02/12 06:00

CRDT- 2018/08/22 06:00

PHST- 2018/04/07 00:00 [received]

PHST- 2018/06/21 00:00 [accepted]

PHST- 2018/06/01 00:00 [revised]

PHST- 2018/08/22 06:00 [pubmed]

PHST- 2019/02/12 06:00 [medline]

PHST- 2018/08/22 06:00 [entrez]

AID - 10.1038/s41386-018-0153-2 [pii]

AID - 153 [pii]

AID - 10.1038/s41386-018-0153-2 [doi]

PST - ppublish

SO - Neuropsychopharmacology. 2019 Jan;44(1):9-21. doi: 10.1038/s41386-018-0153-2.

Epub 2018 Jul 16.

PMID- 20569798

OWN - NLM

STAT- MEDLINE

DCOM- 20100709

LR - 20220408

IS - 1558-318X (Electronic)

IS - 0094-0143 (Linking)

VI - 37

IP - 2

DP - 2010 May

TI - A practical approach to ambiguous genitalia in the newborn period.

PG - 195-205

LID - 10.1016/j.ucl.2010.03.014 [doi]

AB - The evaluation and management of neonates with ambiguous genitalia requires

sensitivity, efficiency, and accuracy. The approach to these neonates is

facilitated by a multidisciplinary team including urology, endocrinology,

genetics, and psychiatry or psychology. Disorders of sex development (DSD)

encompass chromosomal DSD, 46,XX DSD, and 46,XY DSD. The 46,XX DSD is the most

common DSD and in the majority of these children congenital adrenal hyperplasia

is the underlying etiology. The 46,XY DSD is a heterogeneous disorder that often

results from a disruption in the production or response to testosterone,

dihydrotestosterone, or Mullerian inhibitory substance. Chromosomal DSD includes

conditions resulting from abnormal meiosis, including Klinefelter syndrome (47,

XXY) and Turner syndrome. The evaluation of children with DSD demands a thorough

physical examination, medical history, karyotype, metabolic panel, 17-OH

progesterone, testosterone, luteinizing hormone, follicle stimulation hormone,

and urinalysis. A radiographic evaluation should begin with an abdominal and

pelvic ultrasound but may include magnetic resonance imaging, endoscopy, or

laparoscopy.

FAU - Lambert, Sarah M

AU - Lambert SM

AD - Children's Hospital of Philadelphia, University of Pennsylvania School of

Medicine, Philadelphia, PA 19104, USA.

FAU - Vilain, Eric J N

AU - Vilain EJ

FAU - Kolon, Thomas F

AU - Kolon TF

LA - eng

PT - Journal Article

PT - Review

PL - United States

TA - Urol Clin North Am

JT - The Urologic clinics of North America

JID - 0423221

SB - IM

MH - Adrenal Hyperplasia, Congenital/diagnosis

MH - Disorders of Sex Development/*diagnosis

MH - Female

MH - Genitalia/abnormalities

MH - Humans

MH - Infant, Newborn

MH - Male

RF - 57

EDAT- 2010/06/24 06:00

MHDA- 2010/07/10 06:00

CRDT- 2010/06/24 06:00

PHST- 2010/06/24 06:00 [entrez]

PHST- 2010/06/24 06:00 [pubmed]

PHST- 2010/07/10 06:00 [medline]

AID - S0094-0143(10)00020-0 [pii]

AID - 10.1016/j.ucl.2010.03.014 [doi]

PST - ppublish

SO - Urol Clin North Am. 2010 May;37(2):195-205. doi: 10.1016/j.ucl.2010.03.014.

PMID- 21429590

OWN - NLM

STAT- MEDLINE

DCOM- 20111011

LR - 20110816

IS - 0165-1781 (Print)

IS - 0165-1781 (Linking)

VI - 189

IP - 1

DP - 2011 Aug 30

TI - Deficits in inhibitory executive functions in Klinefelter (47, XXY) syndrome.

PG - 135-40

LID - 10.1016/j.psychres.2011.02.028 [doi]

AB - Klinefelter syndrome (47, XXY) is a sex chromosome aneuploidy associated with

mild deficits in cognitive and language functions. Dysfunctions have also been

reported in performance of tasks which examine executive functions. However, it

is unclear whether the impaired performance is caused or accentuated by problems

with semantic processing and information processing speed. In the present study

we used an experimental task which is relatively insensitive to these confounding

factors. We examined inhibitory executive functions in a group of XXY males

compared with male (XY) and female (XX) controls, using a dichotic listening

speech sound task with instructions to focus attention on either the right or the

left ear stimulus. With this task, inhibitory executive functions can be assessed

separately from language, processing speed, and attention orientation abilities.

We found that XXY males showed a selective deficit in inhibitory executive

functions compared to both control groups, whereas attentional orientation was

not impaired. The present findings suggest that executive dysfunctions associated

to Klinefelter syndrome can be selectively identified, and are particularly

accentuated in the inhibitory sub-component. Such improved understanding of the

nature of executive dysfunctions in XXY males may aid the development of specific

neuropsychological rehabilitation strategies.

CI - Copyright © 2011 Elsevier Ltd. All rights reserved.

FAU - Kompus, Kristiina

AU - Kompus K

AD - Department of Biological and Medical Psychology, University of Bergen, Norway.

kristiina.kompus@psybp.uib.no

FAU - Westerhausen, René

AU - Westerhausen R

FAU - Nilsson, Lars-Göran

AU - Nilsson LG

FAU - Hugdahl, Kenneth

AU - Hugdahl K

FAU - Jongstra, Susan

AU - Jongstra S

FAU - Berglund, Alexander

AU - Berglund A

FAU - Arver, Stefan

AU - Arver S

FAU - Savic, Ivanka

AU - Savic I

LA - eng

PT - Journal Article

PT - Research Support, Non-U.S. Gov't

DEP - 20110322

PL - Ireland

TA - Psychiatry Res

JT - Psychiatry research

JID - 7911385

SB - IM

MH - Adult

MH - Analysis of Variance

MH - Attention Deficit Disorder with Hyperactivity/etiology

MH - Chromosomes, Human, X

MH - Cognition Disorders/*etiology

MH - Executive Function/*physiology

MH - Female

MH - Humans

MH - Klinefelter Syndrome/*complications

MH - Male

MH - Middle Aged

MH - Neuropsychological Tests

MH - Psychiatric Status Rating Scales

MH - Young Adult

EDAT- 2011/03/25 06:00

MHDA- 2011/10/12 06:00

CRDT- 2011/03/25 06:00

PHST- 2010/10/18 00:00 [received]

PHST- 2011/01/31 00:00 [revised]

PHST- 2011/02/25 00:00 [accepted]

PHST- 2011/03/25 06:00 [entrez]

PHST- 2011/03/25 06:00 [pubmed]

PHST- 2011/10/12 06:00 [medline]

AID - S0165-1781(11)00174-0 [pii]

AID - 10.1016/j.psychres.2011.02.028 [doi]

PST - ppublish

SO - Psychiatry Res. 2011 Aug 30;189(1):135-40. doi: 10.1016/j.psychres.2011.02.028.

Epub 2011 Mar 22.

PMID- 32415904

OWN - NLM

STAT- MEDLINE

DCOM- 20210505

LR - 20210505

IS - 1552-4876 (Electronic)

IS - 1552-4868 (Linking)

VI - 184

IP - 2

DP - 2020 Jun

TI - Neuropsychological functions, sleep, and mental health in adults with Klinefelter

syndrome.

PG - 482-492

LID - 10.1002/ajmg.c.31797 [doi]

AB - A few studies have examined neuropsychological functions, sleep, and mental

health combined in Klinefelter syndrome (KS; 47,XXY). We investigated

neuropsychological functions with standard tests, sleep with actigraphy, and

self-reported mental health in 30 men with KS (Mean age = 36.7 years) compared to

21 controls (Mean age = 36.8 years). Men with KS scored significantly lower on

mental speed, attention span, working memory, inhibition, and set-shifting tests,

as well as overall IQ (mean effect size difference Cohen's d = 0.79). Men with KS

had significantly longer night wakes, with no differences in other sleep

variables (mean d = 0.34). Men with KS reported poorer mental health than

controls (mean d = 1.16). Regression analyses showed neuropsychological functions

explained variance in some sleep domains for men with KS but not for controls.

Neuropsychological functions explained variance in some mental health domains for

controls. For men with KS, however, verbal IQ was the only significant predictor

of mental health. Altogether, men with KS display problems in neuropsychological

functions and mental health but do not appear different from controls on most

sleep parameters. Our findings indicate that relations between neuropsychological

functions, sleep, and mental health differ between men with KS and controls.

CI - © 2020 The Authors. American Journal of Medical Genetics Part C: Seminars in

Medical Genetics published by Wiley Periodicals, Inc.

FAU - Fjermestad, Krister W

AU - Fjermestad KW

AUID- ORCID: 0000-0001-7501-0033

AD - Department of Psychology, University of Oslo, Oslo, Norway.

AD - Frambu Resource Centre for Rare Disorders, Siggerud, Norway.

FAU - Huster, Rene

AU - Huster R

AD - Department of Psychology, University of Oslo, Oslo, Norway.

FAU - Thunberg, Christina

AU - Thunberg C

AD - Department of Psychology, University of Oslo, Oslo, Norway.

FAU - Stokke, Simen

AU - Stokke S

AD - Frambu Resource Centre for Rare Disorders, Siggerud, Norway.

FAU - Gravholt, Claus H

AU - Gravholt CH

AD - Department of Clinical Medicine, Århus University, Aarhus, Denmark.

FAU - Solbakk, Anne-Kristin

AU - Solbakk AK

AD - Department of Psychology, University of Oslo, Oslo, Norway.

AD - Department of Neurosurgery, Oslo University Hospital, Oslo, Norway.

AD - Department of Neuropsychology, Helgeland Hospital, Mosjøen, Norway.

AD - RITMO Centre for Interdisciplinary Studies in Rhythm, Time and Motion, University

of Oslo, Oslo, Norway.

LA - eng

PT - Journal Article

PT - Research Support, Non-U.S. Gov't

DEP - 20200516

PL - United States

TA - Am J Med Genet C Semin Med Genet

JT - American journal of medical genetics. Part C, Seminars in medical genetics

JID - 101235745

SB - IM

MH - Adult

MH - Attention/physiology

MH - Cognition Disorders/*physiopathology

MH - Humans

MH - Klinefelter Syndrome/*physiopathology

MH - Male

MH - *Mental Health

MH - Middle Aged

MH - Neuropsychological Tests

MH - Sleep/*physiology

OTO - NOTNLM

OT - *47,XXY

OT - *Klinefelter syndrome

OT - *executive functions

OT - *mental health

OT - *sleep

EDAT- 2020/05/18 06:00

MHDA- 2021/05/06 06:00

CRDT- 2020/05/17 06:00

PHST- 2020/04/14 00:00 [received]

PHST- 2020/04/28 00:00 [revised]

PHST- 2020/04/29 00:00 [accepted]

PHST- 2020/05/18 06:00 [pubmed]

PHST- 2021/05/06 06:00 [medline]

PHST- 2020/05/17 06:00 [entrez]

AID - 10.1002/ajmg.c.31797 [doi]

PST - ppublish

SO - Am J Med Genet C Semin Med Genet. 2020 Jun;184(2):482-492. doi:

10.1002/ajmg.c.31797. Epub 2020 May 16.

PMID- 32662248

OWN - NLM

STAT- MEDLINE

DCOM- 20220303

LR - 20220303

IS - 1552-4833 (Electronic)

IS - 1552-4825 (Linking)

VI - 185

IP - 12

DP - 2021 Dec

TI - Neurocognitive development and capabilities in boys with 49,XXXXY syndrome.

PG - 3541-3546

LID - 10.1002/ajmg.a.61736 [doi]

AB - 49,XXXXY was previously associated with profound to severe intellectual deficits.

However, prior research papers on the cognitive profiles of this population were

confounded by small samples sizes, wide age spreads, and incomplete histories of

testosterone replacement therapy. This study is the first comprehensive,

international investigation of the neurocognitive aspects of 49,XXXXY, and the

potential effects of biological treatment on this profile. Sixty-seven boys from

infancy to 11 years of age were enrolled in this longitudinal study, with the

majority of boys postnatally diagnosed though chromosomal analysis. These boys

received a comprehensive neurocognitive evaluation tailored to specific

language-based deficits and cognitive challenges. Results revealed higher

neurocognitive capacities, both verbally and nonverbally, than previously

reported in this disorder. Infant boys with 49,XXXXY who received early hormonal

therapy (EHT) had significantly higher scores on the cognitive domain of the

Bayley Scales of Infant Development than untreated infants (p = .013). In

addition, treated school-aged participants had significantly better scaled scores

than untreated boys in form completion (p = .042), a task that requires deductive

reasoning, on nonverbal testing on the Leiter International Performance Scales.

This study indicates greater cognitive capacities with a wide range of abilities

in the child with 49,XXXXY, thus warranting further investigation to identify and

understand the critical influences on the etiology and the variability of those

capacities.

CI - © 2020 Wiley Periodicals LLC.

FAU - Gropman, Andrea L

AU - Gropman AL

AD - Division of Neurogenetics and Developments Pediatrics, Children's National Health

System, Washington, District of Columbia, USA.

AD - Department of Neurology, George Washington University, Washington, District of

Columbia, USA.

FAU - Porter, Grace F

AU - Porter GF

AD - Research Department, The Focus Foundation, Davidsonville, Maryland, USA.

FAU - Lasutschinkow, Patricia C

AU - Lasutschinkow PC

AUID- ORCID: 0000-0003-0258-1534

AD - Research Department, The Focus Foundation, Davidsonville, Maryland, USA.

FAU - Sadeghin, Teresa

AU - Sadeghin T

AD - Research Department, The Focus Foundation, Davidsonville, Maryland, USA.

FAU - Tipton, Elizabeth S

AU - Tipton ES

AD - Research Department, The Focus Foundation, Davidsonville, Maryland, USA.

FAU - Powell, Sherida

AU - Powell S

AD - Department of Economics, George Washington University, Washington, District of

Columbia, USA.

FAU - Samango-Sprouse, Carole A

AU - Samango-Sprouse CA

AUID- ORCID: 0000-0001-9941-0568

AD - Research Department, The Focus Foundation, Davidsonville, Maryland, USA.

AD - Department of Pediatrics, George Washington University, Washington, District of

Columbia, USA.

AD - Department of Human and Molecular Genetics, Florida International University,

Miami, Florida, USA.

LA - eng

PT - Journal Article

PT - Research Support, Non-U.S. Gov't

DEP - 20200714

PL - United States

TA - Am J Med Genet A

JT - American journal of medical genetics. Part A

JID - 101235741

SB - IM

MH - Aneuploidy

MH - Child

MH - Child, Preschool

MH - Chromosomes, Human, X/genetics

MH - Chromosomes, Human, Y/genetics

MH - Cognition Disorders/complications/*drug therapy/genetics/physiopathology

MH - Hormone Replacement Therapy

MH - Humans

MH - Infant

MH - Infant, Newborn

MH - Klinefelter Syndrome/complications/*drug therapy/genetics/physiopathology

MH - Language Development Disorders/complications/*drug

therapy/genetics/physiopathology

MH - Longitudinal Studies

MH - Male

MH - Neurocognitive Disorders/complications/*drug therapy/genetics/physiopathology

OTO - NOTNLM

OT - *49,XXXXY

OT - *X and Y chromosomal variation

OT - *sex chromosome aneuploidies

OT - *variant of 47,XXY (Klinefelter syndrome)

EDAT- 2020/07/15 06:00

MHDA- 2022/03/04 06:00

CRDT- 2020/07/15 06:00

PHST- 2019/12/12 00:00 [received]

PHST- 2020/06/05 00:00 [accepted]

PHST- 2020/07/15 06:00 [pubmed]

PHST- 2022/03/04 06:00 [medline]

PHST- 2020/07/15 06:00 [entrez]

AID - 10.1002/ajmg.a.61736 [doi]

PST - ppublish

SO - Am J Med Genet A. 2021 Dec;185(12):3541-3546. doi: 10.1002/ajmg.a.61736. Epub

2020 Jul 14.

PMID- 31577063

OWN - NLM

STAT- MEDLINE

DCOM- 20200817

LR - 20200930

IS - 1552-4833 (Electronic)

IS - 1552-4825 (Linking)

VI - 179

IP - 12

DP - 2019 Dec

TI - Hormonal replacement therapy and its potential influence on working memory and

competency/adaptive functioning in 47,XXY (Klinefelter syndrome).

PG - 2374-2381

LID - 10.1002/ajmg.a.61360 [doi]

AB - This cross-sectional, retrospective analysis investigated the possible effect of

hormonal replacement therapy (HRT) on working memory (WM) and competency/adaptive

functioning (CAF) in boys with 47,XXY; the effect of timing of 47,XXY diagnosis

on these variables; and the relationship between WM and CAF, if any. A total of

111 boys with 47,XXY, ranging from 6 to 16 years of age (M = 9 years 4 months; SD

= 2 years 1 month), were evaluated using the Wechsler Intelligence Scale for

Children, and Child Behavior Checklist. Participants were grouped by HRT status

and timing of diagnosis. Analysis of variance testing performed on the prenatally

diagnosed boys revealed a statistically significant difference in WM for the HRT

groups (F[3,84] = 7.467, p = .000174), where WM of the no-HRT group (M = 92.37,

SD = 17.83) was lower than that of the early hormonal therapy group (M = 106.39,

SD = 12.01; p = .0092). Additionally, there was a positive correlation between

low WM capabilities and poor school performance (r = .5106, p = .0027) in the

prenatally diagnosed, untreated boys. Our results highlight the potentially

positive effects of HRT on WM and CAF in boys with 47,XXY. Further research is

required to better determine the underlying relationship among the biological

mechanisms of HRT, WM, and CAF outcomes, and timing of diagnosis in boys with

47,XXY.

CI - © 2019 Wiley Periodicals, Inc.

FAU - Tran, Selena L

AU - Tran SL

AD - The Focus Foundation, Davidsonville, Maryland.

FAU - Samango-Sprouse, Carole A

AU - Samango-Sprouse CA

AUID- ORCID: 0000-0001-9941-0568

AD - The Focus Foundation, Davidsonville, Maryland.

AD - Department of Human and Molecular Genetics, Florida International University,

Miami, Florida.

AD - Department of Pediatrics, George Washington University, Washington, District of

Columbia.

FAU - Sadeghin, Teresa

AU - Sadeghin T

AD - The Focus Foundation, Davidsonville, Maryland.

FAU - Powell, Sherida

AU - Powell S

AD - Department of Economics, George Washington University, Washington, District of

Columbia.

FAU - Gropman, Andrea L

AU - Gropman AL

AUID- ORCID: 0000-0002-2106-6776

AD - Department of Neurology, George Washington University, Washington, District of

Columbia.

AD - Division of Neurogenetics and Developmental Pediatrics, Children's National

Health System, Washington, District of Columbia.

LA - eng

PT - Journal Article

DEP - 20191002

PL - United States

TA - Am J Med Genet A

JT - American journal of medical genetics. Part A

JID - 101235741

RN - 3XMK78S47O (Testosterone)

SB - IM

MH - *Adaptation, Physiological

MH - Adolescent

MH - Child

MH - *Hormone Replacement Therapy

MH - Humans

MH - Klinefelter Syndrome/*diagnosis/*drug therapy/*genetics

MH - Male

MH - *Memory, Short-Term

MH - Social Class

MH - Testosterone/therapeutic use

OTO - NOTNLM

OT - *47,XXY

OT - *adaptive functioning

OT - *competency

OT - *early hormonal therapy

OT - *working memory

EDAT- 2019/10/03 06:00

MHDA- 2020/08/18 06:00

CRDT- 2019/10/03 06:00

PHST- 2019/05/15 00:00 [received]

PHST- 2019/08/12 00:00 [revised]

PHST- 2019/09/05 00:00 [accepted]

PHST- 2019/10/03 06:00 [pubmed]

PHST- 2020/08/18 06:00 [medline]

PHST- 2019/10/03 06:00 [entrez]

AID - 10.1002/ajmg.a.61360 [doi]

PST - ppublish

SO - Am J Med Genet A. 2019 Dec;179(12):2374-2381. doi: 10.1002/ajmg.a.61360. Epub

2019 Oct 2.

PMID- 32685420

OWN - NLM

STAT- PubMed-not-MEDLINE

LR - 20220415

IS - 2228-5482 (Print)

IS - 2251-676X (Electronic)

IS - 2228-5482 (Linking)

VI - 21

IP - 3

DP - 2020 Jul-Sep

TI - Rare Disorder of Sexual Differentiation with a Mosaic 46,XX/47,XXY in a

Klinefelter Syndrome Individual.

PG - 222-224

AB - BACKGROUND: Klinefelter syndrome (KS) mosaicism 46,XX/47,XXY is an extremely rare

disorder of sex development characterized by the presence of both ovarian and

testicular tissues in the same individual. Both elements can be present in the

same gonad (ovotestis) or separately in the same individual or as a unilateral

ovotestis and the other side with testis or ovary. A mosaic with 46,XY would

present with problems related to male infertility and in general, testicular

insufficiency, but with a 46,XX mosaic, it is a completely rare presentation. As

adolescents, these boys may experience severe emotional and behavioral issues; it

is up to the parents to identify these conditions early and get them physician

evaluated for possible abnormalities so that they can get the benefit of

treatment. CASE PRESENTATION: A case of a rare disorder of sexual differentiation

with a mosaic 46,XX/47,XXY in a KS individual is reported for whom karyotyping

and SRY-FISH work-up was done. CONCLUSION: Early cytogenetic testing is essential

to identify these individuals and testosterone replacement therapy and breast

reduction for case management are helpful. Assisted reproductive technology (ART)

may assist these individuals father children in some cases.

CI - Copyright© 2020, Avicenna Research Institute.

FAU - Pattamshetty, Preethi

AU - Pattamshetty P

AD - Department of Genetics and Molecular Medicine, Vasavi Medical and Research

Centre, Hyderabad, India.

FAU - Mantri, Harika

AU - Mantri H

AD - Department of Genetics and Molecular Medicine, Vasavi Medical and Research

Centre, Hyderabad, India.

FAU - Mohan, Vasavi

AU - Mohan V

AD - Department of Genetics and Molecular Medicine, Vasavi Medical and Research

Centre, Hyderabad, India.

AD - Department of Genetics, Apollo Health and lifestyle Ltd, Hyderabad, India.

LA - eng

PT - Case Reports

TA - J Reprod Infertil

JT - Journal of reproduction & infertility

JID - 101535586

PMC - PMC7362088

OTO - NOTNLM

OT - Counseling

OT - Karyotyping

OT - Klinefelter syndrome

OT - Mosaicism

OT - Sex determining region Y (SRY gene)

COIS- Conflict of Interest The authors declare no conflict of interest.

EDAT- 2020/07/21 06:00

MHDA- 2020/07/21 06:01

CRDT- 2020/07/21 06:00

PHST- 2020/07/21 06:00 [entrez]

PHST- 2020/07/21 06:00 [pubmed]

PHST- 2020/07/21 06:01 [medline]

AID - JRI-21-222 [pii]

PST - ppublish

SO - J Reprod Infertil. 2020 Jul-Sep;21(3):222-224.

PMID- 23504510

OWN - NLM

STAT- MEDLINE

DCOM- 20130506

LR - 20220408

IS - 1479-683X (Electronic)

IS - 0804-4643 (Linking)

VI - 168

IP - 4

DP - 2013 Apr

TI - Testicular function and fertility in men with Klinefelter syndrome: a review.

PG - R67-76

LID - 10.1530/EJE-12-0934 [doi]

AB - Klinefelter syndrome, 47,XXY (KS), is the most frequent sex chromosome aberration

in males, affecting 1 in 660 newborn boys. The syndrome is characterized by

testicular destruction with extensive fibrosis and hyalinization of the

seminiferous tubules resulting in small testes, hypergonadotropic hypogonadism,

and azoospermia in the majority of cases. Until recently, infertility was

considered an untreatable condition in KS. However, with the development of new

advanced assisted reproductive techniques such as testicular sperm extraction

(TESE) combined with ICSI it seems that KS patients should no longer be labelled

as infertile. Especially, microdissection (micro)-TESE has proved to be an

advantageous procedure for the identification of testicular spermatozoa in KS.

The aim of this review was to describe current knowledge on the testicular

changes occurring in KS, the associated changes in reproductive hormones and

spermatogenesis, and the existing possibilities of biological fatherhood in

47,XXY patients.

FAU - Aksglaede, L

AU - Aksglaede L

AD - Department of Growth and Reproduction, Rigshospitalet, Section 5064, University

of Copenhagen, Copenhagen, Denmark. lise.aksglaede@rh.regionh.dk

FAU - Juul, A

AU - Juul A

LA - eng

PT - Journal Article

PT - Review

DEP - 20130315

PL - England

TA - Eur J Endocrinol

JT - European journal of endocrinology

JID - 9423848

SB - IM

MH - Animals

MH - Fertility/*physiology

MH - Humans

MH - Klinefelter Syndrome/*pathology/physiopathology

MH - Male

MH - Spermatozoa/pathology/physiology

MH - Testis/pathology/*physiology

EDAT- 2013/03/19 06:00

MHDA- 2013/05/07 06:00

CRDT- 2013/03/19 06:00

PHST- 2013/03/19 06:00 [entrez]

PHST- 2013/03/19 06:00 [pubmed]

PHST- 2013/05/07 06:00 [medline]

AID - 168/4/R67 [pii]

AID - 10.1530/EJE-12-0934 [doi]

PST - epublish

SO - Eur J Endocrinol. 2013 Mar 15;168(4):R67-76. doi: 10.1530/EJE-12-0934. Print 2013

Apr.

PMID- 23335129

OWN - NLM

STAT- MEDLINE

DCOM- 20130520

LR - 20130916

IS - 1552-4876 (Electronic)

IS - 1552-4868 (Linking)

VI - 163C

IP - 1

DP - 2013 Feb 15

TI - Neurocognitive variance and neurological underpinnings of the X and Y chromosomal

variations.

PG - 35-43

LID - 10.1002/ajmg.c.31352 [doi]

AB - X and Y chromosomal variations including tetrasomy and pentasomy conditions are

rare and occur in 1:18,000-1:100,000 male births. The most common sex chromosome

aneuploidy is 47, XXY for which there is a rich literature delineating the

physical and neurobehavioral phenotype. Although the more complex chromosome

aneuploidies 48, XXYY, 48, XXXY, and 49, XXXXY are often compared with 47, XXY

(Klinefelter syndrome) because of shared features including tall stature and

hypergonadotropic hypogonadism, there is a wider spectrum of physical and

cognitive abilities that have recently been delineated. The phenotypic

presentation of the boys with more severe aneuploidy shares some characteristics

with 47, XXY, but there are also other unique and distinctive features.

Previously unappreciated intact nonverbal skills have been demonstrated in

association with severe developmental dyspraxia. MRI findings of white matter

hyperintensities may underlie cognitive deficits and deserve further study. This

report discusses what is known about clinical variability in the XY syndromes

collectively evaluated through careful multidisciplinary clinical evaluation

including the clinical and neurobehavioral aspects of these conditions.

Variability in clinical and cognitive functioning may reflect skewed X

inactivation, mosaicism, or epigenetic factors that warrant further

investigation.

CI - Copyright © 2013 Wiley Periodicals, Inc.

FAU - Gropman, Andrea

AU - Gropman A

AD - Division of Neurogenetics and Neurodevelopmental Pediatrics, Children's National

Medical Center, Washington, DC 2001, USA. agropman@childrensnational.org

FAU - Samango-Sprouse, Carole A

AU - Samango-Sprouse CA

LA - eng

PT - Journal Article

PT - Review

DEP - 20130118

PL - United States

TA - Am J Med Genet C Semin Med Genet

JT - American journal of medical genetics. Part C, Seminars in medical genetics

JID - 101235745

SB - IM

MH - *Aneuploidy

MH - *Chromosomes, Human, X

MH - *Chromosomes, Human, Y

MH - Cognition Disorders/*genetics/physiopathology

MH - Genetic Variation/*genetics

MH - Humans

MH - Male

MH - Nervous System Diseases/*genetics/physiopathology

MH - Syndrome

EDAT- 2013/01/22 06:00

MHDA- 2013/05/22 06:00

CRDT- 2013/01/22 06:00

PHST- 2013/01/22 06:00 [entrez]

PHST- 2013/01/22 06:00 [pubmed]

PHST- 2013/05/22 06:00 [medline]

AID - 10.1002/ajmg.c.31352 [doi]

PST - ppublish

SO - Am J Med Genet C Semin Med Genet. 2013 Feb 15;163C(1):35-43. doi:

10.1002/ajmg.c.31352. Epub 2013 Jan 18.

PMID- 29680294

OWN - NLM

STAT- MEDLINE

DCOM- 20190215

LR - 20190215

IS - 2050-0521 (Electronic)

IS - 2050-0521 (Linking)

VI - 6

IP - 4

DP - 2018 Oct

TI - Klinefelter Syndrome. The Effects of Early Androgen Therapy on Competence and

Behavioral Phenotype.

PG - 595-606

LID - S2050-0521(18)30042-8 [pii]

LID - 10.1016/j.sxmr.2018.02.008 [doi]

AB - INTRODUCTION: Klinefelter syndrome (KS) is the result of sex chromosome

aneuploidy most often characterized as 47,XXY. The typical features of KS include

tall stature, gynecomastia, small firm testicles, hypergonadotropic hypogonadism,

and infertility. However, abnormalities in neurodevelopment, cognition, and

social and behavioral functioning also can be present. The abnormalities in

neurodevelopment are believed to be due in part to androgen deficiency during

early development and puberty. AIM: To discuss the role of androgens in normal

adolescent development; discuss the cognitive, behavioral, and social functioning

of children with KS; evaluate the evidence for early androgen therapy in men with

KS; and discuss management strategies in the development of boys with KS.

METHODS: A systematic review of early androgen therapy and KS was performed using

PubMed-Medline and Scopus databases. Relevant articles commenting on social,

behavioral, cognitive, and physical outcomes among infants, children, and

adolescents were included for reporting and discussion. MAIN OUTCOME MEASURES:

Social and behavior functioning; cognitive outcomes; adverse effects associated

with androgen therapy. RESULTS: 3 retrospective articles and 2 randomized

controlled trials addressing early androgen therapy in boys with KS were

reviewed. These studies showed an improvement in several aspects of social and

cognitive functioning based on validated questionnaires. Treatment strategies,

potential negative effects, and limitations of the literature on early androgen

therapy in boys with KS are discussed. CONCLUSION: Our findings indicate that

early androgen supplementation in children with KS combined with specific

educational, family, and social support improves behavioral functioning. The

optimal timing of hormonal therapy might require prospective studies, but based

on our data and review of the literature, the benefit of early hormonal and

therapeutic intervention in KS is very encouraging. Flannigan R, Patel P, Paduch

DA. Klinefelter Syndrome. The Effects of Early Androgen Therapy on Competence and

Behavioral Phenotype. Sex Med Rev 2018;6:595-606.

CI - Copyright © 2018 International Society for Sexual Medicine. Published by Elsevier

Inc. All rights reserved.

FAU - Flannigan, Ryan

AU - Flannigan R

AD - Department of Urology, Weill Cornell Medicine, New York, NY, USA.

FAU - Patel, Premal

AU - Patel P

AD - Division of Urology, Department of Surgery, University of Manitoba, Winnipeg, MB,

Canada.

FAU - Paduch, Darius A

AU - Paduch DA

AD - Department of Urology, Weill Cornell Medicine, New York, NY, USA; Consulting

Research Services, Inc, North Bergen, NJ, USA. Electronic address:

dap2003@med.cornell.edu.

LA - eng

PT - Journal Article

PT - Research Support, Non-U.S. Gov't

PT - Systematic Review

DEP - 20180419

PL - Netherlands

TA - Sex Med Rev

JT - Sexual medicine reviews

JID - 101614773

RN - 0 (Androgens)

SB - IM

MH - Androgens/*therapeutic use

MH - Gynecomastia

MH - Humans

MH - *Klinefelter Syndrome/diagnosis/drug therapy/physiopathology

MH - Male

MH - Phenotype

MH - Psychomotor Performance

MH - Social Behavior

MH - Treatment Outcome

OTO - NOTNLM

OT - *47,XXY

OT - *Androgen

OT - *Behavioral Phenotype

OT - *Klinefelter Syndrome

OT - *Neurodevelopment

OT - *Testosterone

EDAT- 2018/04/24 06:00

MHDA- 2019/02/16 06:00

CRDT- 2018/04/23 06:00

PHST- 2017/10/17 00:00 [received]

PHST- 2018/01/21 00:00 [revised]

PHST- 2018/02/02 00:00 [accepted]

PHST- 2018/04/24 06:00 [pubmed]

PHST- 2019/02/16 06:00 [medline]

PHST- 2018/04/23 06:00 [entrez]

AID - S2050-0521(18)30042-8 [pii]

AID - 10.1016/j.sxmr.2018.02.008 [doi]

PST - ppublish

SO - Sex Med Rev. 2018 Oct;6(4):595-606. doi: 10.1016/j.sxmr.2018.02.008. Epub 2018

Apr 19.

PMID- 29466784

OWN - NLM

STAT- MEDLINE

DCOM- 20180405

LR - 20180405

IS - 1424-859X (Electronic)

IS - 1424-8581 (Linking)

VI - 153

IP - 4

DP - 2017

TI - Clinical, Hormonal, and Genetic Evaluation of Idiopathic Nonobstructive

Azoospermia and Klinefelter Syndrome Patients.

PG - 190-197

LID - 10.1159/000487039 [doi]

AB - To investigate the clinical, hormonal, and genetic factors in infertile men with

idiopathic nonobstructive azoospermia (NOA) or azoospermic Klinefelter syndrome

(KFS), a total of 556 and 96 patients, respectively, were included in this study.

All patient samples were analyzed cytogenetically. Serum reproductive hormone

levels were measured. Microdeletions in the azoospermia factor (AZF) region of

the Y chromosome were detected by multiplex PCR using 16 specific sequence-tagged

sites. FSH and LH levels in both NOA and KFS patients were significantly higher

than the normal range, and the testosterone level in KFS patients was

significantly lower. Ninety-two (95.8%) of the KFS patients showed non-mosaic

47,XXY karyotypes and 47,XXY,inv(9)(p11.1q13); the other KFS patients had mosaic

karyotypes of 47,XXY/46,XY, 47,XXY/46,XX, and 47,XXY/48,XXXY/46,XX. Among the 556

idiopathic NOA patients with normal karyotypes, 67 (12.05%) had microdeletions in

the AZF region of the Y chromosome. Microdeletions were most frequently detected

in the AZFc region, followed by AZFa, AZFb, AZFbc, and partial AZFc deletions.

However, Y chromosome microdeletions were not found in any of the azoospermic KFS

patients. In view of the hormonal and genetic abnormalities in infertile men with

idiopathic NOA and with azoospermic KFS, genetic testing for karyotype, Y

chromosome microdeletions, and hormonal parameters is advocated.

CI - © 2018 S. Karger AG, Basel.

FAU - Kim, Shin Y

AU - Kim SY

AD - Laboratory of Medical Genetics, Medical Research Institute, Dankook University

College of Medicine, Seoul, Korea.

FAU - Lee, Bom Y

AU - Lee BY

FAU - Oh, Ah R

AU - Oh AR

FAU - Park, So Y

AU - Park SY

FAU - Lee, Hyo S

AU - Lee HS

FAU - Seo, Ju T

AU - Seo JT

LA - eng

PT - Journal Article

DEP - 20180222

PL - Switzerland

TA - Cytogenet Genome Res

JT - Cytogenetic and genome research

JID - 101142708

RN - 0 (Gonadal Steroid Hormones)

RN - 0 (Gonadotropins, Pituitary)

SB - IM

MH - Abnormal Karyotype

MH - Adult

MH - Aged

MH - Aneuploidy

MH - Azoospermia/blood/*genetics/pathology

MH - Chromosomes, Human, Y/genetics/ultrastructure

MH - Gonadal Steroid Hormones/*blood

MH - Gonadotropins, Pituitary/*blood

MH - Humans

MH - Infertility, Male/etiology

MH - Karyotyping

MH - Klinefelter Syndrome/blood/*genetics/pathology

MH - Male

MH - Middle Aged

MH - Mosaicism

MH - Organ Size

MH - Semen Analysis

MH - Sequence Deletion

MH - Testis/pathology

MH - Young Adult

OTO - NOTNLM

OT - Idiopathic nonobstructive azoospermia

OT - Karyotype

OT - Klinefelter syndrome

OT - Reproductive hormones

OT - Y chromosome microdeletions

EDAT- 2018/02/22 06:00

MHDA- 2018/04/06 06:00

CRDT- 2018/02/22 06:00

PHST- 2017/11/09 00:00 [accepted]

PHST- 2018/02/22 06:00 [pubmed]

PHST- 2018/04/06 06:00 [medline]

PHST- 2018/02/22 06:00 [entrez]

AID - 000487039 [pii]

AID - 10.1159/000487039 [doi]

PST - ppublish

SO - Cytogenet Genome Res. 2017;153(4):190-197. doi: 10.1159/000487039. Epub 2018 Feb

22.

PMID- 23322622

OWN - NLM

STAT- MEDLINE

DCOM- 20130701

LR - 20211021

IS - 1552-4833 (Electronic)

IS - 1552-4825 (Print)

IS - 1552-4825 (Linking)

VI - 161A

IP - 2

DP - 2013 Feb

TI - Timing of diagnosis of 47,XXY and 48,XXYY: a survey of parent experiences.

PG - 268-72

LID - 10.1002/ajmg.a.35709 [doi]

AB - 47,XXY/Klinefelter syndrome is the most common sex chromosomal aneuploidy, yet

64% of males with this condition go undiagnosed. 48,XXYY is less common and there

is less known about the diagnosis. The objective of this study is to describe the

diagnosis experiences of parents of males with 47,XXY and 48,XXYY. Parents of 89

males with 47,XXY and 76 males with 48,XXYY completed a survey that gathered data

about their experiences leading to a diagnosis, including the current age of the

child, age at diagnosis, reasons for initial concern, and the specialists

providing the diagnosis. In the 47,XXY cohort diagnosed postnatally, 59%

presented with developmental delay, with a mean age at first parental concern of

5.2 years and mean age of diagnosis at 10.0 years. The remaining 41% presented

with endocrinologic issues with a mean age at first concern of 19.1 years and

mean age of diagnosis at 21.1 years. In the 48,XXYY group, 93% presented with

developmental delay, with mean age at first parental concern of 2.4 years and

mean age of diagnosis at 7.6 years. Hence, the average time from initial parental

concern to diagnosis of 47,XXY or 48,XXYY ranges from 2 to 5 years, with those

presenting with developmental issues having a longer lag to diagnosis compared to

those presenting with endocrinologic issues. Increased awareness of the

developmental, psychological, and medical features of 47,XXY and 48,XXYY is

important to facilitate timely diagnosis and initiation of appropriate screenings

and treatments that are important for optimal outcomes.

CI - Copyright © 2013 Wiley Periodicals, Inc.

FAU - Visootsak, Jeannie

AU - Visootsak J

AD - Department of Human Genetics, Emory University, Atlanta, Georgia 30033, USA.

Jvisoot@emory.edu

FAU - Ayari, Natalie

AU - Ayari N

FAU - Howell, Susan

AU - Howell S

FAU - Lazarus, Joash

AU - Lazarus J

FAU - Tartaglia, Nicole

AU - Tartaglia N

LA - eng

GR - K23 NS070337/NS/NINDS NIH HHS/United States

GR - UL1 RR025780/RR/NCRR NIH HHS/United States

GR - K23 HD058043/HD/NICHD NIH HHS/United States

GR - 1K23HD058043-01A1/HD/NICHD NIH HHS/United States

GR - 1K23NS070337-01A1/NS/NINDS NIH HHS/United States

GR - UL1 TR000454/TR/NCATS NIH HHS/United States

GR - UL1RR025780/RR/NCRR NIH HHS/United States

GR - UL1 TR001082/TR/NCATS NIH HHS/United States

PT - Journal Article

PT - Research Support, N.I.H., Extramural

PT - Research Support, Non-U.S. Gov't

DEP - 20130115

TA - Am J Med Genet A

JT - American journal of medical genetics. Part A

JID - 101235741

SB - IM

MH - Adolescent

MH - Adult

MH - Child

MH - Child, Preschool

MH - Data Collection

MH - Developmental Disabilities/*diagnosis/genetics

MH - Genetic Testing

MH - Humans

MH - Infant

MH - Infant, Newborn

MH - Klinefelter Syndrome/*diagnosis/genetics

MH - Male

MH - Middle Aged

MH - Parents/*psychology

MH - Time Factors

MH - Young Adult

PMC - PMC3558746

MID - NIHMS409561

EDAT- 2013/01/17 06:00

MHDA- 2013/07/03 06:00

CRDT- 2013/01/17 06:00

PHST- 2012/03/20 00:00 [received]

PHST- 2012/09/10 00:00 [accepted]

PHST- 2013/01/17 06:00 [entrez]

PHST- 2013/01/17 06:00 [pubmed]

PHST- 2013/07/03 06:00 [medline]

AID - 10.1002/ajmg.a.35709 [doi]

PST - ppublish

SO - Am J Med Genet A. 2013 Feb;161A(2):268-72. doi: 10.1002/ajmg.a.35709. Epub 2013

Jan 15.

PMID- 30036387

OWN - NLM

STAT- MEDLINE

DCOM- 20190130

LR - 20190130

IS - 1932-6203 (Electronic)

IS - 1932-6203 (Linking)

VI - 13

IP - 7

DP - 2018

TI - Salivary testosterone in relation to social cognition and social anxiety in

children and adolescents with 47,XXY (Klinefelter syndrome).

PG - e0200882

LID - 10.1371/journal.pone.0200882 [doi]

LID - e0200882

AB - BACKGROUND: Approximately 1 in 650 boys are born with an extra X chromosome. Boys

and men with 47,XXY (Klinefelter syndrome) are at risk for neurodevelopmental

disorders and specific cognitive impairments. This study was focused on social

anxiety and social cognition. The aim was to assess if these aspects of the

phenotype are related to testosterone deficiency, which is typically seen in

47,XXY from puberty onwards. METHODS: In the study 20 boys with 47,XXY and 25

non-clinical controls between 8 and 19 years participated. None had ever used

testosterone supplements. Cognitive tests measuring the labeling of facial

expressions and perspective taking (Theory of Mind) were administered.

Self-report questionnaires were used to assess social anxiety. Testosterone was

measured in saliva. RESULTS: Within the 47,XXY group lower levels of salivary

testosterone were significantly associated with higher levels of social anxiety.

The correlation was strong, andindependent of age and pubertal development.

However, salivary levels of testosterone were uncorrelated to social cognitive

skills. DISCUSSION: These findings point out that lower testosterone levels might

contribute to high social anxiety in 47,XXY, suggesting that anxiety should be

monitored in pubertal boys with XXY presenting with testosterone deficiency. This

should be done in addition to exploring cognitive behavioral therapy or

psychopharmacologic treatments targeting anxiety, which are more evidence based.

In contrast, testosterone levels were not associated with social cognitive

functioning, suggesting that other mechanisms are driving vulnerabilities in this

domain.

FAU - van Rijn, Sophie

AU - van Rijn S

AUID- ORCID: 0000-0002-9179-7515

AD - Leiden University, Clinical Child and Adolescent Studies, Leiden, The

Netherlands.

AD - Leiden Institute for Brain and Cognition, Leiden, The Netherlands.

LA - eng

PT - Journal Article

PT - Research Support, Non-U.S. Gov't

DEP - 20180723

TA - PLoS One

JT - PloS one

JID - 101285081

RN - 3XMK78S47O (Testosterone)

SB - IM

MH - Adolescent

MH - *Anxiety

MH - Child

MH - *Cognition

MH - Cognition Disorders/complications

MH - Facial Expression

MH - Humans

MH - Klinefelter Syndrome/*genetics

MH - Male

MH - Neuropsychological Tests

MH - Phenotype

MH - Saliva/*chemistry

MH - Sexual Maturation

MH - Social Behavior

MH - Social Skills

MH - Testosterone/*chemistry

MH - Young Adult

PMC - PMC6056033

COIS- The authors have declared that no competing interests exist.

EDAT- 2018/07/24 06:00

MHDA- 2019/01/31 06:00

CRDT- 2018/07/24 06:00

PHST- 2017/02/28 00:00 [received]

PHST- 2018/07/05 00:00 [accepted]

PHST- 2018/07/24 06:00 [entrez]

PHST- 2018/07/24 06:00 [pubmed]

PHST- 2019/01/31 06:00 [medline]

AID - PONE-D-17-08007 [pii]

AID - 10.1371/journal.pone.0200882 [doi]

PST - epublish

SO - PLoS One. 2018 Jul 23;13(7):e0200882. doi: 10.1371/journal.pone.0200882.

eCollection 2018.

PMID- 30637954

OWN - NLM

STAT- MEDLINE

DCOM- 20200416

LR - 20200930

IS - 1552-4833 (Electronic)

IS - 1552-4825 (Linking)

VI - 179

IP - 3

DP - 2019 Mar

TI - The incidence of anxiety symptoms in boys with 47,XXY (Klinefelter syndrome) and

the possible impact of timing of diagnosis and hormonal replacement therapy.

PG - 423-428

LID - 10.1002/ajmg.a.61038 [doi]

AB - 47,XXY (Klinefelter syndrome) is the most common X and Y chromosomal variation

(1:660 males). The incidence of anxiety disorders and the impact of hormonal

replacement therapy (HRT) is not well understood. Child Behavior Checklist and

Screen for Childhood Anxiety Related Emotional Disorders were completed by

parents of 80 boys with 47,XXY. Forty received HRT prior to 10 years of age while

40 did not. HRT (22.5%) received early hormonal treatment prior to 18 months.

About 32.5% received hormone booster treatment between 5 and 10 years. The

remaining 42.5% received both. There were fewer reported social (p = .015),

thought (p = .012), and affective problems (p = .048) in treated boys when

compared to untreated. Boys with both treatments demonstrated fewer symptoms on

anxious/depressed scale (p = .001) compared to those with early treatment only.

Within the treated group, prenatally diagnosed showed fewer indications of

anxiety problems (p = .02) than their postnatal counterparts. This comparative,

cross-sectional study expands previous findings on the possible positive effect

of HRT in boys with 47,XXY. Anxiety disorders appear to be a penetrant aspect of

the 47,XXY phenotype. Further investigation is warranted to explore the

relationship between biological treatment and individual responses to HRT to

develop more personalized and precise medicine.

CI - © 2019 Wiley Periodicals, Inc.

FAU - Samango-Sprouse, Carole

AU - Samango-Sprouse C

AUID- ORCID: 0000-0001-9941-0568

AD - George Washington University, Department of Pediatrics, Washington, DC.

AD - Children's National Health System, Neurodevelopmental Pediatrics and

Neurogenetics, Washington, DC.

AD - Florida International University, Department of Human and Molecular Genetics,

Miami, Florida.

AD - The Focus Foundation, Davidsonville, Maryland.

FAU - Lasutschinkow, Patricia

AU - Lasutschinkow P

AD - The Focus Foundation, Davidsonville, Maryland.

FAU - Powell, Sherida

AU - Powell S

AD - George Washington University, Department of Pediatrics, Washington, DC.

FAU - Sadeghin, Teresa

AU - Sadeghin T

AD - The Focus Foundation, Davidsonville, Maryland.

FAU - Gropman, Andrea

AU - Gropman A

AD - George Washington University, Department of Pediatrics, Washington, DC.

AD - Children's National Health System, Neurodevelopmental Pediatrics and

Neurogenetics, Washington, DC.

LA - eng

GR - The Focus Foundation/International

PT - Journal Article

PT - Research Support, Non-U.S. Gov't

DEP - 20190113

PL - United States

TA - Am J Med Genet A

JT - American journal of medical genetics. Part A

JID - 101235741

SB - IM

MH - Adolescent

MH - Anxiety/*epidemiology/etiology

MH - Child

MH - Child, Preschool

MH - Cross-Sectional Studies

MH - Disease Management

MH - Hormone Replacement Therapy

MH - Humans

MH - Incidence

MH - Infant

MH - Klinefelter Syndrome/diagnosis/*epidemiology/*psychology/therapy

MH - Male

MH - Patient Education as Topic

MH - Phenotype

MH - Time-to-Treatment

OTO - NOTNLM

OT - *47,XXY

OT - *Klinefelter syndrome

OT - *X and Y chromosomal variations

OT - *anxiety disorders

OT - *hormonal replacement therapy

OT - *sex chromosome abnormalities and aneuploidies

EDAT- 2019/01/15 06:00

MHDA- 2020/04/17 06:00

CRDT- 2019/01/15 06:00

PHST- 2018/05/02 00:00 [received]

PHST- 2018/12/10 00:00 [revised]

PHST- 2018/12/14 00:00 [accepted]

PHST- 2019/01/15 06:00 [pubmed]

PHST- 2020/04/17 06:00 [medline]

PHST- 2019/01/15 06:00 [entrez]

AID - 10.1002/ajmg.a.61038 [doi]

PST - ppublish

SO - Am J Med Genet A. 2019 Mar;179(3):423-428. doi: 10.1002/ajmg.a.61038. Epub 2019

Jan 13.

PMID- 32432413

OWN - NLM

STAT- MEDLINE

DCOM- 20210505

LR - 20210524

IS - 1552-4876 (Electronic)

IS - 1552-4868 (Print)

IS - 1552-4868 (Linking)

VI - 184

IP - 2

DP - 2020 Jun

TI - The behavioral profile of children aged 1-5 years with sex chromosome trisomy

(47,XXX, 47,XXY, 47,XYY).

PG - 444-455

LID - 10.1002/ajmg.c.31788 [doi]

AB - Children with SCT have an increased risk of suboptimal neurodevelopment. Previous

studies have shown an elevated risk for neurobehavioral problems in individuals

with SCT. However, not much is known about neurobehavioral problems in very young

children; knowledge that could help with early identification of children at risk

for suboptimal development, and that could help establish targets for early

intervention. This study addressed the question of what the behavioral profile of

children with SCT aged 1-5 years looks like. In total, 182 children aged

1-5 years participated in this study (N(SCT) =87, N(nonclinical controls) = 95).

Recruitment and assessment took place in the Netherlands and the United States.

The SCT group was recruited through prospective follow-up (50%), information

seeking parents (31%), and clinical referral (18%). Behavioral profiles were

assessed with the child behavior checklist and the ages-and-stages

social-emotional questionnaire. Levels of parent-rated problem behavior were

higher in children with SCT. Difficulties with overall social-emotional

functioning were already present in 1-year-olds, and elevated scores were

persistent across the full age range. Affective and pervasive developmental

behaviors were seen in late toddlerhood and prominent at preschool age. Anxiety,

attention deficit, and oppositional defiant behaviors were seen in preschool-aged

children. Within this cross-sectional study, the developmental trajectory of

affective, pervasive developmental, and oppositional defiant behaviors seemed to

be different for SCT children than nonclinical controls. Collectively, these

results demonstrate the importance of behavioral screening for behavioral

problems in routine clinical care for children with SCT from a young age.

Social-emotional problems may require special attention, as these problems seem

most prominent, showing increased risk across the full age range, and with these

problems occurring regardless of the timing of diagnosis, and across all three

SCT karyotypes.

CI - © 2020 The Authors. American Journal of Medical Genetics Part C: Seminars in

Medical Genetics published by Wiley Periodicals, Inc.

FAU - Urbanus, Evelien

AU - Urbanus E

AUID- ORCID: 0000-0002-4706-9086

AD - Clinical Neurodevelopmental Sciences, Leiden University, Leiden, The Netherlands.

AD - Leiden Institute for Brain and Cognition, Leiden, The Netherlands.

FAU - Swaab, Hanna

AU - Swaab H

AD - Clinical Neurodevelopmental Sciences, Leiden University, Leiden, The Netherlands.

AD - Leiden Institute for Brain and Cognition, Leiden, The Netherlands.

FAU - Tartaglia, Nicole

AU - Tartaglia N

AUID- ORCID: 0000-0002-8529-6722

AD - eXtraordinarY Kids Clinic, Developmental Pediatrics, Children's Hospital

Colorado, Aurora, Colorado, USA.

AD - Department of Pediatrics, University of Colorado School of Medicine, Aurora,

Colorado, USA.

FAU - Cordeiro, Lisa

AU - Cordeiro L

AD - eXtraordinarY Kids Clinic, Developmental Pediatrics, Children's Hospital

Colorado, Aurora, Colorado, USA.

AD - Department of Pediatrics, University of Colorado School of Medicine, Aurora,

Colorado, USA.

FAU - van Rijn, Sophie

AU - van Rijn S

AD - Clinical Neurodevelopmental Sciences, Leiden University, Leiden, The Netherlands.

AD - Leiden Institute for Brain and Cognition, Leiden, The Netherlands.

LA - eng

GR - R01 HD091251/HD/NICHD NIH HHS/United States

GR - UL1 TR002535/TR/NCATS NIH HHS/United States

PT - Journal Article

PT - Research Support, N.I.H., Extramural

PT - Research Support, Non-U.S. Gov't

DEP - 20200520

TA - Am J Med Genet C Semin Med Genet

JT - American journal of medical genetics. Part C, Seminars in medical genetics

JID - 101235745

RN - 47, XYY syndrome

RN - Triple X syndrome

SB - IM

MH - Anxiety/diagnosis/genetics/physiopathology

MH - Attention Deficit Disorder with Hyperactivity/diagnosis/genetics/*physiopathology

MH - Child

MH - Child, Preschool

MH - Chromosomes, Human, X/genetics

MH - Female

MH - Humans

MH - Infant

MH - Male

MH - *Problem Behavior

MH - Sex Chromosome Aberrations

MH - Sex Chromosome Disorders/*diagnosis/genetics/physiopathology

MH - Sex Chromosome Disorders of Sex Development/diagnosis/genetics/physiopathology

MH - Sex Chromosomes/genetics

MH - Trisomy/diagnosis/genetics/*physiopathology

MH - XYY Karyotype/diagnosis/genetics/physiopathology

PMC - PMC7384033

OTO - NOTNLM

OT - *behavioral development

OT - *behavioral problems

OT - *developmental impact

OT - *psychopathology

OT - *sex chromosome trisomy

COIS- The authors declare no conflicts of interest.

EDAT- 2020/05/21 06:00

MHDA- 2021/05/06 06:00

CRDT- 2020/05/21 06:00

PHST- 2020/02/14 00:00 [received]

PHST- 2020/03/27 00:00 [revised]

PHST- 2020/04/09 00:00 [accepted]

PHST- 2020/05/21 06:00 [pubmed]

PHST- 2021/05/06 06:00 [medline]

PHST- 2020/05/21 06:00 [entrez]

AID - AJMGC31788 [pii]

AID - 10.1002/ajmg.c.31788 [doi]

PST - ppublish

SO - Am J Med Genet C Semin Med Genet. 2020 Jun;184(2):444-455. doi:

10.1002/ajmg.c.31788. Epub 2020 May 20.

PMID- 30689602

OWN - NLM

STAT- MEDLINE

DCOM- 20200306

LR - 20200309

IS - 1473-6578 (Electronic)

IS - 0951-7367 (Print)

IS - 0951-7367 (Linking)

VI - 32

IP - 2

DP - 2019 Mar

TI - A review of neurocognitive functioning and risk for psychopathology in sex

chromosome trisomy (47,XXY, 47,XXX, 47, XYY).

PG - 79-84

LID - 10.1097/YCO.0000000000000471 [doi]

AB - PURPOSE OF REVIEW: About one in 650-1000 children is born with an extra X or Y

chromosome, referred to as sex chromosome trisomies (SCTs). Studying SCTs may

uncover unique insights in neurodevelopmental pathways underlying the risk for

neurobehavioral problems and psychopathology. There is also a clinical need for

more knowledge about the phenotype of SCT with the recent introduction of

noninvasive prenatal screening. RECENT FINDINGS: The reviewed studies illustrate

an increased vulnerability for psychopathology such as (symptoms of) autism

spectrum disorder, attention-deficit/hyperactivity disorder, anxiety, depression

and, to a lesser degree, psychotic disorders. Although traditionally the primary

focus has been on language and learning problems, recent research suggests that

impairments in executive functioning, social cognition and emotion regulation may

also be key factors underlying the risk for neurobehavioral problems. SUMMARY:

The research field of SCT is in need of a more longitudinal perspective to

identify early markers of 'at risk' development, and to assess the effectiveness

of early interventions. Neurocognitive markers that signal compromised

neurodevelopment may prove to be helpful in this. Variability in the SCT

phenotype provides a unique opportunity to identify not only genetic but also

environmental factors that shape neurodevelopmental outcome, calling for studies

focused on understanding individual differences.

FAU - van Rijn, Sophie

AU - van Rijn S

AD - Leiden University, Clinical Neurodevelopmental Sciences.

AD - Leiden Institute for Brain and Cognition, Leiden, the Netherlands.

LA - eng

PT - Journal Article

PT - Research Support, Non-U.S. Gov't

PT - Review

TA - Curr Opin Psychiatry

JT - Current opinion in psychiatry

JID - 8809880

SB - IM

MH - Early Intervention, Educational

MH - Executive Function

MH - Humans

MH - *Neurocognitive Disorders/etiology/psychology/therapy

MH - Psychopathology

MH - Self-Control

MH - *Sex Chromosome Disorders/physiopathology/psychology

MH - *Sex Chromosomes

MH - Social Behavior

MH - *Trisomy

PMC - PMC6687415

EDAT- 2019/01/29 06:00

MHDA- 2020/03/07 06:00

CRDT- 2019/01/29 06:00

PHST- 2019/01/29 06:00 [entrez]

PHST- 2019/01/29 06:00 [pubmed]

PHST- 2020/03/07 06:00 [medline]

AID - 00001504-201903000-00007 [pii]

AID - YCO320204 [pii]

AID - 10.1097/YCO.0000000000000471 [doi]

PST - ppublish

SO - Curr Opin Psychiatry. 2019 Mar;32(2):79-84. doi: 10.1097/YCO.0000000000000471.

PMID- 20014370

OWN - NLM

STAT- MEDLINE

DCOM- 20100319

LR - 20211020

IS - 1940-5529 (Electronic)

IS - 1940-5510 (Print)

IS - 1940-5529 (Linking)

VI - 15

IP - 4

DP - 2009

TI - Structural and functional neuroimaging in Klinefelter (47,XXY) syndrome: a review

of the literature and preliminary results from a functional magnetic resonance

imaging study of language.

PG - 295-308

LID - 10.1002/ddrr.84 [doi]

AB - Klinefelter (47,XXY) syndrome (KS), the most common form of sex-chromosomal

aneuploidy, is characterized by physical, endocrinologic, and reproductive

abnormalities. Individuals with KS also exhibit a cognitive/behavioral phenotype

characterized by language and language-based learning disabilities and executive

and attentional dysfunction in the setting of normal general intelligence. The

underlying neurobiologic mechanisms are just now beginning to be elucidated

through structural and functional neuroimaging. Here, we review the literature of

structural and functional neural findings in KS identified by neuroimaging and

present preliminary results from a functional magnetic resonance imaging study

examining brain activity during a verb generation task in KS.

FAU - Steinman, Kyle

AU - Steinman K

AD - Division of Child Neurology, Department of Neurology, University of

California-San Francisco, 350 Parnassus Ave, Suite 609, San Francisco, CA 94117,

USA. steinman@neuropeds.ucsf.edu

FAU - Ross, Judith

AU - Ross J

FAU - Lai, Song

AU - Lai S

FAU - Reiss, Allan

AU - Reiss A

FAU - Hoeft, Fumiko

AU - Hoeft F

LA - eng

GR - 5K23HD054720/HD/NICHD NIH HHS/United States

GR - K23 HD054720/HD/NICHD NIH HHS/United States

GR - K12 NS01692/NS/NINDS NIH HHS/United States

GR - K12 NS001692/NS/NINDS NIH HHS/United States

GR - R01 NS050597/NS/NINDS NIH HHS/United States

GR - R01 NS050597-03S1/NS/NINDS NIH HHS/United States

GR - R01 NS050597-01A2/NS/NINDS NIH HHS/United States

GR - NS050597/NS/NINDS NIH HHS/United States

PT - Journal Article

PT - Research Support, N.I.H., Extramural

PT - Review

TA - Dev Disabil Res Rev

JT - Developmental disabilities research reviews

JID - 101319448

SB - IM

MH - Brain/*anatomy & histology

MH - Humans

MH - Klinefelter Syndrome/*diagnosis/*epidemiology

MH - Language Disorders/diagnosis/*epidemiology

MH - Learning Disabilities/epidemiology

MH - *Magnetic Resonance Imaging

MH - Verbal Learning

PMC - PMC2876340

MID - NIHMS201355

COIS- Conflicts of Interest: The authors have no conflicts of interest to report.

EDAT- 2009/12/17 06:00

MHDA- 2010/03/20 06:00

CRDT- 2009/12/17 06:00

PHST- 2009/12/17 06:00 [entrez]

PHST- 2009/12/17 06:00 [pubmed]

PHST- 2010/03/20 06:00 [medline]

AID - 10.1002/ddrr.84 [doi]

PST - ppublish

SO - Dev Disabil Res Rev. 2009;15(4):295-308. doi: 10.1002/ddrr.84.

PMID- 28611019

OWN - NLM

STAT- MEDLINE

DCOM- 20171012

LR - 20190218

IS - 1479-683X (Electronic)

IS - 0804-4643 (Linking)

VI - 177

IP - 5

DP - 2017 Nov

TI - MECHANISMS IN ENDOCRINOLOGY: Aberrations of the X chromosome as cause of male

infertility.

PG - R249-R259

LID - EJE-17-0246 [pii]

LID - 10.1530/EJE-17-0246 [doi]

AB - Male infertility is most commonly caused by spermatogenetic failure, clinically

noted as oligo- or a-zoospermia. Today, in approximately 20% of azoospermic

patients, a causal genetic defect can be identified. The most frequent genetic

causes of azoospermia (or severe oligozoospermia) are Klinefelter syndrome

(47,XXY), structural chromosomal abnormalities and Y-chromosomal microdeletions.

Consistent with Ohno's law, the human X chromosome is the most stable of all the

chromosomes, but contrary to Ohno's law, the X chromosome is loaded with regions

of acquired, rapidly evolving genes, which are of special interest because they

are predominantly expressed in the testis. Therefore, it is not surprising that

the X chromosome, considered as the female counterpart of the male-associated Y

chromosome, may actually play an essential role in male infertility and sperm

production. This is supported by the recent description of a significantly

increased copy number variation (CNV) burden on both sex chromosomes in infertile

men and point mutations in X-chromosomal genes responsible for male infertility.

Thus, the X chromosome seems to be frequently affected in infertile male

patients. Four principal X-chromosomal aberrations have been identified so far:

(1) aneuploidy of the X chromosome as found in Klinefelter syndrome (47,XXY or

mosaicism for additional X chromosomes). (2) Translocations involving the X

chromosome, e.g. nonsyndromic 46,XX testicular disorders of sex development

(XX-male syndrome) or X-autosome translocations. (3) CNVs affecting the X

chromosome. (4) Point mutations disrupting X-chromosomal genes. All these are

reviewed herein and assessed concerning their importance for the clinical routine

diagnostic workup of the infertile male as well as their potential to shape

research on spermatogenic failure in the next years.

CI - © 2017 European Society of Endocrinology.

FAU - Röpke, Albrecht

AU - Röpke A

FAU - Tüttelmann, Frank

AU - Tüttelmann F

LA - eng

PT - Journal Article

PT - Review

DEP - 20170613

PL - England

TA - Eur J Endocrinol

JT - European journal of endocrinology

JID - 9423848

SB - IM

MH - Azoospermia/diagnosis/genetics/therapy

MH - Chromosomes, Human, X/*genetics

MH - Humans

MH - Infertility, Male/*diagnosis/*genetics/therapy

MH - Male

MH - Sperm Injections, Intracytoplasmic/methods

MH - Spermatogenesis/genetics

EDAT- 2017/06/15 06:00

MHDA- 2017/10/13 06:00

CRDT- 2017/06/15 06:00

PHST- 2017/03/24 00:00 [received]

PHST- 2017/05/22 00:00 [revised]

PHST- 2017/06/13 00:00 [accepted]

PHST- 2017/06/15 06:00 [pubmed]

PHST- 2017/10/13 06:00 [medline]

PHST- 2017/06/15 06:00 [entrez]

AID - EJE-17-0246 [pii]

AID - 10.1530/EJE-17-0246 [doi]

PST - ppublish

SO - Eur J Endocrinol. 2017 Nov;177(5):R249-R259. doi: 10.1530/EJE-17-0246. Epub 2017

Jun 13.

PMID- 33068889

OWN - NLM

STAT- MEDLINE

DCOM- 20210621

LR - 20210621

IS - 1876-7753 (Electronic)

IS - 1873-5061 (Linking)

VI - 49

DP - 2020 Dec

TI - Establishment of an iPSC cohort from three unrelated 47-XXY Klinefelter Syndrome

patients (KAUSTi007-A, KAUSTi007-B, KAUSTi009-A, KAUSTi009-B, KAUSTi010-A,

KAUSTi010-B).

PG - 102042

LID - S1873-5061(20)30343-3 [pii]

LID - 10.1016/j.scr.2020.102042 [doi]

AB - Klinefelter Syndrome (KS) is caused by the presence of a

supernumerary X chromosome. Cytogenetic studies revaled that 80-90% of patients

carry a 47-XXY karyotype, while 10-20% of cases are represented by mosaic

46-XY/47-XXY and high-grade aneuploidies 48-XXXY and 48-XXYY. The phenotypic

traits of KS are highly variable across individuals and include cognitive

dysfunction, metabolic dysregulation, osteoporosis, and cardiovascular diseases.

Here, we describe the derivation of multiple 47-XXY iPSC lines from three

unrelated KS patients to study the impact of supernumerary X chromosome during

early development.

CI - Copyright © 2020 The Author(s). Published by Elsevier B.V. All rights reserved.

FAU - Alowaysi, Maryam

AU - Alowaysi M

AD - Biological and Environmental Science and Engineering Division, King Abdullah

University of Science and Technology, Thuwal 23955-6900, Saudi Arabia.

FAU - Fiacco, Elisabetta

AU - Fiacco E

AD - Biological and Environmental Science and Engineering Division, King Abdullah

University of Science and Technology, Thuwal 23955-6900, Saudi Arabia.

FAU - Astro, Veronica

AU - Astro V

AD - Biological and Environmental Science and Engineering Division, King Abdullah

University of Science and Technology, Thuwal 23955-6900, Saudi Arabia.

FAU - Adamo, Antonio

AU - Adamo A

AD - Biological and Environmental Science and Engineering Division, King Abdullah

University of Science and Technology, Thuwal 23955-6900, Saudi Arabia. Electronic

address: antonio.adamo@kaust.edu.sa.

LA - eng

PT - Journal Article

PT - Research Support, Non-U.S. Gov't

DEP - 20201010

PL - England

TA - Stem Cell Res

JT - Stem cell research

JID - 101316957

SB - IM

MH - Humans

MH - *Induced Pluripotent Stem Cells

MH - Karyotyping

MH - *Klinefelter Syndrome/genetics

MH - Phenotype

MH - Sex Chromosome Aberrations

EDAT- 2020/10/18 06:00

MHDA- 2021/06/22 06:00

CRDT- 2020/10/17 20:11

PHST- 2020/09/28 00:00 [received]

PHST- 2020/10/06 00:00 [accepted]

PHST- 2020/10/18 06:00 [pubmed]

PHST- 2021/06/22 06:00 [medline]

PHST- 2020/10/17 20:11 [entrez]

AID - S1873-5061(20)30343-3 [pii]

AID - 10.1016/j.scr.2020.102042 [doi]

PST - ppublish

SO - Stem Cell Res. 2020 Dec;49:102042. doi: 10.1016/j.scr.2020.102042. Epub 2020 Oct

10.

PMID- 32004174

OWN - NLM

STAT- MEDLINE

DCOM- 20210610

LR - 20210610

IS - 1473-656X (Electronic)

IS - 1040-872X (Linking)

VI - 32

IP - 2

DP - 2020 Apr

TI - A review of the intriguing interaction between testosterone and neurocognitive

development in males with 47,XXY.

PG - 140-146

LID - 10.1097/GCO.0000000000000612 [doi]

AB - PURPOSE OF REVIEW: Although 47,XXY (Klinefelter syndrome) was first discovered

more than 50 years ago, there have been limited comprehensive studies on this

disorder. The present review explains the study of neurodevelopmental dysfunction

and the impact of testosterone replacement at specific junctions in the life of

males with 47,XXY. The intricate relationship between testosterone,

neurodevelopment, health, and well being warrants an in-depth investigation in

order to achieve optimal outcomes. RECENT FINDINGS: Current literature suggests

that the implementation of biological treatment has a positive impact on numerous

areas of neurodevelopment. Further research is needed to determine ideal dosage,

timing, and frequency of biological treatment for efficacy and safety of the

child with 47,XXY. SUMMARY: As noninvasive prenatal screening has detected

increasing numbers of fetuses with 47,XXY, parents may benefit from both prenatal

and postnatal counseling, including the latest innovative biological treatment,

that may further optimize the child's outcome, especially when coupled with

targeted early intervention services.

FAU - Samango-Sprouse, Carole A

AU - Samango-Sprouse CA

AD - Department of Pediatrics, George Washington University, Washington, District of

Columbia.

AD - Department of Human and Molecular Genetics, Florida International University,

Miami, Florida.

AD - The Focus Foundation, Davidsonville, Maryland.

FAU - Yu, Christine

AU - Yu C

AD - Departments of Pediatrics and Internal Medicine, Section of Adult and Pediatric

Endocrinology, Diabetes, & Metabolism, University of Chicago, Chicago, Illinois.

FAU - Porter, Grace F

AU - Porter GF

AD - The Focus Foundation, Davidsonville, Maryland.

FAU - Tipton, Elizabeth S

AU - Tipton ES

AD - The Focus Foundation, Davidsonville, Maryland.

FAU - Lasutschinkow, Patricia C

AU - Lasutschinkow PC

AD - The Focus Foundation, Davidsonville, Maryland.

FAU - Gropman, Andrea L

AU - Gropman AL

AD - Division of Neurogenetics and Developments Pediatrics, Children's National

Hospital.

AD - Department of Neurology, George Washington University, Washington, District of

Columbia.

LA - eng

PT - Journal Article

PT - Research Support, Non-U.S. Gov't

PT - Review

PL - England

TA - Curr Opin Obstet Gynecol

JT - Current opinion in obstetrics & gynecology

JID - 9007264

RN - 3XMK78S47O (Testosterone)

SB - IM

MH - Adolescent

MH - Child

MH - Child Development/*drug effects

MH - Child, Preschool

MH - Hormone Replacement Therapy/methods

MH - Humans

MH - Infant

MH - Klinefelter Syndrome/*therapy

MH - Male

MH - Testosterone/*administration & dosage/adverse effects/pharmacology

EDAT- 2020/02/01 06:00

MHDA- 2021/06/11 06:00

CRDT- 2020/02/01 06:00

PHST- 2020/02/01 06:00 [pubmed]

PHST- 2021/06/11 06:00 [medline]

PHST- 2020/02/01 06:00 [entrez]

AID - 00001703-202004000-00007 [pii]

AID - 10.1097/GCO.0000000000000612 [doi]

PST - ppublish

SO - Curr Opin Obstet Gynecol. 2020 Apr;32(2):140-146. doi:

10.1097/GCO.0000000000000612.

PMID- 27318449

OWN - NLM

STAT- MEDLINE

DCOM- 20170413

LR - 20170413

IS - 1872-7654 (Electronic)

IS - 0301-2115 (Linking)

VI - 203

DP - 2016 Aug

TI - The impact of prenatally diagnosed Klinefelter Syndrome on obstetric and neonatal

outcomes.

PG - 173-6

LID - S0301-2115(16)30220-2 [pii]

LID - 10.1016/j.ejogrb.2016.05.006 [doi]

AB - OBJECTIVE: The objective of this study was to examine the obstetric and neonatal

outcomes as well as the as the associated hospital costs for pregnancies

complicated by prenatally diagnosed Klinefelter Syndrome, 47,XXY. STUDY DESIGN:

We conducted a retrospective cohort study of all of the singleton deliveries in

California from 2005 to 2008 using vital statistics and ICD-9 data, specifically

identifying cases of fetal Klinefelter Syndrome. Specifically, we were interested

in the outcomes of preterm delivery, preeclampsia, intrauterine fetal demise,

cesarean delivery, neonatal death, respiratory distress syndrome (RDS), small for

gestational age, large for gestational age, neonatal death, and infant death.

Bivariate and multivariate analyses were used to compare pregnancies and neonates

affected by prenatally diagnosed Klinefelter Syndrome to those that were not

affected with 47,XXY. RESULTS: There were 2,029,000 deliveries in the cohort,

including 52 women with prenatally diagnosed 47,XXY. Advanced maternal age,

completion of 12th grade, and private insurance were all associated with a

prenatal diagnosis of Klinefelter Syndrome. Compared to unaffected deliveries,

pregnancies complicated by prenatally diagnosed Klinefelter Syndrome had higher

rates of preterm delivery (23.1% vs 9.9%, p=0.0004), cesarean delivery (50.0% vs

30.2%, p=0.004), and RDS (9.6% vs 1.2%, p=<0.0001). Infants with 47,XXY were

markedly more likely to be small for gestational age, including less than the

10th, 5th and 3rd percentile (aOR 5.86 (95% CI 2.99, 11.46), 6.03 (95% CI 2.52,

14.43), and 8.28 (95% CI 3.22, 21.25), p≤0.001). Rates of neonatal death were 9.5

times higher (1.9% vs 0.2% p<0.0001) in the 47,XXY cohort, and rates of infant

death were more than 50 times higher (5.8% vs 0.1%, p<0.0001). In the adjusted

analysis, prenatally diagnosed 47,XXY was associated with increased odds of

preterm delivery <32 weeks (OR 6.81, 95% CI 2. .38, 19.52), IVH (OR 9.08, 95% CI

1.22, 67.7), RDS (OR 8.32, 95% CI 3.22, 21.49), neonatal death (OR 9.77, 1.33,

71.79), and infant death (OR 62.73, 95% CI 19.34, 203.4). CONCLUSION: Pregnancies

affected by prenatally diagnosed Klinefelter Syndrome are at an increased risk of

adverse fetal and neonatal outcomes. These findings may be helpful when

counseling families with pregnancies affected by fetal 47,XXY.

CI - Copyright © 2016 Elsevier Ireland Ltd. All rights reserved.

FAU - Dotters-Katz, Sarah K

AU - Dotters-Katz SK

AD - Department of obstetrics and gynecology, University of North Carolina at Chapel

Hill, Chapel Hill, NC, United States. Electronic address: sarahdk@med.unc.edu.

FAU - Humphrey, Whitney M

AU - Humphrey WM

AD - Department of Obstetrics and Gynecology, Oregon health sciences University,

Portland, OR, United States.

FAU - Senz, Kayli L

AU - Senz KL

AD - Department of Obstetrics and Gynecology, Oregon health sciences University,

Portland, OR, United States.

FAU - Lee, Vanessa R

AU - Lee VR

AD - Department of Obstetrics and Gynecology, Oregon health sciences University,

Portland, OR, United States.

FAU - Shaffer, Brian L

AU - Shaffer BL

AD - Department of Obstetrics and Gynecology, Oregon health sciences University,

Portland, OR, United States.

FAU - Caughey, Aaron B

AU - Caughey AB

AD - Department of Obstetrics and Gynecology, Oregon health sciences University,

Portland, OR, United States.

LA - eng

PT - Journal Article

DEP - 20160609

PL - Ireland

TA - Eur J Obstet Gynecol Reprod Biol

JT - European journal of obstetrics, gynecology, and reproductive biology

JID - 0375672

SB - IM

MH - Adult

MH - Cesarean Section/adverse effects/economics

MH - Cohort Studies

MH - Costs and Cost Analysis

MH - Electronic Health Records

MH - Female

MH - Fetal Death/etiology

MH - *Genetic Testing/economics

MH - Hospital Costs

MH - Humans

MH - Infant, Newborn

MH - Infant, Newborn, Diseases/economics/*etiology/therapy

MH - International Classification of Diseases

MH - Klinefelter Syndrome/*diagnosis/economics/physiopathology/therapy

MH - Male

MH - Maternal Age

MH - Perinatal Death/etiology

MH - Pregnancy

MH - Pregnancy Complications/economics/*etiology/therapy

MH - Pregnancy Outcome

MH - *Prenatal Diagnosis/economics

MH - Retrospective Studies

OTO - NOTNLM

OT - 47 ;XXY

OT - Klinefelter Syndrome

OT - neonatal outcomes

EDAT- 2016/06/20 06:00

MHDA- 2017/04/14 06:00

CRDT- 2016/06/20 06:00

PHST- 2016/04/11 00:00 [received]

PHST- 2016/05/11 00:00 [accepted]

PHST- 2016/06/20 06:00 [entrez]

PHST- 2016/06/20 06:00 [pubmed]

PHST- 2017/04/14 06:00 [medline]

AID - S0301-2115(16)30220-2 [pii]

AID - 10.1016/j.ejogrb.2016.05.006 [doi]

PST - ppublish

SO - Eur J Obstet Gynecol Reprod Biol. 2016 Aug;203:173-6. doi:

10.1016/j.ejogrb.2016.05.006. Epub 2016 Jun 9.

PMID- 31737857

OWN - NLM

STAT- PubMed-not-MEDLINE

LR - 20220411

IS - 2472-1972 (Electronic)

IS - 2472-1972 (Linking)

VI - 3

IP - 12

DP - 2019 Dec 1

TI - Testosterone Treatment in Infants With 47,XXY: Effects on Body Composition.

PG - 2276-2285

LID - 10.1210/js.2019-00274 [doi]

AB - CONTEXT: Boys with XXY have greater adiposity and a higher risk of cardiovascular

disease. Infants with XXY have lower testosterone concentrations than typical

boys, but no studies have evaluated adiposity in infants with XXY or the

physiologic effects of giving testosterone replacement. OBJECTIVE: To determine

the effect of testosterone on body composition in infants with XXY. DESIGN:

Prospective, randomized trial. SETTING: Tertiary care pediatric referral center.

PARTICIPANTS: 20 infants 6 to 15 weeks of age with 47,XXY. INTERVENTION:

Testosterone cypionate 25 mg intramuscularly monthly for three doses vs no

treatment. MAIN OUTCOME MEASURES: Difference in change in adiposity (percent fat

mass z scores); other body composition measures, penile length, and safety

outcomes between treated and untreated infants; and comparison with typical

infants. RESULTS: The increase in percent fat mass (%FM) z scores was greater in

the untreated group than in the treated group (+0.92 ± 0.62 vs -0.12 ± 0.65, P =

0.004). Increases in secondary outcomes were greater in the testosterone-treated

group for total mass, fat-free mass, length z score, stretched penile length, and

growth velocity (P < 0.002 for all). At 5 months of age, adiposity in untreated

infants with XXY was 26.7% compared with 23.2% in healthy male infants of the

same age (P = 0.0037); there was no difference in %FM between the treated XXY

boys and controls. Reported side effects were minimal and self-limited; no

serious adverse events occurred. CONCLUSIONS: Adiposity of untreated infants was

15% greater than that of male controls by 5 months of age. Testosterone treatment

for infants with XXY resulted in positive changes in body composition.

CI - Copyright © 2019 Endocrine Society.

FAU - Davis, Shanlee M

AU - Davis SM

AUID- ORCID: 0000-0002-0304-9550

AD - University of Colorado School of Medicine, Department of Pediatrics, Section of

Endocrinology, Aurora, Colorado.

AD - Children's Hospital Colorado, eXtraordinarY Kids Clinic, Aurora, Colorado.

FAU - Reynolds, Regina M

AU - Reynolds RM

AD - University of Colorado School of Medicine, Department of Pediatrics, Section of

Neonatology, Aurora, Colorado.

FAU - Dabelea, Dana M

AU - Dabelea DM

AD - University of Colorado, School of Public Health, Department of Epidemiology,

Aurora, Colorado.

FAU - Zeitler, Philip S

AU - Zeitler PS

AD - University of Colorado School of Medicine, Department of Pediatrics, Section of

Endocrinology, Aurora, Colorado.

AD - Children's Hospital Colorado, eXtraordinarY Kids Clinic, Aurora, Colorado.

FAU - Tartaglia, Nicole R

AU - Tartaglia NR

AD - Children's Hospital Colorado, eXtraordinarY Kids Clinic, Aurora, Colorado.

AD - University of Colorado School of Medicine, Department of Pediatrics, Section of

Developmental Pediatrics, Aurora, Colorado.

LA - eng

GR - K23 HD092588/HD/NICHD NIH HHS/United States

GR - K23 NS070337/NS/NINDS NIH HHS/United States

GR - R01 HD091251/HD/NICHD NIH HHS/United States

PT - Journal Article

DEP - 20190926

TA - J Endocr Soc

JT - Journal of the Endocrine Society

JID - 101697997

PMC - PMC6846330

OTO - NOTNLM

OT - Klinefelter syndrome

OT - XXY

OT - adiposity

OT - mini-puberty

OT - sex chromosome aneuploidy

OT - testosterone

EDAT- 2019/11/19 06:00

MHDA- 2019/11/19 06:01

CRDT- 2019/11/19 06:00

PHST- 2019/07/20 00:00 [received]

PHST- 2019/09/11 00:00 [accepted]

PHST- 2019/11/19 06:00 [entrez]

PHST- 2019/11/19 06:00 [pubmed]

PHST- 2019/11/19 06:01 [medline]

AID - 201900274 [pii]

AID - 10.1210/js.2019-00274 [doi]

PST - epublish

SO - J Endocr Soc. 2019 Sep 26;3(12):2276-2285. doi: 10.1210/js.2019-00274.

eCollection 2019 Dec 1.

PMID- 27441089

OWN - NLM

STAT- PubMed-not-MEDLINE

DCOM- 20160721

LR - 20181113

IS - 2046-1402 (Print)

IS - 2046-1402 (Electronic)

IS - 2046-1402 (Linking)

VI - 5

DP - 2016

TI - Adolescent Klinefelter syndrome: is there an advantage to testis tissue

harvesting or not?

LID - F1000 Faculty Rev-1595 [pii]

LID - 10.12688/f1000research.8395.1 [doi]

AB - It is currently unclear whether an adolescent with 47,XXY Klinefelter syndrome

will be better off having testicular sperm extraction (TESE) performed in an

effort to 'preserve fertility' for the future or, alternatively, should be

advised to simply wait until adulthood when he and his partner are ready to begin

a family. This report will provide data suggesting that there is no obvious

'preservation' benefit and that recommending TESE to the 47,XXY boy and his

parents may not be as helpful as it might appear and may be overly aggressive.

FAU - Oates, Robert

AU - Oates R

AD - Boston University School of Medicine, Boston, MA, USA.

LA - eng

PT - Journal Article

PT - Review

DEP - 20160706

TA - F1000Res

JT - F1000Research

JID - 101594320

PMC - PMC4937818

OTO - NOTNLM

OT - fertility

OT - non-obstructive azoospermic

OT - spermatogenesis

OT - testicular sperm extraction

COIS- Competing interests: The author declares that he has no competing interests. No

competing interests were disclosed.

EDAT- 2016/07/22 06:00

MHDA- 2016/07/22 06:01

CRDT- 2016/07/22 06:00

PHST- 2016/07/05 00:00 [accepted]

PHST- 2016/07/22 06:00 [entrez]

PHST- 2016/07/22 06:00 [pubmed]

PHST- 2016/07/22 06:01 [medline]

AID - F1000 Faculty Rev-1595 [pii]

AID - 10.12688/f1000research.8395.1 [doi]

PST - epublish

SO - F1000Res. 2016 Jul 6;5:F1000 Faculty Rev-1595. doi:

10.12688/f1000research.8395.1. eCollection 2016.

PMID- 33610733

OWN - NLM

STAT- MEDLINE

DCOM- 20210705

LR - 20220223

IS - 1878-0849 (Electronic)

IS - 1769-7212 (Linking)

VI - 64

IP - 4

DP - 2021 Apr

TI - The contribution of Xp22.31 gene dosage to Turner and Klinefelter syndromes and

sex-biased phenotypes.

PG - 104169

LID - S1769-7212(21)00035-5 [pii]

LID - 10.1016/j.ejmg.2021.104169 [doi]

AB - Turner syndrome (TS) is a rare developmental condition in females caused by

complete, or partial, loss of the second sex chromosome; it is associated with a

number of phenotypes including short stature, ovarian failure and infertility, as

well as neurobehavioural and cognitive manifestations. In contrast, Klinefelter

syndrome (KS) arises from an excess of X chromosome material in males (typical

karyotype is 47,XXY); like TS, KS is associated with infertility and hormonal

imbalance, and behavioural/neurocognitive differences from gonadal sex-matched

counterparts. Lower dosage of genes that escape X-inactivation may partially

explain TS phenotypes, whilst overdosage of these genes may contribute towards

KS-related symptoms. Here, I discuss new findings from individuals with deletions

or duplications limited to Xp22.31 (a region escaping X-inactivation), and

consider the extent to which altered gene dosage within this small interval (and

of the steroid sulfatase (STS) gene in particular) may influence the phenotypic

profiles of TS and KS. The expression of X-escapees can be higher in female than

male tissues; I conclude by considering how lower Xp22.31 gene dosage in males

may increase their likelihood of exhibiting particular phenotypes relative to

females. Understanding the genetic contribution to specific phenotypes in rare

disorders such as TS and KS, and to more common sex-biased phenotypes, will be

important for developing more effective, and more personalised, therapeutic

approaches.

CI - Copyright © 2021 Elsevier Masson SAS. All rights reserved.

FAU - Davies, William

AU - Davies W

AD - School of Psychology, Cardiff University, Cardiff, UK; Division of Psychological

Medicine and Clinical Neurosciences and Centre for Neuropsychiatric Genetics and

Genomics, School of Medicine, Cardiff University, Cardiff, UK; Neuroscience and

Mental Health Research Institute, Cardiff University, Cardiff, UK. Electronic

address: daviesw4@cardiff.ac.uk.

LA - eng

GR - MR/L010305/1/MRC_/Medical Research Council/United Kingdom

PT - Journal Article

PT - Review

DEP - 20210219

PL - Netherlands

TA - Eur J Med Genet

JT - European journal of medical genetics

JID - 101247089

SB - IM

MH - Chromosomes, Human, X/*genetics

MH - Female

MH - *Gene Dosage

MH - Genetic Loci

MH - Humans

MH - Klinefelter Syndrome/*genetics/pathology

MH - Male

MH - *Phenotype

MH - Turner Syndrome/*genetics/pathology

MH - X Chromosome Inactivation

OTO - NOTNLM

OT - Atrial fibrillation

OT - Attention deficit hyperactivity disorder (ADHD)

OT - Autism

OT - NLGN4X

OT - Steroid sulfatase

EDAT- 2021/02/22 06:00

MHDA- 2021/07/06 06:00

CRDT- 2021/02/21 20:30

PHST- 2020/11/30 00:00 [received]

PHST- 2021/01/11 00:00 [revised]

PHST- 2021/02/16 00:00 [accepted]

PHST- 2021/02/22 06:00 [pubmed]

PHST- 2021/07/06 06:00 [medline]

PHST- 2021/02/21 20:30 [entrez]

AID - S1769-7212(21)00035-5 [pii]

AID - 10.1016/j.ejmg.2021.104169 [doi]

PST - ppublish

SO - Eur J Med Genet. 2021 Apr;64(4):104169. doi: 10.1016/j.ejmg.2021.104169. Epub

2021 Feb 19.

PMID- 21217606

OWN - NLM

STAT- MEDLINE

DCOM- 20110303

LR - 20220408

IS - 1565-4753 (Print)

IS - 1565-4753 (Linking)

VI - 8 Suppl 1

DP - 2010 Dec

TI - Considerations for androgen therapy in children and adolescents with Klinefelter

syndrome (47, XXY).

PG - 145-50

AB - The goals of androgen therapy for adolescents are to promote linear growth and

secondary sexual characteristics, at the same time as to permit the normal

accrual of muscle mass, bone mineral content and the adult regional distribution

of body fat. Secondary goals are mainly in the psychosocial sphere, in which

pubertally delayed boys feel that they look too young, are not considered a

'peer' in their age group and have difficulty competing in athletic endeavors.

Puberty often starts normally in adolescents with KS corresponding to the peer

group with genital enlargement and pubic hair growth. The testes start to

enlarge, but rarely expand beyond 6 mL, leaving a discordance between the degree

of sexual development and the size of the testes. Androgen therapy is considered

mainly supplemental and one usually begins with the long acting esters,

testosterone enanthate or cypionate because the other forms patches and gels--are

metered for full male replacement. The dose of testosterone is escalated until

the lower range of the adult dose is reached and then a choice among the various

forms can be made. Treatment-emergent adverse events often represent the

pharmacodynamic effects of an androgen oily skin and acne, but as the dose is

escalated more effects may be noted in the behavioral sphere, especially in

adolescents with Klinefelter syndrome compared to those who receive replacement

therapy with testosterone for other purposes, for example, constitutional delay

of growth and puberty.

FAU - Rogol, Alan D

AU - Rogol AD

AD - The James Whitcomb Riley Hospital for Children, Indiana University School of

Medicine, Indianapolis, IN, USA. adrogol@comcast.net

FAU - Tartaglia, Nicole

AU - Tartaglia N

LA - eng

GR - L40 HD051024/HD/NICHD NIH HHS/United States

PT - Journal Article

PT - Review

PL - Israel

TA - Pediatr Endocrinol Rev

JT - Pediatric endocrinology reviews : PER

JID - 101202124

RN - 3XMK78S47O (Testosterone)

RN - 7Z6522T8N9 (testosterone enanthate)

SB - IM

MH - Adolescent

MH - Child

MH - Chromosomes, Human, X

MH - Hormone Replacement Therapy/*methods

MH - Humans

MH - Klinefelter Syndrome/*drug therapy/physiopathology

MH - Male

MH - Puberty/drug effects/physiology

MH - Testosterone/*administration & dosage/analogs & derivatives

EDAT- 2011/02/09 06:00

MHDA- 2011/03/04 06:00

CRDT- 2011/01/11 06:00

PHST- 2011/01/11 06:00 [entrez]

PHST- 2011/02/09 06:00 [pubmed]

PHST- 2011/03/04 06:00 [medline]

PST - ppublish

SO - Pediatr Endocrinol Rev. 2010 Dec;8 Suppl 1:145-50.

PMID- 32421982

OWN - NLM

STAT- MEDLINE

DCOM- 20210714

LR - 20210714

IS - 1045-4403 (Print)

IS - 1045-4403 (Linking)

VI - 30

IP - 1

DP - 2020

TI - Meiotic Behavior of Extra Sex Chromosomes in Patients with the 47,XXY and 47,XYY

Karyotype and Its Ultimate Consequences for Spermatogenesis.

PG - 19-37

LID - 10.1615/CritRevEukaryotGeneExpr.2020026203 [doi]

AB - Infertility is one of the most important and burning issues in present times, as

a marked increase in the frequency of infertile cases has been observed all over

the world. Chromosomal aneuploidy is among the known factors associated with

infertility, and among sex chromosome aneuploidies, 47,XXY and 47,XYY constitute

the most common class of chromosome abnormality in human live births.

Considerable attention has been given to the somatic abnormalities associated

with these conditions, but less is known about their meiotic progression; that

is, how sex chromosome imbalance influences the meiotic process. It has been

documented that men with the same underlying genetic cause of infertility do not

present with uniform pathology, so it is informative to find out how meiotic

progression differs in patients with similar chromosomal aneuploidy having

different phenotypes. The importance of studying meiotic progression in patients

with sex chromosome abnormalities has increased many fold with the introduction

of assisted reproductive technologies that have made it possible for infertile

men to become biological parents. Hence, exploring the possible consequences of

sex chromosome aneuploidy for meiotic chromosome segregation is worthwhile. The

objective of this review, in the context of current knowledge, is to discuss

problems associated with fertility and progression of meiosis in two relatively

common sex chromosome aneuploidies, 47,XXY and 47,XYY, reported in humans.

FAU - Iqbal, Furhan

AU - Iqbal F

AD - Institute of Pure and Applied Biology, Zoology Division, Bahauddin Zakariya

University, Multan 60800, Pakistan.

LA - eng

PT - Journal Article

PT - Review

PL - United States

TA - Crit Rev Eukaryot Gene Expr

JT - Critical reviews in eukaryotic gene expression

JID - 9007261

RN - 47, XYY syndrome

SB - IM

MH - Humans

MH - Infertility, Male/*genetics/pathology

MH - Klinefelter Syndrome/*genetics/pathology

MH - Male

MH - Meiosis/*genetics

MH - Sex Chromosome Disorders/*genetics/pathology

MH - Sex Chromosomes/genetics

MH - Spermatogenesis/genetics

MH - Spermatozoa/metabolism/pathology

MH - XYY Karyotype/*genetics/pathology

EDAT- 2020/05/19 06:00

MHDA- 2021/07/15 06:00

CRDT- 2020/05/19 06:00

PHST- 2020/05/19 06:00 [entrez]

PHST- 2020/05/19 06:00 [pubmed]

PHST- 2021/07/15 06:00 [medline]

AID - 478e880265896a53,4f7203de5cd64c5e [pii]

AID - 10.1615/CritRevEukaryotGeneExpr.2020026203 [doi]

PST - ppublish

SO - Crit Rev Eukaryot Gene Expr. 2020;30(1):19-37. doi:

10.1615/CritRevEukaryotGeneExpr.2020026203.

PMID- 16192710

OWN - NLM

STAT- MEDLINE

DCOM- 20060123

LR - 20061115

IS - 1424-859X (Electronic)

IS - 1424-8581 (Linking)

VI - 111

IP - 3-4

DP - 2005

TI - Chromosome abnormalities in sperm of individuals with constitutional sex

chromosomal abnormalities.

PG - 310-6

AB - The most common type of karyotype abnormality detected in infertile subjects is

represented by Klinefelter's syndrome, and the most frequent non-chromosomal

alteration is represented by Y chromosome long arm microdeletions. Here we report

our experience and a review of the literature on sperm sex chromosome

aneuploidies in these two conditions. Non mosaic 47,XXY Klinefelter patients (12

subjects) show a significantly lower percentage of normal Y-bearing sperm and

slightly higher percentage of normal X-bearing sperm. Consistent with the

hypothesis that 47,XXY germ cells may undergo and complete meiosis, aneuploidy

rate for XX- and XY-disomies is also increased with respect to controls, whereas

the percentage of YY-disomies is normal. Aneuploidy rates in men with mosaic

47,XXY/46,XY (11 subjects) are lower than those observed in men with non-mosaic

Klinefelter's syndrome, and only the frequency of XY-disomic sperm is

significantly higher with respect to controls. Although the great majority of

children born by intracytoplasmic sperm injection from Klinefelter subjects are

chromosomally normal, the risk of producing offspring with chromosome

aneuploidies is significant. Men with Y chromosome microdeletions (14 subjects)

showed a reduction of normal Y-bearing sperm, and an increase in nullisomic and

XY-disomic sperm, suggesting an instability of the deleted Y chromosome causing

its loss in germ cells, and meiotic alterations leading to XY non-disjunction.

Intracytoplasmic injection of sperm from Y-deleted men will therefore transmit

the deletion to male children, and therefore the spermatogenic impairment, but

raises also concerns of generating 45,X and 47,XXY embryos.

CI - Copyright 2005 S. Karger AG, Basel.

FAU - Ferlin, A

AU - Ferlin A

AD - Department of Histology, Microbiology, and Medical Biotechnologies, Centre for

Male Gamete Cryopreservation, University of Padova, Padova, Italy.

FAU - Garolla, A

AU - Garolla A

FAU - Foresta, C

AU - Foresta C

LA - eng

PT - Journal Article

PT - Research Support, Non-U.S. Gov't

PT - Review

PL - Switzerland

TA - Cytogenet Genome Res

JT - Cytogenetic and genome research

JID - 101142708

SB - IM

MH - Chromosome Aberrations/*statistics & numerical data

MH - Chromosome Deletion

MH - Chromosomes, Human, Y/genetics

MH - Female

MH - Humans

MH - Klinefelter Syndrome/*genetics

MH - Male

MH - Nondisjunction, Genetic/genetics

MH - Sex Chromosome Aberrations/*statistics & numerical data

MH - Spermatozoa/*cytology

RF - 74

EDAT- 2005/09/30 09:00

MHDA- 2006/01/24 09:00

CRDT- 2005/09/30 09:00

PHST- 2004/11/09 00:00 [received]

PHST- 2005/01/06 00:00 [accepted]

PHST- 2005/09/30 09:00 [pubmed]

PHST- 2006/01/24 09:00 [medline]

PHST- 2005/09/30 09:00 [entrez]

AID - 86905 [pii]

AID - 10.1159/000086905 [doi]

PST - ppublish

SO - Cytogenet Genome Res. 2005;111(3-4):310-6. doi: 10.1159/000086905.

PMID- 27832510

OWN - NLM

STAT- MEDLINE

DCOM- 20180910

LR - 20191008

IS - 1573-3599 (Electronic)

IS - 1059-7700 (Print)

IS - 1059-7700 (Linking)

VI - 26

IP - 4

DP - 2017 Aug

TI - The Impact of Living with Klinefelter Syndrome: A Qualitative Exploration of

Adolescents and Adults.

PG - 728-737

LID - 10.1007/s10897-016-0041-z [doi]

AB - Klinefelter syndrome (XXY) is a common yet significantly underdiagnosed condition

with considerable medical, psychological and social implications. Many health

care providers lack familiarity with XXY, resulting in medical management

challenges and a limited understanding of the personal impact of the condition.

Genetic counselors benefit from understanding the challenges adolescents and men

with XXY face to effectively address their medical and psychosocial needs. The

purpose of this study was to understand the impact of living with XXY as an

adolescent or an adult. Individuals aged 14 to 75 years with self-reported XXY

were recruited from online support networks to complete a web-based survey that

included open-ended questions. Open-ended responses were coded and analyzed

thematically (n = 169 to 210 for each open-ended question). Over half of

respondents to the open-ended questions reported challenges in finding health

care providers who are knowledgeable about XXY, with many describing an extensive

diagnostic odyssey and relief when receiving a diagnosis. Individuals sought

support coping with the challenges they face and acknowledgement of the positive

aspects of XXY. Recommendations are made for how genetic counseling can enhance

quality of life for individuals living with XXY.

FAU - Turriff, Amy

AU - Turriff A

AUID- ORCID: 0000-0002-8065-7445

AD - Ophthalmic Genetics & Visual Function Branch, National Eye Institute, National

Institutes of Health, 10 Center Drive MSC 1860, Building 10, Room 10N226,

Bethesda, MD, 20892, USA. turriffa@mail.nih.gov.

FAU - Macnamara, Ellen

AU - Macnamara E

AD - Office of the Clinical Director, National Human Genome Research Institute,

National Institutes of Health, Bethesda, MD, USA.

FAU - Levy, Howard P

AU - Levy HP

AD - Department of Medicine and McKusick-Nathans Institute of Genetic Medicine, Johns

Hopkins University School of Medicine, Baltimore, MD, USA.

FAU - Biesecker, Barbara

AU - Biesecker B

AD - Social and Behavioral Research Branch, National Human Genome Research Institute,

National Institutes of Health, Bethesda, MD, USA.

LA - eng

GR - ZIE HG200353-09/Intramural NIH HHS/United States

PT - Journal Article

DEP - 20161110

TA - J Genet Couns

JT - Journal of genetic counseling

JID - 9206865

SB - IM

MH - Adolescent

MH - Adult

MH - Aged

MH - Genetic Counseling/*psychology

MH - Humans

MH - Klinefelter Syndrome/*psychology

MH - Male

MH - Middle Aged

MH - Qualitative Research

MH - Quality of Life/*psychology

MH - Young Adult

PMC - PMC5425317

MID - NIHMS835038

OTO - NOTNLM

OT - 47,XXY

OT - Klinefelter syndrome

OT - Psychosocial impact

OT - Sex chromosome aneuploidy

COIS- Conflict of Interest Amy Turriff, Ellen Macnamara, Howard Levy, and Barbara

Biesecker declare that they have no conflict of interest.

EDAT- 2016/11/11 06:00

MHDA- 2018/09/11 06:00

CRDT- 2016/11/11 06:00

PHST- 2016/02/26 00:00 [received]

PHST- 2016/10/20 00:00 [accepted]

PHST- 2016/11/11 06:00 [pubmed]

PHST- 2018/09/11 06:00 [medline]

PHST- 2016/11/11 06:00 [entrez]

AID - 10.1007/s10897-016-0041-z [pii]

AID - 10.1007/s10897-016-0041-z [doi]

PST - ppublish

SO - J Genet Couns. 2017 Aug;26(4):728-737. doi: 10.1007/s10897-016-0041-z. Epub 2016

Nov 10.

PMID- 32449318

OWN - NLM

STAT- MEDLINE

DCOM- 20210505

LR - 20210505

IS - 1552-4876 (Electronic)

IS - 1552-4868 (Linking)

VI - 184

IP - 2

DP - 2020 Jun

TI - Integration and reanalysis of transcriptomics and methylomics data derived from

blood and testis tissue of men with 47,XXY Klinefelter syndrome indicates the

primary involvement of Sertoli cells in the testicular pathogenesis.

PG - 239-255

LID - 10.1002/ajmg.c.31793 [doi]

AB - Klinefelter syndrome (KS; 47,XXY) is the most common sex chromosomal anomaly and

causes a multitude of symptoms. Often the most noticeable symptom is infertility

caused by azoospermia with testicular histology showing hyalinization of tubules,

germ cells loss, and Leydig cell hyperplasia. The germ cell loss begins early in

life leading to partial hyalinization of the testis at puberty, but the

mechanistic drivers behind this remain poorly understood. In this systematic

review, we summarize the current knowledge on developmental changes in the

cellularity of KS gonads supplemented by a comparative analysis of the fetal and

adult gonadal transcriptome, and blood transcriptome and methylome of men with

KS. We identified a high fraction of upregulated genes that escape X-chromosome

inactivation, thus supporting previous hypotheses that these are the main drivers

of the testicular phenotype in KS. Enrichment analysis showed overrepresentation

of genes from the X- and Y-chromosome and testicular transcription factors.

Furthermore, by re-evaluation of recent single cell RNA-sequencing data

originating from adult KS testis, we found novel evidence that the Sertoli cell

is the most affected cell type. Our results are consistent with disturbed

cross-talk between somatic and germ cells in the KS testis, and with X-escapee

genes acting as mediators.

CI - © 2020 Wiley Periodicals, Inc.

FAU - Winge, Sofia B

AU - Winge SB

AUID- ORCID: 0000-0003-1666-1228

AD - Department of Growth and Reproduction, Rigshospitalet, University of Copenhagen,

Copenhagen, Denmark.

AD - International Center for Research and Research Training in Endocrine Disruption

of Male Reproduction and Child Health (EDMaRC), Rigshospitalet, University of

Copenhagen, Copenhagen, Denmark.

AD - Bioinformatics Research Centre, Aarhus University, Aarhus, Denmark.

FAU - Soraggi, Samuele

AU - Soraggi S

AD - Bioinformatics Research Centre, Aarhus University, Aarhus, Denmark.

FAU - Schierup, Mikkel H

AU - Schierup MH

AD - Bioinformatics Research Centre, Aarhus University, Aarhus, Denmark.

FAU - Rajpert-De Meyts, Ewa

AU - Rajpert-De Meyts E

AD - Department of Growth and Reproduction, Rigshospitalet, University of Copenhagen,

Copenhagen, Denmark.

AD - International Center for Research and Research Training in Endocrine Disruption

of Male Reproduction and Child Health (EDMaRC), Rigshospitalet, University of

Copenhagen, Copenhagen, Denmark.

FAU - Almstrup, Kristian

AU - Almstrup K

AUID- ORCID: 0000-0002-1832-0307

AD - Department of Growth and Reproduction, Rigshospitalet, University of Copenhagen,

Copenhagen, Denmark.

AD - International Center for Research and Research Training in Endocrine Disruption

of Male Reproduction and Child Health (EDMaRC), Rigshospitalet, University of

Copenhagen, Copenhagen, Denmark.

LA - eng

PT - Journal Article

PT - Research Support, Non-U.S. Gov't

PT - Review

DEP - 20200525

PL - United States

TA - Am J Med Genet C Semin Med Genet

JT - American journal of medical genetics. Part C, Seminars in medical genetics

JID - 101235745

SB - IM

MH - Adult

MH - Chromosomes, Human, X/genetics

MH - Chromosomes, Human, Y/genetics

MH - DNA Methylation/*genetics

MH - Gonads/growth & development/metabolism

MH - Humans

MH - Infertility, Male/*genetics/pathology

MH - Klinefelter Syndrome/*blood/genetics/pathology

MH - Male

MH - Sertoli Cells/metabolism/pathology

MH - Testis/metabolism/pathology

MH - Transcriptome/*genetics

OTO - NOTNLM

OT - *Klinefelter syndrome

OT - *human testis

OT - *methylome

OT - *single cell RNA-sequencing

OT - *transcriptome

EDAT- 2020/05/26 06:00

MHDA- 2021/05/06 06:00

CRDT- 2020/05/26 06:00

PHST- 2020/02/14 00:00 [received]

PHST- 2020/04/03 00:00 [revised]

PHST- 2020/04/22 00:00 [accepted]

PHST- 2020/05/26 06:00 [pubmed]

PHST- 2021/05/06 06:00 [medline]

PHST- 2020/05/26 06:00 [entrez]

AID - 10.1002/ajmg.c.31793 [doi]

PST - ppublish

SO - Am J Med Genet C Semin Med Genet. 2020 Jun;184(2):239-255. doi:

10.1002/ajmg.c.31793. Epub 2020 May 25.

PMID- 35947806

OWN - NLM

STAT- Publisher

LR - 20220815

IS - 1536-7312 (Electronic)

IS - 0196-206X (Linking)

DP - 2022 Aug 15

TI - Novel Neurocognitive Profile in a Minority of Boys with 47,XXY (Klinefelter

Syndrome).

LID - 10.1097/DBP.0000000000001111 [doi]

AB - INTRODUCTION: 47,XXY, also known as Klinefelter syndrome, is the most commonly

occurring sex chromosomal variation (1:660). The neurocognitive profile of boys

with 47,XXY, in addition to verbal abilities, language skills, and general

intelligence, has been explored in this study. METHODS: Fifty-five participants

with 47,XXY were segregated into groups according to their performance on the

Wechsler Intelligence Scale for Children (WISC): (1) those with a higher

performance intelligence quotient (PIQ) in comparison with their verbal IQ (VIQ)

and (2) those with a higher VIQ compared with their PIQ. Two-tailed independent t

tests were completed to analyze group differences. RESULTS: Our study results

demonstrate novel findings that one-third of subjects have higher verbal

capabilities than perceptual skills. Those participants who showed the typical

presentation of 47,XXY with increased PIQ in comparison with their VIQ excelled

on perceptual and visual spatial subtests on the WISC and on nonverbal IQ on the

Leiter International Performance Scale-III. In addition, it was found that

expressive and receptive vocabulary skills were commensurate in both groups,

which has not been reported previously. DISCUSSION: To the best of our knowledge,

this is the first study to identify an alternative profile of 47,XXY with

increased verbal capabilities in comparison with perceptual skills. In addition,

previous research has found that boys with 47,XXY often show increased receptive

vocabulary skills in comparison with their expressive vocabulary skills early in

life. Therefore, our findings of commensurate expressive and receptive vocabulary

skills suggest that age may be an impactful factor in vocabulary development.

Further research is necessary to determine individualized treatment options for

these patients, focusing on the specific cognitive profile they present.

CI - Copyright © 2022 Wolters Kluwer Health, Inc. All rights reserved.

FAU - Samango-Sprouse, Carole A

AU - Samango-Sprouse CA

AD - Department of Pediatrics, George Washington University, Washington, DC.

AD - Department of Human and Molecular Genetics, Florida International University,

Miami, FL.

AD - Department of Research, The Focus Foundation, Davidsonville, MD.

FAU - Hamzik, Mary P

AU - Hamzik MP

AD - Department of Research, The Focus Foundation, Davidsonville, MD.

FAU - Khaksari, Kosar

AU - Khaksari K

AD - Department of Research, The Focus Foundation, Davidsonville, MD.

FAU - Brooks, Michaela R

AU - Brooks MR

AD - Department of Research, The Focus Foundation, Davidsonville, MD.

FAU - Sadeghin, Teresa

AU - Sadeghin T

AD - Department of Research, The Focus Foundation, Davidsonville, MD.

FAU - Gropman, Andrea L

AU - Gropman AL

AD - Division of Neurogenetics and Developments Pediatrics, Children's National Health

System, Washington, DC; and.

AD - Department of Neurology, George Washington University, Washington, DC.

LA - eng

PT - Journal Article

DEP - 20220815

PL - United States

TA - J Dev Behav Pediatr

JT - Journal of developmental and behavioral pediatrics : JDBP

JID - 8006933

SB - IM

COIS- Disclosure: The authors declare no conflict of interest.

EDAT- 2022/08/11 06:00

MHDA- 2022/08/11 06:00

CRDT- 2022/08/10 16:02

PHST- 2022/01/13 00:00 [received]

PHST- 2022/06/01 00:00 [accepted]

PHST- 2022/08/11 06:00 [pubmed]

PHST- 2022/08/11 06:00 [medline]

PHST- 2022/08/10 16:02 [entrez]

AID - 00004703-990000000-00043 [pii]

AID - 10.1097/DBP.0000000000001111 [doi]

PST - aheadofprint

SO - J Dev Behav Pediatr. 2022 Aug 15. doi: 10.1097/DBP.0000000000001111.

PMID- 32235276

OWN - NLM

STAT- MEDLINE

DCOM- 20201214

LR - 20210206

IS - 1473-6586 (Electronic)

IS - 0963-0643 (Linking)

VI - 30

IP - 3

DP - 2020 May

TI - A clinical algorithm for management of fertility in adolescents with the

Klinefelter syndrome.

PG - 324-327

LID - 10.1097/MOU.0000000000000757 [doi]

AB - PURPOSE OF REVIEW: The review presents a clinical algorithm for the evaluation

and treatment for adolescents with Klinefelter's syndrome who desire fertility

preservation. RECENT FINDINGS: Sperm is present in the ejaculate in around 8% of

men with Klinefelter's syndrome. Although most are severely

oligospermic/azoospermic, 43-45% of men will have sperm found during a testicular

sperm extraction, reaching up to 70% in adolescents. SUMMARY: Klinefelter's

syndrome (47, XXY) causes hypogonadotophic hypogonadism and severe

oligospermia/azoospermia rendering natural conception rare. During puberty, boys

often require testosterone replacement therapy to develop secondary sexual

characteristics, which can further decrease spermatogenesis. There is a

progressive decrease of testicular germ cells after the onset of puberty,

suggesting that fertility evaluation and preservation should begin shortly

thereafter. In adolescents desiring fertility evaluation, any testosterone

therapy should be discontinued, hormones and gonadotrophins measured, and a semen

analysis obtained. Adolescents with low testosterone are administered aromatase

inhibitors, selective estrogen receptors modulators and/or human chorionic

gonadotropin to increase endogenous testosterone production. After testosterone

levels are normalized, semen analysis is performed, and cryopreservation

encouraged if sperm is present. For those without sperm in the ejaculate, a

testicular sperm extraction is offered.

FAU - Masterson, Thomas A 3rd

AU - Masterson TA 3rd

AD - Department of Urology, University of Miami Miller School of Medicine, Miami,

Florida.

FAU - Nassau, Daniel E

AU - Nassau DE

AD - Department of Urology, Lenox Hill Hospital, Donald and Barbara Zucker School of

Medicine at Hofstra/Northwell, New York, New York, USA.

FAU - Ramasamy, Ranjith

AU - Ramasamy R

AD - Department of Urology, University of Miami Miller School of Medicine, Miami,

Florida.

LA - eng

PT - Journal Article

PT - Review

PL - United States

TA - Curr Opin Urol

JT - Current opinion in urology

JID - 9200621

RN - 3XMK78S47O (Testosterone)

SB - IM

MH - Adolescent

MH - Algorithms

MH - *Azoospermia/complications

MH - Fertility/physiology

MH - *Fertility Preservation

MH - Humans

MH - Klinefelter Syndrome/*complications/metabolism

MH - Male

MH - Reproductive Techniques, Assisted

MH - Semen Analysis

MH - *Semen Preservation

MH - *Sperm Retrieval

MH - Spermatogenesis

MH - Spermatozoa

MH - Testis

MH - Testosterone/*therapeutic use

EDAT- 2020/04/03 06:00

MHDA- 2020/12/15 06:00

CRDT- 2020/04/03 06:00

PHST- 2020/04/03 06:00 [entrez]

PHST- 2020/04/03 06:00 [pubmed]

PHST- 2020/12/15 06:00 [medline]

AID - 00042307-202005000-00008 [pii]

AID - 10.1097/MOU.0000000000000757 [doi]

PST - ppublish

SO - Curr Opin Urol. 2020 May;30(3):324-327. doi: 10.1097/MOU.0000000000000757.

PMID- 29030589

OWN - NLM

STAT- MEDLINE

DCOM- 20190701

LR - 20190701

IS - 2045-2322 (Electronic)

IS - 2045-2322 (Linking)

VI - 7

IP - 1

DP - 2017 Oct 13

TI - Aberrant ocular architecture and function in patients with Klinefelter syndrome.

PG - 13130

LID - 10.1038/s41598-017-13528-4 [doi]

LID - 13130

AB - Klinefelter Syndrome (KS), the most common chromosomal disorder in men (47,XXY),

is associated with numerous comorbidities. Based on a number of isolated case

reports, we performed the first systematic and comprehensive evaluation of eye

health in KS patients with a focus on ocular structure and vascularization.

Twenty-one KS patients and 26 male and 38 female controls underwent a variety of

non-invasive examinations investigating ocular morphology (examination of retinal

thickness, optic nerve head, and cornea) and function (visual field testing and

quantification of ocular vessel density by optical coherence tomography

angiography). In comparison to healthy controls, KS patients exhibited a smaller

foveal avascular zone and a decreased retinal thickness due to a drastically

thinner outer nuclear layer. The cornea of KS patients showed a decreased

peripheral thickness and volume. In perimetry evaluation, KS patients required

brighter stimuli and gave more irregular values. KS patients show an ocular

phenotype including morphological and functional features, which is very likely

caused by the supernumerary X chromosome. Thus, KS should not be limited to

infertility, endocrine dysfunction, neurocognitive and psychosocial

comorbidities. Defining an aberrant ocular morphology and function, awareness for

possible eye problems should be raised.

FAU - Brand, Cristin

AU - Brand C

AD - Institute of Reproductive and Regenerative Biology, Centre of Reproductive

Medicine and Andrology, University of Muenster, Muenster, Germany.

FAU - Zitzmann, Michael

AU - Zitzmann M

AD - Department of Clinical and Surgical Andrology, Centre of Reproductive Medicine

and Andrology, University of Muenster, Muenster, Germany.

FAU - Eter, Nicole

AU - Eter N

AD - Department of Ophthalmology, University of Muenster, Muenster, Germany.

FAU - Kliesch, Sabine

AU - Kliesch S

AD - Department of Clinical and Surgical Andrology, Centre of Reproductive Medicine

and Andrology, University of Muenster, Muenster, Germany.

FAU - Wistuba, Joachim

AU - Wistuba J

AD - Institute of Reproductive and Regenerative Biology, Centre of Reproductive

Medicine and Andrology, University of Muenster, Muenster, Germany.

FAU - Alnawaiseh, Maged

AU - Alnawaiseh M

AD - Department of Ophthalmology, University of Muenster, Muenster, Germany.

FAU - Heiduschka, Peter

AU - Heiduschka P

AD - Department of Ophthalmology, University of Muenster, Muenster, Germany.

peter.heiduschka@ukmuenster.de.

LA - eng

PT - Journal Article

PT - Research Support, Non-U.S. Gov't

DEP - 20171013

TA - Sci Rep

JT - Scientific reports

JID - 101563288

SB - IM

MH - Chromosomes, Human, X/*genetics

MH - Cornea/metabolism/pathology

MH - Female

MH - Humans

MH - Klinefelter Syndrome/genetics/*pathology

MH - Male

MH - Optic Disk/metabolism/pathology

MH - Retina/metabolism/pathology

MH - Retinal Vessels/metabolism/pathology

PMC - PMC5640645

COIS- JW and MZ received public grants to conduct this study (German Research

Foundation and Medical Faculty at Muenster University). The funders of the study

had no role in the study design, data collection, data analysis or

interpretation, writing of the report, or decision to submit the results for

publication. The remaining authors have nothing to disclose.

EDAT- 2017/10/17 06:00

MHDA- 2019/07/02 06:00

CRDT- 2017/10/15 06:00

PHST- 2017/06/09 00:00 [received]

PHST- 2017/09/25 00:00 [accepted]

PHST- 2017/10/15 06:00 [entrez]

PHST- 2017/10/17 06:00 [pubmed]

PHST- 2019/07/02 06:00 [medline]

AID - 10.1038/s41598-017-13528-4 [pii]

AID - 13528 [pii]

AID - 10.1038/s41598-017-13528-4 [doi]

PST - epublish

SO - Sci Rep. 2017 Oct 13;7(1):13130. doi: 10.1038/s41598-017-13528-4.

PMID- 21737434

OWN - NLM

STAT- MEDLINE

DCOM- 20130123

LR - 20220330

IS - 1749-5024 (Electronic)

IS - 1749-5016 (Print)

IS - 1749-5016 (Linking)

VI - 7

IP - 6

DP - 2012 Aug

TI - Neural systems for social cognition in Klinefelter syndrome (47,XXY): evidence

from fMRI.

PG - 689-97

LID - 10.1093/scan/nsr041 [doi]

AB - Klinefelter syndrome (KS) is a chromosomal condition (47, XXY) that may help us

to unravel gene-brain behavior pathways to psychopathology. The phenotype

includes social cognitive impairments and increased risk for autism traits. We

used functional MRI to study neural mechanisms underlying social information

processing. Eighteen nonclinical controls and thirteen men with XXY were scanned

during judgments of faces with regard to trustworthiness and age. While judging

faces as untrustworthy in comparison to trustworthy, men with XXY displayed less

activation than controls in (i) the amygdala, which plays a key role in screening

information for socio-emotional significance, (ii) the insula, which plays a role

in subjective emotional experience, as well as (iii) the fusiform gyrus and (iv)

the superior temporal sulcus, which are both involved in the perceptual

processing of faces and which were also less involved during age judgments in men

with XXY. This is the first study showing that KS can be associated with reduced

involvement of the neural network subserving social cognition. Studying KS may

increase our understanding of the genetic and hormonal basis of neural

dysfunctions contributing to abnormalities in social cognition and behavior,

which are considered core abnormalities in psychiatric disorders such as autism

and schizophrenia.

FAU - van Rijn, Sophie

AU - van Rijn S

AD - Leiden University, Faculty of Social Sciences, Clinical Child and Adolescent

Studies, Wassenaarseweg 52, 2333 AK, Leiden, The Netherlands.

srijn@fsw.leidenuniv.nl

FAU - Swaab, Hanna

AU - Swaab H

FAU - Baas, Daan

AU - Baas D

FAU - de Haan, Edward

AU - de Haan E

FAU - Kahn, René S

AU - Kahn RS

FAU - Aleman, André

AU - Aleman A

LA - eng

PT - Journal Article

PT - Research Support, Non-U.S. Gov't

DEP - 20110706

TA - Soc Cogn Affect Neurosci

JT - Social cognitive and affective neuroscience

JID - 101288795

RN - S88TT14065 (Oxygen)

SB - IM

MH - Adult

MH - Attention/physiology

MH - Brain/*blood supply/pathology

MH - *Brain Mapping

MH - Cognition Disorders/*etiology/*pathology

MH - Face

MH - Humans

MH - Image Processing, Computer-Assisted

MH - Judgment/physiology

MH - Klinefelter Syndrome/*complications/pathology

MH - Magnetic Resonance Imaging

MH - Male

MH - Middle Aged

MH - Neuropsychological Tests

MH - Oxygen/blood

MH - Photic Stimulation

MH - Severity of Illness Index

MH - *Social Behavior

MH - Young Adult

PMC - PMC3427864

EDAT- 2011/07/09 06:00

MHDA- 2013/01/24 06:00

CRDT- 2011/07/09 06:00

PHST- 2011/07/09 06:00 [entrez]

PHST- 2011/07/09 06:00 [pubmed]

PHST- 2013/01/24 06:00 [medline]

AID - nsr041 [pii]

AID - 10.1093/scan/nsr041 [doi]

PST - ppublish

SO - Soc Cogn Affect Neurosci. 2012 Aug;7(6):689-97. doi: 10.1093/scan/nsr041. Epub

2011 Jul 6.

PMID- 33752588

OWN - NLM

STAT- MEDLINE

DCOM- 20210826

LR - 20210826

IS - 1866-1955 (Electronic)

IS - 1866-1947 (Print)

IS - 1866-1947 (Linking)

VI - 13

IP - 1

DP - 2021 Mar 22

TI - Modeling familial predictors of proband outcomes in neurogenetic disorders:

initial application in XYY syndrome.

PG - 12

LID - 10.1186/s11689-021-09360-7 [doi]

LID - 12

AB - BACKGROUND: Disorders of gene dosage can significantly increase risk for

psychopathology, but outcomes vary greatly amongst carriers of any given

chromosomal aneuploidy or sub-chromosomal copy number variation (CNV). One

potential path to advance precision medicine for neurogenetic disorders is

modeling penetrance in probands relative to observed phenotypes in their

non-carrier relatives. Here, we seek to advance this general analytic framework

by developing new methods in application to XYY syndrome-a sex chromosome

aneuploidy that is known to increase risk for psychopathology. METHODS: We

analyzed a range of cognitive and behavioral domains in XYY probands and their

non-carrier family members (n = 58 families), including general cognitive ability

(FSIQ), as well as continuous measures of traits related to autism spectrum

disorder (ASD) and attention deficit hyperactivity disorder (ADHD). Proband and

relative scores were compared using covariance, regression and cluster analysis.

Comparisons were made both within and across traits. RESULTS: Proband scores were

shifted away from family scores with effect sizes varying between 0.9 and 2.4

across traits. Only FSIQ and vocabulary scores showed a significant positive

correlation between probands and their non-carrier relatives across families

(R(2) ~ 0.4). Variability in family FSIQ also cross-predicted variability in

proband ASD trait severity. Cluster analysis across all trait-relative pairings

revealed that variability in parental psychopathology was more weakly coupled to

their XYY versus their euploid offspring. CONCLUSIONS: We present a suite of

generalizable methods for modeling variable penetrance in aneuploidy and CNV

carriers using family data. These methods update estimates of phenotypic

penetrance for XYY and suggest that the predictive utility of family data is

likely to vary for different traits and different gene dosage disorders. TRIAL

REGISTRATIONS: ClinicalTrials.gov NCT00001246 , "89-M-0006: Brain Imaging of

Childhood Onset Psychiatric Disorders, Endocrine Disorders and Healthy Controls."

Date of registry: 01 October 1989.

FAU - Wilson, Kathleen E

AU - Wilson KE

AD - Section on Developmental Neurogenomics, Human Genetics Branch, National Institute

of Mental Health, National Institutes of Health, Building 10 Room 4N242 MSC 1367,

10 Center Drive, Bethesda, MD, 20892-1367, USA.

FAU - Fish, Ari M

AU - Fish AM

AD - Section on Developmental Neurogenomics, Human Genetics Branch, National Institute

of Mental Health, National Institutes of Health, Building 10 Room 4N242 MSC 1367,

10 Center Drive, Bethesda, MD, 20892-1367, USA.

FAU - Mankiw, Catherine

AU - Mankiw C

AD - Section on Developmental Neurogenomics, Human Genetics Branch, National Institute

of Mental Health, National Institutes of Health, Building 10 Room 4N242 MSC 1367,

10 Center Drive, Bethesda, MD, 20892-1367, USA.

FAU - Xenophontos, Anastasia

AU - Xenophontos A

AD - Section on Developmental Neurogenomics, Human Genetics Branch, National Institute

of Mental Health, National Institutes of Health, Building 10 Room 4N242 MSC 1367,

10 Center Drive, Bethesda, MD, 20892-1367, USA.

FAU - Warling, Allysa

AU - Warling A

AD - Section on Developmental Neurogenomics, Human Genetics Branch, National Institute

of Mental Health, National Institutes of Health, Building 10 Room 4N242 MSC 1367,

10 Center Drive, Bethesda, MD, 20892-1367, USA.

FAU - Whitman, Ethan

AU - Whitman E

AD - Section on Developmental Neurogenomics, Human Genetics Branch, National Institute

of Mental Health, National Institutes of Health, Building 10 Room 4N242 MSC 1367,

10 Center Drive, Bethesda, MD, 20892-1367, USA.

FAU - Clasen, Liv

AU - Clasen L

AD - Section on Developmental Neurogenomics, Human Genetics Branch, National Institute

of Mental Health, National Institutes of Health, Building 10 Room 4N242 MSC 1367,

10 Center Drive, Bethesda, MD, 20892-1367, USA.

FAU - Torres, Erin

AU - Torres E

AD - Section on Developmental Neurogenomics, Human Genetics Branch, National Institute

of Mental Health, National Institutes of Health, Building 10 Room 4N242 MSC 1367,

10 Center Drive, Bethesda, MD, 20892-1367, USA.

FAU - Blumenthal, Jonathan

AU - Blumenthal J

AD - Section on Developmental Neurogenomics, Human Genetics Branch, National Institute

of Mental Health, National Institutes of Health, Building 10 Room 4N242 MSC 1367,

10 Center Drive, Bethesda, MD, 20892-1367, USA.

FAU - Raznahan, Armin

AU - Raznahan A

AD - Section on Developmental Neurogenomics, Human Genetics Branch, National Institute

of Mental Health, National Institutes of Health, Building 10 Room 4N242 MSC 1367,

10 Center Drive, Bethesda, MD, 20892-1367, USA. raznahana@mail.nih.gov.

LA - eng

SI - ClinicalTrials.gov/NCT00001246

GR - ZIA MH002949-04/MH/NIMH NIH HHS/United States

PT - Journal Article

PT - Research Support, N.I.H., Intramural

DEP - 20210322

TA - J Neurodev Disord

JT - Journal of neurodevelopmental disorders

JID - 101483832

RN - 47, XYY syndrome

SB - IM

MH - Adolescent

MH - Adult

MH - Autism Spectrum Disorder

MH - Child

MH - Child, Preschool

MH - DNA Copy Number Variations

MH - Family

MH - Humans

MH - Male

MH - *Sex Chromosome Disorders

MH - *XYY Karyotype

MH - Young Adult

PMC - PMC7986517

OTO - NOTNLM

OT - *Copy number variants

OT - *Modeling penetrance

OT - *Neurogenetic disorders

OT - *Precision psychiatry

OT - *Sex chromosome aneuploidies

COIS- All authors were or currently are employed by the National Institute of Mental

Health (NIMH). The authors have no additional competing interests to declare.

EDAT- 2021/03/24 06:00

MHDA- 2021/08/27 06:00

CRDT- 2021/03/23 05:42

PHST- 2020/05/02 00:00 [received]

PHST- 2021/03/08 00:00 [accepted]

PHST- 2021/03/23 05:42 [entrez]

PHST- 2021/03/24 06:00 [pubmed]

PHST- 2021/08/27 06:00 [medline]

AID - 10.1186/s11689-021-09360-7 [pii]

AID - 9360 [pii]

AID - 10.1186/s11689-021-09360-7 [doi]

PST - epublish

SO - J Neurodev Disord. 2021 Mar 22;13(1):12. doi: 10.1186/s11689-021-09360-7.

PMID- 27257411

OWN - NLM

STAT- PubMed-not-MEDLINE

DCOM- 20160603

LR - 20200929

IS - 1687-9848 (Print)

IS - 1687-9856 (Electronic)

IS - 1687-9848 (Linking)

VI - 2016

DP - 2016

TI - A rare 47 XXY/46 XX mosaicism with clinical features of Klinefelter syndrome.

PG - 11

LID - 10.1186/s13633-016-0029-3 [doi]

LID - 11

AB - BACKGROUND: 47 XXY/46 XX mosaicism with characteristics suggesting Klinefelter

syndrome is very rare and at present, only seven cases have been reported in the

literature. CASE PRESENTATION: We report an Indian boy diagnosed as variant of

Klinefelter syndrome with 47 XXY/46 XX mosaicism at age 12 years. He was noted to

have right cryptorchidism and chordae at birth, but did not have surgery for

these until age 3 years. During surgery, the right gonad was atrophic and

removed. Histology revealed atrophic ovarian tissue. Pelvic ultrasound showed no

Mullerian structures. There was however no clinical follow up and he was raised

as a boy. At 12 years old he was re-evaluated because of parental concern about

his 'female' body habitus. He was slightly overweight, had eunuchoid body habitus

with mild gynaecomastia. The right scrotal sac was empty and a 2mls testis was

present in the left scrotum. Penile length was 5.2 cm and width 2.0 cm. There was

absent pubic or axillary hair. Pronation and supination of his upper limbs were

reduced and x-ray of both elbow joints revealed bilateral radioulnar synostosis.

The baseline laboratory data were LH < 0.1 mIU/ml, FSH 1.4 mIU/ml, testosterone

0.6 nmol/L with raised estradiol, 96 pmol/L. HCG stimulation test showed poor

Leydig cell response. The karyotype based on 76 cells was 47 XXY[9]/46 XX[67]

with SRY positive. Laparoscopic examination revealed no Mullerian structures.

CONCLUSION: Insisting on an adequate number of cells (at least 50) to be examined

during karyotyping is important so as not to miss diagnosing mosaicism.

FAU - Mohd Nor, Noor Shafina

AU - Mohd Nor NS

AD - Faculty of Medicine, Universiti Teknologi MARA, Sungai Buloh, Selangor Malaysia.

FAU - Jalaludin, Muhammad Yazid

AU - Jalaludin MY

AD - Department of Paediatrics, Faculty of Medicine, University of Malaya, 50603 Kuala

Lumpur, Malaysia ; Paediatric and Child Health Research Group, Faculty of

Medicine, University Malaya, Kuala Lumpur, Malaysia.

LA - eng

PT - Case Reports

DEP - 20160602

TA - Int J Pediatr Endocrinol

JT - International journal of pediatric endocrinology

JID - 101516111

PMC - PMC4890323

OTO - NOTNLM

OT - 47XXY/46XX mosaicism

OT - Klinefelter syndrome

OT - Radioulnar synostosis

EDAT- 2016/06/04 06:00

MHDA- 2016/06/04 06:01

CRDT- 2016/06/04 06:00

PHST- 2015/12/30 00:00 [received]

PHST- 2016/05/05 00:00 [accepted]

PHST- 2016/06/04 06:00 [entrez]

PHST- 2016/06/04 06:00 [pubmed]

PHST- 2016/06/04 06:01 [medline]

AID - 29 [pii]

AID - 10.1186/s13633-016-0029-3 [doi]

PST - ppublish

SO - Int J Pediatr Endocrinol. 2016;2016:11. doi: 10.1186/s13633-016-0029-3. Epub 2016

Jun 2.

PMID- 34504726

OWN - NLM

STAT- PubMed-not-MEDLINE

LR - 20210911

IS - 2146-4596 (Print)

IS - 2146-460X (Electronic)

IS - 2146-460X (Linking)

VI - 10

IP - 3

DP - 2021 Sep

TI - Klinefelter's Syndrome with Maternal Uniparental Disomy X, Interstitial Xp22.31

Deletion, X-linked Ichthyosis, and Severe Central Nervous System Regression.

PG - 222-229

LID - 10.1055/s-0040-1715573 [doi]

AB - We presented in this article a patient with Klinefelter syndrome (KS) (47,XXY)

who had maternal nondisjunction and uniparental disomy of the X chromosome with

regions of heterodisomy and isodisomy, an interstitial Xp22.31 deletion of both X

chromosomes, and other problems. His mother also possesses the same Xp22.31

deletion. The patient presented with status epilepticus and stroke, followed by

severe brain atrophy and developmental regression. His unusual clinical and

cytogenetic findings apparently have not been reported with either KS or Xp22.31

deletions. Based on the patient's available genetic and biochemical information,

we cannot satisfactorily explain his seizures, strokes, or catastrophic brain

regression.

CI - Thieme. All rights reserved.

FAU - Brault, Jennifer

AU - Brault J

AD - Department of Pediatrics, Division of Pediatric Neurology, Vanderbilt University

School of Medicine, Nashville, Tennessee, United States.

AD - Department of Pediatrics, Division of Medical Genetic and Genomic Medicine,

Vanderbilt University School of Medicine, Nashville, Tennessee, United States.

FAU - Walsh, Laurence

AU - Walsh L

AD - Department of Medical and Molecular Genetics, Indiana University School of

Medicine, Indianapolis, Indiana, United States.

AD - Department of Pediatrics, Indiana University School of Medicine, Indianapolis,

Indiana, United States.

AD - Department of Neurology, Section of Child Neurology, Indiana University School of

Medicine, Indianapolis, Indiana, United States.

FAU - Vance, Gail H

AU - Vance GH

AD - Department of Medical and Molecular Genetics, Indiana University School of

Medicine, Indianapolis, Indiana, United States.

FAU - Weaver, David D

AU - Weaver DD

AUID- ORCID: 0000-0002-8312-8995

AD - Department of Medical and Molecular Genetics, Indiana University School of

Medicine, Indianapolis, Indiana, United States.

LA - eng

PT - Journal Article

DEP - 20200820

TA - J Pediatr Genet

JT - Journal of pediatric genetics

JID - 101589859

PMC - PMC8416204

OTO - NOTNLM

OT - Klinefelter syndrome

OT - X-linked ichthyosis

OT - interstitial Xp22.3 deletion

OT - maternal nondisjunction

OT - status epilepticus

OT - stroke

OT - uniparental disomy

COIS- Conflict of Interest None declared.

EDAT- 2021/09/11 06:00

MHDA- 2021/09/11 06:01

PMCR- 2022/09/01

CRDT- 2021/09/10 07:02

PHST- 2020/05/21 00:00 [received]

PHST- 2020/07/08 00:00 [accepted]

PHST- 2022/09/01 00:00 [pmc-release]

PHST- 2021/09/10 07:02 [entrez]

PHST- 2021/09/11 06:00 [pubmed]

PHST- 2021/09/11 06:01 [medline]

AID - 2000073 [pii]

AID - 10.1055/s-0040-1715573 [doi]

PST - ppublish

SO - J Pediatr Genet. 2021 Sep;10(3):222-229. doi: 10.1055/s-0040-1715573. Epub 2020

Aug 20.

PMID- 17932453

OWN - NLM

STAT- MEDLINE

DCOM- 20080812

LR - 20131121

IS - 1883-0498 (Electronic)

IS - 0023-2513 (Linking)

VI - 53

IP - 4

DP - 2007

TI - Mosaic tetrasomy 9p case with the phenotype mimicking Klinefelter syndrome and

hyporesponse of gonadotropin-stimulated testosterone production.

PG - 143-50

AB - Tetrasomy 9p is a rare clinical syndrome and about 30% of known cases exhibit

chromosome mosaicism. The cases with tetrasomy 9p mosaicism have been reported to

show the various phenotypes. On the other hand, Klinefelter syndrome is well

recognized chromosomal abnormality caused by an additional X chromosome in males

(47,XXY), and the characteristic clinical findings include tall stature,

immaturity of external genitalia, testicular dysfunction. Here, we report a

10-year-old male with tetrasomy of 9p mosaicism, whose phenotypic feature is

mimicking Klinefelter syndrome. He was referred to our hospital for inconspicuous

penis. He showed tall height (+2.5 SD). Endocrinological examination revealed the

poor testosterone response to human chorionic gonadotropin administration, which

indicated the testicular hypofunction, whereas MRI revealed concealed penis as a

cause of inconspicuous penis. Because of the phenotype mimicking Klinefelter

syndrome, karyotype of his blood lymphocytes was analyzed, and an additional

marker chromosome was detected in 6% of the investigated metaphases. Fluorescence

in situ hybridization analysis revealed that the marker chromosome was an

isochromosome 9p, which resulted in tetrasomy 9p. Chromosome analysis of buccal

smear also showed mosaicism for two karyotypes: 5% of cells had the isochromosome

of 9p, and the other cells showed normal. This case is the second case with

tetrasomy 9p mosaicism mimicking Klinefelter syndrome phenotype in the world. Our

case, together with previously reported cases with the same association,

indicates the possibility of testicular hypofunction and urogenital anomalies

induced by overexpression of some genes on chromosome 9p.

FAU - Ogino, Wakako

AU - Ogino W

AD - Department of Pediatrics, Kobe University Graduate School of Medicine, Kobe,

Japan.

FAU - Takeshima, Yasuhiro

AU - Takeshima Y

FAU - Nishiyama, Atsushi

AU - Nishiyama A

FAU - Yagi, Mariko

AU - Yagi M

FAU - Oka, Nobutoshi

AU - Oka N

FAU - Matsuo, Masafumi

AU - Matsuo M

LA - eng

PT - Case Reports

PT - Journal Article

PT - Review

PL - Japan

TA - Kobe J Med Sci

JT - The Kobe journal of medical sciences

JID - 0413531

RN - 0 (Chorionic Gonadotropin)

RN - 3XMK78S47O (Testosterone)

SB - IM

MH - *Aneuploidy

MH - Child

MH - Chorionic Gonadotropin/*physiology

MH - Chromosomes, Human, Pair 9/*genetics

MH - Diagnosis, Differential

MH - Humans

MH - Karyotyping

MH - Klinefelter Syndrome/diagnosis/*genetics/pathology

MH - Male

MH - *Mosaicism

MH - *Phenotype

MH - Testosterone/*biosynthesis

RF - 38

EDAT- 2007/10/13 09:00

MHDA- 2008/08/13 09:00

CRDT- 2007/10/13 09:00

PHST- 2007/10/13 09:00 [pubmed]

PHST- 2008/08/13 09:00 [medline]

PHST- 2007/10/13 09:00 [entrez]

PST - ppublish

SO - Kobe J Med Sci. 2007;53(4):143-50.

PMID- 32432406

OWN - NLM

STAT- MEDLINE

DCOM- 20210505

LR - 20210505

IS - 1552-4876 (Electronic)

IS - 1552-4868 (Linking)

VI - 184

IP - 2

DP - 2020 Jun

TI - 41,XX(Y) * male mice: An animal model for Klinefelter syndrome.

PG - 267-278

LID - 10.1002/ajmg.c.31796 [doi]

AB - Klinefelter syndrome (KS, 47,XXY) is the most frequent male chromosomal

aneuploidy resulting in a highly heterogeneous clinical phenotype associated with

hormonal dysbalance, increased rate of co-morbidities, and reduced lifespan. Two

hallmarks of KS-affecting testicular functions are consistently observed:

Hypergonadotropic hypogonadism and germ cell (GC) loss resulting in infertility.

Although KS is being studied for decades, the underlying mechanisms for the

observed pathophysiology are still unclear. Due to ethical restrictions, studies

in humans are limited, and consequently, suitable animal models are needed to

address the consequences of a supernumerary X chromosome. Mouse strains with

comparable aneuploidies have been generated and yielded highly relevant insights

into KS. We briefly describe the establishment of the KS mouse models, summarize

the knowledge gained by their use, compare findings from the mouse models to

those obtained in clinical studies, and also reflect on limitations of the

currently used models derived from the B6Ei.Lt-Y* mouse strain, in which the Y

chromosome is altered and its centromere position changed into a more distal

location provoking meiotic non-disjunction. Breeding such as XY* males to XX

females, the target 41,XX(Y) *, and 41,XXY males are generated. Here, we

summarize features of both models but report in particular findings from our

41,XX(Y) * mice including some novel data on Sertoli cell characteristics.

CI - © 2020 The Authors. American Journal of Medical Genetics Part C: Seminars in

Medical Genetics published by Wiley Periodicals, LLC.

FAU - Wistuba, Joachim

AU - Wistuba J

AUID- ORCID: 0000-0001-9215-8582

AD - Institute of Reproductive and Regenerative Biology, Centre of Reproductive

Medicine and Andrology, University of Münster, Münster, Germany.

FAU - Beumer, Cristin

AU - Beumer C

AD - Institute of Reproductive and Regenerative Biology, Centre of Reproductive

Medicine and Andrology, University of Münster, Münster, Germany.

FAU - Brehm, Ralph

AU - Brehm R

AD - Functional Histology and Cell Biology, Institute for Anatomy, University of

Veterinary Medicine Hannover, Foundation, Hannover, Germany.

FAU - Gromoll, Jörg

AU - Gromoll J

AD - Institute of Reproductive and Regenerative Biology, Centre of Reproductive

Medicine and Andrology, University of Münster, Münster, Germany.

LA - eng

PT - Journal Article

PT - Research Support, Non-U.S. Gov't

PT - Review

DEP - 20200520

PL - United States

TA - Am J Med Genet C Semin Med Genet

JT - American journal of medical genetics. Part C, Seminars in medical genetics

JID - 101235745

SB - IM

MH - *Aneuploidy

MH - Animals

MH - Disease Models, Animal

MH - Female

MH - Humans

MH - Karyotyping

MH - Klinefelter Syndrome/*genetics/pathology

MH - Male

MH - Mice

MH - X Chromosome/*genetics

OTO - NOTNLM

OT - *41,XXY* mouse

OT - *Klinefelter syndrome

OT - *Sertoli cell

OT - *chromosomal imbalance

OT - *germ cell loss

EDAT- 2020/05/21 06:00

MHDA- 2021/05/06 06:00

CRDT- 2020/05/21 06:00

PHST- 2020/03/21 00:00 [received]

PHST- 2020/04/28 00:00 [revised]

PHST- 2020/04/29 00:00 [accepted]

PHST- 2020/05/21 06:00 [pubmed]

PHST- 2021/05/06 06:00 [medline]

PHST- 2020/05/21 06:00 [entrez]

AID - 10.1002/ajmg.c.31796 [doi]

PST - ppublish

SO - Am J Med Genet C Semin Med Genet. 2020 Jun;184(2):267-278. doi:

10.1002/ajmg.c.31796. Epub 2020 May 20.

PMID- 22846647

OWN - NLM

STAT- MEDLINE

DCOM- 20121011

LR - 20120731

IS - 1556-5653 (Electronic)

IS - 0015-0282 (Linking)

VI - 98

IP - 2

DP - 2012 Aug

TI - The natural history of endocrine function and spermatogenesis in Klinefelter

syndrome: what the data show.

PG - 266-73

LID - 10.1016/j.fertnstert.2012.06.024 [doi]

AB - Once thought to be a chromosomal aberration associated with absolute sterility,

Klinefelter syndrome may now be potentially treatable by testicular sperm

retrieval coupled with intracytoplasmic sperm injection. With these therapeutic

advances, azoospermic 47,XXY men now may have an opportunity for biological

paternity. However, our knowledge of the basic mechanisms underlying germ cell

loss and Leydig cell compromise is lagging, and is just now beginning to evolve

and provide answers to some of the field's most vexing questions: how to maximize

and preserve fertility in Klinefelter males many years or even decades before

they wish to actively pursue fatherhood. This article reviews the development of

the androgenic and spermatogenic compartments of the Klinefelter testis through

puberty, and recommends that it is only with a clear understanding of the basic

facts that a rational, considered approach to fertility optimization and

preservation can be determined.

CI - Copyright © 2012 American Society for Reproductive Medicine. Published by

Elsevier Inc. All rights reserved.

FAU - Oates, Robert D

AU - Oates RD

AD - School of Medicine, Boston University, Boston, Massachusetts, USA.

robert.oates@bmc.org

LA - eng

PT - Journal Article

PT - Review

PL - United States

TA - Fertil Steril

JT - Fertility and sterility

JID - 0372772

SB - IM

MH - Animals

MH - Azoospermia/diagnosis/physiopathology/therapy

MH - Endocrine System/*physiology

MH - Fertility Preservation/methods/trends

MH - Humans

MH - Klinefelter Syndrome/diagnosis/*physiopathology/*therapy

MH - Male

MH - Sperm Retrieval/trends

MH - Spermatogenesis/*physiology

EDAT- 2012/08/01 06:00

MHDA- 2012/10/12 06:00

CRDT- 2012/08/01 06:00

PHST- 2012/05/04 00:00 [received]

PHST- 2012/06/18 00:00 [revised]

PHST- 2012/06/18 00:00 [accepted]

PHST- 2012/08/01 06:00 [entrez]

PHST- 2012/08/01 06:00 [pubmed]

PHST- 2012/10/12 06:00 [medline]

AID - S0015-0282(12)00677-2 [pii]

AID - 10.1016/j.fertnstert.2012.06.024 [doi]

PST - ppublish

SO - Fertil Steril. 2012 Aug;98(2):266-73. doi: 10.1016/j.fertnstert.2012.06.024.

PMID- 26075116

OWN - NLM

STAT- PubMed-not-MEDLINE

DCOM- 20150615

LR - 20200930

IS - 2090-6544 (Print)

IS - 2090-6552 (Electronic)

IS - 2090-6552 (Linking)

VI - 2015

DP - 2015

TI - Cognitive, Affective Problems and Renal Cross Ectopy in a Patient with

48,XXYY/47,XYY Syndrome.

PG - 950574

LID - 10.1155/2015/950574 [doi]

LID - 950574

AB - Klinefelter syndrome is the most common sex chromosome abnormality (SCA) in

infertile patients and 47,XXY genomic configuration constitutes most of the

cases. However, additional Xs and/or Y such as 48,XXYY, 48,XXXY, and 47,XYY can

occur less frequently than 47,XXY. Those configurations were considered as

variants of Klinefelter syndrome. In this report, we present an infertile man

with tall stature and decreased testicular volume. Semen analysis and hormonal

evaluation supported the diagnosis of nonobstructive azoospermia. Genetic

investigation demonstrated an abnormal male karyotype with two X chromosomes and

two Y chromosomes consistent with 48,XXYY(17)/47,XYY (13). Additionally, the

patient expressed cognitive and affective problems which were documented by

psychomotor retardation and borderline intelligence measured by an IQ value

between 70 and 80. Systemic evaluation also revealed cross ectopy and malrotation

of the right kidney in the patient. The couple was referred to microtesticular

sperm extraction (micro-TESE)/intracytoplasmic sperm injection (ICSI) cycles and

preimplantation genetic diagnosis (PGD). To the best of our knowledge, this is

the first report of combination of XYY and XXYY syndromes associated with

cognitive, affective dysfunction and renal malrotation.

FAU - Resim, Sefa

AU - Resim S

AD - Department of Urology, Kahramanmaras Sutcu Imam University, Kahramanmaras,

Turkey.

FAU - Kucukdurmaz, Faruk

AU - Kucukdurmaz F

AD - Department of Urology, Nizip State Hospital, Gaziantep, Turkey.

FAU - Kankılıc, Nazım

AU - Kankılıc N

AD - Department of Urology, Kahramanmaras Sutcu Imam University, Kahramanmaras,

Turkey.

FAU - Altunoren, Ozlem

AU - Altunoren O

AD - Department of Psychiatry, Kahramanmaras State Hospital, Kahramanmaras, Turkey.

FAU - Efe, Erkan

AU - Efe E

AD - Department of Urology, Kahramanmaras Sutcu Imam University, Kahramanmaras,

Turkey.

FAU - Benlioglu, Can

AU - Benlioglu C

AD - Department of Urology, Adiyaman University, Adiyaman, Turkey.

LA - eng

PT - Journal Article

DEP - 20150505

TA - Case Rep Genet

JT - Case reports in genetics

JID - 101583302

PMC - PMC4436470

EDAT- 2015/06/16 06:00

MHDA- 2015/06/16 06:01

CRDT- 2015/06/16 06:00

PHST- 2015/03/10 00:00 [received]

PHST- 2015/04/27 00:00 [accepted]

PHST- 2015/06/16 06:00 [entrez]

PHST- 2015/06/16 06:00 [pubmed]

PHST- 2015/06/16 06:01 [medline]

AID - 10.1155/2015/950574 [doi]

PST - ppublish

SO - Case Rep Genet. 2015;2015:950574. doi: 10.1155/2015/950574. Epub 2015 May 5.

PMID- 572319

OWN - NLM

STAT- MEDLINE

DCOM- 19790917

LR - 20191021

IS - 0015-5721 (Print)

IS - 0015-5721 (Linking)

VI - 33

IP - 1

DP - 1979

TI - Personality traits of 47,XYY and 47,XXY males found among juvenile delinquents.

PG - 29-34

AB - The mental features of nine 47,XYY males and six 47,XXY males found among

Japanese juvenile delinquents were examined. The results of intelligence tests

suggested a slight impairment among 47,XYY and 47,XXY males. The psychiatric

interviews and the psychological tests revealed that 47,XYY males had a high

activity level while 47,XXY males had a low one. Analyses of the offense type and

psychophysiological features supported these findings. As the activity level is

considered to be largely determined genetically, the extra Y is suspected to be

responsible for the high activity level and the extra X in males to be

responsible for the low activity level.

FAU - Nanko, S

AU - Nanko S

LA - eng

PT - Journal Article

PL - Japan

TA - Folia Psychiatr Neurol Jpn

JT - Folia psychiatrica et neurologica japonica

JID - 0372774

SB - IM

MH - Adolescent

MH - Adult

MH - *Antisocial Personality Disorder

MH - Child

MH - Criminal Psychology

MH - Female

MH - Humans

MH - Intelligence Tests

MH - Juvenile Delinquency/*psychology

MH - Male

MH - Psychophysiologic Disorders

MH - Sex Chromosome Aberrations/*psychology

MH - X Chromosome

MH - XYY Karyotype/*psychology

MH - Y Chromosome

EDAT- 1979/01/01 00:00

MHDA- 1979/01/01 00:01

CRDT- 1979/01/01 00:00

PHST- 1979/01/01 00:00 [pubmed]

PHST- 1979/01/01 00:01 [medline]

PHST- 1979/01/01 00:00 [entrez]

AID - 10.1111/j.1440-1819.1979.tb00171.x [doi]

PST - ppublish

SO - Folia Psychiatr Neurol Jpn. 1979;33(1):29-34. doi:

10.1111/j.1440-1819.1979.tb00171.x.

PMID- 34431088

OWN - NLM

STAT- MEDLINE

DCOM- 20220218

LR - 20220302

IS - 1469-8749 (Electronic)

IS - 0012-1622 (Print)

IS - 0012-1622 (Linking)

VI - 64

IP - 3

DP - 2022 Mar

TI - Effect of sex chromosome number variation on attention-deficit/hyperactivity

disorder symptoms, executive function, and processing speed.

PG - 331-339

LID - 10.1111/dmcn.15020 [doi]

AB - AIM: To study sex differences in attention-deficit/hyperactivity disorder (ADHD)

symptoms, we explored whether X chromosome absence or excess is independently

associated with deficits in attention and hyperactivity, executive function, and

processing speed. METHOD: We assessed 116 children (ages 3y 10mo-11y 11mo, mean

8y 5mo, SD 1y 11mo) with a variable number of sex chromosomes: 36 females with

Turner syndrome (45, X0), 20 males with Klinefelter syndrome (47, XXY), 37

typically developing females (XX), and 23 typically developing males (XY).

RESULTS: X chromosome absence was associated with increased attention problems,

hyperactivity, and deficits in inhibitory control, compared with female children

with XX (all p<0.003). Conversely, X chromosome excess was associated with

weakness in working memory (p=0.018) and approached significance for attention

problems (p=0.071) but not with hyperactivity, or weakness in inhibitory control

relative to male children with XY. Using non-parametric effect size to quantify

the clinical effect revealed that X chromosome absence affected attention,

hyperactivity, executive function, and processing speed (all r>0.4), while X

excess affected in-laboratory as well as parent-reported working memory (all

r>0.4). INTERPRETATION: Our observations provide compelling evidence that the

absence or excess of an X chromosome distinctly affects cognition and behaviors

associated with ADHD.

CI - © 2021 Mac Keith Press.

FAU - Green, Tamar

AU - Green T

AUID- ORCID: 0000-0001-5661-8297

AD - Division of Interdisciplinary Brain Sciences, Stanford University School of

Medicine, Stanford, CA, USA.

AD - Department of Psychiatry and Behavioral Sciences, Stanford University School of

Medicine, Stanford, CA, USA.

FAU - Flash, Shira

AU - Flash S

AD - Division of Interdisciplinary Brain Sciences, Stanford University School of

Medicine, Stanford, CA, USA.

AD - Department of Psychiatry and Behavioral Sciences, Stanford University School of

Medicine, Stanford, CA, USA.

FAU - Shankar, Geeta

AU - Shankar G

AUID- ORCID: 0000-0003-2403-2114

AD - Division of Interdisciplinary Brain Sciences, Stanford University School of

Medicine, Stanford, CA, USA.

AD - Department of Psychiatry and Behavioral Sciences, Stanford University School of

Medicine, Stanford, CA, USA.

FAU - Bade Shrestha, Sharon

AU - Bade Shrestha S

AD - Division of Interdisciplinary Brain Sciences, Stanford University School of

Medicine, Stanford, CA, USA.

AD - Department of Psychiatry and Behavioral Sciences, Stanford University School of

Medicine, Stanford, CA, USA.

FAU - Jo, Booil

AU - Jo B

AD - Division of Interdisciplinary Brain Sciences, Stanford University School of

Medicine, Stanford, CA, USA.

AD - Department of Psychiatry and Behavioral Sciences, Stanford University School of

Medicine, Stanford, CA, USA.

FAU - Klabunde, Megan

AU - Klabunde M

AUID- ORCID: 0000-0003-4210-7968

AD - Department of Psychology, University of Essex, Colchester, UK.

FAU - Hong, David S

AU - Hong DS

AD - Division of Interdisciplinary Brain Sciences, Stanford University School of

Medicine, Stanford, CA, USA.

AD - Department of Psychiatry and Behavioral Sciences, Stanford University School of

Medicine, Stanford, CA, USA.

FAU - Reiss, Allan L

AU - Reiss AL

AD - Division of Interdisciplinary Brain Sciences, Stanford University School of

Medicine, Stanford, CA, USA.

AD - Department of Psychiatry and Behavioral Sciences, Stanford University School of

Medicine, Stanford, CA, USA.

AD - Department of Radiology, Stanford University School of Medicine, Stanford, CA,

USA.

AD - Department of Pediatrics, Stanford University School of Medicine, Stanford, CA,

USA.

LA - eng

GR - R21 MH099630/MH/NIMH NIH HHS/United States

GR - R01 HD092847/HD/NICHD NIH HHS/United States

GR - K23 HD090209/HD/NICHD NIH HHS/United States

GR - R01 HD049653/HD/NICHD NIH HHS/United States

GR - T32 MH019908/MH/NIMH NIH HHS/United States

GR - K23 MH097120/MH/NIMH NIH HHS/United States

PT - Journal Article

PT - Research Support, N.I.H., Extramural

PT - Research Support, Non-U.S. Gov't

DEP - 20210824

TA - Dev Med Child Neurol

JT - Developmental medicine and child neurology

JID - 0006761

SB - IM

MH - Attention Deficit Disorder with Hyperactivity/*genetics/*physiopathology

MH - Child

MH - Child, Preschool

MH - Chromosomes, Human, X/*genetics

MH - Executive Function/*physiology

MH - Female

MH - Humans

MH - *Inhibition, Psychological

MH - Klinefelter Syndrome/genetics/physiopathology

MH - Male

MH - Memory, Short-Term/*physiology

MH - Psychomotor Performance/*physiology

MH - *Sex Characteristics

MH - Turner Syndrome/genetics/physiopathology

PMC - PMC8816867

MID - NIHMS1762802

EDAT- 2021/08/26 06:00

MHDA- 2022/02/19 06:00

CRDT- 2021/08/25 06:36

PHST- 2021/07/15 00:00 [revised]

PHST- 2020/06/24 00:00 [received]

PHST- 2021/07/16 00:00 [accepted]

PHST- 2021/08/26 06:00 [pubmed]

PHST- 2022/02/19 06:00 [medline]

PHST- 2021/08/25 06:36 [entrez]

AID - 10.1111/dmcn.15020 [doi]

PST - ppublish

SO - Dev Med Child Neurol. 2022 Mar;64(3):331-339. doi: 10.1111/dmcn.15020. Epub 2021

Aug 24.

PMID- 25684214

OWN - NLM

STAT- MEDLINE

DCOM- 20151223

LR - 20150326

IS - 1601-183X (Electronic)

IS - 1601-183X (Linking)

VI - 14

IP - 2

DP - 2015 Feb

TI - Executive dysfunction and the relation with behavioral problems in children with

47,XXY and 47,XXX.

PG - 200-8

LID - 10.1111/gbb.12203 [doi]

AB - Neuroimaging studies have shown that having an extra X chromosome is associated

with abnormal structure and function of brain areas in the frontal lobe, which is

crucially involved in executive functioning. However, there is little of

knowledge of the type and severity of executive dysfunction, and the impact on

emotional and behavioral problems. The present study aims to provide in this. In

total, 40 children (23 boys with 47,XXY and 17 girls with 47,XXX) with an extra X

chromosome and 100 non-clinical controls (47 boys and 53 girls) participated in

the study. The participants were 9-18 years old. Processing speed and executive

functioning were assessed using the Amsterdam Neuropsychological Testbattery

(ANT) and the Dysexecutive Questionnaire (DEX). Problems in emotional and

behavioral functioning were assessed with the Childhood Behavior Checklist

(CBCL). Children with an extra X chromosome showed deficits in inhibition, mental

flexibility, sustained attention and visual working memory. Parental report

showed high levels of everyday manifestations of executive dysfunction. More

severe inhibition difficulties were associated with higher levels of thought

problems, aggression and rule breaking behavior. Boys and girls with an extra X

chromosome could not be differentiated based on severity of executive

dysfunction, however, girls had lower information processing speed than boys.

These findings suggest that executive dysfunction may be part of the phenotype of

children with an extra X chromosome, impacting the ability to function adequately

in everyday life. Furthermore, children with impairments in inhibition may have

more problems in regulating their thinking, emotions and behavior.

CI - © 2015 John Wiley & Sons Ltd and International Behavioural and Neural Genetics

Society.

FAU - van Rijn, S

AU - van Rijn S

AD - Clinical Child and Adolescent Studies, Leiden University, Leiden, The

Netherlands; Leiden Institute for Brain and Cognition, Leiden, The Netherlands.

FAU - Swaab, H

AU - Swaab H

LA - eng

PT - Journal Article

PL - England

TA - Genes Brain Behav

JT - Genes, brain, and behavior

JID - 101129617

RN - Triple X syndrome

SB - IM

MH - *Aggression

MH - Brain/physiopathology

MH - Child

MH - Chromosomes, Human, X/genetics

MH - Cognition Disorders/*genetics

MH - *Executive Function/physiology

MH - Female

MH - Humans

MH - Klinefelter Syndrome/*genetics

MH - Male

MH - Neuropsychological Tests

MH - *Problem Behavior/psychology

MH - Sex Chromosome Aberrations

MH - Sex Chromosome Disorders of Sex Development/*genetics

MH - Trisomy/*genetics

OTO - NOTNLM

OT - Behavioral problems

OT - Klinefelter syndrome

OT - Trisomy X

OT - cognition

OT - executive functions

OT - sex chromosome disorders

EDAT- 2015/02/17 06:00

MHDA- 2015/12/24 06:00

CRDT- 2015/02/17 06:00

PHST- 2014/09/16 00:00 [received]

PHST- 2015/01/16 00:00 [revised]

PHST- 2015/01/20 00:00 [accepted]

PHST- 2015/02/17 06:00 [entrez]

PHST- 2015/02/17 06:00 [pubmed]

PHST- 2015/12/24 06:00 [medline]

AID - 10.1111/gbb.12203 [doi]

PST - ppublish

SO - Genes Brain Behav. 2015 Feb;14(2):200-8. doi: 10.1111/gbb.12203.

PMID- 22320868

OWN - NLM

STAT- MEDLINE

DCOM- 20130422

LR - 20131121

IS - 1442-2042 (Electronic)

IS - 0919-8172 (Linking)

VI - 19

IP - 5

DP - 2012 May

TI - Clinical and social characteristics of Korean men with Klinefelter syndrome.

PG - 443-9

LID - 10.1111/j.1442-2042.2012.02964.x [doi]

AB - OBJECTIVES: To investigate the characteristics of Klinefelter syndrome in a

consecutive series of men consulting for sexual as well as fertility problems in

Korea. METHODS: A total of 179 men with non-mosaic 47,XXY Klinefelter syndrome of

the 1876 azoospermic males who visited the fertility center of our university

hospital between January 2002 and January 2010 were included in this analysis.

Their semen characteristics, hormone profiles, size of the prostate gland, and

education level were assessed and compared with an age-matched control group of

218 fertile males. Additionally, a histological evaluation was carried out.

RESULTS: Prostate size, testicular volume, semen volume and symptom severity

showed statistically significant differences in both groups (P < 0.0001). As the

severity of signs and symptoms worsened, the education level decreased. Each

degree of signs and symptoms was associated with a different level of education,

as well as with significant differences in the level of testosterone and

testicular volume (P < 0.05). Spearman's correlation coefficient showed the

severity of signs and symptoms was associated with testosterone (P < 0.0001).

CONCLUSIONS: Testosterone has the strongest negative association with the

severity of signs and symptoms in patients with Klinefelter syndrome. It

influences the reproductive capacity, as well as the manifested signs and

symptoms of hypogonadism. Furthermore, it is also associated with various aspects

of life in these patients.

CI - © 2012 The Japanese Urological Association.

FAU - Bak, Chong Won

AU - Bak CW

AD - Department of Andrology, Center for Reproductive Medicine, CHA University, Seoul,

Korea.

FAU - Byun, Jae Sang

AU - Byun JS

FAU - Lee, Jee Han

AU - Lee JH

FAU - Park, Ji Hye

AU - Park JH

FAU - Lee, Kyung-Ah

AU - Lee KA

FAU - Shim, Sung Han

AU - Shim SH

LA - eng

PT - Journal Article

PT - Research Support, Non-U.S. Gov't

DEP - 20120209

PL - Australia

TA - Int J Urol

JT - International journal of urology : official journal of the Japanese Urological

Association

JID - 9440237

RN - 3XMK78S47O (Testosterone)

SB - IM

MH - Adult

MH - Educational Status

MH - Humans

MH - *Klinefelter Syndrome/blood/classification

MH - Male

MH - Organ Size

MH - Prostate/anatomy & histology

MH - Republic of Korea

MH - Semen Analysis

MH - Severity of Illness Index

MH - Sociology/*statistics & numerical data

MH - Testosterone/blood

MH - Young Adult

EDAT- 2012/02/11 06:00

MHDA- 2013/04/23 06:00

CRDT- 2012/02/11 06:00

PHST- 2012/02/11 06:00 [entrez]

PHST- 2012/02/11 06:00 [pubmed]

PHST- 2013/04/23 06:00 [medline]

AID - 10.1111/j.1442-2042.2012.02964.x [doi]

PST - ppublish

SO - Int J Urol. 2012 May;19(5):443-9. doi: 10.1111/j.1442-2042.2012.02964.x. Epub

2012 Feb 9.

PMID- 20332707

OWN - NLM

STAT- MEDLINE

DCOM- 20110516

LR - 20211020

IS - 1720-8386 (Electronic)

IS - 0391-4097 (Linking)

VI - 33

IP - 11

DP - 2010 Dec

TI - Spermatogenesis in Klinefelter syndrome.

PG - 789-93

AB - BACKGROUND: Klinefelter syndrome (KS) (47,XXY) is the most common sex chromosomal

disorder, and it is a frequent form of male hypogonadism and infertility.

Although the majority of these patients are azoospermic, they might have severe

oligozoospermia or residual single-residual foci with spermatogenesis in the

testis. AIM: We report our experience on sperm retrieval in the ejaculate and

testis, and evaluate the frequency of chromosome abnormalities in sperm of KS.

SUBJECTS AND METHODS: Eighty-four 47,XXY KS were evaluated with seminal analysis,

body hair distribution, reproductive hormones, ultrasonographic scanning of the

testis and prostate, bilateral testicular sperm extraction (TESE), sperm or

testicular cells sex chromosomes aneuploidies. RESULTS: Out of 84 patients, 7

(7/84; 8.3%) had sperm in the ejaculate. Out of the 77 azoospermic patients, 24

underwent TESE and 9 (9/24; 37.5%) had successful sperm recovery. The comparison

of reproductive hormones, age and testicular volume did not show significant

differences between patients with and without successful sperm recovery in semen

or TESE . Patients without successful sperm recovery in semen analysis or TESE

had signs of hypoandrogenism more evident than patients with successful sperm

recovery. Patients with KS produced a higher number of sperm aneuploidy with

respect to normozoospermic fertile controls and non-genetic severely

oligozoospermic men. CONCLUSIONS: Men with KS are not always sterile. In some of

these patients sperm can be found in semen or in the testis, but the proportion

of sperm aneuploidy is high. Signs of hypoandrogenism seem to be associated with

low sperm recovery rate.

FAU - Selice, R

AU - Selice R

AD - Department of Histology, Microbiology and Medical Biotechnologies, Section of

Clinical Pathology & Center for Male Gamete Cryopreservation, University of

Padua, Via Gabelli 63, Padua, Italy.

FAU - Di Mambro, A

AU - Di Mambro A

FAU - Garolla, A

AU - Garolla A

FAU - Ficarra, V

AU - Ficarra V

FAU - Iafrate, M

AU - Iafrate M

FAU - Ferlin, A

AU - Ferlin A

FAU - Foresta, C

AU - Foresta C

LA - eng

PT - Journal Article

DEP - 20100322

PL - Italy

TA - J Endocrinol Invest

JT - Journal of endocrinological investigation

JID - 7806594

SB - IM

CIN - J Endocrinol Invest. 2011 Jul-Aug;34(7):570. PMID: 21897109

MH - Adolescent

MH - Adult

MH - Aneuploidy

MH - Chromosome Aberrations

MH - Humans

MH - Klinefelter Syndrome/*complications/genetics/pathology

MH - Male

MH - Middle Aged

MH - Oligospermia/etiology/genetics

MH - Retrospective Studies

MH - Semen Analysis

MH - Sperm Retrieval

MH - Spermatogenesis/genetics/*physiology

MH - Testis/pathology

EDAT- 2010/03/25 06:00

MHDA- 2011/05/17 06:00

CRDT- 2010/03/25 06:00

PHST- 2010/03/25 06:00 [entrez]

PHST- 2010/03/25 06:00 [pubmed]

PHST- 2011/05/17 06:00 [medline]

AID - 6935 [pii]

AID - 10.1007/BF03350343 [doi]

PST - ppublish

SO - J Endocrinol Invest. 2010 Dec;33(11):789-93. doi: 10.1007/BF03350343. Epub 2010

Mar 22.

PMID- 33096382

OWN - NLM

STAT- MEDLINE

DCOM- 20210621

LR - 20210621

IS - 1876-7753 (Electronic)

IS - 1873-5061 (Linking)

VI - 49

DP - 2020 Dec

TI - Derivation of two naturally isogenic iPSC lines (KAUSTi006-A and KAUSTi006-B)

from a mosaic Klinefelter Syndrome patient (47-XXY/46-XY).

PG - 102049

LID - S1873-5061(20)30350-0 [pii]

LID - 10.1016/j.scr.2020.102049 [doi]

AB - While Klinefelter Syndrome (KS) has a prevalence of 85-250 per 100,000 born

males, patients are typically underdiagnosed due to a subtle phenotype emerging

only late during puberty or adulthood. Rare cases of KS carry a mosaic phenotype

47-XXY/46-XY associated to mild phenotypic traits mostly compatible with a normal

life including preserved fertility. From a genetic modeling perspective, the

derivation of naturally isogenic iPSCs from mosaic patients allows the comparison

of disease and healthy cells carrying a virtually identical genomic background.

CI - Copyright © 2020 The Authors. Published by Elsevier B.V. All rights reserved.

FAU - Fiacco, Elisabetta

AU - Fiacco E

AD - Biological and Environmental Science and Engineering Division, King Abdullah

University of Science and Technology, Thuwal 23955-6900, Saudi Arabia.

FAU - Alowaysi, Maryam

AU - Alowaysi M

AD - Biological and Environmental Science and Engineering Division, King Abdullah

University of Science and Technology, Thuwal 23955-6900, Saudi Arabia.

FAU - Astro, Veronica

AU - Astro V

AD - Biological and Environmental Science and Engineering Division, King Abdullah

University of Science and Technology, Thuwal 23955-6900, Saudi Arabia.

FAU - Adamo, Antonio

AU - Adamo A

AD - Biological and Environmental Science and Engineering Division, King Abdullah

University of Science and Technology, Thuwal 23955-6900, Saudi Arabia. Electronic

address: antonio.adamo@kaust.edu.sa.

LA - eng

PT - Journal Article

PT - Research Support, Non-U.S. Gov't

DEP - 20201015

PL - England

TA - Stem Cell Res

JT - Stem cell research

JID - 101316957

SB - IM

MH - Adult

MH - Humans

MH - *Induced Pluripotent Stem Cells

MH - *Klinefelter Syndrome/genetics

MH - Male

MH - Mosaicism

MH - Puberty

EDAT- 2020/10/24 06:00

MHDA- 2021/06/22 06:00

CRDT- 2020/10/23 20:14

PHST- 2020/09/07 00:00 [received]

PHST- 2020/10/05 00:00 [revised]

PHST- 2020/10/11 00:00 [accepted]

PHST- 2020/10/24 06:00 [pubmed]

PHST- 2021/06/22 06:00 [medline]

PHST- 2020/10/23 20:14 [entrez]

AID - S1873-5061(20)30350-0 [pii]

AID - 10.1016/j.scr.2020.102049 [doi]

PST - ppublish

SO - Stem Cell Res. 2020 Dec;49:102049. doi: 10.1016/j.scr.2020.102049. Epub 2020 Oct

15.

PMID- 18324463

OWN - NLM

STAT- MEDLINE

DCOM- 20090114

LR - 20211020

IS - 0162-3257 (Print)

IS - 0162-3257 (Linking)

VI - 38

IP - 9

DP - 2008 Oct

TI - Social behavior and autism traits in a sex chromosomal disorder: Klinefelter

(47XXY) syndrome.

PG - 1634-41

LID - 10.1007/s10803-008-0542-1 [doi]

AB - Although Klinefelter syndrome (47,XXY) has been associated with psychosocial

difficulties, knowledge of the social behavioral phenotype is limited. We

examined specific social abilities and autism traits in Klinefelter syndrome.

Scores of 31 XXY men on the Scale for Interpersonal Behavior and the Autism

Spectrum Questionnaire were compared to 24 and 20 control men respectively. XXY

men reported increased distress during social interactions and less engagement in

specific social behaviors. In the XXY group, levels of autism traits were

significantly higher across all dimensions of the autism phenotype. These

findings call for a clinical investigation of vulnerability to autism in

Klinefelter syndrome. Klinefelter syndrome might serve as a model for studying a

role of the X chromosome in social behavioral dysfunction and autism-like

behavior.

FAU - van Rijn, Sophie

AU - van Rijn S

AD - Department of Experimental Psychology, Helmholtz Instituut, Universiteit Utrecht,

Utrecht, The Netherlands. srijn@fsw.leidenuniv.nl

FAU - Swaab, Hanna

AU - Swaab H

FAU - Aleman, André

AU - Aleman A

FAU - Kahn, René S

AU - Kahn RS

LA - eng

PT - Journal Article

PT - Research Support, Non-U.S. Gov't

DEP - 20080307

PL - United States

TA - J Autism Dev Disord

JT - Journal of autism and developmental disorders

JID - 7904301

SB - IM

MH - Adult

MH - Autistic Disorder/*diagnosis

MH - Chromosomes, Human, X/*genetics

MH - Cognition Disorders/diagnosis

MH - Depression/psychology

MH - Humans

MH - Interpersonal Relations

MH - Klinefelter Syndrome/*diagnosis/*genetics

MH - Male

MH - Neuropsychological Tests

MH - Psychometrics

MH - Severity of Illness Index

MH - *Social Behavior

MH - Surveys and Questionnaires

EDAT- 2008/03/08 09:00

MHDA- 2009/01/15 09:00

CRDT- 2008/03/08 09:00

PHST- 2007/02/01 00:00 [received]

PHST- 2008/01/07 00:00 [accepted]

PHST- 2008/03/08 09:00 [pubmed]

PHST- 2009/01/15 09:00 [medline]

PHST- 2008/03/08 09:00 [entrez]

AID - 10.1007/s10803-008-0542-1 [doi]

PST - ppublish

SO - J Autism Dev Disord. 2008 Oct;38(9):1634-41. doi: 10.1007/s10803-008-0542-1. Epub

2008 Mar 7.

PMID- 31231689

OWN - NLM

STAT- PubMed-not-MEDLINE

LR - 20200928

IS - 2398-502X (Print)

IS - 2398-502X (Electronic)

IS - 2398-502X (Linking)

VI - 4

DP - 2019

TI - Autism and social anxiety in children with sex chromosome trisomies: an

observational study.

PG - 32

LID - 10.12688/wellcomeopenres.15095.2 [doi]

LID - 32

AB - Background: Recent studies suggest that an extra sex chromosome increases the

risk of both autism and social anxiety, but it unclear whether these risks are

specific to particular karyotypes. Methods: We considered diagnostic data from an

online psychiatric assessment (DAWBA - The Development and Well-Being Assessment)

and questionnaire responses completed by parents of children with 47,XXX (N =

29), 47,XXY (N = 28) and 47,XYY (N = 32) karyotypes. Analysis focused mainly on

54 children who were diagnosed prenatally or on the basis of other medical

concerns in childhood (Low Bias subgroup), to minimise ascertainment bias.

Results: Children with symptoms of autism who fell short of meeting the

Diagnostic and Statistical Manual of Mental Disorders (DSM)-IV criteria were

coded as cases of Pervasive Developmental Disorder Not Otherwise Specified

(PDDNOS). The odds ratio of autism or PDDNOS in the Low Bias group was computed

relative to gender-specific population norms. This gave log odds ratio (95%

confidence interval) of 5.56 (4.25 - 6.88) for XXX girls; 4.00 (2.66 - 5.33) for

XXY boys; and 4.60 (3.46 - 5.74) for XYY boys. Despite this elevated risk, most

children had no autistic features. A diagnosis of DSM-IV Social Phobia was rare,

though, in line with prediction, all three Low Bias cases with this diagnosis had

47,XXY karyotype. All three trisomy groups showed increased risk of milder

symptoms of social anxiety. Conclusions: An increased risk of autism was found in

girls with 47,XXX karyotype, as well as in boys with 47,XXY or 47,XYY. Symptoms

of social anxiety were increased in all three karyotypes. There was wide

variation in psychiatric status of children with the same karyotype, suggesting

that an extra sex chromosome affects developmental stability in a non-specific

way, with a diverse range of possible phenotypes.

FAU - Wilson, Alexander C

AU - Wilson AC

AUID- ORCID: 0000-0001-7077-1618

AD - Department of Experimental Psychology, University of Oxford, Oxford, OX2 6GG, UK.

FAU - King, Judith

AU - King J

AD - Department of Psychiatry, University of Oxford, Oxford, UK.

FAU - Bishop, Dorothy V M

AU - Bishop DVM

AUID- ORCID: 0000-0002-2448-4033

AD - Department of Experimental Psychology, University of Oxford, Oxford, OX2 6GG, UK.

LA - eng

GR - Wellcome Trust/United Kingdom

PT - Journal Article

DEP - 20190902

TA - Wellcome Open Res

JT - Wellcome open research

JID - 101696457

PMC - PMC6567293

OTO - NOTNLM

OT - Autism

OT - DAWBA

OT - Klinefelter syndrome

OT - SRS

OT - XYY syndrome

OT - ascertainment bias

OT - social anxiety

OT - trisomy X

COIS- No competing interests were disclosed.

EDAT- 2019/09/06 06:00

MHDA- 2019/09/06 06:01

CRDT- 2019/09/06 06:00

PHST- 2019/08/27 00:00 [accepted]

PHST- 2019/09/06 06:00 [entrez]

PHST- 2019/09/06 06:00 [pubmed]

PHST- 2019/09/06 06:01 [medline]

AID - 10.12688/wellcomeopenres.15095.2 [doi]

PST - epublish

SO - Wellcome Open Res. 2019 Sep 2;4:32. doi: 10.12688/wellcomeopenres.15095.2.

eCollection 2019.

PMID- 9467998

OWN - NLM

STAT- MEDLINE

DCOM- 19980324

LR - 20220317

IS - 0305-182X (Print)

IS - 0305-182X (Linking)

VI - 24

IP - 12

DP - 1997 Dec

TI - Occlusal plane orientation in Klinefelter syndrome (47,XXY males).

PG - 942-6

AB - Occlusal plane position was analysed cephalometrically in 35 Klinefelter adults

(47,XXY) and compared with 60 eugnath control males (46,XY). The significantly

smaller angles between the occlusal plane and the cranial base (NSL-OLs) and

between the occlusal plane and the Frankfort horizontal plane (Fr-OLs) were

obtained in 47,XXY males (P < 0.01), while the angles between the maxillary base

and the occlusal plane (NL-OLs) and between the Camper's line and the occlusal

plane (Camp.-OLs) were not significantly different (P > 0.05) from the control

group. Significantly smaller angles between the occlusal plane and the cranial

base (NSL-OLs) and between the occlusal plane and the Frankfort horizontal plane

(Fr-OLs) in Klinefelter males are attributed to the hereditary influence of an

extra X chromosome on the smaller growth of the cranial base and the greater

growth of the lower border of the mandible. Although the maxilla was also shifted

forward in XXY males in relation to the cranial base it was not enough to

compensate for the hereditary influence, due to the greater growth of the lower

border of the mandible and the smaller cranial base in 47, XXY males, on the

inclination of the occlusal plane to the Frankfort horizontal plane and the

cranial base. The forward shift of the maxilla was sufficient to compensate for

the inclination of the occlusal plane in 47, XXY males to the maxillary base and

the Camper's line (P > 0.05).

FAU - Celebić, A

AU - Celebić A

AD - School of Dentistry, University of Zagreb, Croatia.

FAU - Brkić, H

AU - Brkić H

FAU - Kaić, Z

AU - Kaić Z

FAU - Vojvodić, D

AU - Vojvodić D

FAU - Poje, Z

AU - Poje Z

FAU - Singer, Z

AU - Singer Z

LA - eng

PT - Comparative Study

PT - Journal Article

PT - Research Support, Non-U.S. Gov't

PL - England

TA - J Oral Rehabil

JT - Journal of oral rehabilitation

JID - 0433604

SB - IM

MH - Adult

MH - Cephalometry

MH - *Dental Occlusion

MH - Humans

MH - Klinefelter Syndrome/*pathology/physiopathology

MH - Male

MH - Mandible/growth & development/pathology

MH - Maxilla/pathology

MH - Observer Variation

MH - Reproducibility of Results

MH - Sella Turcica/pathology

MH - Skull Base/growth & development/pathology

EDAT- 1998/02/19 00:00

MHDA- 1998/02/19 00:01

CRDT- 1998/02/19 00:00

PHST- 1998/02/19 00:00 [pubmed]

PHST- 1998/02/19 00:01 [medline]

PHST- 1998/02/19 00:00 [entrez]

AID - 10.1046/j.1365-2842.1997.00576.x [doi]

PST - ppublish

SO - J Oral Rehabil. 1997 Dec;24(12):942-6. doi: 10.1046/j.1365-2842.1997.00576.x.

PMID- 26746120

OWN - NLM

STAT- MEDLINE

DCOM- 20160802

LR - 20220408

IS - 1097-6833 (Electronic)

IS - 0022-3476 (Linking)

VI - 170

DP - 2016 Mar

TI - Sperm Retrieval in Adolescents and Young Adults with Klinefelter Syndrome: A

Prospective, Pilot Study.

PG - 260-5.e1-2

LID - S0022-3476(15)01540-1 [pii]

LID - 10.1016/j.jpeds.2015.12.028 [doi]

AB - OBJECTIVE: To assess sperm retrieval rates in adolescents and young adults with

Klinefelter syndrome, with the ultimate goal of improving fertility in this

population. Secondary aims were to evaluate other clinical characteristics of the

cohort and identify predictors of sperm retrieval. STUDY DESIGN: Patients 12-25

years of age with Klinefelter syndrome (47,XXY) were recruited at the Boston

Children's Hospital. Physical examination, biochemical evaluation, scrotal

ultrasonography, and semen analysis were performed. Neurocognitive data were

collected. Microdissection sperm extraction (unilateral micro-testicular sperm

extraction) was offered to individuals with no sperm in their ejaculates. Given

the small sample size, analysis was primarily descriptive. RESULTS: Fifteen

patients were enrolled. None had sperm in their ejaculates. Ten patients

underwent unilateral micro-testicular sperm extraction. Sperm retrieval rate was

50%. From a neurocognitive standpoint, subjects reported problems with peers,

conduct, and overall difficulties. Incidentally, one-third of the patients were

found to have testicular microlithiasis and 17% of subjects with renal ultrasound

imaging had bilateral renal medullary nephrocalcinosis. CONCLUSIONS: This pilot

study suggests that sperm retrieval rates in adolescents and young adults with

Klinefelter syndrome are comparable with those reported in older men. However,

larger studies are needed to confirm our findings. The clinical significance of

the scrotal and renal ultrasound findings merits further investigation. TRIAL

REGISTRATION: ClinicalTrials.gov: NCT01817296.

CI - Copyright © 2016 Elsevier Inc. All rights reserved.

FAU - Nahata, Leena

AU - Nahata L

AD - Division of Endocrinology, Nationwide Children's Hospital, Columbus, OH.

Electronic address: leena.nahata@nationwidechildrens.org.

FAU - Yu, Richard N

AU - Yu RN

AD - Department of Urology, Boston Children's Hospital, Boston, MA.

FAU - Paltiel, Harriet J

AU - Paltiel HJ

AD - Department of Radiology, Boston Children's Hospital, Boston, MA.

FAU - Chow, Jeanne S

AU - Chow JS

AD - Department of Radiology, Boston Children's Hospital, Boston, MA.

FAU - Logvinenko, Tanya

AU - Logvinenko T

AD - Department of Urology, Boston Children's Hospital, Boston, MA; Clinical Research

Center, Boston Children's Hospital, Boston, MA.

FAU - Rosoklija, Ilina

AU - Rosoklija I

AD - Department of Urology, Boston Children's Hospital, Boston, MA.

FAU - Cohen, Laurie E

AU - Cohen LE

AD - Division of Endocrinology, Boston Children's Hospital, Boston, MA.

LA - eng

SI - ClinicalTrials.gov/NCT01817296

PT - Clinical Trial

PT - Journal Article

PT - Research Support, Non-U.S. Gov't

DEP - 20151231

PL - United States

TA - J Pediatr

JT - The Journal of pediatrics

JID - 0375410

SB - IM

MH - Adolescent

MH - Adult

MH - Child

MH - Humans

MH - Infertility, Male/*diagnosis/etiology/therapy

MH - Klinefelter Syndrome/*complications/diagnosis

MH - Male

MH - Pilot Projects

MH - Prospective Studies

MH - *Sperm Retrieval

MH - Young Adult

EDAT- 2016/01/10 06:00

MHDA- 2016/08/03 06:00

CRDT- 2016/01/10 06:00

PHST- 2015/09/09 00:00 [received]

PHST- 2015/10/20 00:00 [revised]

PHST- 2015/12/07 00:00 [accepted]

PHST- 2016/01/10 06:00 [entrez]

PHST- 2016/01/10 06:00 [pubmed]

PHST- 2016/08/03 06:00 [medline]

AID - S0022-3476(15)01540-1 [pii]

AID - 10.1016/j.jpeds.2015.12.028 [doi]

PST - ppublish

SO - J Pediatr. 2016 Mar;170:260-5.e1-2. doi: 10.1016/j.jpeds.2015.12.028. Epub 2015

Dec 31.

PMID- 517519

OWN - NLM

STAT- MEDLINE

DCOM- 19800226

LR - 20200824

IS - 0002-9297 (Print)

IS - 1537-6605 (Electronic)

IS - 0002-9297 (Linking)

VI - 31

IP - 6

DP - 1979 Nov

TI - Testicular size and shape of 47,XYY and 47,XXY men in a double-blind,

double-matched population survey.

PG - 697-703

AB - This paper reports the testicular size and shape of 12 men with 47,XYY, 14 men

with 47,XXY, and 52 matched controls with 46,XY. The abnormal karyotypes were

identified in a systematic population search for XYY and XXY men. The subjects

and their matched controls were examined in a double-blind fashion. The testes of

the XYY men showed no significant differences from those of their XY controls for

volume or shape. This indicates that previous reports of abnormal testes in XYYs

reflect selection and publication bias and do not provide an accurate description

of the condition of 47,XYY men's testicles. As expected, the testes of the XXY

men were significantly smaller than those of their XY controls, and there was

also a difference in shape. However, the mean size in this sample of XXYs was

larger than in previous reports on Klinefelter syndrome patients, indicating that

previous reports on XXYs, identified in clinics for male hypogonadism and other

institutions, also suffered from selection bias.

FAU - Boisen, E

AU - Boisen E

LA - eng

PT - Clinical Trial

PT - Controlled Clinical Trial

PT - Journal Article

PT - Research Support, U.S. Gov't, P.H.S.

TA - Am J Hum Genet

JT - American journal of human genetics

JID - 0370475

SB - IM

MH - Adult

MH - Double-Blind Method
[truncated: 3,881,912 more chars]
